# Supplementary material for: The impacts of parity on lung function data (LFD) of healthy females aged 40 years and more issued from an upper middle income country (Algeria): A comparative study
Source: PLoS One. 2019 Nov 8;14(11):e0225067. doi: 10.1371/journal.pone.0225067 (PMC6839841; doi:10.1371/journal.pone.0225067)
Supplement: S1 File — (DOCX) [file pone.0225067.s001.docx]

**S1 File.** Entity,Code,Year,"Estimates, 1950 - 2015: Demographic Indicators - Total fertility (live births per woman) (live births per woman)". **Retrieved from: 'https://ourworldindata.org/fertility-rate': last access: Septembre 25^th^ 2019**

Afghanistan,AFG,1950,7.45

Afghanistan,AFG,1951,7.45

Afghanistan,AFG,1952,7.45

Afghanistan,AFG,1953,7.45

Afghanistan,AFG,1954,7.45

Afghanistan,AFG,1955,7.45

Afghanistan,AFG,1956,7.45

Afghanistan,AFG,1957,7.45

Afghanistan,AFG,1958,7.45

Afghanistan,AFG,1959,7.45

Afghanistan,AFG,1960,7.45

Afghanistan,AFG,1961,7.45

Afghanistan,AFG,1962,7.45

Afghanistan,AFG,1963,7.45

Afghanistan,AFG,1964,7.45

Afghanistan,AFG,1965,7.45

Afghanistan,AFG,1966,7.45

Afghanistan,AFG,1967,7.45

Afghanistan,AFG,1968,7.45

Afghanistan,AFG,1969,7.45

Afghanistan,AFG,1970,7.45

Afghanistan,AFG,1971,7.45

Afghanistan,AFG,1972,7.45

Afghanistan,AFG,1973,7.45

Afghanistan,AFG,1974,7.45

Afghanistan,AFG,1975,7.45

Afghanistan,AFG,1976,7.45

Afghanistan,AFG,1977,7.449

Afghanistan,AFG,1978,7.449

Afghanistan,AFG,1979,7.449

Afghanistan,AFG,1980,7.449

Afghanistan,AFG,1981,7.449

Afghanistan,AFG,1982,7.45

Afghanistan,AFG,1983,7.452

Afghanistan,AFG,1984,7.455

Afghanistan,AFG,1985,7.458

Afghanistan,AFG,1986,7.46

Afghanistan,AFG,1987,7.461

Afghanistan,AFG,1988,7.461

Afghanistan,AFG,1989,7.461

Afghanistan,AFG,1990,7.466

Afghanistan,AFG,1991,7.479

Afghanistan,AFG,1992,7.502

Afghanistan,AFG,1993,7.535

Afghanistan,AFG,1994,7.572

Afghanistan,AFG,1995,7.606

Afghanistan,AFG,1996,7.63

Afghanistan,AFG,1997,7.635

Afghanistan,AFG,1998,7.616

Afghanistan,AFG,1999,7.569

Afghanistan,AFG,2000,7.494

Afghanistan,AFG,2001,7.392

Afghanistan,AFG,2002,7.271

Afghanistan,AFG,2003,7.136

Afghanistan,AFG,2004,6.988

Afghanistan,AFG,2005,6.827

Afghanistan,AFG,2006,6.651

Afghanistan,AFG,2007,6.46

Afghanistan,AFG,2008,6.254

Afghanistan,AFG,2009,6.038

Afghanistan,AFG,2010,5.816

Afghanistan,AFG,2011,5.595

Afghanistan,AFG,2012,5.38

Afghanistan,AFG,2013,5.174

Afghanistan,AFG,2014,4.981

Afghanistan,AFG,2015,4.802

Africa,,1950,6.611

Africa,,1951,6.612

Africa,,1952,6.615

Africa,,1953,6.621

Africa,,1954,6.628

Africa,,1955,6.637

Africa,,1956,6.647

Africa,,1957,6.659

Africa,,1958,6.671

Africa,,1959,6.685

Africa,,1960,6.697

Africa,,1961,6.708

Africa,,1962,6.717

Africa,,1963,6.723

Africa,,1964,6.726

Africa,,1965,6.726

Africa,,1966,6.725

Africa,,1967,6.723

Africa,,1968,6.721

Africa,,1969,6.719

Africa,,1970,6.717

Africa,,1971,6.715

Africa,,1972,6.711

Africa,,1973,6.706

Africa,,1974,6.698

Africa,,1975,6.686

Africa,,1976,6.672

Africa,,1977,6.655

Africa,,1978,6.635

Africa,,1979,6.61

Africa,,1980,6.582

Africa,,1981,6.548

Africa,,1982,6.509

Africa,,1983,6.464

Africa,,1984,6.413

Africa,,1985,6.355

Africa,,1986,6.288

Africa,,1987,6.214

Africa,,1988,6.132

Africa,,1989,6.044

Africa,,1990,5.953

Africa,,1991,5.86

Africa,,1992,5.768

Africa,,1993,5.679

Africa,,1994,5.594

Africa,,1995,5.514

Africa,,1996,5.442

Africa,,1997,5.374

Africa,,1998,5.311

Africa,,1999,5.253

Africa,,2000,5.199

Africa,,2001,5.15

Africa,,2002,5.103

Africa,,2003,5.06

Africa,,2004,5.019

Africa,,2005,4.98

Africa,,2006,4.944

Africa,,2007,4.911

Africa,,2008,4.879

Africa,,2009,4.848

Africa,,2010,4.815

Africa,,2011,4.779

Africa,,2012,4.738

Africa,,2013,4.692

Africa,,2014,4.641

Africa,,2015,4.585

Albania,ALB,1950,5.963

Albania,ALB,1951,6.041

Albania,ALB,1952,6.186

Albania,ALB,1953,6.308

Albania,ALB,1954,6.408

Albania,ALB,1955,6.484

Albania,ALB,1956,6.537

Albania,ALB,1957,6.566

Albania,ALB,1958,6.569

Albania,ALB,1959,6.544

Albania,ALB,1960,6.489

Albania,ALB,1961,6.401

Albania,ALB,1962,6.282

Albania,ALB,1963,6.133

Albania,ALB,1964,5.96

Albania,ALB,1965,5.773

Albania,ALB,1966,5.581

Albania,ALB,1967,5.394

Albania,ALB,1968,5.218

Albania,ALB,1969,5.057

Albania,ALB,1970,4.91

Albania,ALB,1971,4.775

Albania,ALB,1972,4.642

Albania,ALB,1973,4.509

Albania,ALB,1974,4.373

Albania,ALB,1975,4.235

Albania,ALB,1976,4.099

Albania,ALB,1977,3.966

Albania,ALB,1978,3.841

Albania,ALB,1979,3.725

Albania,ALB,1980,3.621

Albania,ALB,1981,3.53

Albania,ALB,1982,3.452

Albania,ALB,1983,3.383

Albania,ALB,1984,3.323

Albania,ALB,1985,3.269

Albania,ALB,1986,3.217

Albania,ALB,1987,3.164

Albania,ALB,1988,3.108

Albania,ALB,1989,3.046

Albania,ALB,1990,2.978

Albania,ALB,1991,2.905

Albania,ALB,1992,2.829

Albania,ALB,1993,2.751

Albania,ALB,1994,2.672

Albania,ALB,1995,2.591

Albania,ALB,1996,2.507

Albania,ALB,1997,2.422

Albania,ALB,1998,2.334

Albania,ALB,1999,2.246

Albania,ALB,2000,2.157

Albania,ALB,2001,2.068

Albania,ALB,2002,1.981

Albania,ALB,2003,1.897

Albania,ALB,2004,1.821

Albania,ALB,2005,1.754

Albania,ALB,2006,1.703

Albania,ALB,2007,1.668

Albania,ALB,2008,1.65

Albania,ALB,2009,1.646

Albania,ALB,2010,1.653

Albania,ALB,2011,1.668

Albania,ALB,2012,1.685

Albania,ALB,2013,1.7

Albania,ALB,2014,1.71

Albania,ALB,2015,1.714

Algeria,DZA,1950,7.287

Algeria,DZA,1951,7.279

Algeria,DZA,1952,7.27

Algeria,DZA,1953,7.271

Algeria,DZA,1954,7.282

Algeria,DZA,1955,7.303

Algeria,DZA,1956,7.335

Algeria,DZA,1957,7.375

Algeria,DZA,1958,7.421

Algeria,DZA,1959,7.472

Algeria,DZA,1960,7.524

Algeria,DZA,1961,7.573

Algeria,DZA,1962,7.614

Algeria,DZA,1963,7.646

Algeria,DZA,1964,7.665

Algeria,DZA,1965,7.675

Algeria,DZA,1966,7.676

Algeria,DZA,1967,7.672

Algeria,DZA,1968,7.666

Algeria,DZA,1969,7.656

Algeria,DZA,1970,7.643

Algeria,DZA,1971,7.624

Algeria,DZA,1972,7.597

Algeria,DZA,1973,7.558

Algeria,DZA,1974,7.505

Algeria,DZA,1975,7.434

Algeria,DZA,1976,7.344

Algeria,DZA,1977,7.234

Algeria,DZA,1978,7.105

Algeria,DZA,1979,6.957

Algeria,DZA,1980,6.794

Algeria,DZA,1981,6.617

Algeria,DZA,1982,6.43

Algeria,DZA,1983,6.237

Algeria,DZA,1984,6.038

Algeria,DZA,1985,5.835

Algeria,DZA,1986,5.627

Algeria,DZA,1987,5.412

Algeria,DZA,1988,5.191

Algeria,DZA,1989,4.962

Algeria,DZA,1990,4.726

Algeria,DZA,1991,4.479

Algeria,DZA,1992,4.223

Algeria,DZA,1993,3.962

Algeria,DZA,1994,3.702

Algeria,DZA,1995,3.448

Algeria,DZA,1996,3.208

Algeria,DZA,1997,2.988

Algeria,DZA,1998,2.796

Algeria,DZA,1999,2.635

Algeria,DZA,2000,2.514

Algeria,DZA,2001,2.438

Algeria,DZA,2002,2.403

Algeria,DZA,2003,2.405

Algeria,DZA,2004,2.438

Algeria,DZA,2005,2.497

Algeria,DZA,2006,2.575

Algeria,DZA,2007,2.662

Algeria,DZA,2008,2.749

Algeria,DZA,2009,2.828

Algeria,DZA,2010,2.889

Algeria,DZA,2011,2.925

Algeria,DZA,2012,2.936

Algeria,DZA,2013,2.924

Algeria,DZA,2014,2.89

Algeria,DZA,2015,2.839

Angola,AGO,1950,7.342

Angola,AGO,1951,7.326

Angola,AGO,1952,7.298

Angola,AGO,1953,7.283

Angola,AGO,1954,7.281

Angola,AGO,1955,7.29

Angola,AGO,1956,7.312

Angola,AGO,1957,7.344

Angola,AGO,1958,7.384

Angola,AGO,1959,7.431

Angola,AGO,1960,7.478

Angola,AGO,1961,7.524

Angola,AGO,1962,7.563

Angola,AGO,1963,7.592

Angola,AGO,1964,7.611

Angola,AGO,1965,7.619

Angola,AGO,1966,7.618

Angola,AGO,1967,7.613

Angola,AGO,1968,7.608

Angola,AGO,1969,7.604

Angola,AGO,1970,7.601

Angola,AGO,1971,7.603

Angola,AGO,1972,7.606

Angola,AGO,1973,7.611

Angola,AGO,1974,7.614

Angola,AGO,1975,7.615

Angola,AGO,1976,7.609

Angola,AGO,1977,7.594

Angola,AGO,1978,7.571

Angola,AGO,1979,7.54

Angola,AGO,1980,7.504

Angola,AGO,1981,7.469

Angola,AGO,1982,7.438

Angola,AGO,1983,7.413

Angola,AGO,1984,7.394

Angola,AGO,1985,7.38

Angola,AGO,1986,7.366

Angola,AGO,1987,7.349

Angola,AGO,1988,7.324

Angola,AGO,1989,7.291

Angola,AGO,1990,7.247

Angola,AGO,1991,7.193

Angola,AGO,1992,7.13

Angola,AGO,1993,7.063

Angola,AGO,1994,6.992

Angola,AGO,1995,6.922

Angola,AGO,1996,6.854

Angola,AGO,1997,6.791

Angola,AGO,1998,6.734

Angola,AGO,1999,6.683

Angola,AGO,2000,6.639

Angola,AGO,2001,6.602

Angola,AGO,2002,6.568

Angola,AGO,2003,6.536

Angola,AGO,2004,6.502

Angola,AGO,2005,6.465

Angola,AGO,2006,6.42

Angola,AGO,2007,6.368

Angola,AGO,2008,6.307

Angola,AGO,2009,6.238

Angola,AGO,2010,6.162

Angola,AGO,2011,6.082

Angola,AGO,2012,6

Angola,AGO,2013,5.92

Angola,AGO,2014,5.841

Angola,AGO,2015,5.766

Antigua and Barbuda,ATG,1950,4.446

Antigua and Barbuda,ATG,1951,4.464

Antigua and Barbuda,ATG,1952,4.496

Antigua and Barbuda,ATG,1953,4.518

Antigua and Barbuda,ATG,1954,4.531

Antigua and Barbuda,ATG,1955,4.534

Antigua and Barbuda,ATG,1956,4.528

Antigua and Barbuda,ATG,1957,4.513

Antigua and Barbuda,ATG,1958,4.49

Antigua and Barbuda,ATG,1959,4.46

Antigua and Barbuda,ATG,1960,4.425

Antigua and Barbuda,ATG,1961,4.386

Antigua and Barbuda,ATG,1962,4.344

Antigua and Barbuda,ATG,1963,4.299

Antigua and Barbuda,ATG,1964,4.25

Antigua and Barbuda,ATG,1965,4.193

Antigua and Barbuda,ATG,1966,4.125

Antigua and Barbuda,ATG,1967,4.042

Antigua and Barbuda,ATG,1968,3.942

Antigua and Barbuda,ATG,1969,3.824

Antigua and Barbuda,ATG,1970,3.684

Antigua and Barbuda,ATG,1971,3.517

Antigua and Barbuda,ATG,1972,3.328

Antigua and Barbuda,ATG,1973,3.122

Antigua and Barbuda,ATG,1974,2.908

Antigua and Barbuda,ATG,1975,2.701

Antigua and Barbuda,ATG,1976,2.514

Antigua and Barbuda,ATG,1977,2.358

Antigua and Barbuda,ATG,1978,2.239

Antigua and Barbuda,ATG,1979,2.159

Antigua and Barbuda,ATG,1980,2.115

Antigua and Barbuda,ATG,1981,2.098

Antigua and Barbuda,ATG,1982,2.096

Antigua and Barbuda,ATG,1983,2.098

Antigua and Barbuda,ATG,1984,2.098

Antigua and Barbuda,ATG,1985,2.092

Antigua and Barbuda,ATG,1986,2.082

Antigua and Barbuda,ATG,1987,2.07

Antigua and Barbuda,ATG,1988,2.062

Antigua and Barbuda,ATG,1989,2.058

Antigua and Barbuda,ATG,1990,2.061

Antigua and Barbuda,ATG,1991,2.073

Antigua and Barbuda,ATG,1992,2.096

Antigua and Barbuda,ATG,1993,2.128

Antigua and Barbuda,ATG,1994,2.166

Antigua and Barbuda,ATG,1995,2.208

Antigua and Barbuda,ATG,1996,2.248

Antigua and Barbuda,ATG,1997,2.281

Antigua and Barbuda,ATG,1998,2.304

Antigua and Barbuda,ATG,1999,2.316

Antigua and Barbuda,ATG,2000,2.316

Antigua and Barbuda,ATG,2001,2.305

Antigua and Barbuda,ATG,2002,2.288

Antigua and Barbuda,ATG,2003,2.268

Antigua and Barbuda,ATG,2004,2.246

Antigua and Barbuda,ATG,2005,2.224

Antigua and Barbuda,ATG,2006,2.203

Antigua and Barbuda,ATG,2007,2.183

Antigua and Barbuda,ATG,2008,2.164

Antigua and Barbuda,ATG,2009,2.146

Antigua and Barbuda,ATG,2010,2.13

Antigua and Barbuda,ATG,2011,2.115

Antigua and Barbuda,ATG,2012,2.102

Antigua and Barbuda,ATG,2013,2.089

Antigua and Barbuda,ATG,2014,2.076

Antigua and Barbuda,ATG,2015,2.063

Argentina,ARG,1950,3.164

Argentina,ARG,1951,3.162

Argentina,ARG,1952,3.157

Argentina,ARG,1953,3.152

Argentina,ARG,1954,3.146

Argentina,ARG,1955,3.141

Argentina,ARG,1956,3.136

Argentina,ARG,1957,3.13

Argentina,ARG,1958,3.124

Argentina,ARG,1959,3.117

Argentina,ARG,1960,3.109

Argentina,ARG,1961,3.1

Argentina,ARG,1962,3.089

Argentina,ARG,1963,3.078

Argentina,ARG,1964,3.068

Argentina,ARG,1965,3.058

Argentina,ARG,1966,3.051

Argentina,ARG,1967,3.048

Argentina,ARG,1968,3.049

Argentina,ARG,1969,3.056

Argentina,ARG,1970,3.073

Argentina,ARG,1971,3.104

Argentina,ARG,1972,3.148

Argentina,ARG,1973,3.203

Argentina,ARG,1974,3.264

Argentina,ARG,1975,3.321

Argentina,ARG,1976,3.366

Argentina,ARG,1977,3.391

Argentina,ARG,1978,3.391

Argentina,ARG,1979,3.368

Argentina,ARG,1980,3.325

Argentina,ARG,1981,3.27

Argentina,ARG,1982,3.213

Argentina,ARG,1983,3.163

Argentina,ARG,1984,3.123

Argentina,ARG,1985,3.093

Argentina,ARG,1986,3.073

Argentina,ARG,1987,3.056

Argentina,ARG,1988,3.037

Argentina,ARG,1989,3.016

Argentina,ARG,1990,2.989

Argentina,ARG,1991,2.954

Argentina,ARG,1992,2.913

Argentina,ARG,1993,2.866

Argentina,ARG,1994,2.815

Argentina,ARG,1995,2.762

Argentina,ARG,1996,2.711

Argentina,ARG,1997,2.664

Argentina,ARG,1998,2.623

Argentina,ARG,1999,2.589

Argentina,ARG,2000,2.561

Argentina,ARG,2001,2.537

Argentina,ARG,2002,2.516

Argentina,ARG,2003,2.495

Argentina,ARG,2004,2.474

Argentina,ARG,2005,2.452

Argentina,ARG,2006,2.432

Argentina,ARG,2007,2.413

Argentina,ARG,2008,2.396

Argentina,ARG,2009,2.382

Argentina,ARG,2010,2.37

Argentina,ARG,2011,2.358

Argentina,ARG,2012,2.347

Argentina,ARG,2013,2.335

Argentina,ARG,2014,2.322

Argentina,ARG,2015,2.308

Armenia,ARM,1950,4.132

Armenia,ARM,1951,4.239

Armenia,ARM,1952,4.437

Armenia,ARM,1953,4.601

Armenia,ARM,1954,4.732

Armenia,ARM,1955,4.828

Armenia,ARM,1956,4.89

Armenia,ARM,1957,4.918

Armenia,ARM,1958,4.91

Armenia,ARM,1959,4.867

Armenia,ARM,1960,4.786

Armenia,ARM,1961,4.67

Armenia,ARM,1962,4.521

Armenia,ARM,1963,4.345

Armenia,ARM,1964,4.15

Armenia,ARM,1965,3.95

Armenia,ARM,1966,3.758

Armenia,ARM,1967,3.582

Armenia,ARM,1968,3.429

Armenia,ARM,1969,3.302

Armenia,ARM,1970,3.199

Armenia,ARM,1971,3.114

Armenia,ARM,1972,3.035

Armenia,ARM,1973,2.956

Armenia,ARM,1974,2.875

Armenia,ARM,1975,2.792

Armenia,ARM,1976,2.712

Armenia,ARM,1977,2.641

Armenia,ARM,1978,2.582

Armenia,ARM,1979,2.538

Armenia,ARM,1980,2.51

Armenia,ARM,1981,2.499

Armenia,ARM,1982,2.503

Armenia,ARM,1983,2.517

Armenia,ARM,1984,2.538

Armenia,ARM,1985,2.559

Armenia,ARM,1986,2.578

Armenia,ARM,1987,2.591

Armenia,ARM,1988,2.592

Armenia,ARM,1989,2.578

Armenia,ARM,1990,2.544

Armenia,ARM,1991,2.484

Armenia,ARM,1992,2.4

Armenia,ARM,1993,2.297

Armenia,ARM,1994,2.179

Armenia,ARM,1995,2.056

Armenia,ARM,1996,1.938

Armenia,ARM,1997,1.832

Armenia,ARM,1998,1.747

Armenia,ARM,1999,1.685

Armenia,ARM,2000,1.648

Armenia,ARM,2001,1.635

Armenia,ARM,2002,1.637

Armenia,ARM,2003,1.648

Armenia,ARM,2004,1.665

Armenia,ARM,2005,1.681

Armenia,ARM,2006,1.694

Armenia,ARM,2007,1.702

Armenia,ARM,2008,1.706

Armenia,ARM,2009,1.703

Armenia,ARM,2010,1.693

Armenia,ARM,2011,1.68

Armenia,ARM,2012,1.664

Armenia,ARM,2013,1.648

Armenia,ARM,2014,1.634

Armenia,ARM,2015,1.622

Aruba,ABW,1950,5.801

Aruba,ABW,1951,5.767

Aruba,ABW,1952,5.695

Aruba,ABW,1953,5.616

Aruba,ABW,1954,5.53

Aruba,ABW,1955,5.436

Aruba,ABW,1956,5.334

Aruba,ABW,1957,5.223

Aruba,ABW,1958,5.102

Aruba,ABW,1959,4.969

Aruba,ABW,1960,4.82

Aruba,ABW,1961,4.655

Aruba,ABW,1962,4.471

Aruba,ABW,1963,4.271

Aruba,ABW,1964,4.059

Aruba,ABW,1965,3.842

Aruba,ABW,1966,3.625

Aruba,ABW,1967,3.417

Aruba,ABW,1968,3.226

Aruba,ABW,1969,3.054

Aruba,ABW,1970,2.908

Aruba,ABW,1971,2.788

Aruba,ABW,1972,2.691

Aruba,ABW,1973,2.613

Aruba,ABW,1974,2.552

Aruba,ABW,1975,2.506

Aruba,ABW,1976,2.472

Aruba,ABW,1977,2.446

Aruba,ABW,1978,2.425

Aruba,ABW,1979,2.408

Aruba,ABW,1980,2.392

Aruba,ABW,1981,2.377

Aruba,ABW,1982,2.364

Aruba,ABW,1983,2.353

Aruba,ABW,1984,2.342

Aruba,ABW,1985,2.332

Aruba,ABW,1986,2.32

Aruba,ABW,1987,2.307

Aruba,ABW,1988,2.291

Aruba,ABW,1989,2.272

Aruba,ABW,1990,2.249

Aruba,ABW,1991,2.221

Aruba,ABW,1992,2.187

Aruba,ABW,1993,2.149

Aruba,ABW,1994,2.108

Aruba,ABW,1995,2.064

Aruba,ABW,1996,2.021

Aruba,ABW,1997,1.978

Aruba,ABW,1998,1.939

Aruba,ABW,1999,1.903

Aruba,ABW,2000,1.872

Aruba,ABW,2001,1.846

Aruba,ABW,2002,1.823

Aruba,ABW,2003,1.803

Aruba,ABW,2004,1.787

Aruba,ABW,2005,1.774

Aruba,ABW,2006,1.766

Aruba,ABW,2007,1.763

Aruba,ABW,2008,1.764

Aruba,ABW,2009,1.769

Aruba,ABW,2010,1.776

Aruba,ABW,2011,1.783

Aruba,ABW,2012,1.791

Aruba,ABW,2013,1.796

Aruba,ABW,2014,1.8

Aruba,ABW,2015,1.801

Asia,,1950,6.028

Asia,,1951,5.957

Asia,,1952,5.828

Asia,,1953,5.725

Asia,,1954,5.649

Asia,,1955,5.598

Asia,,1956,5.573

Asia,,1957,5.571

Asia,,1958,5.59

Asia,,1959,5.627

Asia,,1960,5.675

Asia,,1961,5.729

Asia,,1962,5.779

Asia,,1963,5.82

Asia,,1964,5.843

Asia,,1965,5.842

Asia,,1966,5.813

Asia,,1967,5.757

Asia,,1968,5.676

Asia,,1969,5.571

Asia,,1970,5.44

Asia,,1971,5.285

Asia,,1972,5.111

Asia,,1973,4.923

Asia,,1974,4.729

Asia,,1975,4.539

Asia,,1976,4.36

Asia,,1977,4.198

Asia,,1978,4.057

Asia,,1979,3.939

Asia,,1980,3.846

Asia,,1981,3.778

Asia,,1982,3.728

Asia,,1983,3.688

Asia,,1984,3.652

Asia,,1985,3.614

Asia,,1986,3.564

Asia,,1987,3.501

Asia,,1988,3.421

Asia,,1989,3.327

Asia,,1990,3.22

Asia,,1991,3.106

Asia,,1992,2.993

Asia,,1993,2.887

Asia,,1994,2.791

Asia,,1995,2.709

Asia,,1996,2.641

Asia,,1997,2.585

Asia,,1998,2.537

Asia,,1999,2.498

Asia,,2000,2.465

Asia,,2001,2.437

Asia,,2002,2.414

Asia,,2003,2.392

Asia,,2004,2.372

Asia,,2005,2.352

Asia,,2006,2.331

Asia,,2007,2.31

Asia,,2008,2.289

Asia,,2009,2.269

Asia,,2010,2.249

Asia,,2011,2.23

Asia,,2012,2.213

Asia,,2013,2.198

Asia,,2014,2.184

Asia,,2015,2.173

Australia,AUS,1950,3.005

Australia,AUS,1951,3.056

Australia,AUS,1952,3.15

Australia,AUS,1953,3.231

Australia,AUS,1954,3.297

Australia,AUS,1955,3.349

Australia,AUS,1956,3.386

Australia,AUS,1957,3.409

Australia,AUS,1958,3.418

Australia,AUS,1959,3.411

Australia,AUS,1960,3.389

Australia,AUS,1961,3.353

Australia,AUS,1962,3.303

Australia,AUS,1963,3.24

Australia,AUS,1964,3.168

Australia,AUS,1965,3.09

Australia,AUS,1966,3.011

Australia,AUS,1967,2.933

Australia,AUS,1968,2.858

Australia,AUS,1969,2.786

Australia,AUS,1970,2.713

Australia,AUS,1971,2.636

Australia,AUS,1972,2.549

Australia,AUS,1973,2.452

Australia,AUS,1974,2.348

Australia,AUS,1975,2.242

Australia,AUS,1976,2.142

Australia,AUS,1977,2.057

Australia,AUS,1978,1.989

Australia,AUS,1979,1.941

Australia,AUS,1980,1.912

Australia,AUS,1981,1.898

Australia,AUS,1982,1.891

Australia,AUS,1983,1.887

Australia,AUS,1984,1.883

Australia,AUS,1985,1.877

Australia,AUS,1986,1.87

Australia,AUS,1987,1.865

Australia,AUS,1988,1.863

Australia,AUS,1989,1.863

Australia,AUS,1990,1.863

Australia,AUS,1991,1.861

Australia,AUS,1992,1.857

Australia,AUS,1993,1.85

Australia,AUS,1994,1.839

Australia,AUS,1995,1.825

Australia,AUS,1996,1.809

Australia,AUS,1997,1.793

Australia,AUS,1998,1.778

Australia,AUS,1999,1.767

Australia,AUS,2000,1.762

Australia,AUS,2001,1.766

Australia,AUS,2002,1.781

Australia,AUS,2003,1.804

Australia,AUS,2004,1.834

Australia,AUS,2005,1.867

Australia,AUS,2006,1.898

Australia,AUS,2007,1.923

Australia,AUS,2008,1.938

Australia,AUS,2009,1.942

Australia,AUS,2010,1.936

Australia,AUS,2011,1.922

Australia,AUS,2012,1.904

Australia,AUS,2013,1.886

Australia,AUS,2014,1.87

Australia,AUS,2015,1.856

Australia/New Zealand,,1950,3.075

Australia/New Zealand,,1951,3.133

Australia/New Zealand,,1952,3.24

Australia/New Zealand,,1953,3.331

Australia/New Zealand,,1954,3.406

Australia/New Zealand,,1955,3.464

Australia/New Zealand,,1956,3.506

Australia/New Zealand,,1957,3.531

Australia/New Zealand,,1958,3.54

Australia/New Zealand,,1959,3.532

Australia/New Zealand,,1960,3.508

Australia/New Zealand,,1961,3.468

Australia/New Zealand,,1962,3.413

Australia/New Zealand,,1963,3.346

Australia/New Zealand,,1964,3.27

Australia/New Zealand,,1965,3.187

Australia/New Zealand,,1966,3.103

Australia/New Zealand,,1967,3.02

Australia/New Zealand,,1968,2.94

Australia/New Zealand,,1969,2.862

Australia/New Zealand,,1970,2.784

Australia/New Zealand,,1971,2.7

Australia/New Zealand,,1972,2.608

Australia/New Zealand,,1973,2.505

Australia/New Zealand,,1974,2.396

Australia/New Zealand,,1975,2.286

Australia/New Zealand,,1976,2.182

Australia/New Zealand,,1977,2.091

Australia/New Zealand,,1978,2.018

Australia/New Zealand,,1979,1.965

Australia/New Zealand,,1980,1.932

Australia/New Zealand,,1981,1.914

Australia/New Zealand,,1982,1.906

Australia/New Zealand,,1983,1.902

Australia/New Zealand,,1984,1.899

Australia/New Zealand,,1985,1.896

Australia/New Zealand,,1986,1.893

Australia/New Zealand,,1987,1.892

Australia/New Zealand,,1988,1.893

Australia/New Zealand,,1989,1.895

Australia/New Zealand,,1990,1.897

Australia/New Zealand,,1991,1.896

Australia/New Zealand,,1992,1.892

Australia/New Zealand,,1993,1.884

Australia/New Zealand,,1994,1.872

Australia/New Zealand,,1995,1.857

Australia/New Zealand,,1996,1.84

Australia/New Zealand,,1997,1.822

Australia/New Zealand,,1998,1.807

Australia/New Zealand,,1999,1.795

Australia/New Zealand,,2000,1.791

Australia/New Zealand,,2001,1.796

Australia/New Zealand,,2002,1.811

Australia/New Zealand,,2003,1.835

Australia/New Zealand,,2004,1.866

Australia/New Zealand,,2005,1.899

Australia/New Zealand,,2006,1.93

Australia/New Zealand,,2007,1.955

Australia/New Zealand,,2008,1.969

Australia/New Zealand,,2009,1.972

Australia/New Zealand,,2010,1.965

Australia/New Zealand,,2011,1.95

Australia/New Zealand,,2012,1.931

Australia/New Zealand,,2013,1.912

Australia/New Zealand,,2014,1.895

Australia/New Zealand,,2015,1.881

Austria,AUT,1950,1.866

Austria,AUT,1951,1.93

Austria,AUT,1952,2.053

Austria,AUT,1953,2.168

Austria,AUT,1954,2.275

Austria,AUT,1955,2.374

Austria,AUT,1956,2.464

Austria,AUT,1957,2.545

Austria,AUT,1958,2.616

Austria,AUT,1959,2.677

Austria,AUT,1960,2.725

Austria,AUT,1961,2.759

Austria,AUT,1962,2.778

Austria,AUT,1963,2.78

Austria,AUT,1964,2.765

Austria,AUT,1965,2.731

Austria,AUT,1966,2.677

Austria,AUT,1967,2.606

Austria,AUT,1968,2.52

Austria,AUT,1969,2.422

Austria,AUT,1970,2.317

Austria,AUT,1971,2.207

Austria,AUT,1972,2.098

Austria,AUT,1973,1.992

Austria,AUT,1974,1.894

Austria,AUT,1975,1.809

Austria,AUT,1976,1.74

Austria,AUT,1977,1.687

Austria,AUT,1978,1.648

Austria,AUT,1979,1.621

Austria,AUT,1980,1.604

Austria,AUT,1981,1.591

Austria,AUT,1982,1.577

Austria,AUT,1983,1.56

Austria,AUT,1984,1.538

Austria,AUT,1985,1.514

Austria,AUT,1986,1.491

Austria,AUT,1987,1.473

Austria,AUT,1988,1.463

Austria,AUT,1989,1.46

Austria,AUT,1990,1.462

Austria,AUT,1991,1.466

Austria,AUT,1992,1.467

Austria,AUT,1993,1.463

Austria,AUT,1994,1.453

Austria,AUT,1995,1.438

Austria,AUT,1996,1.421

Austria,AUT,1997,1.404

Austria,AUT,1998,1.39

Austria,AUT,1999,1.381

Austria,AUT,2000,1.375

Austria,AUT,2001,1.374

Austria,AUT,2002,1.375

Austria,AUT,2003,1.377

Austria,AUT,2004,1.381

Austria,AUT,2005,1.385

Austria,AUT,2006,1.39

Austria,AUT,2007,1.396

Austria,AUT,2008,1.403

Austria,AUT,2009,1.411

Austria,AUT,2010,1.421

Austria,AUT,2011,1.431

Austria,AUT,2012,1.443

Austria,AUT,2013,1.455

Austria,AUT,2014,1.467

Austria,AUT,2015,1.48

Azerbaijan,AZE,1950,5.075

Azerbaijan,AZE,1951,5.101

Azerbaijan,AZE,1952,5.158

Azerbaijan,AZE,1953,5.226

Azerbaijan,AZE,1954,5.304

Azerbaijan,AZE,1955,5.393

Azerbaijan,AZE,1956,5.49

Azerbaijan,AZE,1957,5.593

Azerbaijan,AZE,1958,5.697

Azerbaijan,AZE,1959,5.795

Azerbaijan,AZE,1960,5.878

Azerbaijan,AZE,1961,5.937

Azerbaijan,AZE,1962,5.961

Azerbaijan,AZE,1963,5.945

Azerbaijan,AZE,1964,5.888

Azerbaijan,AZE,1965,5.792

Azerbaijan,AZE,1966,5.663

Azerbaijan,AZE,1967,5.512

Azerbaijan,AZE,1968,5.351

Azerbaijan,AZE,1969,5.185

Azerbaijan,AZE,1970,5.018

Azerbaijan,AZE,1971,4.85

Azerbaijan,AZE,1972,4.681

Azerbaijan,AZE,1973,4.51

Azerbaijan,AZE,1974,4.341

Azerbaijan,AZE,1975,4.176

Azerbaijan,AZE,1976,4.018

Azerbaijan,AZE,1977,3.869

Azerbaijan,AZE,1978,3.73

Azerbaijan,AZE,1979,3.605

Azerbaijan,AZE,1980,3.497

Azerbaijan,AZE,1981,3.41

Azerbaijan,AZE,1982,3.343

Azerbaijan,AZE,1983,3.293

Azerbaijan,AZE,1984,3.259

Azerbaijan,AZE,1985,3.235

Azerbaijan,AZE,1986,3.217

Azerbaijan,AZE,1987,3.199

Azerbaijan,AZE,1988,3.175

Azerbaijan,AZE,1989,3.14

Azerbaijan,AZE,1990,3.09

Azerbaijan,AZE,1991,3.019

Azerbaijan,AZE,1992,2.929

Azerbaijan,AZE,1993,2.823

Azerbaijan,AZE,1994,2.705

Azerbaijan,AZE,1995,2.579

Azerbaijan,AZE,1996,2.45

Azerbaijan,AZE,1997,2.326

Azerbaijan,AZE,1998,2.213

Azerbaijan,AZE,1999,2.114

Azerbaijan,AZE,2000,2.031

Azerbaijan,AZE,2001,1.964

Azerbaijan,AZE,2002,1.91

Azerbaijan,AZE,2003,1.867

Azerbaijan,AZE,2004,1.835

Azerbaijan,AZE,2005,1.818

Azerbaijan,AZE,2006,1.817

Azerbaijan,AZE,2007,1.835

Azerbaijan,AZE,2008,1.868

Azerbaijan,AZE,2009,1.913

Azerbaijan,AZE,2010,1.964

Azerbaijan,AZE,2011,2.012

Azerbaijan,AZE,2012,2.053

Azerbaijan,AZE,2013,2.08

Azerbaijan,AZE,2014,2.093

Azerbaijan,AZE,2015,2.092

Bahamas,BHS,1950,3.978

Bahamas,BHS,1951,3.991

Bahamas,BHS,1952,4.021

Bahamas,BHS,1953,4.062

Bahamas,BHS,1954,4.115

Bahamas,BHS,1955,4.178

Bahamas,BHS,1956,4.248

Bahamas,BHS,1957,4.323

Bahamas,BHS,1958,4.397

Bahamas,BHS,1959,4.458

Bahamas,BHS,1960,4.495

Bahamas,BHS,1961,4.496

Bahamas,BHS,1962,4.45

Bahamas,BHS,1963,4.356

Bahamas,BHS,1964,4.22

Bahamas,BHS,1965,4.057

Bahamas,BHS,1966,3.893

Bahamas,BHS,1967,3.75

Bahamas,BHS,1968,3.641

Bahamas,BHS,1969,3.571

Bahamas,BHS,1970,3.531

Bahamas,BHS,1971,3.504

Bahamas,BHS,1972,3.469

Bahamas,BHS,1973,3.411

Bahamas,BHS,1974,3.33

Bahamas,BHS,1975,3.234

Bahamas,BHS,1976,3.139

Bahamas,BHS,1977,3.062

Bahamas,BHS,1978,3.012

Bahamas,BHS,1979,2.99

Bahamas,BHS,1980,2.989

Bahamas,BHS,1981,2.994

Bahamas,BHS,1982,2.989

Bahamas,BHS,1983,2.965

Bahamas,BHS,1984,2.919

Bahamas,BHS,1985,2.857

Bahamas,BHS,1986,2.789

Bahamas,BHS,1987,2.728

Bahamas,BHS,1988,2.683

Bahamas,BHS,1989,2.654

Bahamas,BHS,1990,2.639

Bahamas,BHS,1991,2.631

Bahamas,BHS,1992,2.621

Bahamas,BHS,1993,2.601

Bahamas,BHS,1994,2.566

Bahamas,BHS,1995,2.514

Bahamas,BHS,1996,2.443

Bahamas,BHS,1997,2.357

Bahamas,BHS,1998,2.263

Bahamas,BHS,1999,2.165

Bahamas,BHS,2000,2.071

Bahamas,BHS,2001,1.99

Bahamas,BHS,2002,1.927

Bahamas,BHS,2003,1.885

Bahamas,BHS,2004,1.863

Bahamas,BHS,2005,1.859

Bahamas,BHS,2006,1.865

Bahamas,BHS,2007,1.874

Bahamas,BHS,2008,1.879

Bahamas,BHS,2009,1.876

Bahamas,BHS,2010,1.865

Bahamas,BHS,2011,1.847

Bahamas,BHS,2012,1.827

Bahamas,BHS,2013,1.808

Bahamas,BHS,2014,1.792

Bahamas,BHS,2015,1.778

Bahrain,BHR,1950,7.029

Bahrain,BHR,1951,7.008

Bahrain,BHR,1952,6.973

Bahrain,BHR,1953,6.949

Bahrain,BHR,1954,6.938

Bahrain,BHR,1955,6.938

Bahrain,BHR,1956,6.951

Bahrain,BHR,1957,6.974

Bahrain,BHR,1958,7.006

Bahrain,BHR,1959,7.045

Bahrain,BHR,1960,7.087

Bahrain,BHR,1961,7.129

Bahrain,BHR,1962,7.163

Bahrain,BHR,1963,7.185

Bahrain,BHR,1964,7.188

Bahrain,BHR,1965,7.162

Bahrain,BHR,1966,7.099

Bahrain,BHR,1967,6.997

Bahrain,BHR,1968,6.86

Bahrain,BHR,1969,6.692

Bahrain,BHR,1970,6.501

Bahrain,BHR,1971,6.297

Bahrain,BHR,1972,6.094

Bahrain,BHR,1973,5.899

Bahrain,BHR,1974,5.721

Bahrain,BHR,1975,5.56

Bahrain,BHR,1976,5.417

Bahrain,BHR,1977,5.285

Bahrain,BHR,1978,5.158

Bahrain,BHR,1979,5.036

Bahrain,BHR,1980,4.916

Bahrain,BHR,1981,4.8

Bahrain,BHR,1982,4.688

Bahrain,BHR,1983,4.578

Bahrain,BHR,1984,4.47

Bahrain,BHR,1985,4.36

Bahrain,BHR,1986,4.245

Bahrain,BHR,1987,4.124

Bahrain,BHR,1988,3.997

Bahrain,BHR,1989,3.866

Bahrain,BHR,1990,3.732

Bahrain,BHR,1991,3.599

Bahrain,BHR,1992,3.471

Bahrain,BHR,1993,3.352

Bahrain,BHR,1994,3.244

Bahrain,BHR,1995,3.147

Bahrain,BHR,1996,3.062

Bahrain,BHR,1997,2.987

Bahrain,BHR,1998,2.919

Bahrain,BHR,1999,2.856

Bahrain,BHR,2000,2.795

Bahrain,BHR,2001,2.731

Bahrain,BHR,2002,2.663

Bahrain,BHR,2003,2.59

Bahrain,BHR,2004,2.514

Bahrain,BHR,2005,2.436

Bahrain,BHR,2006,2.363

Bahrain,BHR,2007,2.297

Bahrain,BHR,2008,2.241

Bahrain,BHR,2009,2.197

Bahrain,BHR,2010,2.164

Bahrain,BHR,2011,2.139

Bahrain,BHR,2012,2.118

Bahrain,BHR,2013,2.098

Bahrain,BHR,2014,2.078

Bahrain,BHR,2015,2.056

Bangladesh,BGD,1950,6.233

Bangladesh,BGD,1951,6.266

Bangladesh,BGD,1952,6.331

Bangladesh,BGD,1953,6.392

Bangladesh,BGD,1954,6.45

Bangladesh,BGD,1955,6.504

Bangladesh,BGD,1956,6.555

Bangladesh,BGD,1957,6.602

Bangladesh,BGD,1958,6.646

Bangladesh,BGD,1959,6.687

Bangladesh,BGD,1960,6.725

Bangladesh,BGD,1961,6.761

Bangladesh,BGD,1962,6.794

Bangladesh,BGD,1963,6.825

Bangladesh,BGD,1964,6.853

Bangladesh,BGD,1965,6.878

Bangladesh,BGD,1966,6.901

Bangladesh,BGD,1967,6.92

Bangladesh,BGD,1968,6.935

Bangladesh,BGD,1969,6.945

Bangladesh,BGD,1970,6.947

Bangladesh,BGD,1971,6.942

Bangladesh,BGD,1972,6.928

Bangladesh,BGD,1973,6.904

Bangladesh,BGD,1974,6.869

Bangladesh,BGD,1975,6.821

Bangladesh,BGD,1976,6.758

Bangladesh,BGD,1977,6.681

Bangladesh,BGD,1978,6.59

Bangladesh,BGD,1979,6.483

Bangladesh,BGD,1980,6.359

Bangladesh,BGD,1981,6.217

Bangladesh,BGD,1982,6.058

Bangladesh,BGD,1983,5.884

Bangladesh,BGD,1984,5.698

Bangladesh,BGD,1985,5.501

Bangladesh,BGD,1986,5.298

Bangladesh,BGD,1987,5.092

Bangladesh,BGD,1988,4.887

Bangladesh,BGD,1989,4.686

Bangladesh,BGD,1990,4.494

Bangladesh,BGD,1991,4.314

Bangladesh,BGD,1992,4.146

Bangladesh,BGD,1993,3.99

Bangladesh,BGD,1994,3.846

Bangladesh,BGD,1995,3.713

Bangladesh,BGD,1996,3.592

Bangladesh,BGD,1997,3.479

Bangladesh,BGD,1998,3.372

Bangladesh,BGD,1999,3.27

Bangladesh,BGD,2000,3.17

Bangladesh,BGD,2001,3.071

Bangladesh,BGD,2002,2.973

Bangladesh,BGD,2003,2.876

Bangladesh,BGD,2004,2.782

Bangladesh,BGD,2005,2.69

Bangladesh,BGD,2006,2.603

Bangladesh,BGD,2007,2.522

Bangladesh,BGD,2008,2.449

Bangladesh,BGD,2009,2.384

Bangladesh,BGD,2010,2.328

Bangladesh,BGD,2011,2.279

Bangladesh,BGD,2012,2.236

Bangladesh,BGD,2013,2.199

Bangladesh,BGD,2014,2.165

Bangladesh,BGD,2015,2.133

Barbados,BRB,1950,4.516

Barbados,BRB,1951,4.485

Barbados,BRB,1952,4.429

Barbados,BRB,1953,4.386

Barbados,BRB,1954,4.355

Barbados,BRB,1955,4.336

Barbados,BRB,1956,4.327

Barbados,BRB,1957,4.328

Barbados,BRB,1958,4.333

Barbados,BRB,1959,4.337

Barbados,BRB,1960,4.333

Barbados,BRB,1961,4.313

Barbados,BRB,1962,4.269

Barbados,BRB,1963,4.196

Barbados,BRB,1964,4.094

Barbados,BRB,1965,3.963

Barbados,BRB,1966,3.807

Barbados,BRB,1967,3.637

Barbados,BRB,1968,3.46

Barbados,BRB,1969,3.284

Barbados,BRB,1970,3.113

Barbados,BRB,1971,2.949

Barbados,BRB,1972,2.794

Barbados,BRB,1973,2.648

Barbados,BRB,1974,2.513

Barbados,BRB,1975,2.393

Barbados,BRB,1976,2.288

Barbados,BRB,1977,2.198

Barbados,BRB,1978,2.121

Barbados,BRB,1979,2.057

Barbados,BRB,1980,2.004

Barbados,BRB,1981,1.96

Barbados,BRB,1982,1.922

Barbados,BRB,1983,1.889

Barbados,BRB,1984,1.858

Barbados,BRB,1985,1.83

Barbados,BRB,1986,1.805

Barbados,BRB,1987,1.782

Barbados,BRB,1988,1.765

Barbados,BRB,1989,1.75

Barbados,BRB,1990,1.74

Barbados,BRB,1991,1.733

Barbados,BRB,1992,1.729

Barbados,BRB,1993,1.728

Barbados,BRB,1994,1.729

Barbados,BRB,1995,1.73

Barbados,BRB,1996,1.733

Barbados,BRB,1997,1.736

Barbados,BRB,1998,1.739

Barbados,BRB,1999,1.742

Barbados,BRB,2000,1.744

Barbados,BRB,2001,1.746

Barbados,BRB,2002,1.749

Barbados,BRB,2003,1.752

Barbados,BRB,2004,1.755

Barbados,BRB,2005,1.759

Barbados,BRB,2006,1.763

Barbados,BRB,2007,1.768

Barbados,BRB,2008,1.772

Barbados,BRB,2009,1.777

Barbados,BRB,2010,1.781

Barbados,BRB,2011,1.785

Barbados,BRB,2012,1.788

Barbados,BRB,2013,1.791

Barbados,BRB,2014,1.794

Barbados,BRB,2015,1.796

Belarus,BLR,1950,2.502

Belarus,BLR,1951,2.534

Belarus,BLR,1952,2.593

Belarus,BLR,1953,2.642

Belarus,BLR,1954,2.681

Belarus,BLR,1955,2.71

Belarus,BLR,1956,2.728

Belarus,BLR,1957,2.736

Belarus,BLR,1958,2.733

Belarus,BLR,1959,2.719

Belarus,BLR,1960,2.693

Belarus,BLR,1961,2.656

Belarus,BLR,1962,2.608

Belarus,BLR,1963,2.551

Belarus,BLR,1964,2.49

Belarus,BLR,1965,2.428

Belarus,BLR,1966,2.372

Belarus,BLR,1967,2.326

Belarus,BLR,1968,2.291

Belarus,BLR,1969,2.268

Belarus,BLR,1970,2.254

Belarus,BLR,1971,2.244

Belarus,BLR,1972,2.232

Belarus,BLR,1973,2.216

Belarus,BLR,1974,2.194

Belarus,BLR,1975,2.167

Belarus,BLR,1976,2.139

Belarus,BLR,1977,2.114

Belarus,BLR,1978,2.095

Belarus,BLR,1979,2.083

Belarus,BLR,1980,2.079

Belarus,BLR,1981,2.083

Belarus,BLR,1982,2.092

Belarus,BLR,1983,2.102

Belarus,BLR,1984,2.11

Belarus,BLR,1985,2.112

Belarus,BLR,1986,2.1

Belarus,BLR,1987,2.074

Belarus,BLR,1988,2.032

Belarus,BLR,1989,1.974

Belarus,BLR,1990,1.902

Belarus,BLR,1991,1.818

Belarus,BLR,1992,1.728

Belarus,BLR,1993,1.636

Belarus,BLR,1994,1.549

Belarus,BLR,1995,1.468

Belarus,BLR,1996,1.399

Belarus,BLR,1997,1.341

Belarus,BLR,1998,1.294

Belarus,BLR,1999,1.261

Belarus,BLR,2000,1.242

Belarus,BLR,2001,1.237

Belarus,BLR,2002,1.244

Belarus,BLR,2003,1.261

Belarus,BLR,2004,1.287

Belarus,BLR,2005,1.32

Belarus,BLR,2006,1.359

Belarus,BLR,2007,1.402

Belarus,BLR,2008,1.449

Belarus,BLR,2009,1.496

Belarus,BLR,2010,1.541

Belarus,BLR,2011,1.581

Belarus,BLR,2012,1.616

Belarus,BLR,2013,1.646

Belarus,BLR,2014,1.669

Belarus,BLR,2015,1.686

Belgium,BEL,1950,2.332

Belgium,BEL,1951,2.337

Belgium,BEL,1952,2.352

Belgium,BEL,1953,2.371

Belgium,BEL,1954,2.395

Belgium,BEL,1955,2.425

Belgium,BEL,1956,2.458

Belgium,BEL,1957,2.495

Belgium,BEL,1958,2.534

Belgium,BEL,1959,2.571

Belgium,BEL,1960,2.602

Belgium,BEL,1961,2.624

Belgium,BEL,1962,2.632

Belgium,BEL,1963,2.624

Belgium,BEL,1964,2.599

Belgium,BEL,1965,2.557

Belgium,BEL,1966,2.501

Belgium,BEL,1967,2.433

Belgium,BEL,1968,2.36

Belgium,BEL,1969,2.283

Belgium,BEL,1970,2.206

Belgium,BEL,1971,2.129

Belgium,BEL,1972,2.052

Belgium,BEL,1973,1.977

Belgium,BEL,1974,1.905

Belgium,BEL,1975,1.839

Belgium,BEL,1976,1.781

Belgium,BEL,1977,1.731

Belgium,BEL,1978,1.689

Belgium,BEL,1979,1.655

Belgium,BEL,1980,1.629

Belgium,BEL,1981,1.609

Belgium,BEL,1982,1.594

Belgium,BEL,1983,1.582

Belgium,BEL,1984,1.572

Belgium,BEL,1985,1.565

Belgium,BEL,1986,1.562

Belgium,BEL,1987,1.562

Belgium,BEL,1988,1.567

Belgium,BEL,1989,1.574

Belgium,BEL,1990,1.583

Belgium,BEL,1991,1.592

Belgium,BEL,1992,1.599

Belgium,BEL,1993,1.603

Belgium,BEL,1994,1.605

Belgium,BEL,1995,1.605

Belgium,BEL,1996,1.605

Belgium,BEL,1997,1.607

Belgium,BEL,1998,1.611

Belgium,BEL,1999,1.62

Belgium,BEL,2000,1.633

Belgium,BEL,2001,1.653

Belgium,BEL,2002,1.677

Belgium,BEL,2003,1.704

Belgium,BEL,2004,1.733

Belgium,BEL,2005,1.761

Belgium,BEL,2006,1.784

Belgium,BEL,2007,1.801

Belgium,BEL,2008,1.811

Belgium,BEL,2009,1.813

Belgium,BEL,2010,1.81

Belgium,BEL,2011,1.803

Belgium,BEL,2012,1.795

Belgium,BEL,2013,1.789

Belgium,BEL,2014,1.786

Belgium,BEL,2015,1.786

Belize,BLZ,1950,6.69

Belize,BLZ,1951,6.68

Belize,BLZ,1952,6.66

Belize,BLZ,1953,6.64

Belize,BLZ,1954,6.62

Belize,BLZ,1955,6.6

Belize,BLZ,1956,6.58

Belize,BLZ,1957,6.56

Belize,BLZ,1958,6.54

Belize,BLZ,1959,6.52

Belize,BLZ,1960,6.5

Belize,BLZ,1961,6.48

Belize,BLZ,1962,6.46

Belize,BLZ,1963,6.44

Belize,BLZ,1964,6.42

Belize,BLZ,1965,6.4

Belize,BLZ,1966,6.379

Belize,BLZ,1967,6.358

Belize,BLZ,1968,6.337

Belize,BLZ,1969,6.316

Belize,BLZ,1970,6.299

Belize,BLZ,1971,6.288

Belize,BLZ,1972,6.284

Belize,BLZ,1973,6.285

Belize,BLZ,1974,6.287

Belize,BLZ,1975,6.278

Belize,BLZ,1976,6.25

Belize,BLZ,1977,6.195

Belize,BLZ,1978,6.109

Belize,BLZ,1979,5.992

Belize,BLZ,1980,5.849

Belize,BLZ,1981,5.684

Belize,BLZ,1982,5.51

Belize,BLZ,1983,5.336

Belize,BLZ,1984,5.17

Belize,BLZ,1985,5.019

Belize,BLZ,1986,4.886

Belize,BLZ,1987,4.771

Belize,BLZ,1988,4.671

Belize,BLZ,1989,4.584

Belize,BLZ,1990,4.508

Belize,BLZ,1991,4.436

Belize,BLZ,1992,4.363

Belize,BLZ,1993,4.286

Belize,BLZ,1994,4.201

Belize,BLZ,1995,4.109

Belize,BLZ,1996,4.01

Belize,BLZ,1997,3.908

Belize,BLZ,1998,3.805

Belize,BLZ,1999,3.703

Belize,BLZ,2000,3.6

Belize,BLZ,2001,3.496

Belize,BLZ,2002,3.39

Belize,BLZ,2003,3.282

Belize,BLZ,2004,3.175

Belize,BLZ,2005,3.072

Belize,BLZ,2006,2.977

Belize,BLZ,2007,2.893

Belize,BLZ,2008,2.821

Belize,BLZ,2009,2.762

Belize,BLZ,2010,2.715

Belize,BLZ,2011,2.676

Belize,BLZ,2012,2.642

Belize,BLZ,2013,2.61

Belize,BLZ,2014,2.578

Belize,BLZ,2015,2.544

Benin,BEN,1950,5.751

Benin,BEN,1951,5.777

Benin,BEN,1952,5.829

Benin,BEN,1953,5.883

Benin,BEN,1954,5.938

Benin,BEN,1955,5.993

Benin,BEN,1956,6.05

Benin,BEN,1957,6.108

Benin,BEN,1958,6.166

Benin,BEN,1959,6.225

Benin,BEN,1960,6.282

Benin,BEN,1961,6.339

Benin,BEN,1962,6.394

Benin,BEN,1963,6.447

Benin,BEN,1964,6.497

Benin,BEN,1965,6.544

Benin,BEN,1966,6.588

Benin,BEN,1967,6.63

Benin,BEN,1968,6.671

Benin,BEN,1969,6.71

Benin,BEN,1970,6.748

Benin,BEN,1971,6.786

Benin,BEN,1972,6.823

Benin,BEN,1973,6.86

Benin,BEN,1974,6.896

Benin,BEN,1975,6.929

Benin,BEN,1976,6.959

Benin,BEN,1977,6.984

Benin,BEN,1978,7.004

Benin,BEN,1979,7.018

Benin,BEN,1980,7.025

Benin,BEN,1981,7.025

Benin,BEN,1982,7.021

Benin,BEN,1983,7.01

Benin,BEN,1984,6.994

Benin,BEN,1985,6.972

Benin,BEN,1986,6.942

Benin,BEN,1987,6.904

Benin,BEN,1988,6.858

Benin,BEN,1989,6.805

Benin,BEN,1990,6.744

Benin,BEN,1991,6.677

Benin,BEN,1992,6.603

Benin,BEN,1993,6.526

Benin,BEN,1994,6.446

Benin,BEN,1995,6.364

Benin,BEN,1996,6.282

Benin,BEN,1997,6.199

Benin,BEN,1998,6.118

Benin,BEN,1999,6.038

Benin,BEN,2000,5.962

Benin,BEN,2001,5.889

Benin,BEN,2002,5.82

Benin,BEN,2003,5.754

Benin,BEN,2004,5.691

Benin,BEN,2005,5.632

Benin,BEN,2006,5.576

Benin,BEN,2007,5.522

Benin,BEN,2008,5.469

Benin,BEN,2009,5.416

Benin,BEN,2010,5.362

Benin,BEN,2011,5.305

Benin,BEN,2012,5.246

Benin,BEN,2013,5.183

Benin,BEN,2014,5.117

Benin,BEN,2015,5.048

Bhutan,BTN,1950,6.67

Bhutan,BTN,1951,6.67

Bhutan,BTN,1952,6.67

Bhutan,BTN,1953,6.67

Bhutan,BTN,1954,6.67

Bhutan,BTN,1955,6.67

Bhutan,BTN,1956,6.67

Bhutan,BTN,1957,6.67

Bhutan,BTN,1958,6.67

Bhutan,BTN,1959,6.67

Bhutan,BTN,1960,6.67

Bhutan,BTN,1961,6.67

Bhutan,BTN,1962,6.67

Bhutan,BTN,1963,6.67

Bhutan,BTN,1964,6.67

Bhutan,BTN,1965,6.67

Bhutan,BTN,1966,6.67

Bhutan,BTN,1967,6.67

Bhutan,BTN,1968,6.67

Bhutan,BTN,1969,6.67

Bhutan,BTN,1970,6.671

Bhutan,BTN,1971,6.674

Bhutan,BTN,1972,6.679

Bhutan,BTN,1973,6.685

Bhutan,BTN,1974,6.69

Bhutan,BTN,1975,6.692

Bhutan,BTN,1976,6.685

Bhutan,BTN,1977,6.668

Bhutan,BTN,1978,6.64

Bhutan,BTN,1979,6.601

Bhutan,BTN,1980,6.553

Bhutan,BTN,1981,6.504

Bhutan,BTN,1982,6.456

Bhutan,BTN,1983,6.41

Bhutan,BTN,1984,6.362

Bhutan,BTN,1985,6.306

Bhutan,BTN,1986,6.23

Bhutan,BTN,1987,6.127

Bhutan,BTN,1988,5.994

Bhutan,BTN,1989,5.829

Bhutan,BTN,1990,5.639

Bhutan,BTN,1991,5.431

Bhutan,BTN,1992,5.216

Bhutan,BTN,1993,5.003

Bhutan,BTN,1994,4.796

Bhutan,BTN,1995,4.597

Bhutan,BTN,1996,4.4

Bhutan,BTN,1997,4.203

Bhutan,BTN,1998,4.002

Bhutan,BTN,1999,3.801

Bhutan,BTN,2000,3.604

Bhutan,BTN,2001,3.416

Bhutan,BTN,2002,3.244

Bhutan,BTN,2003,3.089

Bhutan,BTN,2004,2.954

Bhutan,BTN,2005,2.837

Bhutan,BTN,2006,2.734

Bhutan,BTN,2007,2.64

Bhutan,BTN,2008,2.55

Bhutan,BTN,2009,2.464

Bhutan,BTN,2010,2.382

Bhutan,BTN,2011,2.305

Bhutan,BTN,2012,2.236

Bhutan,BTN,2013,2.177

Bhutan,BTN,2014,2.128

Bhutan,BTN,2015,2.086

Bolivia,BOL,1950,6.928

Bolivia,BOL,1951,6.919

Bolivia,BOL,1952,6.901

Bolivia,BOL,1953,6.881

Bolivia,BOL,1954,6.86

Bolivia,BOL,1955,6.837

Bolivia,BOL,1956,6.813

Bolivia,BOL,1957,6.787

Bolivia,BOL,1958,6.76

Bolivia,BOL,1959,6.731

Bolivia,BOL,1960,6.7

Bolivia,BOL,1961,6.668

Bolivia,BOL,1962,6.633

Bolivia,BOL,1963,6.597

Bolivia,BOL,1964,6.559

Bolivia,BOL,1965,6.519

Bolivia,BOL,1966,6.477

Bolivia,BOL,1967,6.432

Bolivia,BOL,1968,6.384

Bolivia,BOL,1969,6.335

Bolivia,BOL,1970,6.284

Bolivia,BOL,1971,6.233

Bolivia,BOL,1972,6.182

Bolivia,BOL,1973,6.132

Bolivia,BOL,1974,6.081

Bolivia,BOL,1975,6.029

Bolivia,BOL,1976,5.975

Bolivia,BOL,1977,5.917

Bolivia,BOL,1978,5.855

Bolivia,BOL,1979,5.787

Bolivia,BOL,1980,5.715

Bolivia,BOL,1981,5.637

Bolivia,BOL,1982,5.556

Bolivia,BOL,1983,5.472

Bolivia,BOL,1984,5.386

Bolivia,BOL,1985,5.3

Bolivia,BOL,1986,5.215

Bolivia,BOL,1987,5.132

Bolivia,BOL,1988,5.051

Bolivia,BOL,1989,4.971

Bolivia,BOL,1990,4.894

Bolivia,BOL,1991,4.817

Bolivia,BOL,1992,4.741

Bolivia,BOL,1993,4.664

Bolivia,BOL,1994,4.585

Bolivia,BOL,1995,4.503

Bolivia,BOL,1996,4.418

Bolivia,BOL,1997,4.331

Bolivia,BOL,1998,4.241

Bolivia,BOL,1999,4.148

Bolivia,BOL,2000,4.055

Bolivia,BOL,2001,3.96

Bolivia,BOL,2002,3.866

Bolivia,BOL,2003,3.773

Bolivia,BOL,2004,3.683

Bolivia,BOL,2005,3.595

Bolivia,BOL,2006,3.51

Bolivia,BOL,2007,3.427

Bolivia,BOL,2008,3.348

Bolivia,BOL,2009,3.272

Bolivia,BOL,2010,3.2

Bolivia,BOL,2011,3.134

Bolivia,BOL,2012,3.072

Bolivia,BOL,2013,3.017

Bolivia,BOL,2014,2.966

Bolivia,BOL,2015,2.921

Bosnia and Herzegovina,BIH,1950,5.321

Bosnia and Herzegovina,BIH,1951,5.18

Bosnia and Herzegovina,BIH,1952,4.915

Bosnia and Herzegovina,BIH,1953,4.68

Bosnia and Herzegovina,BIH,1954,4.475

Bosnia and Herzegovina,BIH,1955,4.3

Bosnia and Herzegovina,BIH,1956,4.155

Bosnia and Herzegovina,BIH,1957,4.037

Bosnia and Herzegovina,BIH,1958,3.942

Bosnia and Herzegovina,BIH,1959,3.866

Bosnia and Herzegovina,BIH,1960,3.801

Bosnia and Herzegovina,BIH,1961,3.74

Bosnia and Herzegovina,BIH,1962,3.676

Bosnia and Herzegovina,BIH,1963,3.601

Bosnia and Herzegovina,BIH,1964,3.515

Bosnia and Herzegovina,BIH,1965,3.417

Bosnia and Herzegovina,BIH,1966,3.313

Bosnia and Herzegovina,BIH,1967,3.208

Bosnia and Herzegovina,BIH,1968,3.109

Bosnia and Herzegovina,BIH,1969,3.016

Bosnia and Herzegovina,BIH,1970,2.929

Bosnia and Herzegovina,BIH,1971,2.844

Bosnia and Herzegovina,BIH,1972,2.759

Bosnia and Herzegovina,BIH,1973,2.671

Bosnia and Herzegovina,BIH,1974,2.58

Bosnia and Herzegovina,BIH,1975,2.489

Bosnia and Herzegovina,BIH,1976,2.401

Bosnia and Herzegovina,BIH,1977,2.318

Bosnia and Herzegovina,BIH,1978,2.243

Bosnia and Herzegovina,BIH,1979,2.178

Bosnia and Herzegovina,BIH,1980,2.122

Bosnia and Herzegovina,BIH,1981,2.075

Bosnia and Herzegovina,BIH,1982,2.034

Bosnia and Herzegovina,BIH,1983,1.998

Bosnia and Herzegovina,BIH,1984,1.964

Bosnia and Herzegovina,BIH,1985,1.932

Bosnia and Herzegovina,BIH,1986,1.9

Bosnia and Herzegovina,BIH,1987,1.868

Bosnia and Herzegovina,BIH,1988,1.835

Bosnia and Herzegovina,BIH,1989,1.803

Bosnia and Herzegovina,BIH,1990,1.772

Bosnia and Herzegovina,BIH,1991,1.748

Bosnia and Herzegovina,BIH,1992,1.73

Bosnia and Herzegovina,BIH,1993,1.72

Bosnia and Herzegovina,BIH,1994,1.713

Bosnia and Herzegovina,BIH,1995,1.706

Bosnia and Herzegovina,BIH,1996,1.69

Bosnia and Herzegovina,BIH,1997,1.662

Bosnia and Herzegovina,BIH,1998,1.619

Bosnia and Herzegovina,BIH,1999,1.562

Bosnia and Herzegovina,BIH,2000,1.497

Bosnia and Herzegovina,BIH,2001,1.432

Bosnia and Herzegovina,BIH,2002,1.374

Bosnia and Herzegovina,BIH,2003,1.33

Bosnia and Herzegovina,BIH,2004,1.301

Bosnia and Herzegovina,BIH,2005,1.288

Bosnia and Herzegovina,BIH,2006,1.287

Bosnia and Herzegovina,BIH,2007,1.291

Bosnia and Herzegovina,BIH,2008,1.297

Bosnia and Herzegovina,BIH,2009,1.302

Bosnia and Herzegovina,BIH,2010,1.306

Bosnia and Herzegovina,BIH,2011,1.309

Bosnia and Herzegovina,BIH,2012,1.314

Bosnia and Herzegovina,BIH,2013,1.322

Bosnia and Herzegovina,BIH,2014,1.332

Bosnia and Herzegovina,BIH,2015,1.345

Botswana,BWA,1950,6.469

Botswana,BWA,1951,6.477

Botswana,BWA,1952,6.492

Botswana,BWA,1953,6.508

Botswana,BWA,1954,6.523

Botswana,BWA,1955,6.538

Botswana,BWA,1956,6.553

Botswana,BWA,1957,6.568

Botswana,BWA,1958,6.583

Botswana,BWA,1959,6.599

Botswana,BWA,1960,6.615

Botswana,BWA,1961,6.632

Botswana,BWA,1962,6.649

Botswana,BWA,1963,6.666

Botswana,BWA,1964,6.681

Botswana,BWA,1965,6.692

Botswana,BWA,1966,6.698

Botswana,BWA,1967,6.697

Botswana,BWA,1968,6.687

Botswana,BWA,1969,6.67

Botswana,BWA,1970,6.645

Botswana,BWA,1971,6.615

Botswana,BWA,1972,6.582

Botswana,BWA,1973,6.547

Botswana,BWA,1974,6.51

Botswana,BWA,1975,6.472

Botswana,BWA,1976,6.433

Botswana,BWA,1977,6.392

Botswana,BWA,1978,6.345

Botswana,BWA,1979,6.288

Botswana,BWA,1980,6.214

Botswana,BWA,1981,6.114

Botswana,BWA,1982,5.984

Botswana,BWA,1983,5.826

Botswana,BWA,1984,5.644

Botswana,BWA,1985,5.445

Botswana,BWA,1986,5.239

Botswana,BWA,1987,5.04

Botswana,BWA,1988,4.854

Botswana,BWA,1989,4.687

Botswana,BWA,1990,4.54

Botswana,BWA,1991,4.411

Botswana,BWA,1992,4.292

Botswana,BWA,1993,4.177

Botswana,BWA,1994,4.064

Botswana,BWA,1995,3.951

Botswana,BWA,1996,3.836

Botswana,BWA,1997,3.72

Botswana,BWA,1998,3.606

Botswana,BWA,1999,3.494

Botswana,BWA,2000,3.387

Botswana,BWA,2001,3.286

Botswana,BWA,2002,3.194

Botswana,BWA,2003,3.113

Botswana,BWA,2004,3.043

Botswana,BWA,2005,2.987

Botswana,BWA,2006,2.946

Botswana,BWA,2007,2.918

Botswana,BWA,2008,2.901

Botswana,BWA,2009,2.891

Botswana,BWA,2010,2.884

Botswana,BWA,2011,2.876

Botswana,BWA,2012,2.863

Botswana,BWA,2013,2.842

Botswana,BWA,2014,2.812

Botswana,BWA,2015,2.774

Brazil,BRA,1950,6.141

Brazil,BRA,1951,6.127

Brazil,BRA,1952,6.104

Brazil,BRA,1953,6.087

Brazil,BRA,1954,6.076

Brazil,BRA,1955,6.071

Brazil,BRA,1956,6.07

Brazil,BRA,1957,6.074

Brazil,BRA,1958,6.078

Brazil,BRA,1959,6.079

Brazil,BRA,1960,6.07

Brazil,BRA,1961,6.047

Brazil,BRA,1962,6.004

Brazil,BRA,1963,5.937

Brazil,BRA,1964,5.847

Brazil,BRA,1965,5.733

Brazil,BRA,1966,5.6

Brazil,BRA,1967,5.454

Brazil,BRA,1968,5.304

Brazil,BRA,1969,5.153

Brazil,BRA,1970,5.009

Brazil,BRA,1971,4.877

Brazil,BRA,1972,4.756

Brazil,BRA,1973,4.647

Brazil,BRA,1974,4.55

Brazil,BRA,1975,4.463

Brazil,BRA,1976,4.385

Brazil,BRA,1977,4.311

Brazil,BRA,1978,4.235

Brazil,BRA,1979,4.156

Brazil,BRA,1980,4.068

Brazil,BRA,1981,3.97

Brazil,BRA,1982,3.861

Brazil,BRA,1983,3.743

Brazil,BRA,1984,3.618

Brazil,BRA,1985,3.488

Brazil,BRA,1986,3.358

Brazil,BRA,1987,3.232

Brazil,BRA,1988,3.113

Brazil,BRA,1989,3.005

Brazil,BRA,1990,2.909

Brazil,BRA,1991,2.827

Brazil,BRA,1992,2.756

Brazil,BRA,1993,2.694

Brazil,BRA,1994,2.638

Brazil,BRA,1995,2.586

Brazil,BRA,1996,2.535

Brazil,BRA,1997,2.482

Brazil,BRA,1998,2.426

Brazil,BRA,1999,2.365

Brazil,BRA,2000,2.3

Brazil,BRA,2001,2.232

Brazil,BRA,2002,2.163

Brazil,BRA,2003,2.096

Brazil,BRA,2004,2.033

Brazil,BRA,2005,1.975

Brazil,BRA,2006,1.926

Brazil,BRA,2007,1.884

Brazil,BRA,2008,1.851

Brazil,BRA,2009,1.824

Brazil,BRA,2010,1.805

Brazil,BRA,2011,1.79

Brazil,BRA,2012,1.777

Brazil,BRA,2013,1.765

Brazil,BRA,2014,1.753

Brazil,BRA,2015,1.74

Brunei,BRN,1950,6.862

Brunei,BRN,1951,6.872

Brunei,BRN,1952,6.891

Brunei,BRN,1953,6.906

Brunei,BRN,1954,6.917

Brunei,BRN,1955,6.924

Brunei,BRN,1956,6.925

Brunei,BRN,1957,6.92

Brunei,BRN,1958,6.906

Brunei,BRN,1959,6.879

Brunei,BRN,1960,6.836

Brunei,BRN,1961,6.771

Brunei,BRN,1962,6.683

Brunei,BRN,1963,6.572

Brunei,BRN,1964,6.44

Brunei,BRN,1965,6.297

Brunei,BRN,1966,6.156

Brunei,BRN,1967,6.026

Brunei,BRN,1968,5.912

Brunei,BRN,1969,5.812

Brunei,BRN,1970,5.719

Brunei,BRN,1971,5.619

Brunei,BRN,1972,5.5

Brunei,BRN,1973,5.355

Brunei,BRN,1974,5.183

Brunei,BRN,1975,4.99

Brunei,BRN,1976,4.784

Brunei,BRN,1977,4.58

Brunei,BRN,1978,4.389

Brunei,BRN,1979,4.216

Brunei,BRN,1980,4.067

Brunei,BRN,1981,3.943

Brunei,BRN,1982,3.84

Brunei,BRN,1983,3.751

Brunei,BRN,1984,3.675

Brunei,BRN,1985,3.608

Brunei,BRN,1986,3.546

Brunei,BRN,1987,3.488

Brunei,BRN,1988,3.428

Brunei,BRN,1989,3.363

Brunei,BRN,1990,3.291

Brunei,BRN,1991,3.209

Brunei,BRN,1992,3.118

Brunei,BRN,1993,3.02

Brunei,BRN,1994,2.914

Brunei,BRN,1995,2.802

Brunei,BRN,1996,2.685

Brunei,BRN,1997,2.564

Brunei,BRN,1998,2.444

Brunei,BRN,1999,2.328

Brunei,BRN,2000,2.218

Brunei,BRN,2001,2.119

Brunei,BRN,2002,2.03

Brunei,BRN,2003,1.954

Brunei,BRN,2004,1.893

Brunei,BRN,2005,1.847

Brunei,BRN,2006,1.819

Brunei,BRN,2007,1.808

Brunei,BRN,2008,1.809

Brunei,BRN,2009,1.821

Brunei,BRN,2010,1.838

Brunei,BRN,2011,1.857

Brunei,BRN,2012,1.874

Brunei,BRN,2013,1.884

Brunei,BRN,2014,1.888

Brunei,BRN,2015,1.884

Bulgaria,BGR,1950,2.66

Bulgaria,BGR,1951,2.622

Bulgaria,BGR,1952,2.552

Bulgaria,BGR,1953,2.489

Bulgaria,BGR,1954,2.434

Bulgaria,BGR,1955,2.387

Bulgaria,BGR,1956,2.346

Bulgaria,BGR,1957,2.313

Bulgaria,BGR,1958,2.286

Bulgaria,BGR,1959,2.264

Bulgaria,BGR,1960,2.245

Bulgaria,BGR,1961,2.229

Bulgaria,BGR,1962,2.213

Bulgaria,BGR,1963,2.198

Bulgaria,BGR,1964,2.181

Bulgaria,BGR,1965,2.165

Bulgaria,BGR,1966,2.151

Bulgaria,BGR,1967,2.14

Bulgaria,BGR,1968,2.133

Bulgaria,BGR,1969,2.132

Bulgaria,BGR,1970,2.135

Bulgaria,BGR,1971,2.144

Bulgaria,BGR,1972,2.155

Bulgaria,BGR,1973,2.169

Bulgaria,BGR,1974,2.181

Bulgaria,BGR,1975,2.19

Bulgaria,BGR,1976,2.191

Bulgaria,BGR,1977,2.184

Bulgaria,BGR,1978,2.168

Bulgaria,BGR,1979,2.143

Bulgaria,BGR,1980,2.113

Bulgaria,BGR,1981,2.082

Bulgaria,BGR,1982,2.055

Bulgaria,BGR,1983,2.033

Bulgaria,BGR,1984,2.015

Bulgaria,BGR,1985,1.997

Bulgaria,BGR,1986,1.976

Bulgaria,BGR,1987,1.944

Bulgaria,BGR,1988,1.899

Bulgaria,BGR,1989,1.841

Bulgaria,BGR,1990,1.769

Bulgaria,BGR,1991,1.686

Bulgaria,BGR,1992,1.597

Bulgaria,BGR,1993,1.507

Bulgaria,BGR,1994,1.421

Bulgaria,BGR,1995,1.344

Bulgaria,BGR,1996,1.28

Bulgaria,BGR,1997,1.23

Bulgaria,BGR,1998,1.196

Bulgaria,BGR,1999,1.179

Bulgaria,BGR,2000,1.178

Bulgaria,BGR,2001,1.193

Bulgaria,BGR,2002,1.221

Bulgaria,BGR,2003,1.258

Bulgaria,BGR,2004,1.3

Bulgaria,BGR,2005,1.343

Bulgaria,BGR,2006,1.383

Bulgaria,BGR,2007,1.418

Bulgaria,BGR,2008,1.448

Bulgaria,BGR,2009,1.471

Bulgaria,BGR,2010,1.488

Bulgaria,BGR,2011,1.499

Bulgaria,BGR,2012,1.509

Bulgaria,BGR,2013,1.52

Bulgaria,BGR,2014,1.532

Bulgaria,BGR,2015,1.545

Burkina Faso,BFA,1950,6.034

Burkina Faso,BFA,1951,6.053

Burkina Faso,BFA,1952,6.089

Burkina Faso,BFA,1953,6.122

Burkina Faso,BFA,1954,6.152

Burkina Faso,BFA,1955,6.18

Burkina Faso,BFA,1956,6.205

Burkina Faso,BFA,1957,6.227

Burkina Faso,BFA,1958,6.248

Burkina Faso,BFA,1959,6.269

Burkina Faso,BFA,1960,6.291

Burkina Faso,BFA,1961,6.316

Burkina Faso,BFA,1962,6.344

Burkina Faso,BFA,1963,6.377

Burkina Faso,BFA,1964,6.414

Burkina Faso,BFA,1965,6.454

Burkina Faso,BFA,1966,6.493

Burkina Faso,BFA,1967,6.53

Burkina Faso,BFA,1968,6.563

Burkina Faso,BFA,1969,6.593

Burkina Faso,BFA,1970,6.623

Burkina Faso,BFA,1971,6.657

Burkina Faso,BFA,1972,6.697

Burkina Faso,BFA,1973,6.744

Burkina Faso,BFA,1974,6.799

Burkina Faso,BFA,1975,6.859

Burkina Faso,BFA,1976,6.922

Burkina Faso,BFA,1977,6.984

Burkina Faso,BFA,1978,7.041

Burkina Faso,BFA,1979,7.09

Burkina Faso,BFA,1980,7.127

Burkina Faso,BFA,1981,7.152

Burkina Faso,BFA,1982,7.165

Burkina Faso,BFA,1983,7.167

Burkina Faso,BFA,1984,7.159

Burkina Faso,BFA,1985,7.142

Burkina Faso,BFA,1986,7.119

Burkina Faso,BFA,1987,7.092

Burkina Faso,BFA,1988,7.064

Burkina Faso,BFA,1989,7.036

Burkina Faso,BFA,1990,7.007

Burkina Faso,BFA,1991,6.979

Burkina Faso,BFA,1992,6.95

Burkina Faso,BFA,1993,6.917

Burkina Faso,BFA,1994,6.882

Burkina Faso,BFA,1995,6.844

Burkina Faso,BFA,1996,6.801

Burkina Faso,BFA,1997,6.754

Burkina Faso,BFA,1998,6.704

Burkina Faso,BFA,1999,6.65

Burkina Faso,BFA,2000,6.592

Burkina Faso,BFA,2001,6.531

Burkina Faso,BFA,2002,6.468

Burkina Faso,BFA,2003,6.403

Burkina Faso,BFA,2004,6.335

Burkina Faso,BFA,2005,6.265

Burkina Faso,BFA,2006,6.192

Burkina Faso,BFA,2007,6.116

Burkina Faso,BFA,2008,6.036

Burkina Faso,BFA,2009,5.953

Burkina Faso,BFA,2010,5.868

Burkina Faso,BFA,2011,5.781

Burkina Faso,BFA,2012,5.693

Burkina Faso,BFA,2013,5.607

Burkina Faso,BFA,2014,5.521

Burkina Faso,BFA,2015,5.436

Burundi,BDI,1950,6.819

Burundi,BDI,1951,6.811

Burundi,BDI,1952,6.798

Burundi,BDI,1953,6.793

Burundi,BDI,1954,6.795

Burundi,BDI,1955,6.804

Burundi,BDI,1956,6.821

Burundi,BDI,1957,6.845

Burundi,BDI,1958,6.875

Burundi,BDI,1959,6.912

Burundi,BDI,1960,6.953

Burundi,BDI,1961,6.999

Burundi,BDI,1962,7.046

Burundi,BDI,1963,7.094

Burundi,BDI,1964,7.141

Burundi,BDI,1965,7.183

Burundi,BDI,1966,7.218

Burundi,BDI,1967,7.245

Burundi,BDI,1968,7.265

Burundi,BDI,1969,7.279

Burundi,BDI,1970,7.289

Burundi,BDI,1971,7.299

Burundi,BDI,1972,7.312

Burundi,BDI,1973,7.329

Burundi,BDI,1974,7.35

Burundi,BDI,1975,7.373

Burundi,BDI,1976,7.394

Burundi,BDI,1977,7.41

Burundi,BDI,1978,7.418

Burundi,BDI,1979,7.42

Burundi,BDI,1980,7.418

Burundi,BDI,1981,7.417

Burundi,BDI,1982,7.423

Burundi,BDI,1983,7.438

Burundi,BDI,1984,7.459

Burundi,BDI,1985,7.485

Burundi,BDI,1986,7.509

Burundi,BDI,1987,7.527

Burundi,BDI,1988,7.533

Burundi,BDI,1989,7.526

Burundi,BDI,1990,7.505

Burundi,BDI,1991,7.473

Burundi,BDI,1992,7.432

Burundi,BDI,1993,7.387

Burundi,BDI,1994,7.338

Burundi,BDI,1995,7.288

Burundi,BDI,1996,7.236

Burundi,BDI,1997,7.182

Burundi,BDI,1998,7.126

Burundi,BDI,1999,7.068

Burundi,BDI,2000,7.008

Burundi,BDI,2001,6.948

Burundi,BDI,2002,6.887

Burundi,BDI,2003,6.824

Burundi,BDI,2004,6.759

Burundi,BDI,2005,6.69

Burundi,BDI,2006,6.616

Burundi,BDI,2007,6.534

Burundi,BDI,2008,6.447

Burundi,BDI,2009,6.353

Burundi,BDI,2010,6.256

Burundi,BDI,2011,6.156

Burundi,BDI,2012,6.058

Burundi,BDI,2013,5.962

Burundi,BDI,2014,5.869

Burundi,BDI,2015,5.781

Cambodia,KHM,1950,6.953

Cambodia,KHM,1951,6.95

Cambodia,KHM,1952,6.946

Cambodia,KHM,1953,6.945

Cambodia,KHM,1954,6.945

Cambodia,KHM,1955,6.947

Cambodia,KHM,1956,6.95

Cambodia,KHM,1957,6.955

Cambodia,KHM,1958,6.96

Cambodia,KHM,1959,6.965

Cambodia,KHM,1960,6.967

Cambodia,KHM,1961,6.964

Cambodia,KHM,1962,6.955

Cambodia,KHM,1963,6.937

Cambodia,KHM,1964,6.909

Cambodia,KHM,1965,6.868

Cambodia,KHM,1966,6.813

Cambodia,KHM,1967,6.745

Cambodia,KHM,1968,6.666

Cambodia,KHM,1969,6.574

Cambodia,KHM,1970,6.465

Cambodia,KHM,1971,6.332

Cambodia,KHM,1972,6.175

Cambodia,KHM,1973,6.004

Cambodia,KHM,1974,5.831

Cambodia,KHM,1975,5.681

Cambodia,KHM,1976,5.585

Cambodia,KHM,1977,5.557

Cambodia,KHM,1978,5.603

Cambodia,KHM,1979,5.714

Cambodia,KHM,1980,5.87

Cambodia,KHM,1981,6.042

Cambodia,KHM,1982,6.193

Cambodia,KHM,1983,6.296

Cambodia,KHM,1984,6.338

Cambodia,KHM,1985,6.313

Cambodia,KHM,1986,6.226

Cambodia,KHM,1987,6.097

Cambodia,KHM,1988,5.944

Cambodia,KHM,1989,5.777

Cambodia,KHM,1990,5.601

Cambodia,KHM,1991,5.421

Cambodia,KHM,1992,5.237

Cambodia,KHM,1993,5.052

Cambodia,KHM,1994,4.867

Cambodia,KHM,1995,4.685

Cambodia,KHM,1996,4.504

Cambodia,KHM,1997,4.325

Cambodia,KHM,1998,4.146

Cambodia,KHM,1999,3.971

Cambodia,KHM,2000,3.805

Cambodia,KHM,2001,3.654

Cambodia,KHM,2002,3.521

Cambodia,KHM,2003,3.406

Cambodia,KHM,2004,3.309

Cambodia,KHM,2005,3.227

Cambodia,KHM,2006,3.155

Cambodia,KHM,2007,3.086

Cambodia,KHM,2008,3.018

Cambodia,KHM,2009,2.947

Cambodia,KHM,2010,2.875

Cambodia,KHM,2011,2.805

Cambodia,KHM,2012,2.739

Cambodia,KHM,2013,2.682

Cambodia,KHM,2014,2.634

Cambodia,KHM,2015,2.594

Cameroon,CMR,1950,5.537

Cameroon,CMR,1951,5.519

Cameroon,CMR,1952,5.49

Cameroon,CMR,1953,5.471

Cameroon,CMR,1954,5.465

Cameroon,CMR,1955,5.469

Cameroon,CMR,1956,5.485

Cameroon,CMR,1957,5.512

Cameroon,CMR,1958,5.549

Cameroon,CMR,1959,5.594

Cameroon,CMR,1960,5.647

Cameroon,CMR,1961,5.705

Cameroon,CMR,1962,5.766

Cameroon,CMR,1963,5.828

Cameroon,CMR,1964,5.89

Cameroon,CMR,1965,5.949

Cameroon,CMR,1966,6.004

Cameroon,CMR,1967,6.058

Cameroon,CMR,1968,6.108

Cameroon,CMR,1969,6.157

Cameroon,CMR,1970,6.203

Cameroon,CMR,1971,6.246

Cameroon,CMR,1972,6.287

Cameroon,CMR,1973,6.328

Cameroon,CMR,1974,6.367

Cameroon,CMR,1975,6.406

Cameroon,CMR,1976,6.448

Cameroon,CMR,1977,6.492

Cameroon,CMR,1978,6.538

Cameroon,CMR,1979,6.583

Cameroon,CMR,1980,6.625

Cameroon,CMR,1981,6.661

Cameroon,CMR,1982,6.688

Cameroon,CMR,1983,6.704

Cameroon,CMR,1984,6.707

Cameroon,CMR,1985,6.695

Cameroon,CMR,1986,6.667

Cameroon,CMR,1987,6.626

Cameroon,CMR,1988,6.573

Cameroon,CMR,1989,6.509

Cameroon,CMR,1990,6.436

Cameroon,CMR,1991,6.354

Cameroon,CMR,1992,6.264

Cameroon,CMR,1993,6.17

Cameroon,CMR,1994,6.073

Cameroon,CMR,1995,5.978

Cameroon,CMR,1996,5.886

Cameroon,CMR,1997,5.799

Cameroon,CMR,1998,5.718

Cameroon,CMR,1999,5.645

Cameroon,CMR,2000,5.58

Cameroon,CMR,2001,5.524

Cameroon,CMR,2002,5.475

Cameroon,CMR,2003,5.431

Cameroon,CMR,2004,5.39

Cameroon,CMR,2005,5.35

Cameroon,CMR,2006,5.309

Cameroon,CMR,2007,5.266

Cameroon,CMR,2008,5.219

Cameroon,CMR,2009,5.167

Cameroon,CMR,2010,5.111

Cameroon,CMR,2011,5.049

Cameroon,CMR,2012,4.984

Cameroon,CMR,2013,4.917

Cameroon,CMR,2014,4.848

Cameroon,CMR,2015,4.778

Canada,CAN,1950,3.469

Canada,CAN,1951,3.518

Canada,CAN,1952,3.612

Canada,CAN,1953,3.695

Canada,CAN,1954,3.766

Canada,CAN,1955,3.827

Canada,CAN,1956,3.874

Canada,CAN,1957,3.907

Canada,CAN,1958,3.923

Canada,CAN,1959,3.916

Canada,CAN,1960,3.88

Canada,CAN,1961,3.809

Canada,CAN,1962,3.701

Canada,CAN,1963,3.555

Canada,CAN,1964,3.377

Canada,CAN,1965,3.175

Canada,CAN,1966,2.963

Canada,CAN,1967,2.752

Canada,CAN,1968,2.555

Canada,CAN,1969,2.38

Canada,CAN,1970,2.232

Canada,CAN,1971,2.112

Canada,CAN,1972,2.014

Canada,CAN,1973,1.933

Canada,CAN,1974,1.866

Canada,CAN,1975,1.812

Canada,CAN,1976,1.769

Canada,CAN,1977,1.735

Canada,CAN,1978,1.708

Canada,CAN,1979,1.685

Canada,CAN,1980,1.665

Canada,CAN,1981,1.648

Canada,CAN,1982,1.634

Canada,CAN,1983,1.623

Canada,CAN,1984,1.615

Canada,CAN,1985,1.612

Canada,CAN,1986,1.613

Canada,CAN,1987,1.621

Canada,CAN,1988,1.633

Canada,CAN,1989,1.648

Canada,CAN,1990,1.663

Canada,CAN,1991,1.674

Canada,CAN,1992,1.677

Canada,CAN,1993,1.672

Canada,CAN,1994,1.658

Canada,CAN,1995,1.637

Canada,CAN,1996,1.61

Canada,CAN,1997,1.582

Canada,CAN,1998,1.556

Canada,CAN,1999,1.535

Canada,CAN,2000,1.521

Canada,CAN,2001,1.517

Canada,CAN,2002,1.522

Canada,CAN,2003,1.534

Canada,CAN,2004,1.552

Canada,CAN,2005,1.574

Canada,CAN,2006,1.596

Canada,CAN,2007,1.615

Canada,CAN,2008,1.628

Canada,CAN,2009,1.635

Canada,CAN,2010,1.635

Canada,CAN,2011,1.629

Canada,CAN,2012,1.619

Canada,CAN,2013,1.608

Canada,CAN,2014,1.596

Canada,CAN,2015,1.586

Cape Verde,CPV,1950,6.497

Cape Verde,CPV,1951,6.513

Cape Verde,CPV,1952,6.546

Cape Verde,CPV,1953,6.582

Cape Verde,CPV,1954,6.621

Cape Verde,CPV,1955,6.662

Cape Verde,CPV,1956,6.706

Cape Verde,CPV,1957,6.751

Cape Verde,CPV,1958,6.797

Cape Verde,CPV,1959,6.842

Cape Verde,CPV,1960,6.885

Cape Verde,CPV,1961,6.922

Cape Verde,CPV,1962,6.953

Cape Verde,CPV,1963,6.976

Cape Verde,CPV,1964,6.989

Cape Verde,CPV,1965,6.993

Cape Verde,CPV,1966,6.99

Cape Verde,CPV,1967,6.982

Cape Verde,CPV,1968,6.97

Cape Verde,CPV,1969,6.954

Cape Verde,CPV,1970,6.935

Cape Verde,CPV,1971,6.913

Cape Verde,CPV,1972,6.887

Cape Verde,CPV,1973,6.855

Cape Verde,CPV,1974,6.818

Cape Verde,CPV,1975,6.771

Cape Verde,CPV,1976,6.714

Cape Verde,CPV,1977,6.645

Cape Verde,CPV,1978,6.564

Cape Verde,CPV,1979,6.473

Cape Verde,CPV,1980,6.375

Cape Verde,CPV,1981,6.274

Cape Verde,CPV,1982,6.174

Cape Verde,CPV,1983,6.075

Cape Verde,CPV,1984,5.979

Cape Verde,CPV,1985,5.883

Cape Verde,CPV,1986,5.784

Cape Verde,CPV,1987,5.68

Cape Verde,CPV,1988,5.565

Cape Verde,CPV,1989,5.441

Cape Verde,CPV,1990,5.307

Cape Verde,CPV,1991,5.162

Cape Verde,CPV,1992,5.011

Cape Verde,CPV,1993,4.856

Cape Verde,CPV,1994,4.698

Cape Verde,CPV,1995,4.539

Cape Verde,CPV,1996,4.38

Cape Verde,CPV,1997,4.222

Cape Verde,CPV,1998,4.065

Cape Verde,CPV,1999,3.911

Cape Verde,CPV,2000,3.762

Cape Verde,CPV,2001,3.62

Cape Verde,CPV,2002,3.484

Cape Verde,CPV,2003,3.357

Cape Verde,CPV,2004,3.237

Cape Verde,CPV,2005,3.126

Cape Verde,CPV,2006,3.022

Cape Verde,CPV,2007,2.925

Cape Verde,CPV,2008,2.833

Cape Verde,CPV,2009,2.747

Cape Verde,CPV,2010,2.666

Cape Verde,CPV,2011,2.593

Cape Verde,CPV,2012,2.528

Cape Verde,CPV,2013,2.47

Cape Verde,CPV,2014,2.419

Cape Verde,CPV,2015,2.374

Caribbean,,1950,5.417

Caribbean,,1951,5.367

Caribbean,,1952,5.278

Caribbean,,1953,5.215

Caribbean,,1954,5.178

Caribbean,,1955,5.166

Caribbean,,1956,5.177

Caribbean,,1957,5.209

Caribbean,,1958,5.258

Caribbean,,1959,5.314

Caribbean,,1960,5.37

Caribbean,,1961,5.414

Caribbean,,1962,5.436

Caribbean,,1963,5.428

Caribbean,,1964,5.387

Caribbean,,1965,5.314

Caribbean,,1966,5.214

Caribbean,,1967,5.097

Caribbean,,1968,4.971

Caribbean,,1969,4.841

Caribbean,,1970,4.708

Caribbean,,1971,4.569

Caribbean,,1972,4.422

Caribbean,,1973,4.269

Caribbean,,1974,4.112

Caribbean,,1975,3.961

Caribbean,,1976,3.823

Caribbean,,1977,3.704

Caribbean,,1978,3.607

Caribbean,,1979,3.533

Caribbean,,1980,3.478

Caribbean,,1981,3.437

Caribbean,,1982,3.401

Caribbean,,1983,3.364

Caribbean,,1984,3.322

Caribbean,,1985,3.273

Caribbean,,1986,3.217

Caribbean,,1987,3.157

Caribbean,,1988,3.095

Caribbean,,1989,3.034

Caribbean,,1990,2.975

Caribbean,,1991,2.917

Caribbean,,1992,2.864

Caribbean,,1993,2.814

Caribbean,,1994,2.768

Caribbean,,1995,2.726

Caribbean,,1996,2.687

Caribbean,,1997,2.652

Caribbean,,1998,2.618

Caribbean,,1999,2.586

Caribbean,,2000,2.556

Caribbean,,2001,2.527

Caribbean,,2002,2.499

Caribbean,,2003,2.474

Caribbean,,2004,2.451

Caribbean,,2005,2.429

Caribbean,,2006,2.408

Caribbean,,2007,2.388

Caribbean,,2008,2.368

Caribbean,,2009,2.349

Caribbean,,2010,2.331

Caribbean,,2011,2.312

Caribbean,,2012,2.293

Caribbean,,2013,2.275

Caribbean,,2014,2.257

Caribbean,,2015,2.239

Central African Republic,CAF,1950,5.403

Central African Republic,CAF,1951,5.433

Central African Republic,CAF,1952,5.491

Central African Republic,CAF,1953,5.546

Central African Republic,CAF,1954,5.597

Central African Republic,CAF,1955,5.646

Central African Republic,CAF,1956,5.692

Central African Republic,CAF,1957,5.734

Central African Republic,CAF,1958,5.773

Central African Republic,CAF,1959,5.808

Central African Republic,CAF,1960,5.84

Central African Republic,CAF,1961,5.867

Central African Republic,CAF,1962,5.891

Central African Republic,CAF,1963,5.91

Central African Republic,CAF,1964,5.925

Central African Republic,CAF,1965,5.937

Central African Republic,CAF,1966,5.945

Central African Republic,CAF,1967,5.95

Central African Republic,CAF,1968,5.953

Central African Republic,CAF,1969,5.954

Central African Republic,CAF,1970,5.954

Central African Republic,CAF,1971,5.954

Central African Republic,CAF,1972,5.953

Central African Republic,CAF,1973,5.952

Central African Republic,CAF,1974,5.951

Central African Republic,CAF,1975,5.95

Central African Republic,CAF,1976,5.951

Central African Republic,CAF,1977,5.952

Central African Republic,CAF,1978,5.953

Central African Republic,CAF,1979,5.954

Central African Republic,CAF,1980,5.954

Central African Republic,CAF,1981,5.955

Central African Republic,CAF,1982,5.955

Central African Republic,CAF,1983,5.953

Central African Republic,CAF,1984,5.949

Central African Republic,CAF,1985,5.94

Central African Republic,CAF,1986,5.926

Central African Republic,CAF,1987,5.905

Central African Republic,CAF,1988,5.878

Central African Republic,CAF,1989,5.845

Central African Republic,CAF,1990,5.808

Central African Republic,CAF,1991,5.767

Central African Republic,CAF,1992,5.727

Central African Republic,CAF,1993,5.687

Central African Republic,CAF,1994,5.651

Central African Republic,CAF,1995,5.618

Central African Republic,CAF,1996,5.589

Central African Republic,CAF,1997,5.564

Central African Republic,CAF,1998,5.541

Central African Republic,CAF,1999,5.52

Central African Republic,CAF,2000,5.5

Central African Republic,CAF,2001,5.48

Central African Republic,CAF,2002,5.459

Central African Republic,CAF,2003,5.436

Central African Republic,CAF,2004,5.411

Central African Republic,CAF,2005,5.383

Central African Republic,CAF,2006,5.353

Central African Republic,CAF,2007,5.322

Central African Republic,CAF,2008,5.289

Central African Republic,CAF,2009,5.254

Central African Republic,CAF,2010,5.215

Central African Republic,CAF,2011,5.172

Central African Republic,CAF,2012,5.123

Central African Republic,CAF,2013,5.067

Central African Republic,CAF,2014,5.006

Central African Republic,CAF,2015,4.94

Central America,,1950,6.714

Central America,,1951,6.729

Central America,,1952,6.756

Central America,,1953,6.778

Central America,,1954,6.794

Central America,,1955,6.805

Central America,,1956,6.811

Central America,,1957,6.812

Central America,,1958,6.809

Central America,,1959,6.802

Central America,,1960,6.792

Central America,,1961,6.78

Central America,,1962,6.767

Central America,,1963,6.753

Central America,,1964,6.739

Central America,,1965,6.727

Central America,,1966,6.722

Central America,,1967,6.721

Central America,,1968,6.721

Central America,,1969,6.715

Central America,,1970,6.694

Central America,,1971,6.644

Central America,,1972,6.558

Central America,,1973,6.434

Central America,,1974,6.272

Central America,,1975,6.078

Central America,,1976,5.862

Central America,,1977,5.637

Central America,,1978,5.415

Central America,,1979,5.205

Central America,,1980,5.011

Central America,,1981,4.837

Central America,,1982,4.677

Central America,,1983,4.528

Central America,,1984,4.39

Central America,,1985,4.263

Central America,,1986,4.145

Central America,,1987,4.033

Central America,,1988,3.927

Central America,,1989,3.825

Central America,,1990,3.726

Central America,,1991,3.63

Central America,,1992,3.537

Central America,,1993,3.447

Central America,,1994,3.361

Central America,,1995,3.279

Central America,,1996,3.202

Central America,,1997,3.129

Central America,,1998,3.061

Central America,,1999,2.996

Central America,,2000,2.936

Central America,,2001,2.877

Central America,,2002,2.821

Central America,,2003,2.765

Central America,,2004,2.711

Central America,,2005,2.658

Central America,,2006,2.608

Central America,,2007,2.562

Central America,,2008,2.52

Central America,,2009,2.482

Central America,,2010,2.448

Central America,,2011,2.416

Central America,,2012,2.386

Central America,,2013,2.355

Central America,,2014,2.325

Central America,,2015,2.293

Central Asia,,1950,4.642

Central Asia,,1951,4.697

Central Asia,,1952,4.806

Central Asia,,1953,4.913

Central Asia,,1954,5.017

Central Asia,,1955,5.118

Central Asia,,1956,5.215

Central Asia,,1957,5.307

Central Asia,,1958,5.39

Central Asia,,1959,5.461

Central Asia,,1960,5.514

Central Asia,,1961,5.543

Central Asia,,1962,5.545

Central Asia,,1963,5.519

Central Asia,,1964,5.467

Central Asia,,1965,5.398

Central Asia,,1966,5.323

Central Asia,,1967,5.252

Central Asia,,1968,5.191

Central Asia,,1969,5.142

Central Asia,,1970,5.103

Central Asia,,1971,5.064

Central Asia,,1972,5.017

Central Asia,,1973,4.954

Central Asia,,1974,4.876

Central Asia,,1975,4.783

Central Asia,,1976,4.68

Central Asia,,1977,4.574

Central Asia,,1978,4.472

Central Asia,,1979,4.377

Central Asia,,1980,4.295

Central Asia,,1981,4.228

Central Asia,,1982,4.174

Central Asia,,1983,4.132

Central Asia,,1984,4.098

Central Asia,,1985,4.069

Central Asia,,1986,4.039

Central Asia,,1987,4.003

Central Asia,,1988,3.958

Central Asia,,1989,3.899

Central Asia,,1990,3.824

Central Asia,,1991,3.729

Central Asia,,1992,3.618

Central Asia,,1993,3.495

Central Asia,,1994,3.363

Central Asia,,1995,3.226

Central Asia,,1996,3.088

Central Asia,,1997,2.956

Central Asia,,1998,2.833

Central Asia,,1999,2.726

Central Asia,,2000,2.639

Central Asia,,2001,2.578

Central Asia,,2002,2.541

Central Asia,,2003,2.527

Central Asia,,2004,2.533

Central Asia,,2005,2.555

Central Asia,,2006,2.587

Central Asia,,2007,2.623

Central Asia,,2008,2.658

Central Asia,,2009,2.686

Central Asia,,2010,2.704

Central Asia,,2011,2.71

Central Asia,,2012,2.705

Central Asia,,2013,2.692

Central Asia,,2014,2.672

Central Asia,,2015,2.645

Chad,TCD,1950,6.062

Chad,TCD,1951,6.071

Chad,TCD,1952,6.09

Chad,TCD,1953,6.109

Chad,TCD,1954,6.129

Chad,TCD,1955,6.149

Chad,TCD,1956,6.169

Chad,TCD,1957,6.19

Chad,TCD,1958,6.21

Chad,TCD,1959,6.23

Chad,TCD,1960,6.25

Chad,TCD,1961,6.268

Chad,TCD,1962,6.285

Chad,TCD,1963,6.301

Chad,TCD,1964,6.318

Chad,TCD,1965,6.337

Chad,TCD,1966,6.362

Chad,TCD,1967,6.393

Chad,TCD,1968,6.432

Chad,TCD,1969,6.477

Chad,TCD,1970,6.528

Chad,TCD,1971,6.582

Chad,TCD,1972,6.636

Chad,TCD,1973,6.688

Chad,TCD,1974,6.736

Chad,TCD,1975,6.78

Chad,TCD,1976,6.82

Chad,TCD,1977,6.857

Chad,TCD,1978,6.892

Chad,TCD,1979,6.925

Chad,TCD,1980,6.958

Chad,TCD,1981,6.99

Chad,TCD,1982,7.023

Chad,TCD,1983,7.056

Chad,TCD,1984,7.089

Chad,TCD,1985,7.124

Chad,TCD,1986,7.161

Chad,TCD,1987,7.199

Chad,TCD,1988,7.238

Chad,TCD,1989,7.276

Chad,TCD,1990,7.313

Chad,TCD,1991,7.346

Chad,TCD,1992,7.376

Chad,TCD,1993,7.399

Chad,TCD,1994,7.416

Chad,TCD,1995,7.425

Chad,TCD,1996,7.426

Chad,TCD,1997,7.419

Chad,TCD,1998,7.405

Chad,TCD,1999,7.384

Chad,TCD,2000,7.354

Chad,TCD,2001,7.315

Chad,TCD,2002,7.268

Chad,TCD,2003,7.212

Chad,TCD,2004,7.147

Chad,TCD,2005,7.074

Chad,TCD,2006,6.992

Chad,TCD,2007,6.901

Chad,TCD,2008,6.803

Chad,TCD,2009,6.699

Chad,TCD,2010,6.592

Chad,TCD,2011,6.482

Chad,TCD,2012,6.372

Chad,TCD,2013,6.263

Chad,TCD,2014,6.155

Chad,TCD,2015,6.05

Channel Islands,OWID_CIS,1950,2.053

Channel Islands,OWID_CIS,1951,2.047

Channel Islands,OWID_CIS,1952,2.042

Channel Islands,OWID_CIS,1953,2.05

Channel Islands,OWID_CIS,1954,2.072

Channel Islands,OWID_CIS,1955,2.108

Channel Islands,OWID_CIS,1956,2.156

Channel Islands,OWID_CIS,1957,2.215

Channel Islands,OWID_CIS,1958,2.282

Channel Islands,OWID_CIS,1959,2.353

Channel Islands,OWID_CIS,1960,2.421

Channel Islands,OWID_CIS,1961,2.481

Channel Islands,OWID_CIS,1962,2.525

Channel Islands,OWID_CIS,1963,2.55

Channel Islands,OWID_CIS,1964,2.551

Channel Islands,OWID_CIS,1965,2.526

Channel Islands,OWID_CIS,1966,2.475

Channel Islands,OWID_CIS,1967,2.404

Channel Islands,OWID_CIS,1968,2.318

Channel Islands,OWID_CIS,1969,2.222

Channel Islands,OWID_CIS,1970,2.121

Channel Islands,OWID_CIS,1971,2.019

Channel Islands,OWID_CIS,1972,1.918

Channel Islands,OWID_CIS,1973,1.822

Channel Islands,OWID_CIS,1974,1.735

Channel Islands,OWID_CIS,1975,1.659

Channel Islands,OWID_CIS,1976,1.596

Channel Islands,OWID_CIS,1977,1.546

Channel Islands,OWID_CIS,1978,1.505

Channel Islands,OWID_CIS,1979,1.475

Channel Islands,OWID_CIS,1980,1.454

Channel Islands,OWID_CIS,1981,1.441

Channel Islands,OWID_CIS,1982,1.434

Channel Islands,OWID_CIS,1983,1.433

Channel Islands,OWID_CIS,1984,1.434

Channel Islands,OWID_CIS,1985,1.437

Channel Islands,OWID_CIS,1986,1.441

Channel Islands,OWID_CIS,1987,1.446

Channel Islands,OWID_CIS,1988,1.452

Channel Islands,OWID_CIS,1989,1.457

Channel Islands,OWID_CIS,1990,1.46

Channel Islands,OWID_CIS,1991,1.461

Channel Islands,OWID_CIS,1992,1.457

Channel Islands,OWID_CIS,1993,1.45

Channel Islands,OWID_CIS,1994,1.441

Channel Islands,OWID_CIS,1995,1.43

Channel Islands,OWID_CIS,1996,1.42

Channel Islands,OWID_CIS,1997,1.411

Channel Islands,OWID_CIS,1998,1.405

Channel Islands,OWID_CIS,1999,1.402

Channel Islands,OWID_CIS,2000,1.402

Channel Islands,OWID_CIS,2001,1.404

Channel Islands,OWID_CIS,2002,1.408

Channel Islands,OWID_CIS,2003,1.411

Channel Islands,OWID_CIS,2004,1.413

Channel Islands,OWID_CIS,2005,1.415

Channel Islands,OWID_CIS,2006,1.417

Channel Islands,OWID_CIS,2007,1.42

Channel Islands,OWID_CIS,2008,1.424

Channel Islands,OWID_CIS,2009,1.43

Channel Islands,OWID_CIS,2010,1.436

Channel Islands,OWID_CIS,2011,1.444

Channel Islands,OWID_CIS,2012,1.451

Channel Islands,OWID_CIS,2013,1.459

Channel Islands,OWID_CIS,2014,1.465

Channel Islands,OWID_CIS,2015,1.472

Chile,CHL,1950,5.083

Chile,CHL,1951,5.103

Chile,CHL,1952,5.139

Chile,CHL,1953,5.166

Chile,CHL,1954,5.185

Chile,CHL,1955,5.194

Chile,CHL,1956,5.194

Chile,CHL,1957,5.185

Chile,CHL,1958,5.167

Chile,CHL,1959,5.139

Chile,CHL,1960,5.102

Chile,CHL,1961,5.054

Chile,CHL,1962,4.996

Chile,CHL,1963,4.926

Chile,CHL,1964,4.844

Chile,CHL,1965,4.747

Chile,CHL,1966,4.632

Chile,CHL,1967,4.499

Chile,CHL,1968,4.351

Chile,CHL,1969,4.189

Chile,CHL,1970,4.019

Chile,CHL,1971,3.844

Chile,CHL,1972,3.672

Chile,CHL,1973,3.506

Chile,CHL,1974,3.353

Chile,CHL,1975,3.215

Chile,CHL,1976,3.095

Chile,CHL,1977,2.993

Chile,CHL,1978,2.906

Chile,CHL,1979,2.834

Chile,CHL,1980,2.778

Chile,CHL,1981,2.737

Chile,CHL,1982,2.709

Chile,CHL,1983,2.691

Chile,CHL,1984,2.678

Chile,CHL,1985,2.667

Chile,CHL,1986,2.654

Chile,CHL,1987,2.637

Chile,CHL,1988,2.614

Chile,CHL,1989,2.584

Chile,CHL,1990,2.547

Chile,CHL,1991,2.503

Chile,CHL,1992,2.456

Chile,CHL,1993,2.408

Chile,CHL,1994,2.36

Chile,CHL,1995,2.313

Chile,CHL,1996,2.269

Chile,CHL,1997,2.225

Chile,CHL,1998,2.183

Chile,CHL,1999,2.144

Chile,CHL,2000,2.107

Chile,CHL,2001,2.074

Chile,CHL,2002,2.046

Chile,CHL,2003,2.022

Chile,CHL,2004,2.001

Chile,CHL,2005,1.982

Chile,CHL,2006,1.964

Chile,CHL,2007,1.945

Chile,CHL,2008,1.925

Chile,CHL,2009,1.903

Chile,CHL,2010,1.879

Chile,CHL,2011,1.856

Chile,CHL,2012,1.834

Chile,CHL,2013,1.815

Chile,CHL,2014,1.798

Chile,CHL,2015,1.785

China,CHN,1950,6.665

China,CHN,1951,6.468

China,CHN,1952,6.111

China,CHN,1953,5.828

China,CHN,1954,5.619

China,CHN,1955,5.482

China,CHN,1956,5.417

China,CHN,1957,5.42

China,CHN,1958,5.484

China,CHN,1959,5.598

China,CHN,1960,5.748

China,CHN,1961,5.919

China,CHN,1962,6.089

China,CHN,1963,6.237

China,CHN,1964,6.346

China,CHN,1965,6.396

China,CHN,1966,6.375

China,CHN,1967,6.286

China,CHN,1968,6.133

China,CHN,1969,5.92

China,CHN,1970,5.648

China,CHN,1971,5.322

China,CHN,1972,4.956

China,CHN,1973,4.57

China,CHN,1974,4.181

China,CHN,1975,3.809

China,CHN,1976,3.472

China,CHN,1977,3.18

China,CHN,1978,2.938

China,CHN,1979,2.753

China,CHN,1980,2.63

China,CHN,1981,2.57

China,CHN,1982,2.56

China,CHN,1983,2.582

China,CHN,1984,2.623

China,CHN,1985,2.661

China,CHN,1986,2.675

China,CHN,1987,2.654

China,CHN,1988,2.593

China,CHN,1989,2.489

China,CHN,1990,2.35

China,CHN,1991,2.187

China,CHN,1992,2.021

China,CHN,1993,1.868

China,CHN,1994,1.739

China,CHN,1995,1.639

China,CHN,1996,1.571

China,CHN,1997,1.527

China,CHN,1998,1.503

China,CHN,1999,1.494

China,CHN,2000,1.497

China,CHN,2001,1.508

China,CHN,2002,1.524

China,CHN,2003,1.54

China,CHN,2004,1.554

China,CHN,2005,1.565

China,CHN,2006,1.572

China,CHN,2007,1.577

China,CHN,2008,1.581

China,CHN,2009,1.586

China,CHN,2010,1.59

China,CHN,2011,1.594

China,CHN,2012,1.599

China,CHN,2013,1.604

China,CHN,2014,1.61

China,CHN,2015,1.617

Colombia,COL,1950,6.774

Colombia,COL,1951,6.768

Colombia,COL,1952,6.759

Colombia,COL,1953,6.754

Colombia,COL,1954,6.754

Colombia,COL,1955,6.759

Colombia,COL,1956,6.767

Colombia,COL,1957,6.779

Colombia,COL,1958,6.792

Colombia,COL,1959,6.802

Colombia,COL,1960,6.807

Colombia,COL,1961,6.801

Colombia,COL,1962,6.779

Colombia,COL,1963,6.736

Colombia,COL,1964,6.668

Colombia,COL,1965,6.567

Colombia,COL,1966,6.425

Colombia,COL,1967,6.246

Colombia,COL,1968,6.034

Colombia,COL,1969,5.797

Colombia,COL,1970,5.548

Colombia,COL,1971,5.3

Colombia,COL,1972,5.067

Colombia,COL,1973,4.857

Colombia,COL,1974,4.675

Colombia,COL,1975,4.523

Colombia,COL,1976,4.396

Colombia,COL,1977,4.284

Colombia,COL,1978,4.177

Colombia,COL,1979,4.071

Colombia,COL,1980,3.965

Colombia,COL,1981,3.855

Colombia,COL,1982,3.744

Colombia,COL,1983,3.634

Colombia,COL,1984,3.526

Colombia,COL,1985,3.42

Colombia,COL,1986,3.32

Colombia,COL,1987,3.227

Colombia,COL,1988,3.142

Colombia,COL,1989,3.065

Colombia,COL,1990,2.994

Colombia,COL,1991,2.927

Colombia,COL,1992,2.86

Colombia,COL,1993,2.794

Colombia,COL,1994,2.727

Colombia,COL,1995,2.66

Colombia,COL,1996,2.596

Colombia,COL,1997,2.535

Colombia,COL,1998,2.481

Colombia,COL,1999,2.432

Colombia,COL,2000,2.389

Colombia,COL,2001,2.349

Colombia,COL,2002,2.311

Colombia,COL,2003,2.274

Colombia,COL,2004,2.236

Colombia,COL,2005,2.197

Colombia,COL,2006,2.157

Colombia,COL,2007,2.118

Colombia,COL,2008,2.08

Colombia,COL,2009,2.044

Colombia,COL,2010,2.01

Colombia,COL,2011,1.978

Colombia,COL,2012,1.948

Colombia,COL,2013,1.921

Colombia,COL,2014,1.897

Colombia,COL,2015,1.874

Comoros,COM,1950,5.686

Comoros,COM,1951,5.771

Comoros,COM,1952,5.934

Comoros,COM,1953,6.085

Comoros,COM,1954,6.223

Comoros,COM,1955,6.348

Comoros,COM,1956,6.46

Comoros,COM,1957,6.56

Comoros,COM,1958,6.648

Comoros,COM,1959,6.725

Comoros,COM,1960,6.792

Comoros,COM,1961,6.849

Comoros,COM,1962,6.897

Comoros,COM,1963,6.939

Comoros,COM,1964,6.975

Comoros,COM,1965,7.004

Comoros,COM,1966,7.027

Comoros,COM,1967,7.044

Comoros,COM,1968,7.055

Comoros,COM,1969,7.06

Comoros,COM,1970,7.061

Comoros,COM,1971,7.06

Comoros,COM,1972,7.058

Comoros,COM,1973,7.055

Comoros,COM,1974,7.052

Comoros,COM,1975,7.052

Comoros,COM,1976,7.055

Comoros,COM,1977,7.061

Comoros,COM,1978,7.069

Comoros,COM,1979,7.075

Comoros,COM,1980,7.078

Comoros,COM,1981,7.072

Comoros,COM,1982,7.056

Comoros,COM,1983,7.026

Comoros,COM,1984,6.982

Comoros,COM,1985,6.921

Comoros,COM,1986,6.842

Comoros,COM,1987,6.749

Comoros,COM,1988,6.644

Comoros,COM,1989,6.531

Comoros,COM,1990,6.412

Comoros,COM,1991,6.291

Comoros,COM,1992,6.171

Comoros,COM,1993,6.053

Comoros,COM,1994,5.941

Comoros,COM,1995,5.835

Comoros,COM,1996,5.736

Comoros,COM,1997,5.642

Comoros,COM,1998,5.552

Comoros,COM,1999,5.466

Comoros,COM,2000,5.384

Comoros,COM,2001,5.307

Comoros,COM,2002,5.235

Comoros,COM,2003,5.167

Comoros,COM,2004,5.103

Comoros,COM,2005,5.042

Comoros,COM,2006,4.984

Comoros,COM,2007,4.927

Comoros,COM,2008,4.87

Comoros,COM,2009,4.813

Comoros,COM,2010,4.754

Comoros,COM,2011,4.693

Comoros,COM,2012,4.628

Comoros,COM,2013,4.561

Comoros,COM,2014,4.491

Comoros,COM,2015,4.42

Congo,COG,1950,5.67

Congo,COG,1951,5.671

Congo,COG,1952,5.676

Congo,COG,1953,5.685

Congo,COG,1954,5.7

Congo,COG,1955,5.719

Congo,COG,1956,5.743

Congo,COG,1957,5.771

Congo,COG,1958,5.804

Congo,COG,1959,5.84

Congo,COG,1960,5.88

Congo,COG,1961,5.921

Congo,COG,1962,5.965

Congo,COG,1963,6.008

Congo,COG,1964,6.052

Congo,COG,1965,6.094

Congo,COG,1966,6.136

Congo,COG,1967,6.177

Congo,COG,1968,6.216

Congo,COG,1969,6.253

Congo,COG,1970,6.288

Congo,COG,1971,6.319

Congo,COG,1972,6.347

Congo,COG,1973,6.369

Congo,COG,1974,6.384

Congo,COG,1975,6.389

Congo,COG,1976,6.382

Congo,COG,1977,6.36

Congo,COG,1978,6.324

Congo,COG,1979,6.273

Congo,COG,1980,6.21

Congo,COG,1981,6.134

Congo,COG,1982,6.05

Congo,COG,1983,5.959

Congo,COG,1984,5.866

Congo,COG,1985,5.773

Congo,COG,1986,5.681

Congo,COG,1987,5.59

Congo,COG,1988,5.503

Congo,COG,1989,5.422

Congo,COG,1990,5.349

Congo,COG,1991,5.287

Congo,COG,1992,5.236

Congo,COG,1993,5.195

Congo,COG,1994,5.165

Congo,COG,1995,5.144

Congo,COG,1996,5.13

Congo,COG,1997,5.12

Congo,COG,1998,5.112

Congo,COG,1999,5.105

Congo,COG,2000,5.096

Congo,COG,2001,5.086

Congo,COG,2002,5.076

Congo,COG,2003,5.066

Congo,COG,2004,5.054

Congo,COG,2005,5.042

Congo,COG,2006,5.028

Congo,COG,2007,5.012

Congo,COG,2008,4.995

Congo,COG,2009,4.973

Congo,COG,2010,4.948

Congo,COG,2011,4.915

Congo,COG,2012,4.876

Congo,COG,2013,4.83

Congo,COG,2014,4.778

Congo,COG,2015,4.721

Costa Rica,CRI,1950,5.651

Costa Rica,CRI,1951,5.734

Costa Rica,CRI,1952,5.89

Costa Rica,CRI,1953,6.029

Costa Rica,CRI,1954,6.15

Costa Rica,CRI,1955,6.253

Costa Rica,CRI,1956,6.338

Costa Rica,CRI,1957,6.403

Costa Rica,CRI,1958,6.446

Costa Rica,CRI,1959,6.464

Costa Rica,CRI,1960,6.451

Costa Rica,CRI,1961,6.405

Costa Rica,CRI,1962,6.322

Costa Rica,CRI,1963,6.201

Costa Rica,CRI,1964,6.044

Costa Rica,CRI,1965,5.853

Costa Rica,CRI,1966,5.63

Costa Rica,CRI,1967,5.383

Costa Rica,CRI,1968,5.123

Costa Rica,CRI,1969,4.861

Costa Rica,CRI,1970,4.611

Costa Rica,CRI,1971,4.385

Costa Rica,CRI,1972,4.188

Costa Rica,CRI,1973,4.026

Costa Rica,CRI,1974,3.898

Costa Rica,CRI,1975,3.805

Costa Rica,CRI,1976,3.741

Costa Rica,CRI,1977,3.695

Costa Rica,CRI,1978,3.657

Costa Rica,CRI,1979,3.622

Costa Rica,CRI,1980,3.588

Costa Rica,CRI,1981,3.552

Costa Rica,CRI,1982,3.517

Costa Rica,CRI,1983,3.482

Costa Rica,CRI,1984,3.448

Costa Rica,CRI,1985,3.411

Costa Rica,CRI,1986,3.371

Costa Rica,CRI,1987,3.328

Costa Rica,CRI,1988,3.281

Costa Rica,CRI,1989,3.229

Costa Rica,CRI,1990,3.172

Costa Rica,CRI,1991,3.11

Costa Rica,CRI,1992,3.044

Costa Rica,CRI,1993,2.973

Costa Rica,CRI,1994,2.899

Costa Rica,CRI,1995,2.821

Costa Rica,CRI,1996,2.737

Costa Rica,CRI,1997,2.649

Costa Rica,CRI,1998,2.557

Costa Rica,CRI,1999,2.463

Costa Rica,CRI,2000,2.373

Costa Rica,CRI,2001,2.29

Costa Rica,CRI,2002,2.216

Costa Rica,CRI,2003,2.155

Costa Rica,CRI,2004,2.105

Costa Rica,CRI,2005,2.065

Costa Rica,CRI,2006,2.034

Costa Rica,CRI,2007,2.006

Costa Rica,CRI,2008,1.979

Costa Rica,CRI,2009,1.951

Costa Rica,CRI,2010,1.922

Costa Rica,CRI,2011,1.893

Costa Rica,CRI,2012,1.866

Costa Rica,CRI,2013,1.841

Costa Rica,CRI,2014,1.819

Costa Rica,CRI,2015,1.8

Cote d'Ivoire,CIV,1950,7.375

Cote d'Ivoire,CIV,1951,7.395

Cote d'Ivoire,CIV,1952,7.434

Cote d'Ivoire,CIV,1953,7.471

Cote d'Ivoire,CIV,1954,7.507

Cote d'Ivoire,CIV,1955,7.541

Cote d'Ivoire,CIV,1956,7.573

Cote d'Ivoire,CIV,1957,7.604

Cote d'Ivoire,CIV,1958,7.633

Cote d'Ivoire,CIV,1959,7.662

Cote d'Ivoire,CIV,1960,7.691

Cote d'Ivoire,CIV,1961,7.72

Cote d'Ivoire,CIV,1962,7.75

Cote d'Ivoire,CIV,1963,7.781

Cote d'Ivoire,CIV,1964,7.811

Cote d'Ivoire,CIV,1965,7.841

Cote d'Ivoire,CIV,1966,7.868

Cote d'Ivoire,CIV,1967,7.893

Cote d'Ivoire,CIV,1968,7.912

Cote d'Ivoire,CIV,1969,7.927

Cote d'Ivoire,CIV,1970,7.936

Cote d'Ivoire,CIV,1971,7.941

Cote d'Ivoire,CIV,1972,7.942

Cote d'Ivoire,CIV,1973,7.939

Cote d'Ivoire,CIV,1974,7.929

Cote d'Ivoire,CIV,1975,7.91

Cote d'Ivoire,CIV,1976,7.877

Cote d'Ivoire,CIV,1977,7.828

Cote d'Ivoire,CIV,1978,7.763

Cote d'Ivoire,CIV,1979,7.682

Cote d'Ivoire,CIV,1980,7.59

Cote d'Ivoire,CIV,1981,7.488

Cote d'Ivoire,CIV,1982,7.383

Cote d'Ivoire,CIV,1983,7.278

Cote d'Ivoire,CIV,1984,7.176

Cote d'Ivoire,CIV,1985,7.078

Cote d'Ivoire,CIV,1986,6.984

Cote d'Ivoire,CIV,1987,6.892

Cote d'Ivoire,CIV,1988,6.801

Cote d'Ivoire,CIV,1989,6.71

Cote d'Ivoire,CIV,1990,6.622

Cote d'Ivoire,CIV,1991,6.536

Cote d'Ivoire,CIV,1992,6.454

Cote d'Ivoire,CIV,1993,6.374

Cote d'Ivoire,CIV,1994,6.298

Cote d'Ivoire,CIV,1995,6.224

Cote d'Ivoire,CIV,1996,6.152

Cote d'Ivoire,CIV,1997,6.079

Cote d'Ivoire,CIV,1998,6.006

Cote d'Ivoire,CIV,1999,5.932

Cote d'Ivoire,CIV,2000,5.859

Cote d'Ivoire,CIV,2001,5.787

Cote d'Ivoire,CIV,2002,5.717

Cote d'Ivoire,CIV,2003,5.651

Cote d'Ivoire,CIV,2004,5.589

Cote d'Ivoire,CIV,2005,5.531

Cote d'Ivoire,CIV,2006,5.476

Cote d'Ivoire,CIV,2007,5.423

Cote d'Ivoire,CIV,2008,5.372

Cote d'Ivoire,CIV,2009,5.321

Cote d'Ivoire,CIV,2010,5.269

Cote d'Ivoire,CIV,2011,5.216

Cote d'Ivoire,CIV,2012,5.16

Cote d'Ivoire,CIV,2013,5.101

Cote d'Ivoire,CIV,2014,5.039

Cote d'Ivoire,CIV,2015,4.976

Croatia,HRV,1950,2.924

Croatia,HRV,1951,2.87

Croatia,HRV,1952,2.768

Croatia,HRV,1953,2.676

Croatia,HRV,1954,2.595

Croatia,HRV,1955,2.523

Croatia,HRV,1956,2.461

Croatia,HRV,1957,2.408

Croatia,HRV,1958,2.363

Croatia,HRV,1959,2.323

Croatia,HRV,1960,2.288

Croatia,HRV,1961,2.253

Croatia,HRV,1962,2.218

Croatia,HRV,1963,2.18

Croatia,HRV,1964,2.14

Croatia,HRV,1965,2.1

Croatia,HRV,1966,2.062

Croatia,HRV,1967,2.03

Croatia,HRV,1968,2.006

Croatia,HRV,1969,1.989

Croatia,HRV,1970,1.979

Croatia,HRV,1971,1.973

Croatia,HRV,1972,1.967

Croatia,HRV,1973,1.96

Croatia,HRV,1974,1.95

Croatia,HRV,1975,1.937

Croatia,HRV,1976,1.925

Croatia,HRV,1977,1.913

Croatia,HRV,1978,1.903

Croatia,HRV,1979,1.895

Croatia,HRV,1980,1.888

Croatia,HRV,1981,1.879

Croatia,HRV,1982,1.868

Croatia,HRV,1983,1.852

Croatia,HRV,1984,1.831

Croatia,HRV,1985,1.805

Croatia,HRV,1986,1.771

Croatia,HRV,1987,1.731

Croatia,HRV,1988,1.688

Croatia,HRV,1989,1.644

Croatia,HRV,1990,1.604

Croatia,HRV,1991,1.574

Croatia,HRV,1992,1.557

Croatia,HRV,1993,1.552

Croatia,HRV,1994,1.559

Croatia,HRV,1995,1.572

Croatia,HRV,1996,1.582

Croatia,HRV,1997,1.583

Croatia,HRV,1998,1.571

Croatia,HRV,1999,1.546

Croatia,HRV,2000,1.513

Croatia,HRV,2001,1.48

Croatia,HRV,2002,1.454

Croatia,HRV,2003,1.441

Croatia,HRV,2004,1.441

Croatia,HRV,2005,1.454

Croatia,HRV,2006,1.473

Croatia,HRV,2007,1.493

Croatia,HRV,2008,1.508

Croatia,HRV,2009,1.516

Croatia,HRV,2010,1.516

Croatia,HRV,2011,1.509

Croatia,HRV,2012,1.498

Croatia,HRV,2013,1.486

Croatia,HRV,2014,1.474

Croatia,HRV,2015,1.464

Cuba,CUB,1950,4.758

Cuba,CUB,1951,4.564

Cuba,CUB,1952,4.218

Cuba,CUB,1953,3.956

Cuba,CUB,1954,3.778

Cuba,CUB,1955,3.682

Cuba,CUB,1956,3.665

Cuba,CUB,1957,3.721

Cuba,CUB,1958,3.839

Cuba,CUB,1959,4.001

Cuba,CUB,1960,4.182

Cuba,CUB,1961,4.36

Cuba,CUB,1962,4.509

Cuba,CUB,1963,4.606

Cuba,CUB,1964,4.643

Cuba,CUB,1965,4.617

Cuba,CUB,1966,4.54

Cuba,CUB,1967,4.433

Cuba,CUB,1968,4.312

Cuba,CUB,1969,4.18

Cuba,CUB,1970,4.033

Cuba,CUB,1971,3.857

Cuba,CUB,1972,3.646

Cuba,CUB,1973,3.4

Cuba,CUB,1974,3.127

Cuba,CUB,1975,2.844

Cuba,CUB,1976,2.573

Cuba,CUB,1977,2.332

Cuba,CUB,1978,2.134

Cuba,CUB,1979,1.987

Cuba,CUB,1980,1.892

Cuba,CUB,1981,1.843

Cuba,CUB,1982,1.826

Cuba,CUB,1983,1.825

Cuba,CUB,1984,1.832

Cuba,CUB,1985,1.837

Cuba,CUB,1986,1.836

Cuba,CUB,1987,1.826

Cuba,CUB,1988,1.809

Cuba,CUB,1989,1.782

Cuba,CUB,1990,1.75

Cuba,CUB,1991,1.715

Cuba,CUB,1992,1.684

Cuba,CUB,1993,1.66

Cuba,CUB,1994,1.645

Cuba,CUB,1995,1.637

Cuba,CUB,1996,1.635

Cuba,CUB,1997,1.634

Cuba,CUB,1998,1.631

Cuba,CUB,1999,1.625

Cuba,CUB,2000,1.617

Cuba,CUB,2001,1.606

Cuba,CUB,2002,1.594

Cuba,CUB,2003,1.583

Cuba,CUB,2004,1.575

Cuba,CUB,2005,1.57

Cuba,CUB,2006,1.572

Cuba,CUB,2007,1.581

Cuba,CUB,2008,1.596

Cuba,CUB,2009,1.616

Cuba,CUB,2010,1.639

Cuba,CUB,2011,1.663

Cuba,CUB,2012,1.685

Cuba,CUB,2013,1.702

Cuba,CUB,2014,1.713

Cuba,CUB,2015,1.72

Curacao,CUW,1950,4.856

Curacao,CUW,1951,4.928

Curacao,CUW,1952,5.054

Curacao,CUW,1953,5.142

Curacao,CUW,1954,5.193

Curacao,CUW,1955,5.207

Curacao,CUW,1956,5.185

Curacao,CUW,1957,5.128

Curacao,CUW,1958,5.041

Curacao,CUW,1959,4.93

Curacao,CUW,1960,4.801

Curacao,CUW,1961,4.661

Curacao,CUW,1962,4.519

Curacao,CUW,1963,4.381

Curacao,CUW,1964,4.248

Curacao,CUW,1965,4.118

Curacao,CUW,1966,3.983

Curacao,CUW,1967,3.836

Curacao,CUW,1968,3.675

Curacao,CUW,1969,3.501

Curacao,CUW,1970,3.32

Curacao,CUW,1971,3.142

Curacao,CUW,1972,2.976

Curacao,CUW,1973,2.83

Curacao,CUW,1974,2.705

Curacao,CUW,1975,2.604

Curacao,CUW,1976,2.524

Curacao,CUW,1977,2.458

Curacao,CUW,1978,2.402

Curacao,CUW,1979,2.353

Curacao,CUW,1980,2.312

Curacao,CUW,1981,2.282

Curacao,CUW,1982,2.262

Curacao,CUW,1983,2.253

Curacao,CUW,1984,2.254

Curacao,CUW,1985,2.261

Curacao,CUW,1986,2.273

Curacao,CUW,1987,2.286

Curacao,CUW,1988,2.297

Curacao,CUW,1989,2.304

Curacao,CUW,1990,2.305

Curacao,CUW,1991,2.297

Curacao,CUW,1992,2.281

Curacao,CUW,1993,2.259

Curacao,CUW,1994,2.231

Curacao,CUW,1995,2.202

Curacao,CUW,1996,2.173

Curacao,CUW,1997,2.148

Curacao,CUW,1998,2.128

Curacao,CUW,1999,2.113

Curacao,CUW,2000,2.102

Curacao,CUW,2001,2.093

Curacao,CUW,2002,2.081

Curacao,CUW,2003,2.065

Curacao,CUW,2004,2.046

Curacao,CUW,2005,2.026

Curacao,CUW,2006,2.01

Curacao,CUW,2007,2

Curacao,CUW,2008,1.999

Curacao,CUW,2009,2.005

Curacao,CUW,2010,2.018

Curacao,CUW,2011,2.032

Curacao,CUW,2012,2.045

Curacao,CUW,2013,2.053

Curacao,CUW,2014,2.055

Curacao,CUW,2015,2.051

Cyprus,CYP,1950,3.851

Cyprus,CYP,1951,3.808

Cyprus,CYP,1952,3.727

Cyprus,CYP,1953,3.661

Cyprus,CYP,1954,3.609

Cyprus,CYP,1955,3.571

Cyprus,CYP,1956,3.546

Cyprus,CYP,1957,3.531

Cyprus,CYP,1958,3.522

Cyprus,CYP,1959,3.514

Cyprus,CYP,1960,3.5

Cyprus,CYP,1961,3.471

Cyprus,CYP,1962,3.421

Cyprus,CYP,1963,3.346

Cyprus,CYP,1964,3.249

Cyprus,CYP,1965,3.133

Cyprus,CYP,1966,3.01

Cyprus,CYP,1967,2.889

Cyprus,CYP,1968,2.779

Cyprus,CYP,1969,2.686

Cyprus,CYP,1970,2.61

Cyprus,CYP,1971,2.547

Cyprus,CYP,1972,2.491

Cyprus,CYP,1973,2.44

Cyprus,CYP,1974,2.393

Cyprus,CYP,1975,2.352

Cyprus,CYP,1976,2.323

Cyprus,CYP,1977,2.31

Cyprus,CYP,1978,2.312

Cyprus,CYP,1979,2.328

Cyprus,CYP,1980,2.354

Cyprus,CYP,1981,2.385

Cyprus,CYP,1982,2.415

Cyprus,CYP,1983,2.438

Cyprus,CYP,1984,2.453

Cyprus,CYP,1985,2.458

Cyprus,CYP,1986,2.456

Cyprus,CYP,1987,2.45

Cyprus,CYP,1988,2.442

Cyprus,CYP,1989,2.43

Cyprus,CYP,1990,2.411

Cyprus,CYP,1991,2.381

Cyprus,CYP,1992,2.337

Cyprus,CYP,1993,2.278

Cyprus,CYP,1994,2.206

Cyprus,CYP,1995,2.124

Cyprus,CYP,1996,2.036

Cyprus,CYP,1997,1.946

Cyprus,CYP,1998,1.861

Cyprus,CYP,1999,1.783

Cyprus,CYP,2000,1.716

Cyprus,CYP,2001,1.66

Cyprus,CYP,2002,1.614

Cyprus,CYP,2003,1.576

Cyprus,CYP,2004,1.545

Cyprus,CYP,2005,1.52

Cyprus,CYP,2006,1.498

Cyprus,CYP,2007,1.479

Cyprus,CYP,2008,1.461

Cyprus,CYP,2009,1.443

Cyprus,CYP,2010,1.424

Cyprus,CYP,2011,1.405

Cyprus,CYP,2012,1.388

Cyprus,CYP,2013,1.373

Cyprus,CYP,2014,1.36

Cyprus,CYP,2015,1.35

Czech Republic,CZE,1950,2.943

Czech Republic,CZE,1951,2.886

Czech Republic,CZE,1952,2.778

Czech Republic,CZE,1953,2.681

Czech Republic,CZE,1954,2.596

Czech Republic,CZE,1955,2.522

Czech Republic,CZE,1956,2.459

Czech Republic,CZE,1957,2.406

Czech Republic,CZE,1958,2.361

Czech Republic,CZE,1959,2.321

Czech Republic,CZE,1960,2.282

Czech Republic,CZE,1961,2.243

Czech Republic,CZE,1962,2.199

Czech Republic,CZE,1963,2.15

Czech Republic,CZE,1964,2.099

Czech Republic,CZE,1965,2.05

Czech Republic,CZE,1966,2.013

Czech Republic,CZE,1967,1.993

Czech Republic,CZE,1968,1.994

Czech Republic,CZE,1969,2.015

Czech Republic,CZE,1970,2.055

Czech Republic,CZE,1971,2.112

Czech Republic,CZE,1972,2.175

Czech Republic,CZE,1973,2.238

Czech Republic,CZE,1974,2.293

Czech Republic,CZE,1975,2.332

Czech Republic,CZE,1976,2.347

Czech Republic,CZE,1977,2.336

Czech Republic,CZE,1978,2.302

Czech Republic,CZE,1979,2.248

Czech Republic,CZE,1980,2.18

Czech Republic,CZE,1981,2.108

Czech Republic,CZE,1982,2.042

Czech Republic,CZE,1983,1.989

Czech Republic,CZE,1984,1.95

Czech Republic,CZE,1985,1.925

Czech Republic,CZE,1986,1.91

Czech Republic,CZE,1987,1.896

Czech Republic,CZE,1988,1.876

Czech Republic,CZE,1989,1.846

Czech Republic,CZE,1990,1.802

Czech Republic,CZE,1991,1.741

Czech Republic,CZE,1992,1.664

Czech Republic,CZE,1993,1.576

Czech Republic,CZE,1994,1.481

Czech Republic,CZE,1995,1.385

Czech Republic,CZE,1996,1.297

Czech Republic,CZE,1997,1.222

Czech Republic,CZE,1998,1.167

Czech Republic,CZE,1999,1.133

Czech Republic,CZE,2000,1.123

Czech Republic,CZE,2001,1.136

Czech Republic,CZE,2002,1.166

Czech Republic,CZE,2003,1.207

Czech Republic,CZE,2004,1.254

Czech Republic,CZE,2005,1.304

Czech Republic,CZE,2006,1.35

Czech Republic,CZE,2007,1.39

Czech Republic,CZE,2008,1.424

Czech Republic,CZE,2009,1.448

Czech Republic,CZE,2010,1.465

Czech Republic,CZE,2011,1.476

Czech Republic,CZE,2012,1.485

Czech Republic,CZE,2013,1.495

Czech Republic,CZE,2014,1.507

Czech Republic,CZE,2015,1.522

Democratic Republic of Congo,COD,1950,5.994

Democratic Republic of Congo,COD,1951,5.99

Democratic Republic of Congo,COD,1952,5.981

Democratic Republic of Congo,COD,1953,5.975

Democratic Republic of Congo,COD,1954,5.972

Democratic Republic of Congo,COD,1955,5.97

Democratic Republic of Congo,COD,1956,5.972

Democratic Republic of Congo,COD,1957,5.976

Democratic Republic of Congo,COD,1958,5.982

Democratic Republic of Congo,COD,1959,5.99

Democratic Republic of Congo,COD,1960,6.001

Democratic Republic of Congo,COD,1961,6.015

Democratic Republic of Congo,COD,1962,6.03

Democratic Republic of Congo,COD,1963,6.048

Democratic Republic of Congo,COD,1964,6.067

Democratic Republic of Congo,COD,1965,6.089

Democratic Republic of Congo,COD,1966,6.111

Democratic Republic of Congo,COD,1967,6.135

Democratic Republic of Congo,COD,1968,6.161

Democratic Republic of Congo,COD,1969,6.187

Democratic Republic of Congo,COD,1970,6.215

Democratic Republic of Congo,COD,1971,6.244

Democratic Republic of Congo,COD,1972,6.275

Democratic Republic of Congo,COD,1973,6.308

Democratic Republic of Congo,COD,1974,6.341

Democratic Republic of Congo,COD,1975,6.376

Democratic Republic of Congo,COD,1976,6.41

Democratic Republic of Congo,COD,1977,6.444

Democratic Republic of Congo,COD,1978,6.476

Democratic Republic of Congo,COD,1979,6.506

Democratic Republic of Congo,COD,1980,6.535

Democratic Republic of Congo,COD,1981,6.562

Democratic Republic of Congo,COD,1982,6.587

Democratic Republic of Congo,COD,1983,6.612

Democratic Republic of Congo,COD,1984,6.636

Democratic Republic of Congo,COD,1985,6.658

Democratic Republic of Congo,COD,1986,6.679

Democratic Republic of Congo,COD,1987,6.699

Democratic Republic of Congo,COD,1988,6.717

Democratic Republic of Congo,COD,1989,6.733

Democratic Republic of Congo,COD,1990,6.746

Democratic Republic of Congo,COD,1991,6.758

Democratic Republic of Congo,COD,1992,6.767

Democratic Republic of Congo,COD,1993,6.773

Democratic Republic of Congo,COD,1994,6.777

Democratic Republic of Congo,COD,1995,6.779

Democratic Republic of Congo,COD,1996,6.777

Democratic Republic of Congo,COD,1997,6.774

Democratic Republic of Congo,COD,1998,6.768

Democratic Republic of Congo,COD,1999,6.76

Democratic Republic of Congo,COD,2000,6.751

Democratic Republic of Congo,COD,2001,6.74

Democratic Republic of Congo,COD,2002,6.73

Democratic Republic of Congo,COD,2003,6.718

Democratic Republic of Congo,COD,2004,6.705

Democratic Republic of Congo,COD,2005,6.69

Democratic Republic of Congo,COD,2006,6.671

Democratic Republic of Congo,COD,2007,6.649

Democratic Republic of Congo,COD,2008,6.621

Democratic Republic of Congo,COD,2009,6.587

Democratic Republic of Congo,COD,2010,6.544

Democratic Republic of Congo,COD,2011,6.493

Democratic Republic of Congo,COD,2012,6.432

Democratic Republic of Congo,COD,2013,6.363

Democratic Republic of Congo,COD,2014,6.286

Democratic Republic of Congo,COD,2015,6.202

Denmark,DNK,1950,2.575

Denmark,DNK,1951,2.566

Denmark,DNK,1952,2.551

Denmark,DNK,1953,2.541

Denmark,DNK,1954,2.537

Denmark,DNK,1955,2.539

Denmark,DNK,1956,2.546

Denmark,DNK,1957,2.557

Denmark,DNK,1958,2.569

Denmark,DNK,1959,2.581

Denmark,DNK,1960,2.588

Denmark,DNK,1961,2.587

Denmark,DNK,1962,2.575

Denmark,DNK,1963,2.548

Denmark,DNK,1964,2.507

Denmark,DNK,1965,2.453

Denmark,DNK,1966,2.39

Denmark,DNK,1967,2.321

Denmark,DNK,1968,2.251

Denmark,DNK,1969,2.183

Denmark,DNK,1970,2.117

Denmark,DNK,1971,2.055

Denmark,DNK,1972,1.994

Denmark,DNK,1973,1.934

Denmark,DNK,1974,1.876

Denmark,DNK,1975,1.817

Denmark,DNK,1976,1.758

Denmark,DNK,1977,1.697

Denmark,DNK,1978,1.636

Denmark,DNK,1979,1.577

Denmark,DNK,1980,1.524

Denmark,DNK,1981,1.48

Denmark,DNK,1982,1.45

Denmark,DNK,1983,1.435

Denmark,DNK,1984,1.435

Denmark,DNK,1985,1.45

Denmark,DNK,1986,1.479

Denmark,DNK,1987,1.518

Denmark,DNK,1988,1.562

Denmark,DNK,1989,1.608

Denmark,DNK,1990,1.652

Denmark,DNK,1991,1.691

Denmark,DNK,1992,1.722

Denmark,DNK,1993,1.745

Denmark,DNK,1994,1.76

Denmark,DNK,1995,1.767

Denmark,DNK,1996,1.767

Denmark,DNK,1997,1.763

Denmark,DNK,1998,1.758

Denmark,DNK,1999,1.754

Denmark,DNK,2000,1.753

Denmark,DNK,2001,1.757

Denmark,DNK,2002,1.767

Denmark,DNK,2003,1.782

Denmark,DNK,2004,1.799

Denmark,DNK,2005,1.816

Denmark,DNK,2006,1.829

Denmark,DNK,2007,1.833

Denmark,DNK,2008,1.829

Denmark,DNK,2009,1.816

Denmark,DNK,2010,1.796

Denmark,DNK,2011,1.775

Denmark,DNK,2012,1.755

Denmark,DNK,2013,1.742

Denmark,DNK,2014,1.735

Denmark,DNK,2015,1.736

Djibouti,DJI,1950,6.304

Djibouti,DJI,1951,6.304

Djibouti,DJI,1952,6.306

Djibouti,DJI,1953,6.312

Djibouti,DJI,1954,6.322

Djibouti,DJI,1955,6.336

Djibouti,DJI,1956,6.354

Djibouti,DJI,1957,6.376

Djibouti,DJI,1958,6.401

Djibouti,DJI,1959,6.43

Djibouti,DJI,1960,6.461

Djibouti,DJI,1961,6.493

Djibouti,DJI,1962,6.527

Djibouti,DJI,1963,6.561

Djibouti,DJI,1964,6.595

Djibouti,DJI,1965,6.629

Djibouti,DJI,1966,6.665

Djibouti,DJI,1967,6.702

Djibouti,DJI,1968,6.739

Djibouti,DJI,1969,6.774

Djibouti,DJI,1970,6.804

Djibouti,DJI,1971,6.823

Djibouti,DJI,1972,6.83

Djibouti,DJI,1973,6.823

Djibouti,DJI,1974,6.803

Djibouti,DJI,1975,6.77

Djibouti,DJI,1976,6.729

Djibouti,DJI,1977,6.685

Djibouti,DJI,1978,6.639

Djibouti,DJI,1979,6.596

Djibouti,DJI,1980,6.554

Djibouti,DJI,1981,6.513

Djibouti,DJI,1982,6.471

Djibouti,DJI,1983,6.426

Djibouti,DJI,1984,6.378

Djibouti,DJI,1985,6.328

Djibouti,DJI,1986,6.28

Djibouti,DJI,1987,6.234

Djibouti,DJI,1988,6.188

Djibouti,DJI,1989,6.137

Djibouti,DJI,1990,6.073

Djibouti,DJI,1991,5.986

Djibouti,DJI,1992,5.869

Djibouti,DJI,1993,5.721

Djibouti,DJI,1994,5.546

Djibouti,DJI,1995,5.352

Djibouti,DJI,1996,5.152

Djibouti,DJI,1997,4.958

Djibouti,DJI,1998,4.78

Djibouti,DJI,1999,4.622

Djibouti,DJI,2000,4.484

Djibouti,DJI,2001,4.359

Djibouti,DJI,2002,4.24

Djibouti,DJI,2003,4.118

Djibouti,DJI,2004,3.994

Djibouti,DJI,2005,3.867

Djibouti,DJI,2006,3.739

Djibouti,DJI,2007,3.617

Djibouti,DJI,2008,3.502

Djibouti,DJI,2009,3.397

Djibouti,DJI,2010,3.301

Djibouti,DJI,2011,3.213

Djibouti,DJI,2012,3.131

Djibouti,DJI,2013,3.055

Djibouti,DJI,2014,2.982

Djibouti,DJI,2015,2.913

Dominican Republic,DOM,1950,7.507

Dominican Republic,DOM,1951,7.536

Dominican Republic,DOM,1952,7.589

Dominican Republic,DOM,1953,7.629

Dominican Republic,DOM,1954,7.656

Dominican Republic,DOM,1955,7.672

Dominican Republic,DOM,1956,7.675

Dominican Republic,DOM,1957,7.665

Dominican Republic,DOM,1958,7.643

Dominican Republic,DOM,1959,7.606

Dominican Republic,DOM,1960,7.555

Dominican Republic,DOM,1961,7.487

Dominican Republic,DOM,1962,7.404

Dominican Republic,DOM,1963,7.303

Dominican Republic,DOM,1964,7.186

Dominican Republic,DOM,1965,7.053

Dominican Republic,DOM,1966,6.903

Dominican Republic,DOM,1967,6.738

Dominican Republic,DOM,1968,6.561

Dominican Republic,DOM,1969,6.375

Dominican Republic,DOM,1970,6.182

Dominican Republic,DOM,1971,5.983

Dominican Republic,DOM,1972,5.781

Dominican Republic,DOM,1973,5.58

Dominican Republic,DOM,1974,5.382

Dominican Republic,DOM,1975,5.193

Dominican Republic,DOM,1976,5.014

Dominican Republic,DOM,1977,4.847

Dominican Republic,DOM,1978,4.693

Dominican Republic,DOM,1979,4.552

Dominican Republic,DOM,1980,4.422

Dominican Republic,DOM,1981,4.303

Dominican Republic,DOM,1982,4.19

Dominican Republic,DOM,1983,4.082

Dominican Republic,DOM,1984,3.978

Dominican Republic,DOM,1985,3.878

Dominican Republic,DOM,1986,3.783

Dominican Republic,DOM,1987,3.694

Dominican Republic,DOM,1988,3.612

Dominican Republic,DOM,1989,3.537

Dominican Republic,DOM,1990,3.465

Dominican Republic,DOM,1991,3.397

Dominican Republic,DOM,1992,3.329

Dominican Republic,DOM,1993,3.262

Dominican Republic,DOM,1994,3.195

Dominican Republic,DOM,1995,3.13

Dominican Republic,DOM,1996,3.069

Dominican Republic,DOM,1997,3.014

Dominican Republic,DOM,1998,2.966

Dominican Republic,DOM,1999,2.926

Dominican Republic,DOM,2000,2.892

Dominican Republic,DOM,2001,2.862

Dominican Republic,DOM,2002,2.835

Dominican Republic,DOM,2003,2.807

Dominican Republic,DOM,2004,2.779

Dominican Republic,DOM,2005,2.749

Dominican Republic,DOM,2006,2.718

Dominican Republic,DOM,2007,2.687

Dominican Republic,DOM,2008,2.656

Dominican Republic,DOM,2009,2.626

Dominican Republic,DOM,2010,2.597

Dominican Republic,DOM,2011,2.568

Dominican Republic,DOM,2012,2.539

Dominican Republic,DOM,2013,2.51

Dominican Republic,DOM,2014,2.481

Dominican Republic,DOM,2015,2.451

Eastern Africa,,1950,7.07

Eastern Africa,,1951,7.064

Eastern Africa,,1952,7.052

Eastern Africa,,1953,7.044

Eastern Africa,,1954,7.04

Eastern Africa,,1955,7.039

Eastern Africa,,1956,7.042

Eastern Africa,,1957,7.048

Eastern Africa,,1958,7.057

Eastern Africa,,1959,7.068

Eastern Africa,,1960,7.079

Eastern Africa,,1961,7.089

Eastern Africa,,1962,7.098

Eastern Africa,,1963,7.105

Eastern Africa,,1964,7.109

Eastern Africa,,1965,7.111

Eastern Africa,,1966,7.113

Eastern Africa,,1967,7.115

Eastern Africa,,1968,7.119

Eastern Africa,,1969,7.124

Eastern Africa,,1970,7.13

Eastern Africa,,1971,7.135

Eastern Africa,,1972,7.139

Eastern Africa,,1973,7.14

Eastern Africa,,1974,7.137

Eastern Africa,,1975,7.132

Eastern Africa,,1976,7.124

Eastern Africa,,1977,7.114

Eastern Africa,,1978,7.102

Eastern Africa,,1979,7.089

Eastern Africa,,1980,7.074

Eastern Africa,,1981,7.055

Eastern Africa,,1982,7.033

Eastern Africa,,1983,7.005

Eastern Africa,,1984,6.972

Eastern Africa,,1985,6.931

Eastern Africa,,1986,6.882

Eastern Africa,,1987,6.824

Eastern Africa,,1988,6.758

Eastern Africa,,1989,6.686

Eastern Africa,,1990,6.611

Eastern Africa,,1991,6.535

Eastern Africa,,1992,6.461

Eastern Africa,,1993,6.391

Eastern Africa,,1994,6.326

Eastern Africa,,1995,6.264

Eastern Africa,,1996,6.205

Eastern Africa,,1997,6.145

Eastern Africa,,1998,6.083

Eastern Africa,,1999,6.018

Eastern Africa,,2000,5.948

Eastern Africa,,2001,5.875

Eastern Africa,,2002,5.8

Eastern Africa,,2003,5.722

Eastern Africa,,2004,5.642

Eastern Africa,,2005,5.56

Eastern Africa,,2006,5.476

Eastern Africa,,2007,5.389

Eastern Africa,,2008,5.299

Eastern Africa,,2009,5.208

Eastern Africa,,2010,5.116

Eastern Africa,,2011,5.024

Eastern Africa,,2012,4.933

Eastern Africa,,2013,4.844

Eastern Africa,,2014,4.757

Eastern Africa,,2015,4.673

Eastern Asia,,1950,6.102

Eastern Asia,,1951,5.939

Eastern Asia,,1952,5.641

Eastern Asia,,1953,5.401

Eastern Asia,,1954,5.218

Eastern Asia,,1955,5.093

Eastern Asia,,1956,5.023

Eastern Asia,,1957,5.007

Eastern Asia,,1958,5.038

Eastern Asia,,1959,5.11

Eastern Asia,,1960,5.21

Eastern Asia,,1961,5.328

Eastern Asia,,1962,5.447

Eastern Asia,,1963,5.551

Eastern Asia,,1964,5.626

Eastern Asia,,1965,5.657

Eastern Asia,,1966,5.636

Eastern Asia,,1967,5.563

Eastern Asia,,1968,5.441

Eastern Asia,,1969,5.273

Eastern Asia,,1970,5.058

Eastern Asia,,1971,4.797

Eastern Asia,,1972,4.5

Eastern Asia,,1973,4.184

Eastern Asia,,1974,3.861

Eastern Asia,,1975,3.549

Eastern Asia,,1976,3.263

Eastern Asia,,1977,3.014

Eastern Asia,,1978,2.808

Eastern Asia,,1979,2.651

Eastern Asia,,1980,2.546

Eastern Asia,,1981,2.494

Eastern Asia,,1982,2.484

Eastern Asia,,1983,2.5

Eastern Asia,,1984,2.53

Eastern Asia,,1985,2.557

Eastern Asia,,1986,2.563

Eastern Asia,,1987,2.54

Eastern Asia,,1988,2.481

Eastern Asia,,1989,2.388

Eastern Asia,,1990,2.265

Eastern Asia,,1991,2.121

Eastern Asia,,1992,1.975

Eastern Asia,,1993,1.84

Eastern Asia,,1994,1.724

Eastern Asia,,1995,1.632

Eastern Asia,,1996,1.568

Eastern Asia,,1997,1.524

Eastern Asia,,1998,1.497

Eastern Asia,,1999,1.484

Eastern Asia,,2000,1.482

Eastern Asia,,2001,1.488

Eastern Asia,,2002,1.5

Eastern Asia,,2003,1.513

Eastern Asia,,2004,1.526

Eastern Asia,,2005,1.537

Eastern Asia,,2006,1.547

Eastern Asia,,2007,1.555

Eastern Asia,,2008,1.563

Eastern Asia,,2009,1.57

Eastern Asia,,2010,1.578

Eastern Asia,,2011,1.584

Eastern Asia,,2012,1.59

Eastern Asia,,2013,1.596

Eastern Asia,,2014,1.601

Eastern Asia,,2015,1.606

Eastern Europe,,1950,2.883

Eastern Europe,,1951,2.899

Eastern Europe,,1952,2.925

Eastern Europe,,1953,2.936

Eastern Europe,,1954,2.933

Eastern Europe,,1955,2.916

Eastern Europe,,1956,2.885

Eastern Europe,,1957,2.84

Eastern Europe,,1958,2.784

Eastern Europe,,1959,2.717

Eastern Europe,,1960,2.642

Eastern Europe,,1961,2.562

Eastern Europe,,1962,2.48

Eastern Europe,,1963,2.401

Eastern Europe,,1964,2.327

Eastern Europe,,1965,2.264

Eastern Europe,,1966,2.213

Eastern Europe,,1967,2.175

Eastern Europe,,1968,2.149

Eastern Europe,,1969,2.134

Eastern Europe,,1970,2.127

Eastern Europe,,1971,2.125

Eastern Europe,,1972,2.124

Eastern Europe,,1973,2.121

Eastern Europe,,1974,2.115

Eastern Europe,,1975,2.106

Eastern Europe,,1976,2.095

Eastern Europe,,1977,2.085

Eastern Europe,,1978,2.078

Eastern Europe,,1979,2.074

Eastern Europe,,1980,2.075

Eastern Europe,,1981,2.082

Eastern Europe,,1982,2.094

Eastern Europe,,1983,2.108

Eastern Europe,,1984,2.12

Eastern Europe,,1985,2.125

Eastern Europe,,1986,2.114

Eastern Europe,,1987,2.086

Eastern Europe,,1988,2.037

Eastern Europe,,1989,1.97

Eastern Europe,,1990,1.886

Eastern Europe,,1991,1.791

Eastern Europe,,1992,1.691

Eastern Europe,,1993,1.595

Eastern Europe,,1994,1.506

Eastern Europe,,1995,1.43

Eastern Europe,,1996,1.367

Eastern Europe,,1997,1.317

Eastern Europe,,1998,1.279

Eastern Europe,,1999,1.253

Eastern Europe,,2000,1.24

Eastern Europe,,2001,1.24

Eastern Europe,,2002,1.25

Eastern Europe,,2003,1.269

Eastern Europe,,2004,1.294

Eastern Europe,,2005,1.325

Eastern Europe,,2006,1.359

Eastern Europe,,2007,1.396

Eastern Europe,,2008,1.433

Eastern Europe,,2009,1.47

Eastern Europe,,2010,1.503

Eastern Europe,,2011,1.532

Eastern Europe,,2012,1.555

Eastern Europe,,2013,1.575

Eastern Europe,,2014,1.589

Eastern Europe,,2015,1.6

Ecuador,ECU,1950,6.725

Ecuador,ECU,1951,6.733

Ecuador,ECU,1952,6.748

Ecuador,ECU,1953,6.758

Ecuador,ECU,1954,6.765

Ecuador,ECU,1955,6.767

Ecuador,ECU,1956,6.765

Ecuador,ECU,1957,6.76

Ecuador,ECU,1958,6.75

Ecuador,ECU,1959,6.737

Ecuador,ECU,1960,6.721

Ecuador,ECU,1961,6.7

Ecuador,ECU,1962,6.676

Ecuador,ECU,1963,6.646

Ecuador,ECU,1964,6.61

Ecuador,ECU,1965,6.564

Ecuador,ECU,1966,6.507

Ecuador,ECU,1967,6.436

Ecuador,ECU,1968,6.351

Ecuador,ECU,1969,6.252

Ecuador,ECU,1970,6.138

Ecuador,ECU,1971,6.011

Ecuador,ECU,1972,5.873

Ecuador,ECU,1973,5.727

Ecuador,ECU,1974,5.577

Ecuador,ECU,1975,5.426

Ecuador,ECU,1976,5.276

Ecuador,ECU,1977,5.129

Ecuador,ECU,1978,4.988

Ecuador,ECU,1979,4.853

Ecuador,ECU,1980,4.727

Ecuador,ECU,1981,4.611

Ecuador,ECU,1982,4.503

Ecuador,ECU,1983,4.401

Ecuador,ECU,1984,4.305

Ecuador,ECU,1985,4.213

Ecuador,ECU,1986,4.123

Ecuador,ECU,1987,4.034

Ecuador,ECU,1988,3.945

Ecuador,ECU,1989,3.856

Ecuador,ECU,1990,3.767

Ecuador,ECU,1991,3.679

Ecuador,ECU,1992,3.594

Ecuador,ECU,1993,3.514

Ecuador,ECU,1994,3.437

Ecuador,ECU,1995,3.364

Ecuador,ECU,1996,3.294

Ecuador,ECU,1997,3.225

Ecuador,ECU,1998,3.157

Ecuador,ECU,1999,3.09

Ecuador,ECU,2000,3.027

Ecuador,ECU,2001,2.968

Ecuador,ECU,2002,2.914

Ecuador,ECU,2003,2.868

Ecuador,ECU,2004,2.828

Ecuador,ECU,2005,2.793

Ecuador,ECU,2006,2.763

Ecuador,ECU,2007,2.736

Ecuador,ECU,2008,2.71

Ecuador,ECU,2009,2.684

Ecuador,ECU,2010,2.656

Ecuador,ECU,2011,2.628

Ecuador,ECU,2012,2.599

Ecuador,ECU,2013,2.571

Ecuador,ECU,2014,2.542

Ecuador,ECU,2015,2.514

Egypt,EGY,1950,6.724

Egypt,EGY,1951,6.733

Egypt,EGY,1952,6.748

Egypt,EGY,1953,6.759

Egypt,EGY,1954,6.765

Egypt,EGY,1955,6.767

Egypt,EGY,1956,6.764

Egypt,EGY,1957,6.758

Egypt,EGY,1958,6.748

Egypt,EGY,1959,6.734

Egypt,EGY,1960,6.716

Egypt,EGY,1961,6.696

Egypt,EGY,1962,6.673

Egypt,EGY,1963,6.646

Egypt,EGY,1964,6.615

Egypt,EGY,1965,6.577

Egypt,EGY,1966,6.529

Egypt,EGY,1967,6.469

Egypt,EGY,1968,6.399

Egypt,EGY,1969,6.319

Egypt,EGY,1970,6.233

Egypt,EGY,1971,6.144

Egypt,EGY,1972,6.056

Egypt,EGY,1973,5.974

Egypt,EGY,1974,5.899

Egypt,EGY,1975,5.833

Egypt,EGY,1976,5.776

Egypt,EGY,1977,5.727

Egypt,EGY,1978,5.682

Egypt,EGY,1979,5.639

Egypt,EGY,1980,5.6

Egypt,EGY,1981,5.563

Egypt,EGY,1982,5.528

Egypt,EGY,1983,5.489

Egypt,EGY,1984,5.444

Egypt,EGY,1985,5.382

Egypt,EGY,1986,5.294

Egypt,EGY,1987,5.177

Egypt,EGY,1988,5.031

Egypt,EGY,1989,4.857

Egypt,EGY,1990,4.663

Egypt,EGY,1991,4.455

Egypt,EGY,1992,4.247

Egypt,EGY,1993,4.048

Egypt,EGY,1994,3.865

Egypt,EGY,1995,3.705

Egypt,EGY,1996,3.571

Egypt,EGY,1997,3.459

Egypt,EGY,1998,3.367

Egypt,EGY,1999,3.293

Egypt,EGY,2000,3.233

Egypt,EGY,2001,3.181

Egypt,EGY,2002,3.133

Egypt,EGY,2003,3.088

Egypt,EGY,2004,3.047

Egypt,EGY,2005,3.016

Egypt,EGY,2006,3.004

Egypt,EGY,2007,3.016

Egypt,EGY,2008,3.055

Egypt,EGY,2009,3.114

Egypt,EGY,2010,3.185

Egypt,EGY,2011,3.253

Egypt,EGY,2012,3.306

Egypt,EGY,2013,3.335

Egypt,EGY,2014,3.336

Egypt,EGY,2015,3.311

El Salvador,SLV,1950,6.236

El Salvador,SLV,1951,6.269

El Salvador,SLV,1952,6.334

El Salvador,SLV,1953,6.395

El Salvador,SLV,1954,6.452

El Salvador,SLV,1955,6.504

El Salvador,SLV,1956,6.552

El Salvador,SLV,1957,6.594

El Salvador,SLV,1958,6.63

El Salvador,SLV,1959,6.657

El Salvador,SLV,1960,6.674

El Salvador,SLV,1961,6.679

El Salvador,SLV,1962,6.67

El Salvador,SLV,1963,6.645

El Salvador,SLV,1964,6.606

El Salvador,SLV,1965,6.552

El Salvador,SLV,1966,6.488

El Salvador,SLV,1967,6.415

El Salvador,SLV,1968,6.337

El Salvador,SLV,1969,6.255

El Salvador,SLV,1970,6.172

El Salvador,SLV,1971,6.087

El Salvador,SLV,1972,6

El Salvador,SLV,1973,5.91

El Salvador,SLV,1974,5.816

El Salvador,SLV,1975,5.717

El Salvador,SLV,1976,5.609

El Salvador,SLV,1977,5.493

El Salvador,SLV,1978,5.369

El Salvador,SLV,1979,5.237

El Salvador,SLV,1980,5.1

El Salvador,SLV,1981,4.96

El Salvador,SLV,1982,4.82

El Salvador,SLV,1983,4.684

El Salvador,SLV,1984,4.554

El Salvador,SLV,1985,4.433

El Salvador,SLV,1986,4.322

El Salvador,SLV,1987,4.221

El Salvador,SLV,1988,4.129

El Salvador,SLV,1989,4.043

El Salvador,SLV,1990,3.964

El Salvador,SLV,1991,3.889

El Salvador,SLV,1992,3.815

El Salvador,SLV,1993,3.74

El Salvador,SLV,1994,3.662

El Salvador,SLV,1995,3.577

El Salvador,SLV,1996,3.482

El Salvador,SLV,1997,3.377

El Salvador,SLV,1998,3.263

El Salvador,SLV,1999,3.144

El Salvador,SLV,2000,3.022

El Salvador,SLV,2001,2.903

El Salvador,SLV,2002,2.792

El Salvador,SLV,2003,2.691

El Salvador,SLV,2004,2.604

El Salvador,SLV,2005,2.529

El Salvador,SLV,2006,2.465

El Salvador,SLV,2007,2.41

El Salvador,SLV,2008,2.358

El Salvador,SLV,2009,2.31

El Salvador,SLV,2010,2.265

El Salvador,SLV,2011,2.224

El Salvador,SLV,2012,2.187

El Salvador,SLV,2013,2.155

El Salvador,SLV,2014,2.126

El Salvador,SLV,2015,2.101

Equatorial Guinea,GNQ,1950,5.676

Equatorial Guinea,GNQ,1951,5.673

Equatorial Guinea,GNQ,1952,5.668

Equatorial Guinea,GNQ,1953,5.663

Equatorial Guinea,GNQ,1954,5.659

Equatorial Guinea,GNQ,1955,5.655

Equatorial Guinea,GNQ,1956,5.652

Equatorial Guinea,GNQ,1957,5.649

Equatorial Guinea,GNQ,1958,5.648

Equatorial Guinea,GNQ,1959,5.649

Equatorial Guinea,GNQ,1960,5.653

Equatorial Guinea,GNQ,1961,5.661

Equatorial Guinea,GNQ,1962,5.673

Equatorial Guinea,GNQ,1963,5.69

Equatorial Guinea,GNQ,1964,5.711

Equatorial Guinea,GNQ,1965,5.734

Equatorial Guinea,GNQ,1966,5.756

Equatorial Guinea,GNQ,1967,5.776

Equatorial Guinea,GNQ,1968,5.791

Equatorial Guinea,GNQ,1969,5.802

Equatorial Guinea,GNQ,1970,5.808

Equatorial Guinea,GNQ,1971,5.808

Equatorial Guinea,GNQ,1972,5.805

Equatorial Guinea,GNQ,1973,5.8

Equatorial Guinea,GNQ,1974,5.795

Equatorial Guinea,GNQ,1975,5.791

Equatorial Guinea,GNQ,1976,5.791

Equatorial Guinea,GNQ,1977,5.795

Equatorial Guinea,GNQ,1978,5.804

Equatorial Guinea,GNQ,1979,5.817

Equatorial Guinea,GNQ,1980,5.835

Equatorial Guinea,GNQ,1981,5.856

Equatorial Guinea,GNQ,1982,5.88

Equatorial Guinea,GNQ,1983,5.903

Equatorial Guinea,GNQ,1984,5.926

Equatorial Guinea,GNQ,1985,5.946

Equatorial Guinea,GNQ,1986,5.962

Equatorial Guinea,GNQ,1987,5.974

Equatorial Guinea,GNQ,1988,5.982

Equatorial Guinea,GNQ,1989,5.986

Equatorial Guinea,GNQ,1990,5.987

Equatorial Guinea,GNQ,1991,5.985

Equatorial Guinea,GNQ,1992,5.984

Equatorial Guinea,GNQ,1993,5.981

Equatorial Guinea,GNQ,1994,5.978

Equatorial Guinea,GNQ,1995,5.971

Equatorial Guinea,GNQ,1996,5.96

Equatorial Guinea,GNQ,1997,5.941

Equatorial Guinea,GNQ,1998,5.914

Equatorial Guinea,GNQ,1999,5.878

Equatorial Guinea,GNQ,2000,5.834

Equatorial Guinea,GNQ,2001,5.785

Equatorial Guinea,GNQ,2002,5.731

Equatorial Guinea,GNQ,2003,5.675

Equatorial Guinea,GNQ,2004,5.618

Equatorial Guinea,GNQ,2005,5.559

Equatorial Guinea,GNQ,2006,5.498

Equatorial Guinea,GNQ,2007,5.433

Equatorial Guinea,GNQ,2008,5.363

Equatorial Guinea,GNQ,2009,5.288

Equatorial Guinea,GNQ,2010,5.209

Equatorial Guinea,GNQ,2011,5.126

Equatorial Guinea,GNQ,2012,5.04

Equatorial Guinea,GNQ,2013,4.952

Equatorial Guinea,GNQ,2014,4.864

Equatorial Guinea,GNQ,2015,4.775

Eritrea,ERI,1950,6.924

Eritrea,ERI,1951,6.938

Eritrea,ERI,1952,6.962

Eritrea,ERI,1953,6.979

Eritrea,ERI,1954,6.988

Eritrea,ERI,1955,6.99

Eritrea,ERI,1956,6.984

Eritrea,ERI,1957,6.971

Eritrea,ERI,1958,6.952

Eritrea,ERI,1959,6.928

Eritrea,ERI,1960,6.899

Eritrea,ERI,1961,6.868

Eritrea,ERI,1962,6.837

Eritrea,ERI,1963,6.806

Eritrea,ERI,1964,6.778

Eritrea,ERI,1965,6.752

Eritrea,ERI,1966,6.728

Eritrea,ERI,1967,6.706

Eritrea,ERI,1968,6.686

Eritrea,ERI,1969,6.667

Eritrea,ERI,1970,6.65

Eritrea,ERI,1971,6.636

Eritrea,ERI,1972,6.624

Eritrea,ERI,1973,6.615

Eritrea,ERI,1974,6.609

Eritrea,ERI,1975,6.609

Eritrea,ERI,1976,6.614

Eritrea,ERI,1977,6.625

Eritrea,ERI,1978,6.64

Eritrea,ERI,1979,6.658

Eritrea,ERI,1980,6.675

Eritrea,ERI,1981,6.686

Eritrea,ERI,1982,6.69

Eritrea,ERI,1983,6.683

Eritrea,ERI,1984,6.665

Eritrea,ERI,1985,6.636

Eritrea,ERI,1986,6.597

Eritrea,ERI,1987,6.552

Eritrea,ERI,1988,6.502

Eritrea,ERI,1989,6.448

Eritrea,ERI,1990,6.387

Eritrea,ERI,1991,6.316

Eritrea,ERI,1992,6.233

Eritrea,ERI,1993,6.137

Eritrea,ERI,1994,6.03

Eritrea,ERI,1995,5.914

Eritrea,ERI,1996,5.793

Eritrea,ERI,1997,5.67

Eritrea,ERI,1998,5.549

Eritrea,ERI,1999,5.434

Eritrea,ERI,2000,5.328

Eritrea,ERI,2001,5.233

Eritrea,ERI,2002,5.149

Eritrea,ERI,2003,5.073

Eritrea,ERI,2004,5.005

Eritrea,ERI,2005,4.942

Eritrea,ERI,2006,4.881

Eritrea,ERI,2007,4.818

Eritrea,ERI,2008,4.752

Eritrea,ERI,2009,4.681

Eritrea,ERI,2010,4.605

Eritrea,ERI,2011,4.525

Eritrea,ERI,2012,4.444

Eritrea,ERI,2013,4.363

Eritrea,ERI,2014,4.285

Eritrea,ERI,2015,4.208

Estonia,EST,1950,2.089

Estonia,EST,1951,2.082

Estonia,EST,1952,2.068

Estonia,EST,1953,2.053

Estonia,EST,1954,2.038

Estonia,EST,1955,2.023

Estonia,EST,1956,2.007

Estonia,EST,1957,1.992

Estonia,EST,1958,1.977

Estonia,EST,1959,1.964

Estonia,EST,1960,1.953

Estonia,EST,1961,1.946

Estonia,EST,1962,1.943

Estonia,EST,1963,1.945

Estonia,EST,1964,1.953

Estonia,EST,1965,1.967

Estonia,EST,1966,1.987

Estonia,EST,1967,2.012

Estonia,EST,1968,2.041

Estonia,EST,1969,2.07

Estonia,EST,1970,2.097

Estonia,EST,1971,2.118

Estonia,EST,1972,2.129

Estonia,EST,1973,2.132

Estonia,EST,1974,2.125

Estonia,EST,1975,2.112

Estonia,EST,1976,2.095

Estonia,EST,1977,2.079

Estonia,EST,1978,2.066

Estonia,EST,1979,2.06

Estonia,EST,1980,2.063

Estonia,EST,1981,2.078

Estonia,EST,1982,2.103

Estonia,EST,1983,2.134

Estonia,EST,1984,2.166

Estonia,EST,1985,2.189

Estonia,EST,1986,2.193

Estonia,EST,1987,2.171

Estonia,EST,1988,2.121

Estonia,EST,1989,2.043

Estonia,EST,1990,1.943

Estonia,EST,1991,1.828

Estonia,EST,1992,1.711

Estonia,EST,1993,1.602

Estonia,EST,1994,1.507

Estonia,EST,1995,1.432

Estonia,EST,1996,1.378

Estonia,EST,1997,1.341

Estonia,EST,1998,1.319

Estonia,EST,1999,1.311

Estonia,EST,2000,1.318

Estonia,EST,2001,1.341

Estonia,EST,2002,1.378

Estonia,EST,2003,1.426

Estonia,EST,2004,1.481

Estonia,EST,2005,1.535

Estonia,EST,2006,1.582

Estonia,EST,2007,1.617

Estonia,EST,2008,1.639

Estonia,EST,2009,1.646

Estonia,EST,2010,1.641

Estonia,EST,2011,1.63

Estonia,EST,2012,1.618

Estonia,EST,2013,1.61

Estonia,EST,2014,1.61

Estonia,EST,2015,1.617

Ethiopia,ETH,1950,7.35

Ethiopia,ETH,1951,7.298

Ethiopia,ETH,1952,7.201

Ethiopia,ETH,1953,7.118

Ethiopia,ETH,1954,7.048

Ethiopia,ETH,1955,6.992

Ethiopia,ETH,1956,6.948

Ethiopia,ETH,1957,6.917

Ethiopia,ETH,1958,6.897

Ethiopia,ETH,1959,6.886

Ethiopia,ETH,1960,6.88

Ethiopia,ETH,1961,6.877

Ethiopia,ETH,1962,6.875

Ethiopia,ETH,1963,6.872

Ethiopia,ETH,1964,6.867

Ethiopia,ETH,1965,6.864

Ethiopia,ETH,1966,6.867

Ethiopia,ETH,1967,6.88

Ethiopia,ETH,1968,6.903

Ethiopia,ETH,1969,6.937

Ethiopia,ETH,1970,6.978

Ethiopia,ETH,1971,7.02

Ethiopia,ETH,1972,7.06

Ethiopia,ETH,1973,7.094

Ethiopia,ETH,1974,7.121

Ethiopia,ETH,1975,7.143

Ethiopia,ETH,1976,7.167

Ethiopia,ETH,1977,7.195

Ethiopia,ETH,1978,7.23

Ethiopia,ETH,1979,7.271

Ethiopia,ETH,1980,7.316

Ethiopia,ETH,1981,7.36

Ethiopia,ETH,1982,7.397

Ethiopia,ETH,1983,7.424

Ethiopia,ETH,1984,7.437

Ethiopia,ETH,1985,7.435

Ethiopia,ETH,1986,7.418

Ethiopia,ETH,1987,7.387

Ethiopia,ETH,1988,7.347

Ethiopia,ETH,1989,7.298

Ethiopia,ETH,1990,7.246

Ethiopia,ETH,1991,7.193

Ethiopia,ETH,1992,7.143

Ethiopia,ETH,1993,7.094

Ethiopia,ETH,1994,7.046

Ethiopia,ETH,1995,6.995

Ethiopia,ETH,1996,6.935

Ethiopia,ETH,1997,6.861

Ethiopia,ETH,1998,6.769

Ethiopia,ETH,1999,6.659

Ethiopia,ETH,2000,6.529

Ethiopia,ETH,2001,6.38

Ethiopia,ETH,2002,6.216

Ethiopia,ETH,2003,6.044

Ethiopia,ETH,2004,5.867

Ethiopia,ETH,2005,5.69

Ethiopia,ETH,2006,5.519

Ethiopia,ETH,2007,5.355

Ethiopia,ETH,2008,5.201

Ethiopia,ETH,2009,5.057

Ethiopia,ETH,2010,4.924

Ethiopia,ETH,2011,4.798

Ethiopia,ETH,2012,4.677

Ethiopia,ETH,2013,4.556

Ethiopia,ETH,2014,4.437

Ethiopia,ETH,2015,4.317

Europe,,1950,2.643

Europe,,1951,2.649

Europe,,1952,2.661

Europe,,1953,2.669

Europe,,1954,2.674

Europe,,1955,2.676

Europe,,1956,2.674

Europe,,1957,2.669

Europe,,1958,2.66

Europe,,1959,2.648

Europe,,1960,2.631

Europe,,1961,2.609

Europe,,1962,2.582

Europe,,1963,2.551

Europe,,1964,2.516

Europe,,1965,2.477

Europe,,1966,2.437

Europe,,1967,2.395

Europe,,1968,2.353

Europe,,1969,2.312

Europe,,1970,2.271

Europe,,1971,2.23

Europe,,1972,2.189

Europe,,1973,2.148

Europe,,1974,2.107

Europe,,1975,2.067

Europe,,1976,2.03

Europe,,1977,1.996

Europe,,1978,1.966

Europe,,1979,1.94

Europe,,1980,1.919

Europe,,1981,1.902

Europe,,1982,1.889

Europe,,1983,1.878

Europe,,1984,1.868

Europe,,1985,1.855

Europe,,1986,1.837

Europe,,1987,1.812

Europe,,1988,1.779

Europe,,1989,1.74

Europe,,1990,1.696

Europe,,1991,1.648

Europe,,1992,1.6

Europe,,1993,1.556

Europe,,1994,1.516

Europe,,1995,1.483

Europe,,1996,1.457

Europe,,1997,1.437

Europe,,1998,1.423

Europe,,1999,1.415

Europe,,2000,1.413

Europe,,2001,1.418

Europe,,2002,1.428

Europe,,2003,1.444

Europe,,2004,1.464

Europe,,2005,1.486

Europe,,2006,1.508

Europe,,2007,1.53

Europe,,2008,1.55

Europe,,2009,1.568

Europe,,2010,1.582

Europe,,2011,1.592

Europe,,2012,1.6

Europe,,2013,1.606

Europe,,2014,1.612

Europe,,2015,1.616

Fiji,FJI,1950,6.297

Fiji,FJI,1951,6.402

Fiji,FJI,1952,6.59

Fiji,FJI,1953,6.731

Fiji,FJI,1954,6.824

Fiji,FJI,1955,6.871

Fiji,FJI,1956,6.871

Fiji,FJI,1957,6.827

Fiji,FJI,1958,6.74

Fiji,FJI,1959,6.616

Fiji,FJI,1960,6.461

Fiji,FJI,1961,6.281

Fiji,FJI,1962,6.085

Fiji,FJI,1963,5.881

Fiji,FJI,1964,5.676

Fiji,FJI,1965,5.475

Fiji,FJI,1966,5.278

Fiji,FJI,1967,5.083

Fiji,FJI,1968,4.892

Fiji,FJI,1969,4.71

Fiji,FJI,1970,4.543

Fiji,FJI,1971,4.397

Fiji,FJI,1972,4.275

Fiji,FJI,1973,4.178

Fiji,FJI,1974,4.106

Fiji,FJI,1975,4.054

Fiji,FJI,1976,4.018

Fiji,FJI,1977,3.992

Fiji,FJI,1978,3.968

Fiji,FJI,1979,3.941

Fiji,FJI,1980,3.907

Fiji,FJI,1981,3.864

Fiji,FJI,1982,3.813

Fiji,FJI,1983,3.755

Fiji,FJI,1984,3.693

Fiji,FJI,1985,3.629

Fiji,FJI,1986,3.568

Fiji,FJI,1987,3.512

Fiji,FJI,1988,3.465

Fiji,FJI,1989,3.427

Fiji,FJI,1990,3.398

Fiji,FJI,1991,3.374

Fiji,FJI,1992,3.352

Fiji,FJI,1993,3.33

Fiji,FJI,1994,3.305

Fiji,FJI,1995,3.276

Fiji,FJI,1996,3.244

Fiji,FJI,1997,3.209

Fiji,FJI,1998,3.171

Fiji,FJI,1999,3.132

Fiji,FJI,2000,3.09

Fiji,FJI,2001,3.046

Fiji,FJI,2002,3.001

Fiji,FJI,2003,2.954

Fiji,FJI,2004,2.907

Fiji,FJI,2005,2.86

Fiji,FJI,2006,2.816

Fiji,FJI,2007,2.774

Fiji,FJI,2008,2.735

Fiji,FJI,2009,2.701

Fiji,FJI,2010,2.669

Fiji,FJI,2011,2.641

Fiji,FJI,2012,2.615

Fiji,FJI,2013,2.59

Fiji,FJI,2014,2.565

Fiji,FJI,2015,2.541

Finland,FIN,1950,3.132

Finland,FIN,1951,3.094

Finland,FIN,1952,3.021

Finland,FIN,1953,2.958

Finland,FIN,1954,2.905

Finland,FIN,1955,2.861

Finland,FIN,1956,2.826

Finland,FIN,1957,2.798

Finland,FIN,1958,2.776

Finland,FIN,1959,2.755

Finland,FIN,1960,2.733

Finland,FIN,1961,2.705

Finland,FIN,1962,2.666

Finland,FIN,1963,2.613

Finland,FIN,1964,2.544

Finland,FIN,1965,2.458

Finland,FIN,1966,2.355

Finland,FIN,1967,2.237

Finland,FIN,1968,2.113

Finland,FIN,1969,1.987

Finland,FIN,1970,1.869

Finland,FIN,1971,1.768

Finland,FIN,1972,1.689

Finland,FIN,1973,1.634

Finland,FIN,1974,1.605

Finland,FIN,1975,1.598

Finland,FIN,1976,1.609

Finland,FIN,1977,1.628

Finland,FIN,1978,1.648

Finland,FIN,1979,1.665

Finland,FIN,1980,1.676

Finland,FIN,1981,1.68

Finland,FIN,1982,1.678

Finland,FIN,1983,1.673

Finland,FIN,1984,1.667

Finland,FIN,1985,1.663

Finland,FIN,1986,1.664

Finland,FIN,1987,1.674

Finland,FIN,1988,1.691

Finland,FIN,1989,1.715

Finland,FIN,1990,1.743

Finland,FIN,1991,1.769

Finland,FIN,1992,1.789

Finland,FIN,1993,1.8

Finland,FIN,1994,1.8

Finland,FIN,1995,1.792

Finland,FIN,1996,1.777

Finland,FIN,1997,1.761

Finland,FIN,1998,1.748

Finland,FIN,1999,1.74

Finland,FIN,2000,1.737

Finland,FIN,2001,1.743

Finland,FIN,2002,1.754

Finland,FIN,2003,1.768

Finland,FIN,2004,1.786

Finland,FIN,2005,1.802

Finland,FIN,2006,1.816

Finland,FIN,2007,1.824

Finland,FIN,2008,1.825

Finland,FIN,2009,1.82

Finland,FIN,2010,1.81

Finland,FIN,2011,1.798

Finland,FIN,2012,1.786

Finland,FIN,2013,1.777

Finland,FIN,2014,1.771

Finland,FIN,2015,1.77

France,FRA,1950,2.834

France,FRA,1951,2.806

France,FRA,1952,2.756

France,FRA,1953,2.719

France,FRA,1954,2.695

France,FRA,1955,2.684

France,FRA,1956,2.685

France,FRA,1957,2.697

France,FRA,1958,2.718

France,FRA,1959,2.744

France,FRA,1960,2.771

France,FRA,1961,2.795

France,FRA,1962,2.81

France,FRA,1963,2.812

France,FRA,1964,2.8

France,FRA,1965,2.772

France,FRA,1966,2.73

France,FRA,1967,2.678

France,FRA,1968,2.62

France,FRA,1969,2.557

France,FRA,1970,2.488

France,FRA,1971,2.412

France,FRA,1972,2.328

France,FRA,1973,2.239

France,FRA,1974,2.147

France,FRA,1975,2.06

France,FRA,1976,1.984

France,FRA,1977,1.922

France,FRA,1978,1.878

France,FRA,1979,1.851

France,FRA,1980,1.84

France,FRA,1981,1.839

France,FRA,1982,1.844

France,FRA,1983,1.847

France,FRA,1984,1.846

France,FRA,1985,1.84

France,FRA,1986,1.827

France,FRA,1987,1.809

France,FRA,1988,1.79

France,FRA,1989,1.77

France,FRA,1990,1.752

France,FRA,1991,1.736

France,FRA,1992,1.724

France,FRA,1993,1.717

France,FRA,1994,1.717

France,FRA,1995,1.723

France,FRA,1996,1.734

France,FRA,1997,1.751

France,FRA,1998,1.771

France,FRA,1999,1.794

France,FRA,2000,1.818

France,FRA,2001,1.844

France,FRA,2002,1.869

France,FRA,2003,1.894

France,FRA,2004,1.918

France,FRA,2005,1.939

France,FRA,2006,1.956

France,FRA,2007,1.97

France,FRA,2008,1.979

France,FRA,2009,1.985

France,FRA,2010,1.988

France,FRA,2011,1.988

France,FRA,2012,1.986

France,FRA,2013,1.984

France,FRA,2014,1.981

France,FRA,2015,1.979

French Guiana,GUF,1950,5.076

French Guiana,GUF,1951,5.053

French Guiana,GUF,1952,5.011

French Guiana,GUF,1953,4.977

French Guiana,GUF,1954,4.951

French Guiana,GUF,1955,4.934

French Guiana,GUF,1956,4.925

French Guiana,GUF,1957,4.924

French Guiana,GUF,1958,4.93

French Guiana,GUF,1959,4.945

French Guiana,GUF,1960,4.967

French Guiana,GUF,1961,4.995

French Guiana,GUF,1962,5.025

French Guiana,GUF,1963,5.054

French Guiana,GUF,1964,5.076

French Guiana,GUF,1965,5.08

French Guiana,GUF,1966,5.059

French Guiana,GUF,1967,5.006

French Guiana,GUF,1968,4.921

French Guiana,GUF,1969,4.803

French Guiana,GUF,1970,4.653

French Guiana,GUF,1971,4.471

French Guiana,GUF,1972,4.267

French Guiana,GUF,1973,4.055

French Guiana,GUF,1974,3.847

French Guiana,GUF,1975,3.659

French Guiana,GUF,1976,3.508

French Guiana,GUF,1977,3.402

French Guiana,GUF,1978,3.343

French Guiana,GUF,1979,3.332

French Guiana,GUF,1980,3.361

French Guiana,GUF,1981,3.417

French Guiana,GUF,1982,3.484

French Guiana,GUF,1983,3.548

French Guiana,GUF,1984,3.603

French Guiana,GUF,1985,3.65

French Guiana,GUF,1986,3.691

French Guiana,GUF,1987,3.737

French Guiana,GUF,1988,3.791

French Guiana,GUF,1989,3.851

French Guiana,GUF,1990,3.912

French Guiana,GUF,1991,3.967

French Guiana,GUF,1992,4.009

French Guiana,GUF,1993,4.034

French Guiana,GUF,1994,4.04

French Guiana,GUF,1995,4.027

French Guiana,GUF,1996,3.998

French Guiana,GUF,1997,3.958

French Guiana,GUF,1998,3.914

French Guiana,GUF,1999,3.868

French Guiana,GUF,2000,3.824

French Guiana,GUF,2001,3.786

French Guiana,GUF,2002,3.752

French Guiana,GUF,2003,3.724

French Guiana,GUF,2004,3.701

French Guiana,GUF,2005,3.681

French Guiana,GUF,2006,3.662

French Guiana,GUF,2007,3.641

French Guiana,GUF,2008,3.617

French Guiana,GUF,2009,3.588

French Guiana,GUF,2010,3.555

French Guiana,GUF,2011,3.517

French Guiana,GUF,2012,3.477

French Guiana,GUF,2013,3.437

French Guiana,GUF,2014,3.397

French Guiana,GUF,2015,3.358

French Polynesia,PYF,1950,5.972

French Polynesia,PYF,1951,5.987

French Polynesia,PYF,1952,6.009

French Polynesia,PYF,1953,6.015

French Polynesia,PYF,1954,6.005

French Polynesia,PYF,1955,5.979

French Polynesia,PYF,1956,5.938

French Polynesia,PYF,1957,5.882

French Polynesia,PYF,1958,5.815

French Polynesia,PYF,1959,5.739

French Polynesia,PYF,1960,5.658

French Polynesia,PYF,1961,5.577

French Polynesia,PYF,1962,5.5

French Polynesia,PYF,1963,5.43

French Polynesia,PYF,1964,5.368

French Polynesia,PYF,1965,5.314

French Polynesia,PYF,1966,5.267

French Polynesia,PYF,1967,5.222

French Polynesia,PYF,1968,5.174

French Polynesia,PYF,1969,5.12

French Polynesia,PYF,1970,5.057

French Polynesia,PYF,1971,4.98

French Polynesia,PYF,1972,4.889

French Polynesia,PYF,1973,4.785

French Polynesia,PYF,1974,4.67

French Polynesia,PYF,1975,4.548

French Polynesia,PYF,1976,4.423

French Polynesia,PYF,1977,4.301

French Polynesia,PYF,1978,4.185

French Polynesia,PYF,1979,4.08

French Polynesia,PYF,1980,3.989

French Polynesia,PYF,1981,3.916

French Polynesia,PYF,1982,3.858

French Polynesia,PYF,1983,3.812

French Polynesia,PYF,1984,3.774

French Polynesia,PYF,1985,3.738

French Polynesia,PYF,1986,3.697

French Polynesia,PYF,1987,3.645

French Polynesia,PYF,1988,3.579

French Polynesia,PYF,1989,3.498

French Polynesia,PYF,1990,3.401

French Polynesia,PYF,1991,3.291

French Polynesia,PYF,1992,3.175

French Polynesia,PYF,1993,3.059

French Polynesia,PYF,1994,2.946

French Polynesia,PYF,1995,2.841

French Polynesia,PYF,1996,2.745

French Polynesia,PYF,1997,2.66

French Polynesia,PYF,1998,2.585

French Polynesia,PYF,1999,2.52

French Polynesia,PYF,2000,2.463

French Polynesia,PYF,2001,2.414

French Polynesia,PYF,2002,2.37

French Polynesia,PYF,2003,2.329

French Polynesia,PYF,2004,2.289

French Polynesia,PYF,2005,2.251

French Polynesia,PYF,2006,2.216

French Polynesia,PYF,2007,2.184

French Polynesia,PYF,2008,2.155

French Polynesia,PYF,2009,2.131

French Polynesia,PYF,2010,2.11

French Polynesia,PYF,2011,2.092

French Polynesia,PYF,2012,2.075

French Polynesia,PYF,2013,2.059

French Polynesia,PYF,2014,2.044

French Polynesia,PYF,2015,2.028

Gabon,GAB,1950,3.948

Gabon,GAB,1951,3.953

Gabon,GAB,1952,3.969

Gabon,GAB,1953,3.992

Gabon,GAB,1954,4.025

Gabon,GAB,1955,4.066

Gabon,GAB,1956,4.115

Gabon,GAB,1957,4.173

Gabon,GAB,1958,4.237

Gabon,GAB,1959,4.308

Gabon,GAB,1960,4.384

Gabon,GAB,1961,4.462

Gabon,GAB,1962,4.541

Gabon,GAB,1963,4.619

Gabon,GAB,1964,4.694

Gabon,GAB,1965,4.766

Gabon,GAB,1966,4.834

Gabon,GAB,1967,4.899

Gabon,GAB,1968,4.961

Gabon,GAB,1969,5.021

Gabon,GAB,1970,5.081

Gabon,GAB,1971,5.143

Gabon,GAB,1972,5.208

Gabon,GAB,1973,5.275

Gabon,GAB,1974,5.343

Gabon,GAB,1975,5.412

Gabon,GAB,1976,5.48

Gabon,GAB,1977,5.543

Gabon,GAB,1978,5.6

Gabon,GAB,1979,5.648

Gabon,GAB,1980,5.684

Gabon,GAB,1981,5.709

Gabon,GAB,1982,5.722

Gabon,GAB,1983,5.723

Gabon,GAB,1984,5.712

Gabon,GAB,1985,5.689

Gabon,GAB,1986,5.654

Gabon,GAB,1987,5.609

Gabon,GAB,1988,5.554

Gabon,GAB,1989,5.491

Gabon,GAB,1990,5.421

Gabon,GAB,1991,5.345

Gabon,GAB,1992,5.263

Gabon,GAB,1993,5.176

Gabon,GAB,1994,5.087

Gabon,GAB,1995,4.996

Gabon,GAB,1996,4.903

Gabon,GAB,1997,4.809

Gabon,GAB,1998,4.716

Gabon,GAB,1999,4.625

Gabon,GAB,2000,4.539

Gabon,GAB,2001,4.46

Gabon,GAB,2002,4.389

Gabon,GAB,2003,4.327

Gabon,GAB,2004,4.274

Gabon,GAB,2005,4.23

Gabon,GAB,2006,4.194

Gabon,GAB,2007,4.164

Gabon,GAB,2008,4.137

Gabon,GAB,2009,4.112

Gabon,GAB,2010,4.083

Gabon,GAB,2011,4.05

Gabon,GAB,2012,4.01

Gabon,GAB,2013,3.963

Gabon,GAB,2014,3.91

Gabon,GAB,2015,3.85

Gambia,GMB,1950,5.23

Gambia,GMB,1951,5.244

Gambia,GMB,1952,5.271

Gambia,GMB,1953,5.301

Gambia,GMB,1954,5.333

Gambia,GMB,1955,5.367

Gambia,GMB,1956,5.404

Gambia,GMB,1957,5.443

Gambia,GMB,1958,5.484

Gambia,GMB,1959,5.527

Gambia,GMB,1960,5.573

Gambia,GMB,1961,5.621

Gambia,GMB,1962,5.672

Gambia,GMB,1963,5.723

Gambia,GMB,1964,5.776

Gambia,GMB,1965,5.83

Gambia,GMB,1966,5.884

Gambia,GMB,1967,5.938

Gambia,GMB,1968,5.991

Gambia,GMB,1969,6.043

Gambia,GMB,1970,6.093

Gambia,GMB,1971,6.141

Gambia,GMB,1972,6.185

Gambia,GMB,1973,6.226

Gambia,GMB,1974,6.262

Gambia,GMB,1975,6.293

Gambia,GMB,1976,6.316

Gambia,GMB,1977,6.333

Gambia,GMB,1978,6.342

Gambia,GMB,1979,6.343

Gambia,GMB,1980,6.337

Gambia,GMB,1981,6.323

Gambia,GMB,1982,6.303

Gambia,GMB,1983,6.277

Gambia,GMB,1984,6.248

Gambia,GMB,1985,6.219

Gambia,GMB,1986,6.19

Gambia,GMB,1987,6.164

Gambia,GMB,1988,6.141

Gambia,GMB,1989,6.122

Gambia,GMB,1990,6.107

Gambia,GMB,1991,6.094

Gambia,GMB,1992,6.082

Gambia,GMB,1993,6.069

Gambia,GMB,1994,6.054

Gambia,GMB,1995,6.038

Gambia,GMB,1996,6.021

Gambia,GMB,1997,6.002

Gambia,GMB,1998,5.984

Gambia,GMB,1999,5.966

Gambia,GMB,2000,5.947

Gambia,GMB,2001,5.927

Gambia,GMB,2002,5.906

Gambia,GMB,2003,5.884

Gambia,GMB,2004,5.86

Gambia,GMB,2005,5.835

Gambia,GMB,2006,5.81

Gambia,GMB,2007,5.785

Gambia,GMB,2008,5.76

Gambia,GMB,2009,5.735

Gambia,GMB,2010,5.707

Gambia,GMB,2011,5.676

Gambia,GMB,2012,5.638

Gambia,GMB,2013,5.595

Gambia,GMB,2014,5.544

Gambia,GMB,2015,5.488

Georgia,GEO,1950,2.655

Georgia,GEO,1951,2.681

Georgia,GEO,1952,2.731

Georgia,GEO,1953,2.776

Georgia,GEO,1954,2.816

Georgia,GEO,1955,2.851

Georgia,GEO,1956,2.881

Georgia,GEO,1957,2.905

Georgia,GEO,1958,2.923

Georgia,GEO,1959,2.936

Georgia,GEO,1960,2.942

Georgia,GEO,1961,2.943

Georgia,GEO,1962,2.937

Georgia,GEO,1963,2.924

Georgia,GEO,1964,2.906

Georgia,GEO,1965,2.882

Georgia,GEO,1966,2.854

Georgia,GEO,1967,2.821

Georgia,GEO,1968,2.785

Georgia,GEO,1969,2.747

Georgia,GEO,1970,2.707

Georgia,GEO,1971,2.665

Georgia,GEO,1972,2.622

Georgia,GEO,1973,2.577

Georgia,GEO,1974,2.533

Georgia,GEO,1975,2.489

Georgia,GEO,1976,2.448

Georgia,GEO,1977,2.409

Georgia,GEO,1978,2.373

Georgia,GEO,1979,2.341

Georgia,GEO,1980,2.314

Georgia,GEO,1981,2.296

Georgia,GEO,1982,2.284

Georgia,GEO,1983,2.277

Georgia,GEO,1984,2.275

Georgia,GEO,1985,2.273

Georgia,GEO,1986,2.269

Georgia,GEO,1987,2.26

Georgia,GEO,1988,2.243

Georgia,GEO,1989,2.217

Georgia,GEO,1990,2.18

Georgia,GEO,1991,2.132

Georgia,GEO,1992,2.076

Georgia,GEO,1993,2.013

Georgia,GEO,1994,1.946

Georgia,GEO,1995,1.879

Georgia,GEO,1996,1.812

Georgia,GEO,1997,1.749

Georgia,GEO,1998,1.692

Georgia,GEO,1999,1.643

Georgia,GEO,2000,1.608

Georgia,GEO,2001,1.589

Georgia,GEO,2002,1.586

Georgia,GEO,2003,1.599

Georgia,GEO,2004,1.625

Georgia,GEO,2005,1.664

Georgia,GEO,2006,1.712

Georgia,GEO,2007,1.766

Georgia,GEO,2008,1.821

Georgia,GEO,2009,1.873

Georgia,GEO,2010,1.918

Georgia,GEO,2011,1.954

Georgia,GEO,2012,1.98

Georgia,GEO,2013,1.996

Georgia,GEO,2014,2.003

Georgia,GEO,2015,2.003

Germany,DEU,1950,2.089

Germany,DEU,1951,2.097

Germany,DEU,1952,2.114

Germany,DEU,1953,2.135

Germany,DEU,1954,2.162

Germany,DEU,1955,2.193

Germany,DEU,1956,2.228

Germany,DEU,1957,2.267

Germany,DEU,1958,2.309

Germany,DEU,1959,2.353

Germany,DEU,1960,2.395

Germany,DEU,1961,2.434

Germany,DEU,1962,2.466

Germany,DEU,1963,2.488

Germany,DEU,1964,2.495

Germany,DEU,1965,2.481

Germany,DEU,1966,2.44

Germany,DEU,1967,2.371

Germany,DEU,1968,2.278

Germany,DEU,1969,2.166

Germany,DEU,1970,2.042

Germany,DEU,1971,1.917

Germany,DEU,1972,1.8

Germany,DEU,1973,1.7

Germany,DEU,1974,1.62

Germany,DEU,1975,1.563

Germany,DEU,1976,1.527

Germany,DEU,1977,1.504

Germany,DEU,1978,1.489

Germany,DEU,1979,1.479

Germany,DEU,1980,1.472

Germany,DEU,1981,1.466

Germany,DEU,1982,1.463

Germany,DEU,1983,1.46

Germany,DEU,1984,1.458

Germany,DEU,1985,1.452

Germany,DEU,1986,1.442

Germany,DEU,1987,1.426

Germany,DEU,1988,1.406

Germany,DEU,1989,1.383

Germany,DEU,1990,1.359

Germany,DEU,1991,1.337

Germany,DEU,1992,1.321

Germany,DEU,1993,1.311

Germany,DEU,1994,1.309

Germany,DEU,1995,1.313

Germany,DEU,1996,1.322

Germany,DEU,1997,1.331

Germany,DEU,1998,1.34

Germany,DEU,1999,1.347

Germany,DEU,2000,1.35

Germany,DEU,2001,1.351

Germany,DEU,2002,1.351

Germany,DEU,2003,1.351

Germany,DEU,2004,1.351

Germany,DEU,2005,1.352

Germany,DEU,2006,1.356

Germany,DEU,2007,1.362

Germany,DEU,2008,1.37

Germany,DEU,2009,1.38

Germany,DEU,2010,1.392

Germany,DEU,2011,1.405

Germany,DEU,2012,1.418

Germany,DEU,2013,1.43

Germany,DEU,2014,1.441

Germany,DEU,2015,1.45

Ghana,GHA,1950,6.367

Ghana,GHA,1951,6.385

Ghana,GHA,1952,6.422

Ghana,GHA,1953,6.46

Ghana,GHA,1954,6.499

Ghana,GHA,1955,6.539

Ghana,GHA,1956,6.58

Ghana,GHA,1957,6.622

Ghana,GHA,1958,6.665

Ghana,GHA,1959,6.707

Ghana,GHA,1960,6.749

Ghana,GHA,1961,6.789

Ghana,GHA,1962,6.827

Ghana,GHA,1963,6.862

Ghana,GHA,1964,6.893

Ghana,GHA,1965,6.918

Ghana,GHA,1966,6.937

Ghana,GHA,1967,6.95

Ghana,GHA,1968,6.957

Ghana,GHA,1969,6.957

Ghana,GHA,1970,6.95

Ghana,GHA,1971,6.936

Ghana,GHA,1972,6.915

Ghana,GHA,1973,6.888

Ghana,GHA,1974,6.855

Ghana,GHA,1975,6.815

Ghana,GHA,1976,6.771

Ghana,GHA,1977,6.721

Ghana,GHA,1978,6.665

Ghana,GHA,1979,6.605

Ghana,GHA,1980,6.539

Ghana,GHA,1981,6.468

Ghana,GHA,1982,6.392

Ghana,GHA,1983,6.31

Ghana,GHA,1984,6.223

Ghana,GHA,1985,6.131

Ghana,GHA,1986,6.032

Ghana,GHA,1987,5.928

Ghana,GHA,1988,5.82

Ghana,GHA,1989,5.71

Ghana,GHA,1990,5.602

Ghana,GHA,1991,5.499

Ghana,GHA,1992,5.403

Ghana,GHA,1993,5.317

Ghana,GHA,1994,5.239

Ghana,GHA,1995,5.168

Ghana,GHA,1996,5.102

Ghana,GHA,1997,5.036

Ghana,GHA,1998,4.969

Ghana,GHA,1999,4.899

Ghana,GHA,2000,4.826

Ghana,GHA,2001,4.753

Ghana,GHA,2002,4.681

Ghana,GHA,2003,4.612

Ghana,GHA,2004,4.548

Ghana,GHA,2005,4.49

Ghana,GHA,2006,4.438

Ghana,GHA,2007,4.392

Ghana,GHA,2008,4.35

Ghana,GHA,2009,4.311

Ghana,GHA,2010,4.273

Ghana,GHA,2011,4.234

Ghana,GHA,2012,4.192

Ghana,GHA,2013,4.146

Ghana,GHA,2014,4.096

Ghana,GHA,2015,4.042

Greece,GRC,1950,2.467

Greece,GRC,1951,2.475

Greece,GRC,1952,2.485

Greece,GRC,1953,2.487

Greece,GRC,1954,2.48

Greece,GRC,1955,2.466

Greece,GRC,1956,2.444

Greece,GRC,1957,2.416

Greece,GRC,1958,2.385

Greece,GRC,1959,2.355

Greece,GRC,1960,2.33

Greece,GRC,1961,2.316

Greece,GRC,1962,2.318

Greece,GRC,1963,2.336

Greece,GRC,1964,2.369

Greece,GRC,1965,2.414

Greece,GRC,1966,2.462

Greece,GRC,1967,2.507

Greece,GRC,1968,2.541

Greece,GRC,1969,2.562

Greece,GRC,1970,2.569

Greece,GRC,1971,2.565

Greece,GRC,1972,2.554

Greece,GRC,1973,2.54

Greece,GRC,1974,2.523

Greece,GRC,1975,2.502

Greece,GRC,1976,2.474

Greece,GRC,1977,2.438

Greece,GRC,1978,2.392

Greece,GRC,1979,2.335

Greece,GRC,1980,2.268

Greece,GRC,1981,2.188

Greece,GRC,1982,2.099

Greece,GRC,1983,2.002

Greece,GRC,1984,1.903

Greece,GRC,1985,1.805

Greece,GRC,1986,1.714

Greece,GRC,1987,1.633

Greece,GRC,1988,1.565

Greece,GRC,1989,1.51

Greece,GRC,1990,1.469

Greece,GRC,1991,1.439

Greece,GRC,1992,1.417

Greece,GRC,1993,1.4

Greece,GRC,1994,1.384

Greece,GRC,1995,1.37

Greece,GRC,1996,1.357

Greece,GRC,1997,1.345

Greece,GRC,1998,1.334

Greece,GRC,1999,1.327

Greece,GRC,2000,1.323

Greece,GRC,2001,1.327

Greece,GRC,2002,1.338

Greece,GRC,2003,1.356

Greece,GRC,2004,1.379

Greece,GRC,2005,1.403

Greece,GRC,2006,1.423

Greece,GRC,2007,1.435

Greece,GRC,2008,1.438

Greece,GRC,2009,1.431

Greece,GRC,2010,1.414

Greece,GRC,2011,1.391

Greece,GRC,2012,1.367

Greece,GRC,2013,1.345

Greece,GRC,2014,1.327

Greece,GRC,2015,1.314

Grenada,GRD,1950,5.184

Grenada,GRD,1951,5.358

Grenada,GRD,1952,5.686

Grenada,GRD,1953,5.974

Grenada,GRD,1954,6.221

Grenada,GRD,1955,6.428

Grenada,GRD,1956,6.59

Grenada,GRD,1957,6.709

Grenada,GRD,1958,6.778

Grenada,GRD,1959,6.792

Grenada,GRD,1960,6.743

Grenada,GRD,1961,6.627

Grenada,GRD,1962,6.442

Grenada,GRD,1963,6.196

Grenada,GRD,1964,5.906

Grenada,GRD,1965,5.597

Grenada,GRD,1966,5.3

Grenada,GRD,1967,5.04

Grenada,GRD,1968,4.835

Grenada,GRD,1969,4.69

Grenada,GRD,1970,4.604

Grenada,GRD,1971,4.561

Grenada,GRD,1972,4.537

Grenada,GRD,1973,4.512

Grenada,GRD,1974,4.479

Grenada,GRD,1975,4.436

Grenada,GRD,1976,4.385

Grenada,GRD,1977,4.337

Grenada,GRD,1978,4.299

Grenada,GRD,1979,4.269

Grenada,GRD,1980,4.251

Grenada,GRD,1981,4.244

Grenada,GRD,1982,4.243

Grenada,GRD,1983,4.245

Grenada,GRD,1984,4.243

Grenada,GRD,1985,4.231

Grenada,GRD,1986,4.2

Grenada,GRD,1987,4.146

Grenada,GRD,1988,4.068

Grenada,GRD,1989,3.966

Grenada,GRD,1990,3.842

Grenada,GRD,1991,3.701

Grenada,GRD,1992,3.551

Grenada,GRD,1993,3.401

Grenada,GRD,1994,3.254

Grenada,GRD,1995,3.116

Grenada,GRD,1996,2.988

Grenada,GRD,1997,2.87

Grenada,GRD,1998,2.762

Grenada,GRD,1999,2.666

Grenada,GRD,2000,2.582

Grenada,GRD,2001,2.512

Grenada,GRD,2002,2.455

Grenada,GRD,2003,2.409

Grenada,GRD,2004,2.373

Grenada,GRD,2005,2.344

Grenada,GRD,2006,2.321

Grenada,GRD,2007,2.301

Grenada,GRD,2008,2.282

Grenada,GRD,2009,2.262

Grenada,GRD,2010,2.24

Grenada,GRD,2011,2.217

Grenada,GRD,2012,2.194

Grenada,GRD,2013,2.171

Grenada,GRD,2014,2.148

Grenada,GRD,2015,2.126

Guadeloupe (including Saint-Barthélemy and Saint-Martin),,1950,5.621

Guadeloupe (including Saint-Barthélemy and Saint-Martin),,1951,5.616

Guadeloupe (including Saint-Barthélemy and Saint-Martin),,1952,5.609

Guadeloupe (including Saint-Barthélemy and Saint-Martin),,1953,5.606

Guadeloupe (including Saint-Barthélemy and Saint-Martin),,1954,5.605

Guadeloupe (including Saint-Barthélemy and Saint-Martin),,1955,5.609

Guadeloupe (including Saint-Barthélemy and Saint-Martin),,1956,5.615

Guadeloupe (including Saint-Barthélemy and Saint-Martin),,1957,5.623

Guadeloupe (including Saint-Barthélemy and Saint-Martin),,1958,5.632

Guadeloupe (including Saint-Barthélemy and Saint-Martin),,1959,5.639

Guadeloupe (including Saint-Barthélemy and Saint-Martin),,1960,5.641

Guadeloupe (including Saint-Barthélemy and Saint-Martin),,1961,5.636

Guadeloupe (including Saint-Barthélemy and Saint-Martin),,1962,5.619

Guadeloupe (including Saint-Barthélemy and Saint-Martin),,1963,5.587

Guadeloupe (including Saint-Barthélemy and Saint-Martin),,1964,5.539

Guadeloupe (including Saint-Barthélemy and Saint-Martin),,1965,5.472

Guadeloupe (including Saint-Barthélemy and Saint-Martin),,1966,5.387

Guadeloupe (including Saint-Barthélemy and Saint-Martin),,1967,5.286

Guadeloupe (including Saint-Barthélemy and Saint-Martin),,1968,5.17

Guadeloupe (including Saint-Barthélemy and Saint-Martin),,1969,5.042

Guadeloupe (including Saint-Barthélemy and Saint-Martin),,1970,4.901

Guadeloupe (including Saint-Barthélemy and Saint-Martin),,1971,4.746

Guadeloupe (including Saint-Barthélemy and Saint-Martin),,1972,4.58

Guadeloupe (including Saint-Barthélemy and Saint-Martin),,1973,4.403

Guadeloupe (including Saint-Barthélemy and Saint-Martin),,1974,4.217

Guadeloupe (including Saint-Barthélemy and Saint-Martin),,1975,4.022

Guadeloupe (including Saint-Barthélemy and Saint-Martin),,1976,3.816

Guadeloupe (including Saint-Barthélemy and Saint-Martin),,1977,3.602

Guadeloupe (including Saint-Barthélemy and Saint-Martin),,1978,3.384

Guadeloupe (including Saint-Barthélemy and Saint-Martin),,1979,3.17

Guadeloupe (including Saint-Barthélemy and Saint-Martin),,1980,2.97

Guadeloupe (including Saint-Barthélemy and Saint-Martin),,1981,2.798

Guadeloupe (including Saint-Barthélemy and Saint-Martin),,1982,2.66

Guadeloupe (including Saint-Barthélemy and Saint-Martin),,1983,2.558

Guadeloupe (including Saint-Barthélemy and Saint-Martin),,1984,2.492

Guadeloupe (including Saint-Barthélemy and Saint-Martin),,1985,2.451

Guadeloupe (including Saint-Barthélemy and Saint-Martin),,1986,2.426

Guadeloupe (including Saint-Barthélemy and Saint-Martin),,1987,2.401

Guadeloupe (including Saint-Barthélemy and Saint-Martin),,1988,2.366

Guadeloupe (including Saint-Barthélemy and Saint-Martin),,1989,2.32

Guadeloupe (including Saint-Barthélemy and Saint-Martin),,1990,2.263

Guadeloupe (including Saint-Barthélemy and Saint-Martin),,1991,2.202

Guadeloupe (including Saint-Barthélemy and Saint-Martin),,1992,2.149

Guadeloupe (including Saint-Barthélemy and Saint-Martin),,1993,2.109

Guadeloupe (including Saint-Barthélemy and Saint-Martin),,1994,2.084

Guadeloupe (including Saint-Barthélemy and Saint-Martin),,1995,2.074

Guadeloupe (including Saint-Barthélemy and Saint-Martin),,1996,2.075

Guadeloupe (including Saint-Barthélemy and Saint-Martin),,1997,2.08

Guadeloupe (including Saint-Barthélemy and Saint-Martin),,1998,2.086

Guadeloupe (including Saint-Barthélemy and Saint-Martin),,1999,2.09

Guadeloupe (including Saint-Barthélemy and Saint-Martin),,2000,2.091

Guadeloupe (including Saint-Barthélemy and Saint-Martin),,2001,2.088

Guadeloupe (including Saint-Barthélemy and Saint-Martin),,2002,2.084

Guadeloupe (including Saint-Barthélemy and Saint-Martin),,2003,2.081

Guadeloupe (including Saint-Barthélemy and Saint-Martin),,2004,2.077

Guadeloupe (including Saint-Barthélemy and Saint-Martin),,2005,2.073

Guadeloupe (including Saint-Barthélemy and Saint-Martin),,2006,2.068

Guadeloupe (including Saint-Barthélemy and Saint-Martin),,2007,2.062

Guadeloupe (including Saint-Barthélemy and Saint-Martin),,2008,2.054

Guadeloupe (including Saint-Barthélemy and Saint-Martin),,2009,2.044

Guadeloupe (including Saint-Barthélemy and Saint-Martin),,2010,2.032

Guadeloupe (including Saint-Barthélemy and Saint-Martin),,2011,2.019

Guadeloupe (including Saint-Barthélemy and Saint-Martin),,2012,2.004

Guadeloupe (including Saint-Barthélemy and Saint-Martin),,2013,1.989

Guadeloupe (including Saint-Barthélemy and Saint-Martin),,2014,1.973

Guadeloupe (including Saint-Barthélemy and Saint-Martin),,2015,1.957

Guam,GUM,1950,5.444

Guam,GUM,1951,5.458

Guam,GUM,1952,5.493

Guam,GUM,1953,5.541

Guam,GUM,1954,5.603

Guam,GUM,1955,5.678

Guam,GUM,1956,5.762

Guam,GUM,1957,5.851

Guam,GUM,1958,5.939

Guam,GUM,1959,6.011

Guam,GUM,1960,6.052

Guam,GUM,1961,6.048

Guam,GUM,1962,5.984

Guam,GUM,1963,5.857

Guam,GUM,1964,5.67

Guam,GUM,1965,5.439

Guam,GUM,1966,5.187

Guam,GUM,1967,4.939

Guam,GUM,1968,4.716

Guam,GUM,1969,4.526

Guam,GUM,1970,4.372

Guam,GUM,1971,4.248

Guam,GUM,1972,4.137

Guam,GUM,1973,4.028

Guam,GUM,1974,3.917

Guam,GUM,1975,3.801

Guam,GUM,1976,3.681

Guam,GUM,1977,3.561

Guam,GUM,1978,3.447

Guam,GUM,1979,3.34

Guam,GUM,1980,3.248

Guam,GUM,1981,3.177

Guam,GUM,1982,3.128

Guam,GUM,1983,3.1

Guam,GUM,1984,3.092

Guam,GUM,1985,3.095

Guam,GUM,1986,3.099

Guam,GUM,1987,3.097

Guam,GUM,1988,3.082

Guam,GUM,1989,3.053

Guam,GUM,1990,3.013

Guam,GUM,1991,2.969

Guam,GUM,1992,2.929

Guam,GUM,1993,2.899

Guam,GUM,1994,2.881

Guam,GUM,1995,2.872

Guam,GUM,1996,2.87

Guam,GUM,1997,2.867

Guam,GUM,1998,2.86

Guam,GUM,1999,2.846

Guam,GUM,2000,2.824

Guam,GUM,2001,2.794

Guam,GUM,2002,2.759

Guam,GUM,2003,2.72

Guam,GUM,2004,2.679

Guam,GUM,2005,2.638

Guam,GUM,2006,2.599

Guam,GUM,2007,2.562

Guam,GUM,2008,2.528

Guam,GUM,2009,2.498

Guam,GUM,2010,2.472

Guam,GUM,2011,2.448

Guam,GUM,2012,2.427

Guam,GUM,2013,2.406

Guam,GUM,2014,2.386

Guam,GUM,2015,2.367

Guatemala,GTM,1950,7.147

Guatemala,GTM,1951,7.143

Guatemala,GTM,1952,7.131

Guatemala,GTM,1953,7.115

Guatemala,GTM,1954,7.094

Guatemala,GTM,1955,7.069

Guatemala,GTM,1956,7.04

Guatemala,GTM,1957,7.007

Guatemala,GTM,1958,6.971

Guatemala,GTM,1959,6.934

Guatemala,GTM,1960,6.896

Guatemala,GTM,1961,6.859

Guatemala,GTM,1962,6.824

Guatemala,GTM,1963,6.794

Guatemala,GTM,1964,6.767

Guatemala,GTM,1965,6.744

Guatemala,GTM,1966,6.724

Guatemala,GTM,1967,6.705

Guatemala,GTM,1968,6.685

Guatemala,GTM,1969,6.664

Guatemala,GTM,1970,6.642

Guatemala,GTM,1971,6.62

Guatemala,GTM,1972,6.599

Guatemala,GTM,1973,6.578

Guatemala,GTM,1974,6.558

Guatemala,GTM,1975,6.535

Guatemala,GTM,1976,6.51

Guatemala,GTM,1977,6.48

Guatemala,GTM,1978,6.443

Guatemala,GTM,1979,6.399

Guatemala,GTM,1980,6.342

Guatemala,GTM,1981,6.271

Guatemala,GTM,1982,6.185

Guatemala,GTM,1983,6.086

Guatemala,GTM,1984,5.977

Guatemala,GTM,1985,5.865

Guatemala,GTM,1986,5.757

Guatemala,GTM,1987,5.658

Guatemala,GTM,1988,5.572

Guatemala,GTM,1989,5.5

Guatemala,GTM,1990,5.437

Guatemala,GTM,1991,5.38

Guatemala,GTM,1992,5.32

Guatemala,GTM,1993,5.252

Guatemala,GTM,1994,5.174

Guatemala,GTM,1995,5.087

Guatemala,GTM,1996,4.993

Guatemala,GTM,1997,4.895

Guatemala,GTM,1998,4.797

Guatemala,GTM,1999,4.699

Guatemala,GTM,2000,4.598

Guatemala,GTM,2001,4.49

Guatemala,GTM,2002,4.372

Guatemala,GTM,2003,4.245

Guatemala,GTM,2004,4.11

Guatemala,GTM,2005,3.971

Guatemala,GTM,2006,3.833

Guatemala,GTM,2007,3.701

Guatemala,GTM,2008,3.58

Guatemala,GTM,2009,3.471

Guatemala,GTM,2010,3.375

Guatemala,GTM,2011,3.292

Guatemala,GTM,2012,3.219

Guatemala,GTM,2013,3.151

Guatemala,GTM,2014,3.089

Guatemala,GTM,2015,3.029

Guinea,GIN,1950,5.973

Guinea,GIN,1951,5.979

Guinea,GIN,1952,5.993

Guinea,GIN,1953,6.006

Guinea,GIN,1954,6.021

Guinea,GIN,1955,6.036

Guinea,GIN,1956,6.052

Guinea,GIN,1957,6.068

Guinea,GIN,1958,6.084

Guinea,GIN,1959,6.1

Guinea,GIN,1960,6.114

Guinea,GIN,1961,6.127

Guinea,GIN,1962,6.138

Guinea,GIN,1963,6.147

Guinea,GIN,1964,6.154

Guinea,GIN,1965,6.16

Guinea,GIN,1966,6.168

Guinea,GIN,1967,6.177

Guinea,GIN,1968,6.189

Guinea,GIN,1969,6.205

Guinea,GIN,1970,6.225

Guinea,GIN,1971,6.249

Guinea,GIN,1972,6.277

Guinea,GIN,1973,6.306

Guinea,GIN,1974,6.337

Guinea,GIN,1975,6.369

Guinea,GIN,1976,6.402

Guinea,GIN,1977,6.436

Guinea,GIN,1978,6.468

Guinea,GIN,1979,6.5

Guinea,GIN,1980,6.529

Guinea,GIN,1981,6.557

Guinea,GIN,1982,6.581

Guinea,GIN,1983,6.602

Guinea,GIN,1984,6.619

Guinea,GIN,1985,6.631

Guinea,GIN,1986,6.637

Guinea,GIN,1987,6.637

Guinea,GIN,1988,6.631

Guinea,GIN,1989,6.618

Guinea,GIN,1990,6.598

Guinea,GIN,1991,6.57

Guinea,GIN,1992,6.535

Guinea,GIN,1993,6.493

Guinea,GIN,1994,6.444

Guinea,GIN,1995,6.391

Guinea,GIN,1996,6.334

Guinea,GIN,1997,6.273

Guinea,GIN,1998,6.211

Guinea,GIN,1999,6.147

Guinea,GIN,2000,6.082

Guinea,GIN,2001,6.015

Guinea,GIN,2002,5.947

Guinea,GIN,2003,5.877

Guinea,GIN,2004,5.804

Guinea,GIN,2005,5.729

Guinea,GIN,2006,5.653

Guinea,GIN,2007,5.575

Guinea,GIN,2008,5.496

Guinea,GIN,2009,5.417

Guinea,GIN,2010,5.336

Guinea,GIN,2011,5.256

Guinea,GIN,2012,5.175

Guinea,GIN,2013,5.094

Guinea,GIN,2014,5.014

Guinea,GIN,2015,4.934

Guinea-Bissau,GNB,1950,5.914

Guinea-Bissau,GNB,1951,5.909

Guinea-Bissau,GNB,1952,5.901

Guinea-Bissau,GNB,1953,5.895

Guinea-Bissau,GNB,1954,5.892

Guinea-Bissau,GNB,1955,5.892

Guinea-Bissau,GNB,1956,5.893

Guinea-Bissau,GNB,1957,5.898

Guinea-Bissau,GNB,1958,5.904

Guinea-Bissau,GNB,1959,5.912

Guinea-Bissau,GNB,1960,5.921

Guinea-Bissau,GNB,1961,5.931

Guinea-Bissau,GNB,1962,5.941

Guinea-Bissau,GNB,1963,5.951

Guinea-Bissau,GNB,1964,5.961

Guinea-Bissau,GNB,1965,5.971

Guinea-Bissau,GNB,1966,5.982

Guinea-Bissau,GNB,1967,5.994

Guinea-Bissau,GNB,1968,6.008

Guinea-Bissau,GNB,1969,6.024

Guinea-Bissau,GNB,1970,6.041

Guinea-Bissau,GNB,1971,6.06

Guinea-Bissau,GNB,1972,6.078

Guinea-Bissau,GNB,1973,6.098

Guinea-Bissau,GNB,1974,6.12

Guinea-Bissau,GNB,1975,6.15

Guinea-Bissau,GNB,1976,6.192

Guinea-Bissau,GNB,1977,6.249

Guinea-Bissau,GNB,1978,6.32

Guinea-Bissau,GNB,1979,6.401

Guinea-Bissau,GNB,1980,6.487

Guinea-Bissau,GNB,1981,6.569

Guinea-Bissau,GNB,1982,6.64

Guinea-Bissau,GNB,1983,6.694

Guinea-Bissau,GNB,1984,6.727

Guinea-Bissau,GNB,1985,6.739

Guinea-Bissau,GNB,1986,6.734

Guinea-Bissau,GNB,1987,6.717

Guinea-Bissau,GNB,1988,6.693

Guinea-Bissau,GNB,1989,6.663

Guinea-Bissau,GNB,1990,6.627

Guinea-Bissau,GNB,1991,6.582

Guinea-Bissau,GNB,1992,6.526

Guinea-Bissau,GNB,1993,6.458

Guinea-Bissau,GNB,1994,6.381

Guinea-Bissau,GNB,1995,6.294

Guinea-Bissau,GNB,1996,6.201

Guinea-Bissau,GNB,1997,6.105

Guinea-Bissau,GNB,1998,6.009

Guinea-Bissau,GNB,1999,5.913

Guinea-Bissau,GNB,2000,5.82

Guinea-Bissau,GNB,2001,5.73

Guinea-Bissau,GNB,2002,5.641

Guinea-Bissau,GNB,2003,5.555

Guinea-Bissau,GNB,2004,5.47

Guinea-Bissau,GNB,2005,5.389

Guinea-Bissau,GNB,2006,5.312

Guinea-Bissau,GNB,2007,5.241

Guinea-Bissau,GNB,2008,5.174

Guinea-Bissau,GNB,2009,5.11

Guinea-Bissau,GNB,2010,5.049

Guinea-Bissau,GNB,2011,4.987

Guinea-Bissau,GNB,2012,4.923

Guinea-Bissau,GNB,2013,4.855

Guinea-Bissau,GNB,2014,4.784

Guinea-Bissau,GNB,2015,4.71

Guyana,GUY,1950,5.689

Guyana,GUY,1951,5.794

Guyana,GUY,1952,5.988

Guyana,GUY,1953,6.147

Guyana,GUY,1954,6.273

Guyana,GUY,1955,6.365

Guyana,GUY,1956,6.425

Guyana,GUY,1957,6.452

Guyana,GUY,1958,6.45

Guyana,GUY,1959,6.422

Guyana,GUY,1960,6.372

Guyana,GUY,1961,6.305

Guyana,GUY,1962,6.226

Guyana,GUY,1963,6.14

Guyana,GUY,1964,6.05

Guyana,GUY,1965,5.957

Guyana,GUY,1966,5.86

Guyana,GUY,1967,5.756

Guyana,GUY,1968,5.642

Guyana,GUY,1969,5.517

Guyana,GUY,1970,5.38

Guyana,GUY,1971,5.226

Guyana,GUY,1972,5.057

Guyana,GUY,1973,4.877

Guyana,GUY,1974,4.689

Guyana,GUY,1975,4.503

Guyana,GUY,1976,4.327

Guyana,GUY,1977,4.169

Guyana,GUY,1978,4.031

Guyana,GUY,1979,3.915

Guyana,GUY,1980,3.817

Guyana,GUY,1981,3.728

Guyana,GUY,1982,3.64

Guyana,GUY,1983,3.548

Guyana,GUY,1984,3.452

Guyana,GUY,1985,3.354

Guyana,GUY,1986,3.263

Guyana,GUY,1987,3.186

Guyana,GUY,1988,3.127

Guyana,GUY,1989,3.089

Guyana,GUY,1990,3.069

Guyana,GUY,1991,3.063

Guyana,GUY,1992,3.066

Guyana,GUY,1993,3.071

Guyana,GUY,1994,3.074

Guyana,GUY,1995,3.074

Guyana,GUY,1996,3.069

Guyana,GUY,1997,3.061

Guyana,GUY,1998,3.05

Guyana,GUY,1999,3.036

Guyana,GUY,2000,3.017

Guyana,GUY,2001,2.992

Guyana,GUY,2002,2.961

Guyana,GUY,2003,2.924

Guyana,GUY,2004,2.883

Guyana,GUY,2005,2.84

Guyana,GUY,2006,2.797

Guyana,GUY,2007,2.755

Guyana,GUY,2008,2.718

Guyana,GUY,2009,2.684

Guyana,GUY,2010,2.655

Guyana,GUY,2011,2.63

Guyana,GUY,2012,2.606

Guyana,GUY,2013,2.582

Guyana,GUY,2014,2.559

Guyana,GUY,2015,2.534

Haiti,HTI,1950,6.31

Haiti,HTI,1951,6.306

Haiti,HTI,1952,6.299

Haiti,HTI,1953,6.296

Haiti,HTI,1954,6.296

Haiti,HTI,1955,6.298

Haiti,HTI,1956,6.303

Haiti,HTI,1957,6.31

Haiti,HTI,1958,6.317

Haiti,HTI,1959,6.322

Haiti,HTI,1960,6.324

Haiti,HTI,1961,6.318

Haiti,HTI,1962,6.302

Haiti,HTI,1963,6.274

Haiti,HTI,1964,6.233

Haiti,HTI,1965,6.179

Haiti,HTI,1966,6.109

Haiti,HTI,1967,6.026

Haiti,HTI,1968,5.937

Haiti,HTI,1969,5.846

Haiti,HTI,1970,5.762

Haiti,HTI,1971,5.692

Haiti,HTI,1972,5.642

Haiti,HTI,1973,5.616

Haiti,HTI,1974,5.614

Haiti,HTI,1975,5.641

Haiti,HTI,1976,5.698

Haiti,HTI,1977,5.779

Haiti,HTI,1978,5.872

Haiti,HTI,1979,5.97

Haiti,HTI,1980,6.058

Haiti,HTI,1981,6.122

Haiti,HTI,1982,6.154

Haiti,HTI,1983,6.149

Haiti,HTI,1984,6.106

Haiti,HTI,1985,6.028

Haiti,HTI,1986,5.921

Haiti,HTI,1987,5.799

Haiti,HTI,1988,5.673

Haiti,HTI,1989,5.548

Haiti,HTI,1990,5.43

Haiti,HTI,1991,5.318

Haiti,HTI,1992,5.21

Haiti,HTI,1993,5.103

Haiti,HTI,1994,4.997

Haiti,HTI,1995,4.89

Haiti,HTI,1996,4.779

Haiti,HTI,1997,4.665

Haiti,HTI,1998,4.547

Haiti,HTI,1999,4.425

Haiti,HTI,2000,4.302

Haiti,HTI,2001,4.182

Haiti,HTI,2002,4.065

Haiti,HTI,2003,3.955

Haiti,HTI,2004,3.853

Haiti,HTI,2005,3.757

Haiti,HTI,2006,3.667

Haiti,HTI,2007,3.579

Haiti,HTI,2008,3.493

Haiti,HTI,2009,3.408

Haiti,HTI,2010,3.325

Haiti,HTI,2011,3.245

Haiti,HTI,2012,3.169

Haiti,HTI,2013,3.098

Haiti,HTI,2014,3.033

Haiti,HTI,2015,2.973

High-income countries,,1950,2.948

High-income countries,,1951,2.957

High-income countries,,1952,2.975

High-income countries,,1953,2.99

High-income countries,,1954,3.002

High-income countries,,1955,3.012

High-income countries,,1956,3.019

High-income countries,,1957,3.022

High-income countries,,1958,3.021

High-income countries,,1959,3.015

High-income countries,,1960,3.003

High-income countries,,1961,2.982

High-income countries,,1962,2.952

High-income countries,,1963,2.913

High-income countries,,1964,2.865

High-income countries,,1965,2.809

High-income countries,,1966,2.747

High-income countries,,1967,2.681

High-income countries,,1968,2.614

High-income countries,,1969,2.546

High-income countries,,1970,2.479

High-income countries,,1971,2.412

High-income countries,,1972,2.345

High-income countries,,1973,2.279

High-income countries,,1974,2.213

High-income countries,,1975,2.151

High-income countries,,1976,2.094

High-income countries,,1977,2.045

High-income countries,,1978,2.002

High-income countries,,1979,1.968

High-income countries,,1980,1.94

High-income countries,,1981,1.918

High-income countries,,1982,1.9

High-income countries,,1983,1.883

High-income countries,,1984,1.866

High-income countries,,1985,1.851

High-income countries,,1986,1.836

High-income countries,,1987,1.823

High-income countries,,1988,1.813

High-income countries,,1989,1.804

High-income countries,,1990,1.795

High-income countries,,1991,1.786

High-income countries,,1992,1.776

High-income countries,,1993,1.764

High-income countries,,1994,1.75

High-income countries,,1995,1.735

High-income countries,,1996,1.719

High-income countries,,1997,1.705

High-income countries,,1998,1.692

High-income countries,,1999,1.682

High-income countries,,2000,1.676

High-income countries,,2001,1.675

High-income countries,,2002,1.678

High-income countries,,2003,1.685

High-income countries,,2004,1.694

High-income countries,,2005,1.705

High-income countries,,2006,1.714

High-income countries,,2007,1.72

High-income countries,,2008,1.722

High-income countries,,2009,1.72

High-income countries,,2010,1.716

High-income countries,,2011,1.709

High-income countries,,2012,1.704

High-income countries,,2013,1.7

High-income countries,,2014,1.699

High-income countries,,2015,1.701

Honduras,HND,1950,7.469

Honduras,HND,1951,7.479

Honduras,HND,1952,7.496

Honduras,HND,1953,7.507

Honduras,HND,1954,7.512

Honduras,HND,1955,7.513

Honduras,HND,1956,7.508

Honduras,HND,1957,7.498

Honduras,HND,1958,7.485

Honduras,HND,1959,7.471

Honduras,HND,1960,7.458

Honduras,HND,1961,7.448

Honduras,HND,1962,7.443

Honduras,HND,1963,7.441

Honduras,HND,1964,7.443

Honduras,HND,1965,7.442

Honduras,HND,1966,7.435

Honduras,HND,1967,7.417

Honduras,HND,1968,7.383

Honduras,HND,1969,7.334

Honduras,HND,1970,7.27

Honduras,HND,1971,7.195

Honduras,HND,1972,7.111

Honduras,HND,1973,7.025

Honduras,HND,1974,6.935

Honduras,HND,1975,6.844

Honduras,HND,1976,6.749

Honduras,HND,1977,6.649

Honduras,HND,1978,6.543

Honduras,HND,1979,6.431

Honduras,HND,1980,6.313

Honduras,HND,1981,6.19

Honduras,HND,1982,6.062

Honduras,HND,1983,5.932

Honduras,HND,1984,5.802

Honduras,HND,1985,5.674

Honduras,HND,1986,5.553

Honduras,HND,1987,5.439

Honduras,HND,1988,5.333

Honduras,HND,1989,5.233

Honduras,HND,1990,5.139

Honduras,HND,1991,5.046

Honduras,HND,1992,4.952

Honduras,HND,1993,4.853

Honduras,HND,1994,4.749

Honduras,HND,1995,4.64

Honduras,HND,1996,4.525

Honduras,HND,1997,4.408

Honduras,HND,1998,4.291

Honduras,HND,1999,4.173

Honduras,HND,2000,4.055

Honduras,HND,2001,3.936

Honduras,HND,2002,3.815

Honduras,HND,2003,3.693

Honduras,HND,2004,3.57

Honduras,HND,2005,3.447

Honduras,HND,2006,3.325

Honduras,HND,2007,3.204

Honduras,HND,2008,3.087

Honduras,HND,2009,2.975

Honduras,HND,2010,2.871

Honduras,HND,2011,2.777

Honduras,HND,2012,2.694

Honduras,HND,2013,2.621

Honduras,HND,2014,2.559

Honduras,HND,2015,2.507

Hong Kong,HKG,1950,4.414

Hong Kong,HKG,1951,4.409

Hong Kong,HKG,1952,4.41

Hong Kong,HKG,1953,4.435

Hong Kong,HKG,1954,4.483

Hong Kong,HKG,1955,4.553

Hong Kong,HKG,1956,4.643

Hong Kong,HKG,1957,4.746

Hong Kong,HKG,1958,4.853

Hong Kong,HKG,1959,4.949

Hong Kong,HKG,1960,5.014

Hong Kong,HKG,1961,5.03

Hong Kong,HKG,1962,4.98

Hong Kong,HKG,1963,4.857

Hong Kong,HKG,1964,4.665

Hong Kong,HKG,1965,4.422

Hong Kong,HKG,1966,4.152

Hong Kong,HKG,1967,3.887

Hong Kong,HKG,1968,3.649

Hong Kong,HKG,1969,3.448

Hong Kong,HKG,1970,3.284

Hong Kong,HKG,1971,3.149

Hong Kong,HKG,1972,3.023

Hong Kong,HKG,1973,2.892

Hong Kong,HKG,1974,2.754

Hong Kong,HKG,1975,2.608

Hong Kong,HKG,1976,2.458

Hong Kong,HKG,1977,2.312

Hong Kong,HKG,1978,2.176

Hong Kong,HKG,1979,2.052

Hong Kong,HKG,1980,1.94

Hong Kong,HKG,1981,1.838

Hong Kong,HKG,1982,1.745

Hong Kong,HKG,1983,1.657

Hong Kong,HKG,1984,1.576

Hong Kong,HKG,1985,1.503

Hong Kong,HKG,1986,1.44

Hong Kong,HKG,1987,1.388

Hong Kong,HKG,1988,1.349

Hong Kong,HKG,1989,1.319

Hong Kong,HKG,1990,1.295

Hong Kong,HKG,1991,1.274

Hong Kong,HKG,1992,1.252

Hong Kong,HKG,1993,1.226

Hong Kong,HKG,1994,1.195

Hong Kong,HKG,1995,1.16

Hong Kong,HKG,1996,1.12

Hong Kong,HKG,1997,1.08

Hong Kong,HKG,1998,1.042

Hong Kong,HKG,1999,1.009

Hong Kong,HKG,2000,0.983

Hong Kong,HKG,2001,0.965

Hong Kong,HKG,2002,0.955

Hong Kong,HKG,2003,0.952

Hong Kong,HKG,2004,0.958

Hong Kong,HKG,2005,0.972

Hong Kong,HKG,2006,0.992

Hong Kong,HKG,2007,1.019

Hong Kong,HKG,2008,1.05

Hong Kong,HKG,2009,1.083

Hong Kong,HKG,2010,1.117

Hong Kong,HKG,2011,1.151

Hong Kong,HKG,2012,1.184

Hong Kong,HKG,2013,1.215

Hong Kong,HKG,2014,1.244

Hong Kong,HKG,2015,1.271

Hungary,HUN,1950,2.77

Hungary,HUN,1951,2.756

Hungary,HUN,1952,2.721

Hungary,HUN,1953,2.672

Hungary,HUN,1954,2.609

Hungary,HUN,1955,2.531

Hungary,HUN,1956,2.44

Hungary,HUN,1957,2.339

Hungary,HUN,1958,2.231

Hungary,HUN,1959,2.123

Hungary,HUN,1960,2.023

Hungary,HUN,1961,1.938

Hungary,HUN,1962,1.875

Hungary,HUN,1963,1.84

Hungary,HUN,1964,1.833

Hungary,HUN,1965,1.85

Hungary,HUN,1966,1.882

Hungary,HUN,1967,1.92

Hungary,HUN,1968,1.954

Hungary,HUN,1969,1.982

Hungary,HUN,1970,2.006

Hungary,HUN,1971,2.031

Hungary,HUN,1972,2.062

Hungary,HUN,1973,2.101

Hungary,HUN,1974,2.145

Hungary,HUN,1975,2.184

Hungary,HUN,1976,2.206

Hungary,HUN,1977,2.203

Hungary,HUN,1978,2.173

Hungary,HUN,1979,2.117

Hungary,HUN,1980,2.043

Hungary,HUN,1981,1.964

Hungary,HUN,1982,1.892

Hungary,HUN,1983,1.837

Hungary,HUN,1984,1.803

Hungary,HUN,1985,1.79

Hungary,HUN,1986,1.794

Hungary,HUN,1987,1.805

Hungary,HUN,1988,1.814

Hungary,HUN,1989,1.817

Hungary,HUN,1990,1.808

Hungary,HUN,1991,1.783

Hungary,HUN,1992,1.743

Hungary,HUN,1993,1.691

Hungary,HUN,1994,1.628

Hungary,HUN,1995,1.559

Hungary,HUN,1996,1.491

Hungary,HUN,1997,1.427

Hungary,HUN,1998,1.374

Hungary,HUN,1999,1.334

Hungary,HUN,2000,1.307

Hungary,HUN,2001,1.294

Hungary,HUN,2002,1.29

Hungary,HUN,2003,1.292

Hungary,HUN,2004,1.297

Hungary,HUN,2005,1.304

Hungary,HUN,2006,1.311

Hungary,HUN,2007,1.317

Hungary,HUN,2008,1.322

Hungary,HUN,2009,1.325

Hungary,HUN,2010,1.328

Hungary,HUN,2011,1.33

Hungary,HUN,2012,1.334

Hungary,HUN,2013,1.34

Hungary,HUN,2014,1.349

Hungary,HUN,2015,1.361

Iceland,ISL,1950,3.621

Iceland,ISL,1951,3.691

Iceland,ISL,1952,3.822

Iceland,ISL,1953,3.933

Iceland,ISL,1954,4.024

Iceland,ISL,1955,4.095

Iceland,ISL,1956,4.146

Iceland,ISL,1957,4.176

Iceland,ISL,1958,4.185

Iceland,ISL,1959,4.17

Iceland,ISL,1960,4.131

Iceland,ISL,1961,4.066

Iceland,ISL,1962,3.976

Iceland,ISL,1963,3.864

Iceland,ISL,1964,3.736

Iceland,ISL,1965,3.599

Iceland,ISL,1966,3.463

Iceland,ISL,1967,3.335

Iceland,ISL,1968,3.218

Iceland,ISL,1969,3.118

Iceland,ISL,1970,3.031

Iceland,ISL,1971,2.954

Iceland,ISL,1972,2.88

Iceland,ISL,1973,2.804

Iceland,ISL,1974,2.726

Iceland,ISL,1975,2.645

Iceland,ISL,1976,2.565

Iceland,ISL,1977,2.489

Iceland,ISL,1978,2.422

Iceland,ISL,1979,2.362

Iceland,ISL,1980,2.312

Iceland,ISL,1981,2.269

Iceland,ISL,1982,2.232

Iceland,ISL,1983,2.199

Iceland,ISL,1984,2.172

Iceland,ISL,1985,2.15

Iceland,ISL,1986,2.136

Iceland,ISL,1987,2.131

Iceland,ISL,1988,2.135

Iceland,ISL,1989,2.145

Iceland,ISL,1990,2.157

Iceland,ISL,1991,2.167

Iceland,ISL,1992,2.171

Iceland,ISL,1993,2.167

Iceland,ISL,1994,2.154

Iceland,ISL,1995,2.132

Iceland,ISL,1996,2.104

Iceland,ISL,1997,2.074

Iceland,ISL,1998,2.046

Iceland,ISL,1999,2.022

Iceland,ISL,2000,2.005

Iceland,ISL,2001,2

Iceland,ISL,2002,2.006

Iceland,ISL,2003,2.021

Iceland,ISL,2004,2.044

Iceland,ISL,2005,2.068

Iceland,ISL,2006,2.089

Iceland,ISL,2007,2.102

Iceland,ISL,2008,2.103

Iceland,ISL,2009,2.093

Iceland,ISL,2010,2.072

Iceland,ISL,2011,2.044

Iceland,ISL,2012,2.014

Iceland,ISL,2013,1.986

Iceland,ISL,2014,1.963

Iceland,ISL,2015,1.946

India,IND,1950,5.91

India,IND,1951,5.908

India,IND,1952,5.903

India,IND,1953,5.9

India,IND,1954,5.899

India,IND,1955,5.899

India,IND,1956,5.9

India,IND,1957,5.902

India,IND,1958,5.904

India,IND,1959,5.906

India,IND,1960,5.906

India,IND,1961,5.902

India,IND,1962,5.894

India,IND,1963,5.88

India,IND,1964,5.859

India,IND,1965,5.83

India,IND,1966,5.794

India,IND,1967,5.751

India,IND,1968,5.702

India,IND,1969,5.648

India,IND,1970,5.587

India,IND,1971,5.519

India,IND,1972,5.444

India,IND,1973,5.363

India,IND,1974,5.278

India,IND,1975,5.192

India,IND,1976,5.108

India,IND,1977,5.029

India,IND,1978,4.956

India,IND,1979,4.889

India,IND,1980,4.827

India,IND,1981,4.766

India,IND,1982,4.703

India,IND,1983,4.636

India,IND,1984,4.563

India,IND,1985,4.484

India,IND,1986,4.399

India,IND,1987,4.311

India,IND,1988,4.222

India,IND,1989,4.132

India,IND,1990,4.045

India,IND,1991,3.959

India,IND,1992,3.877

India,IND,1993,3.799

India,IND,1994,3.723

India,IND,1995,3.651

India,IND,1996,3.582

India,IND,1997,3.514

India,IND,1998,3.446

India,IND,1999,3.379

India,IND,2000,3.311

India,IND,2001,3.243

India,IND,2002,3.175

India,IND,2003,3.107

India,IND,2004,3.038

India,IND,2005,2.969

India,IND,2006,2.897

India,IND,2007,2.823

India,IND,2008,2.748

India,IND,2009,2.673

India,IND,2010,2.601

India,IND,2011,2.534

India,IND,2012,2.475

India,IND,2013,2.425

India,IND,2014,2.384

India,IND,2015,2.352

Indonesia,IDN,1950,5.346

Indonesia,IDN,1951,5.387

Indonesia,IDN,1952,5.463

Indonesia,IDN,1953,5.527

Indonesia,IDN,1954,5.579

Indonesia,IDN,1955,5.619

Indonesia,IDN,1956,5.647

Indonesia,IDN,1957,5.665

Indonesia,IDN,1958,5.672

Indonesia,IDN,1959,5.672

Indonesia,IDN,1960,5.666

Indonesia,IDN,1961,5.656

Indonesia,IDN,1962,5.645

Indonesia,IDN,1963,5.634

Indonesia,IDN,1964,5.623

Indonesia,IDN,1965,5.612

Indonesia,IDN,1966,5.599

Indonesia,IDN,1967,5.582

Indonesia,IDN,1968,5.556

Indonesia,IDN,1969,5.521

Indonesia,IDN,1970,5.474

Indonesia,IDN,1971,5.413

Indonesia,IDN,1972,5.338

Indonesia,IDN,1973,5.251

Indonesia,IDN,1974,5.152

Indonesia,IDN,1975,5.043

Indonesia,IDN,1976,4.927

Indonesia,IDN,1977,4.806

Indonesia,IDN,1978,4.682

Indonesia,IDN,1979,4.557

Indonesia,IDN,1980,4.43

Indonesia,IDN,1981,4.301

Indonesia,IDN,1982,4.167

Indonesia,IDN,1983,4.028

Indonesia,IDN,1984,3.887

Indonesia,IDN,1985,3.745

Indonesia,IDN,1986,3.606

Indonesia,IDN,1987,3.472

Indonesia,IDN,1988,3.346

Indonesia,IDN,1989,3.229

Indonesia,IDN,1990,3.122

Indonesia,IDN,1991,3.022

Indonesia,IDN,1992,2.929

Indonesia,IDN,1993,2.842

Indonesia,IDN,1994,2.761

Indonesia,IDN,1995,2.688

Indonesia,IDN,1996,2.627

Indonesia,IDN,1997,2.579

Indonesia,IDN,1998,2.544

Indonesia,IDN,1999,2.523

Indonesia,IDN,2000,2.512

Indonesia,IDN,2001,2.509

Indonesia,IDN,2002,2.511

Indonesia,IDN,2003,2.513

Indonesia,IDN,2004,2.515

Indonesia,IDN,2005,2.514

Indonesia,IDN,2006,2.51

Indonesia,IDN,2007,2.505

Indonesia,IDN,2008,2.499

Indonesia,IDN,2009,2.492

Indonesia,IDN,2010,2.483

Indonesia,IDN,2011,2.471

Indonesia,IDN,2012,2.455

Indonesia,IDN,2013,2.436

Indonesia,IDN,2014,2.414

Indonesia,IDN,2015,2.389

Iran,IRN,1950,6.915

Iran,IRN,1951,6.912

Iran,IRN,1952,6.908

Iran,IRN,1953,6.906

Iran,IRN,1954,6.906

Iran,IRN,1955,6.908

Iran,IRN,1956,6.911

Iran,IRN,1957,6.916

Iran,IRN,1958,6.921

Iran,IRN,1959,6.925

Iran,IRN,1960,6.927

Iran,IRN,1961,6.924

Iran,IRN,1962,6.914

Iran,IRN,1963,6.896

Iran,IRN,1964,6.868

Iran,IRN,1965,6.827

Iran,IRN,1966,6.77

Iran,IRN,1967,6.699

Iran,IRN,1968,6.617

Iran,IRN,1969,6.528

Iran,IRN,1970,6.44

Iran,IRN,1971,6.359

Iran,IRN,1972,6.292

Iran,IRN,1973,6.243

Iran,IRN,1974,6.214

Iran,IRN,1975,6.212

Iran,IRN,1976,6.238

Iran,IRN,1977,6.289

Iran,IRN,1978,6.354

Iran,IRN,1979,6.422

Iran,IRN,1980,6.481

Iran,IRN,1981,6.516

Iran,IRN,1982,6.515

Iran,IRN,1983,6.47

Iran,IRN,1984,6.374

Iran,IRN,1985,6.223

Iran,IRN,1986,6.014

Iran,IRN,1987,5.758

Iran,IRN,1988,5.467

Iran,IRN,1989,5.15

Iran,IRN,1990,4.818

Iran,IRN,1991,4.478

Iran,IRN,1992,4.139

Iran,IRN,1993,3.81

Iran,IRN,1994,3.5

Iran,IRN,1995,3.216

Iran,IRN,1996,2.961

Iran,IRN,1997,2.734

Iran,IRN,1998,2.533

Iran,IRN,1999,2.358

Iran,IRN,2000,2.211

Iran,IRN,2001,2.092

Iran,IRN,2002,1.997

Iran,IRN,2003,1.925

Iran,IRN,2004,1.87

Iran,IRN,2005,1.831

Iran,IRN,2006,1.805

Iran,IRN,2007,1.788

Iran,IRN,2008,1.778

Iran,IRN,2009,1.772

Iran,IRN,2010,1.765

Iran,IRN,2011,1.755

Iran,IRN,2012,1.742

Iran,IRN,2013,1.727

Iran,IRN,2014,1.708

Iran,IRN,2015,1.686

Iraq,IRQ,1950,8.11

Iraq,IRQ,1951,7.875

Iraq,IRQ,1952,7.437

Iraq,IRQ,1953,7.065

Iraq,IRQ,1954,6.758

Iraq,IRQ,1955,6.516

Iraq,IRQ,1956,6.34

Iraq,IRQ,1957,6.228

Iraq,IRQ,1958,6.178

Iraq,IRQ,1959,6.188

Iraq,IRQ,1960,6.252

Iraq,IRQ,1961,6.364

Iraq,IRQ,1962,6.514

Iraq,IRQ,1963,6.689

Iraq,IRQ,1964,6.872

Iraq,IRQ,1965,7.044

Iraq,IRQ,1966,7.188

Iraq,IRQ,1967,7.295

Iraq,IRQ,1968,7.362

Iraq,IRQ,1969,7.383

Iraq,IRQ,1970,7.362

Iraq,IRQ,1971,7.308

Iraq,IRQ,1972,7.234

Iraq,IRQ,1973,7.153

Iraq,IRQ,1974,7.072

Iraq,IRQ,1975,6.993

Iraq,IRQ,1976,6.915

Iraq,IRQ,1977,6.834

Iraq,IRQ,1978,6.748

Iraq,IRQ,1979,6.658

Iraq,IRQ,1980,6.569

Iraq,IRQ,1981,6.485

Iraq,IRQ,1982,6.407

Iraq,IRQ,1983,6.338

Iraq,IRQ,1984,6.276

Iraq,IRQ,1985,6.22

Iraq,IRQ,1986,6.164

Iraq,IRQ,1987,6.105

Iraq,IRQ,1988,6.039

Iraq,IRQ,1989,5.965

Iraq,IRQ,1990,5.882

Iraq,IRQ,1991,5.794

Iraq,IRQ,1992,5.702

Iraq,IRQ,1993,5.61

Iraq,IRQ,1994,5.517

Iraq,IRQ,1995,5.423

Iraq,IRQ,1996,5.325

Iraq,IRQ,1997,5.221

Iraq,IRQ,1998,5.112

Iraq,IRQ,1999,5.002

Iraq,IRQ,2000,4.894

Iraq,IRQ,2001,4.796

Iraq,IRQ,2002,4.711

Iraq,IRQ,2003,4.644

Iraq,IRQ,2004,4.594

Iraq,IRQ,2005,4.563

Iraq,IRQ,2006,4.549

Iraq,IRQ,2007,4.547

Iraq,IRQ,2008,4.552

Iraq,IRQ,2009,4.559

Iraq,IRQ,2010,4.562

Iraq,IRQ,2011,4.557

Iraq,IRQ,2012,4.542

Iraq,IRQ,2013,4.515

Iraq,IRQ,2014,4.476

Iraq,IRQ,2015,4.427

Ireland,IRL,1950,3.475

Ireland,IRL,1951,3.449

Ireland,IRL,1952,3.409

Ireland,IRL,1953,3.394

Ireland,IRL,1954,3.404

Ireland,IRL,1955,3.439

Ireland,IRL,1956,3.496

Ireland,IRL,1957,3.574

Ireland,IRL,1958,3.666

Ireland,IRL,1959,3.764

Ireland,IRL,1960,3.857

Ireland,IRL,1961,3.933

Ireland,IRL,1962,3.984

Ireland,IRL,1963,4.002

Ireland,IRL,1964,3.987

Ireland,IRL,1965,3.949

Ireland,IRL,1966,3.902

Ireland,IRL,1967,3.859

Ireland,IRL,1968,3.83

Ireland,IRL,1969,3.817

Ireland,IRL,1970,3.813

Ireland,IRL,1971,3.807

Ireland,IRL,1972,3.785

Ireland,IRL,1973,3.739

Ireland,IRL,1974,3.667

Ireland,IRL,1975,3.571

Ireland,IRL,1976,3.458

Ireland,IRL,1977,3.338

Ireland,IRL,1978,3.221

Ireland,IRL,1979,3.109

Ireland,IRL,1980,3.001

Ireland,IRL,1981,2.897

Ireland,IRL,1982,2.79

Ireland,IRL,1983,2.678

Ireland,IRL,1984,2.564

Ireland,IRL,1985,2.45

Ireland,IRL,1986,2.338

Ireland,IRL,1987,2.234

Ireland,IRL,1988,2.14

Ireland,IRL,1989,2.06

Ireland,IRL,1990,1.996

Ireland,IRL,1991,1.949

Ireland,IRL,1992,1.918

Ireland,IRL,1993,1.901

Ireland,IRL,1994,1.895

Ireland,IRL,1995,1.898

Ireland,IRL,1996,1.908

Ireland,IRL,1997,1.92

Ireland,IRL,1998,1.932

Ireland,IRL,1999,1.943

Ireland,IRL,2000,1.952

Ireland,IRL,2001,1.958

Ireland,IRL,2002,1.964

Ireland,IRL,2003,1.97

Ireland,IRL,2004,1.977

Ireland,IRL,2005,1.983

Ireland,IRL,2006,1.989

Ireland,IRL,2007,1.994

Ireland,IRL,2008,1.997

Ireland,IRL,2009,1.999

Ireland,IRL,2010,2

Ireland,IRL,2011,1.999

Ireland,IRL,2012,1.998

Ireland,IRL,2013,1.995

Ireland,IRL,2014,1.992

Ireland,IRL,2015,1.989

Israel,ISR,1950,4.523

Israel,ISR,1951,4.453

Israel,ISR,1952,4.322

Israel,ISR,1953,4.207

Israel,ISR,1954,4.109

Israel,ISR,1955,4.028

Israel,ISR,1956,3.964

Israel,ISR,1957,3.915

Israel,ISR,1958,3.88

Israel,ISR,1959,3.857

Israel,ISR,1960,3.843

Israel,ISR,1961,3.835

Israel,ISR,1962,3.83

Israel,ISR,1963,3.824

Israel,ISR,1964,3.817

Israel,ISR,1965,3.81

Israel,ISR,1966,3.804

Israel,ISR,1967,3.803

Israel,ISR,1968,3.806

Israel,ISR,1969,3.812

Israel,ISR,1970,3.817

Israel,ISR,1971,3.813

Israel,ISR,1972,3.798

Israel,ISR,1973,3.767

Israel,ISR,1974,3.722

Israel,ISR,1975,3.662

Israel,ISR,1976,3.59

Israel,ISR,1977,3.511

Israel,ISR,1978,3.43

Israel,ISR,1979,3.351

Israel,ISR,1980,3.28

Israel,ISR,1981,3.219

Israel,ISR,1982,3.17

Israel,ISR,1983,3.133

Israel,ISR,1984,3.106

Israel,ISR,1985,3.087

Israel,ISR,1986,3.072

Israel,ISR,1987,3.057

Israel,ISR,1988,3.04

Israel,ISR,1989,3.019

Israel,ISR,1990,2.996

Israel,ISR,1991,2.973

Israel,ISR,1992,2.952

Israel,ISR,1993,2.937

Israel,ISR,1994,2.928

Israel,ISR,1995,2.924

Israel,ISR,1996,2.923

Israel,ISR,1997,2.924

Israel,ISR,1998,2.924

Israel,ISR,1999,2.923

Israel,ISR,2000,2.92

Israel,ISR,2001,2.916

Israel,ISR,2002,2.911

Israel,ISR,2003,2.908

Israel,ISR,2004,2.907

Israel,ISR,2005,2.909

Israel,ISR,2006,2.917

Israel,ISR,2007,2.932

Israel,ISR,2008,2.951

Israel,ISR,2009,2.974

Israel,ISR,2010,2.996

Israel,ISR,2011,3.014

Israel,ISR,2012,3.025

Israel,ISR,2013,3.026

Israel,ISR,2014,3.018

Israel,ISR,2015,2.999

Italy,ITA,1950,2.461

Italy,ITA,1951,2.427

Italy,ITA,1952,2.366

Italy,ITA,1953,2.321

Italy,ITA,1954,2.291

Italy,ITA,1955,2.276

Italy,ITA,1956,2.276

Italy,ITA,1957,2.29

Italy,ITA,1958,2.317

Italy,ITA,1959,2.352

Italy,ITA,1960,2.392

Italy,ITA,1961,2.434

Italy,ITA,1962,2.472

Italy,ITA,1963,2.503

Italy,ITA,1964,2.523

Italy,ITA,1965,2.531

Italy,ITA,1966,2.529

Italy,ITA,1967,2.519

Italy,ITA,1968,2.501

Italy,ITA,1969,2.477

Italy,ITA,1970,2.444

Italy,ITA,1971,2.401

Italy,ITA,1972,2.346

Italy,ITA,1973,2.279

Italy,ITA,1974,2.202

Italy,ITA,1975,2.118

Italy,ITA,1976,2.029

Italy,ITA,1977,1.938

Italy,ITA,1978,1.848

Italy,ITA,1979,1.763

Italy,ITA,1980,1.685

Italy,ITA,1981,1.616

Italy,ITA,1982,1.554

Italy,ITA,1983,1.5

Italy,ITA,1984,1.454

Italy,ITA,1985,1.415

Italy,ITA,1986,1.383

Italy,ITA,1987,1.357

Italy,ITA,1988,1.335

Italy,ITA,1989,1.316

Italy,ITA,1990,1.3

Italy,ITA,1991,1.285

Italy,ITA,1992,1.27

Italy,ITA,1993,1.257

Italy,ITA,1994,1.245

Italy,ITA,1995,1.236

Italy,ITA,1996,1.229

Italy,ITA,1997,1.226

Italy,ITA,1998,1.228

Italy,ITA,1999,1.236

Italy,ITA,2000,1.248

Italy,ITA,2001,1.266

Italy,ITA,2002,1.287

Italy,ITA,2003,1.312

Italy,ITA,2004,1.337

Italy,ITA,2005,1.361

Italy,ITA,2006,1.383

Italy,ITA,2007,1.4

Italy,ITA,2008,1.413

Italy,ITA,2009,1.421

Italy,ITA,2010,1.426

Italy,ITA,2011,1.43

Italy,ITA,2012,1.433

Italy,ITA,2013,1.438

Italy,ITA,2014,1.446

Italy,ITA,2015,1.457

Jamaica,JAM,1950,3.802

Jamaica,JAM,1951,3.912

Jamaica,JAM,1952,4.128

Jamaica,JAM,1953,4.331

Jamaica,JAM,1954,4.522

Jamaica,JAM,1955,4.7

Jamaica,JAM,1956,4.867

Jamaica,JAM,1957,5.022

Jamaica,JAM,1958,5.165

Jamaica,JAM,1959,5.298

Jamaica,JAM,1960,5.419

Jamaica,JAM,1961,5.529

Jamaica,JAM,1962,5.627

Jamaica,JAM,1963,5.711

Jamaica,JAM,1964,5.775

Jamaica,JAM,1965,5.815

Jamaica,JAM,1966,5.821

Jamaica,JAM,1967,5.79

Jamaica,JAM,1968,5.722

Jamaica,JAM,1969,5.617

Jamaica,JAM,1970,5.477

Jamaica,JAM,1971,5.305

Jamaica,JAM,1972,5.108

Jamaica,JAM,1973,4.899

Jamaica,JAM,1974,4.685

Jamaica,JAM,1975,4.478

Jamaica,JAM,1976,4.286

Jamaica,JAM,1977,4.115

Jamaica,JAM,1978,3.965

Jamaica,JAM,1979,3.839

Jamaica,JAM,1980,3.733

Jamaica,JAM,1981,3.643

Jamaica,JAM,1982,3.559

Jamaica,JAM,1983,3.477

Jamaica,JAM,1984,3.394

Jamaica,JAM,1985,3.308

Jamaica,JAM,1986,3.223

Jamaica,JAM,1987,3.142

Jamaica,JAM,1988,3.069

Jamaica,JAM,1989,3.003

Jamaica,JAM,1990,2.947

Jamaica,JAM,1991,2.899

Jamaica,JAM,1992,2.86

Jamaica,JAM,1993,2.826

Jamaica,JAM,1994,2.797

Jamaica,JAM,1995,2.768

Jamaica,JAM,1996,2.738

Jamaica,JAM,1997,2.704

Jamaica,JAM,1998,2.666

Jamaica,JAM,1999,2.623

Jamaica,JAM,2000,2.577

Jamaica,JAM,2001,2.529

Jamaica,JAM,2002,2.482

Jamaica,JAM,2003,2.439

Jamaica,JAM,2004,2.398

Jamaica,JAM,2005,2.361

Jamaica,JAM,2006,2.325

Jamaica,JAM,2007,2.289

Jamaica,JAM,2008,2.251

Jamaica,JAM,2009,2.212

Jamaica,JAM,2010,2.173

Jamaica,JAM,2011,2.136

Jamaica,JAM,2012,2.102

Jamaica,JAM,2013,2.072

Jamaica,JAM,2014,2.047

Jamaica,JAM,2015,2.026

Japan,JPN,1950,3.444

Japan,JPN,1951,3.308

Japan,JPN,1952,3.051

Japan,JPN,1953,2.824

Japan,JPN,1954,2.627

Japan,JPN,1955,2.46

Japan,JPN,1956,2.322

Japan,JPN,1957,2.213

Japan,JPN,1958,2.131

Japan,JPN,1959,2.073

Japan,JPN,1960,2.036

Japan,JPN,1961,2.016

Japan,JPN,1962,2.007

Japan,JPN,1963,2.007

Japan,JPN,1964,2.01

Japan,JPN,1965,2.016

Japan,JPN,1966,2.027

Japan,JPN,1967,2.044

Japan,JPN,1968,2.066

Japan,JPN,1969,2.09

Japan,JPN,1970,2.11

Japan,JPN,1971,2.118

Japan,JPN,1972,2.11

Japan,JPN,1973,2.086

Japan,JPN,1974,2.046

Japan,JPN,1975,1.995

Japan,JPN,1976,1.939

Japan,JPN,1977,1.885

Japan,JPN,1978,1.84

Japan,JPN,1979,1.806

Japan,JPN,1980,1.783

Japan,JPN,1981,1.769

Japan,JPN,1982,1.758

Japan,JPN,1983,1.746

Japan,JPN,1984,1.732

Japan,JPN,1985,1.713

Japan,JPN,1986,1.689

Japan,JPN,1987,1.661

Japan,JPN,1988,1.63

Japan,JPN,1989,1.597

Japan,JPN,1990,1.563

Japan,JPN,1991,1.529

Japan,JPN,1992,1.496

Japan,JPN,1993,1.466

Japan,JPN,1994,1.439

Japan,JPN,1995,1.414

Japan,JPN,1996,1.393

Japan,JPN,1997,1.373

Japan,JPN,1998,1.354

Japan,JPN,1999,1.337

Japan,JPN,2000,1.322

Japan,JPN,2001,1.31

Japan,JPN,2002,1.303

Japan,JPN,2003,1.3

Japan,JPN,2004,1.302

Japan,JPN,2005,1.307

Japan,JPN,2006,1.317

Japan,JPN,2007,1.329

Japan,JPN,2008,1.342

Japan,JPN,2009,1.357

Japan,JPN,2010,1.371

Japan,JPN,2011,1.386

Japan,JPN,2012,1.401

Japan,JPN,2013,1.415

Japan,JPN,2014,1.43

Japan,JPN,2015,1.444

Jordan,JOR,1950,7.564

Jordan,JOR,1951,7.5

Jordan,JOR,1952,7.39

Jordan,JOR,1953,7.316

Jordan,JOR,1954,7.278

Jordan,JOR,1955,7.276

Jordan,JOR,1956,7.308

Jordan,JOR,1957,7.371

Jordan,JOR,1958,7.461

Jordan,JOR,1959,7.57

Jordan,JOR,1960,7.687

Jordan,JOR,1961,7.802

Jordan,JOR,1962,7.904

Jordan,JOR,1963,7.983

Jordan,JOR,1964,8.034

Jordan,JOR,1965,8.057

Jordan,JOR,1966,8.053

Jordan,JOR,1967,8.033

Jordan,JOR,1968,8.004

Jordan,JOR,1969,7.968

Jordan,JOR,1970,7.926

Jordan,JOR,1971,7.876

Jordan,JOR,1972,7.818

Jordan,JOR,1973,7.75

Jordan,JOR,1974,7.675

Jordan,JOR,1975,7.598

Jordan,JOR,1976,7.523

Jordan,JOR,1977,7.455

Jordan,JOR,1978,7.391

Jordan,JOR,1979,7.329

Jordan,JOR,1980,7.262

Jordan,JOR,1981,7.18

Jordan,JOR,1982,7.074

Jordan,JOR,1983,6.94

Jordan,JOR,1984,6.777

Jordan,JOR,1985,6.588

Jordan,JOR,1986,6.376

Jordan,JOR,1987,6.153

Jordan,JOR,1988,5.926

Jordan,JOR,1989,5.702

Jordan,JOR,1990,5.488

Jordan,JOR,1991,5.286

Jordan,JOR,1992,5.096

Jordan,JOR,1993,4.918

Jordan,JOR,1994,4.754

Jordan,JOR,1995,4.605

Jordan,JOR,1996,4.469

Jordan,JOR,1997,4.345

Jordan,JOR,1998,4.231

Jordan,JOR,1999,4.126

Jordan,JOR,2000,4.033

Jordan,JOR,2001,3.952

Jordan,JOR,2002,3.884

Jordan,JOR,2003,3.828

Jordan,JOR,2004,3.784

Jordan,JOR,2005,3.75

Jordan,JOR,2006,3.725

Jordan,JOR,2007,3.707

Jordan,JOR,2008,3.693

Jordan,JOR,2009,3.68

Jordan,JOR,2010,3.663

Jordan,JOR,2011,3.638

Jordan,JOR,2012,3.604

Jordan,JOR,2013,3.56

Jordan,JOR,2014,3.506

Jordan,JOR,2015,3.445

Kazakhstan,KAZ,1950,4.305

Kazakhstan,KAZ,1951,4.332

Kazakhstan,KAZ,1952,4.385

Kazakhstan,KAZ,1953,4.434

Kazakhstan,KAZ,1954,4.477

Kazakhstan,KAZ,1955,4.516

Kazakhstan,KAZ,1956,4.549

Kazakhstan,KAZ,1957,4.573

Kazakhstan,KAZ,1958,4.588

Kazakhstan,KAZ,1959,4.586

Kazakhstan,KAZ,1960,4.562

Kazakhstan,KAZ,1961,4.512

Kazakhstan,KAZ,1962,4.432

Kazakhstan,KAZ,1963,4.323

Kazakhstan,KAZ,1964,4.191

Kazakhstan,KAZ,1965,4.048

Kazakhstan,KAZ,1966,3.912

Kazakhstan,KAZ,1967,3.795

Kazakhstan,KAZ,1968,3.705

Kazakhstan,KAZ,1969,3.646

Kazakhstan,KAZ,1970,3.611

Kazakhstan,KAZ,1971,3.59

Kazakhstan,KAZ,1972,3.569

Kazakhstan,KAZ,1973,3.536

Kazakhstan,KAZ,1974,3.488

Kazakhstan,KAZ,1975,3.427

Kazakhstan,KAZ,1976,3.355

Kazakhstan,KAZ,1977,3.283

Kazakhstan,KAZ,1978,3.216

Kazakhstan,KAZ,1979,3.158

Kazakhstan,KAZ,1980,3.114

Kazakhstan,KAZ,1981,3.085

Kazakhstan,KAZ,1982,3.069

Kazakhstan,KAZ,1983,3.063

Kazakhstan,KAZ,1984,3.063

Kazakhstan,KAZ,1985,3.061

Kazakhstan,KAZ,1986,3.05

Kazakhstan,KAZ,1987,3.024

Kazakhstan,KAZ,1988,2.98

Kazakhstan,KAZ,1989,2.916

Kazakhstan,KAZ,1990,2.83

Kazakhstan,KAZ,1991,2.725

Kazakhstan,KAZ,1992,2.605

Kazakhstan,KAZ,1993,2.48

Kazakhstan,KAZ,1994,2.355

Kazakhstan,KAZ,1995,2.237

Kazakhstan,KAZ,1996,2.133

Kazakhstan,KAZ,1997,2.045

Kazakhstan,KAZ,1998,1.978

Kazakhstan,KAZ,1999,1.935

Kazakhstan,KAZ,2000,1.921

Kazakhstan,KAZ,2001,1.941

Kazakhstan,KAZ,2002,1.991

Kazakhstan,KAZ,2003,2.065

Kazakhstan,KAZ,2004,2.157

Kazakhstan,KAZ,2005,2.261

Kazakhstan,KAZ,2006,2.366

Kazakhstan,KAZ,2007,2.466

Kazakhstan,KAZ,2008,2.553

Kazakhstan,KAZ,2009,2.622

Kazakhstan,KAZ,2010,2.669

Kazakhstan,KAZ,2011,2.695

Kazakhstan,KAZ,2012,2.702

Kazakhstan,KAZ,2013,2.697

Kazakhstan,KAZ,2014,2.681

Kazakhstan,KAZ,2015,2.658

Kenya,KEN,1950,7.36

Kenya,KEN,1951,7.39

Kenya,KEN,1952,7.45

Kenya,KEN,1953,7.511

Kenya,KEN,1954,7.573

Kenya,KEN,1955,7.636

Kenya,KEN,1956,7.7

Kenya,KEN,1957,7.763

Kenya,KEN,1958,7.827

Kenya,KEN,1959,7.888

Kenya,KEN,1960,7.946

Kenya,KEN,1961,7.998

Kenya,KEN,1962,8.042

Kenya,KEN,1963,8.078

Kenya,KEN,1964,8.103

Kenya,KEN,1965,8.119

Kenya,KEN,1966,8.126

Kenya,KEN,1967,8.125

Kenya,KEN,1968,8.117

Kenya,KEN,1969,8.103

Kenya,KEN,1970,8.081

Kenya,KEN,1971,8.051

Kenya,KEN,1972,8.012

Kenya,KEN,1973,7.963

Kenya,KEN,1974,7.905

Kenya,KEN,1975,7.84

Kenya,KEN,1976,7.769

Kenya,KEN,1977,7.695

Kenya,KEN,1978,7.618

Kenya,KEN,1979,7.538

Kenya,KEN,1980,7.455

Kenya,KEN,1981,7.365

Kenya,KEN,1982,7.268

Kenya,KEN,1983,7.16

Kenya,KEN,1984,7.042

Kenya,KEN,1985,6.911

Kenya,KEN,1986,6.763

Kenya,KEN,1987,6.599

Kenya,KEN,1988,6.425

Kenya,KEN,1989,6.244

Kenya,KEN,1990,6.066

Kenya,KEN,1991,5.901

Kenya,KEN,1992,5.755

Kenya,KEN,1993,5.633

Kenya,KEN,1994,5.535

Kenya,KEN,1995,5.459

Kenya,KEN,1996,5.4

Kenya,KEN,1997,5.348

Kenya,KEN,1998,5.296

Kenya,KEN,1999,5.239

Kenya,KEN,2000,5.178

Kenya,KEN,2001,5.111

Kenya,KEN,2002,5.044

Kenya,KEN,2003,4.977

Kenya,KEN,2004,4.91

Kenya,KEN,2005,4.84

Kenya,KEN,2006,4.763

Kenya,KEN,2007,4.677

Kenya,KEN,2008,4.582

Kenya,KEN,2009,4.48

Kenya,KEN,2010,4.373

Kenya,KEN,2011,4.267

Kenya,KEN,2012,4.165

Kenya,KEN,2013,4.072

Kenya,KEN,2014,3.989

Kenya,KEN,2015,3.917

Kiribati,KIR,1950,5.846

Kiribati,KIR,1951,5.912

Kiribati,KIR,1952,6.043

Kiribati,KIR,1953,6.167

Kiribati,KIR,1954,6.284

Kiribati,KIR,1955,6.396

Kiribati,KIR,1956,6.5

Kiribati,KIR,1957,6.595

Kiribati,KIR,1958,6.678

Kiribati,KIR,1959,6.745

Kiribati,KIR,1960,6.788

Kiribati,KIR,1961,6.803

Kiribati,KIR,1962,6.784

Kiribati,KIR,1963,6.728

Kiribati,KIR,1964,6.634

Kiribati,KIR,1965,6.501

Kiribati,KIR,1966,6.329

Kiribati,KIR,1967,6.125

Kiribati,KIR,1968,5.902

Kiribati,KIR,1969,5.673

Kiribati,KIR,1970,5.457

Kiribati,KIR,1971,5.273

Kiribati,KIR,1972,5.13

Kiribati,KIR,1973,5.034

Kiribati,KIR,1974,4.985

Kiribati,KIR,1975,4.978

Kiribati,KIR,1976,4.999

Kiribati,KIR,1977,5.031

Kiribati,KIR,1978,5.058

Kiribati,KIR,1979,5.072

Kiribati,KIR,1980,5.07

Kiribati,KIR,1981,5.051

Kiribati,KIR,1982,5.022

Kiribati,KIR,1983,4.988

Kiribati,KIR,1984,4.952

Kiribati,KIR,1985,4.912

Kiribati,KIR,1986,4.871

Kiribati,KIR,1987,4.829

Kiribati,KIR,1988,4.784

Kiribati,KIR,1989,4.738

Kiribati,KIR,1990,4.688

Kiribati,KIR,1991,4.635

Kiribati,KIR,1992,4.576

Kiribati,KIR,1993,4.512

Kiribati,KIR,1994,4.445

Kiribati,KIR,1995,4.374

Kiribati,KIR,1996,4.304

Kiribati,KIR,1997,4.235

Kiribati,KIR,1998,4.17

Kiribati,KIR,1999,4.11

Kiribati,KIR,2000,4.058

Kiribati,KIR,2001,4.015

Kiribati,KIR,2002,3.979

Kiribati,KIR,2003,3.95

Kiribati,KIR,2004,3.927

Kiribati,KIR,2005,3.909

Kiribati,KIR,2006,3.895

Kiribati,KIR,2007,3.883

Kiribati,KIR,2008,3.872

Kiribati,KIR,2009,3.859

Kiribati,KIR,2010,3.843

Kiribati,KIR,2011,3.822

Kiribati,KIR,2012,3.796

Kiribati,KIR,2013,3.766

Kiribati,KIR,2014,3.731

Kiribati,KIR,2015,3.693

Kuwait,KWT,1950,7.218

Kuwait,KWT,1951,7.212

Kuwait,KWT,1952,7.202

Kuwait,KWT,1953,7.194

Kuwait,KWT,1954,7.189

Kuwait,KWT,1955,7.188

Kuwait,KWT,1956,7.19

Kuwait,KWT,1957,7.195

Kuwait,KWT,1958,7.206

Kuwait,KWT,1959,7.221

Kuwait,KWT,1960,7.244

Kuwait,KWT,1961,7.273

Kuwait,KWT,1962,7.309

Kuwait,KWT,1963,7.347

Kuwait,KWT,1964,7.383

Kuwait,KWT,1965,7.409

Kuwait,KWT,1966,7.417

Kuwait,KWT,1967,7.402

Kuwait,KWT,1968,7.358

Kuwait,KWT,1969,7.281

Kuwait,KWT,1970,7.169

Kuwait,KWT,1971,7.018

Kuwait,KWT,1972,6.835

Kuwait,KWT,1973,6.627

Kuwait,KWT,1974,6.403

Kuwait,KWT,1975,6.176

Kuwait,KWT,1976,5.962

Kuwait,KWT,1977,5.769

Kuwait,KWT,1978,5.599

Kuwait,KWT,1979,5.452

Kuwait,KWT,1980,5.316

Kuwait,KWT,1981,5.177

Kuwait,KWT,1982,5.018

Kuwait,KWT,1983,4.83

Kuwait,KWT,1984,4.61

Kuwait,KWT,1985,4.357

Kuwait,KWT,1986,4.074

Kuwait,KWT,1987,3.777

Kuwait,KWT,1988,3.482

Kuwait,KWT,1989,3.205

Kuwait,KWT,1990,2.967

Kuwait,KWT,1991,2.791

Kuwait,KWT,1992,2.682

Kuwait,KWT,1993,2.641

Kuwait,KWT,1994,2.66

Kuwait,KWT,1995,2.72

Kuwait,KWT,1996,2.796

Kuwait,KWT,1997,2.861

Kuwait,KWT,1998,2.895

Kuwait,KWT,1999,2.89

Kuwait,KWT,2000,2.845

Kuwait,KWT,2001,2.773

Kuwait,KWT,2002,2.691

Kuwait,KWT,2003,2.616

Kuwait,KWT,2004,2.552

Kuwait,KWT,2005,2.498

Kuwait,KWT,2006,2.45

Kuwait,KWT,2007,2.4

Kuwait,KWT,2008,2.344

Kuwait,KWT,2009,2.281

Kuwait,KWT,2010,2.215

Kuwait,KWT,2011,2.151

Kuwait,KWT,2012,2.094

Kuwait,KWT,2013,2.046

Kuwait,KWT,2014,2.011

Kuwait,KWT,2015,1.986

Kyrgyzstan,KGZ,1950,3.925

Kyrgyzstan,KGZ,1951,4.042

Kyrgyzstan,KGZ,1952,4.267

Kyrgyzstan,KGZ,1953,4.477

Kyrgyzstan,KGZ,1954,4.672

Kyrgyzstan,KGZ,1955,4.852

Kyrgyzstan,KGZ,1956,5.015

Kyrgyzstan,KGZ,1957,5.161

Kyrgyzstan,KGZ,1958,5.288

Kyrgyzstan,KGZ,1959,5.392

Kyrgyzstan,KGZ,1960,5.469

Kyrgyzstan,KGZ,1961,5.516

Kyrgyzstan,KGZ,1962,5.532

Kyrgyzstan,KGZ,1963,5.517

Kyrgyzstan,KGZ,1964,5.475

Kyrgyzstan,KGZ,1965,5.417

Kyrgyzstan,KGZ,1966,5.354

Kyrgyzstan,KGZ,1967,5.295

Kyrgyzstan,KGZ,1968,5.245

Kyrgyzstan,KGZ,1969,5.207

Kyrgyzstan,KGZ,1970,5.177

Kyrgyzstan,KGZ,1971,5.147

Kyrgyzstan,KGZ,1972,5.105

Kyrgyzstan,KGZ,1973,5.047

Kyrgyzstan,KGZ,1974,4.97

Kyrgyzstan,KGZ,1975,4.876

Kyrgyzstan,KGZ,1976,4.77

Kyrgyzstan,KGZ,1977,4.66

Kyrgyzstan,KGZ,1978,4.553

Kyrgyzstan,KGZ,1979,4.454

Kyrgyzstan,KGZ,1980,4.367

Kyrgyzstan,KGZ,1981,4.296

Kyrgyzstan,KGZ,1982,4.24

Kyrgyzstan,KGZ,1983,4.195

Kyrgyzstan,KGZ,1984,4.16

Kyrgyzstan,KGZ,1985,4.13

Kyrgyzstan,KGZ,1986,4.1

Kyrgyzstan,KGZ,1987,4.065

Kyrgyzstan,KGZ,1988,4.02

Kyrgyzstan,KGZ,1989,3.962

Kyrgyzstan,KGZ,1990,3.888

Kyrgyzstan,KGZ,1991,3.795

Kyrgyzstan,KGZ,1992,3.686

Kyrgyzstan,KGZ,1993,3.566

Kyrgyzstan,KGZ,1994,3.436

Kyrgyzstan,KGZ,1995,3.299

Kyrgyzstan,KGZ,1996,3.159

Kyrgyzstan,KGZ,1997,3.018

Kyrgyzstan,KGZ,1998,2.882

Kyrgyzstan,KGZ,1999,2.759

Kyrgyzstan,KGZ,2000,2.656

Kyrgyzstan,KGZ,2001,2.581

Kyrgyzstan,KGZ,2002,2.536

Kyrgyzstan,KGZ,2003,2.522

Kyrgyzstan,KGZ,2004,2.537

Kyrgyzstan,KGZ,2005,2.58

Kyrgyzstan,KGZ,2006,2.647

Kyrgyzstan,KGZ,2007,2.73

Kyrgyzstan,KGZ,2008,2.82

Kyrgyzstan,KGZ,2009,2.907

Kyrgyzstan,KGZ,2010,2.983

Kyrgyzstan,KGZ,2011,3.04

Kyrgyzstan,KGZ,2012,3.076

Kyrgyzstan,KGZ,2013,3.089

Kyrgyzstan,KGZ,2014,3.08

Kyrgyzstan,KGZ,2015,3.051

Laos,LAO,1950,5.938

Laos,LAO,1951,5.94

Laos,LAO,1952,5.942

Laos,LAO,1953,5.944

Laos,LAO,1954,5.947

Laos,LAO,1955,5.949

Laos,LAO,1956,5.952

Laos,LAO,1957,5.954

Laos,LAO,1958,5.956

Laos,LAO,1959,5.959

Laos,LAO,1960,5.961

Laos,LAO,1961,5.964

Laos,LAO,1962,5.966

Laos,LAO,1963,5.969

Laos,LAO,1964,5.971

Laos,LAO,1965,5.973

Laos,LAO,1966,5.974

Laos,LAO,1967,5.973

Laos,LAO,1968,5.973

Laos,LAO,1969,5.972

Laos,LAO,1970,5.974

Laos,LAO,1971,5.979

Laos,LAO,1972,5.99

Laos,LAO,1973,6.006

Laos,LAO,1974,6.029

Laos,LAO,1975,6.059

Laos,LAO,1976,6.096

Laos,LAO,1977,6.139

Laos,LAO,1978,6.186

Laos,LAO,1979,6.233

Laos,LAO,1980,6.277

Laos,LAO,1981,6.315

Laos,LAO,1982,6.345

Laos,LAO,1983,6.363

Laos,LAO,1984,6.369

Laos,LAO,1985,6.361

Laos,LAO,1986,6.342

Laos,LAO,1987,6.313

Laos,LAO,1988,6.274

Laos,LAO,1989,6.222

Laos,LAO,1990,6.151

Laos,LAO,1991,6.054

Laos,LAO,1992,5.928

Laos,LAO,1993,5.772

Laos,LAO,1994,5.589

Laos,LAO,1995,5.385

Laos,LAO,1996,5.165

Laos,LAO,1997,4.939

Laos,LAO,1998,4.717

Laos,LAO,1999,4.506

Laos,LAO,2000,4.311

Laos,LAO,2001,4.137

Laos,LAO,2002,3.983

Laos,LAO,2003,3.845

Laos,LAO,2004,3.724

Laos,LAO,2005,3.615

Laos,LAO,2006,3.517

Laos,LAO,2007,3.423

Laos,LAO,2008,3.331

Laos,LAO,2009,3.24

Laos,LAO,2010,3.149

Laos,LAO,2011,3.059

Laos,LAO,2012,2.974

Laos,LAO,2013,2.895

Laos,LAO,2014,2.823

Laos,LAO,2015,2.758

Latin America and the Caribbean,,1950,5.885

Latin America and the Caribbean,,1951,5.88

Latin America and the Caribbean,,1952,5.872

Latin America and the Caribbean,,1953,5.868

Latin America and the Caribbean,,1954,5.868

Latin America and the Caribbean,,1955,5.873

Latin America and the Caribbean,,1956,5.88

Latin America and the Caribbean,,1957,5.891

Latin America and the Caribbean,,1958,5.902

Latin America and the Caribbean,,1959,5.911

Latin America and the Caribbean,,1960,5.916

Latin America and the Caribbean,,1961,5.911

Latin America and the Caribbean,,1962,5.894

Latin America and the Caribbean,,1963,5.863

Latin America and the Caribbean,,1964,5.816

Latin America and the Caribbean,,1965,5.753

Latin America and the Caribbean,,1966,5.676

Latin America and the Caribbean,,1967,5.589

Latin America and the Caribbean,,1968,5.495

Latin America and the Caribbean,,1969,5.396

Latin America and the Caribbean,,1970,5.294

Latin America and the Caribbean,,1971,5.19

Latin America and the Caribbean,,1972,5.083

Latin America and the Caribbean,,1973,4.974

Latin America and the Caribbean,,1974,4.864

Latin America and the Caribbean,,1975,4.754

Latin America and the Caribbean,,1976,4.645

Latin America and the Caribbean,,1977,4.536

Latin America and the Caribbean,,1978,4.428

Latin America and the Caribbean,,1979,4.321

Latin America and the Caribbean,,1980,4.215

Latin America and the Caribbean,,1981,4.11

Latin America and the Caribbean,,1982,4.005

Latin America and the Caribbean,,1983,3.901

Latin America and the Caribbean,,1984,3.798

Latin America and the Caribbean,,1985,3.696

Latin America and the Caribbean,,1986,3.598

Latin America and the Caribbean,,1987,3.502

Latin America and the Caribbean,,1988,3.411

Latin America and the Caribbean,,1989,3.324

Latin America and the Caribbean,,1990,3.242

Latin America and the Caribbean,,1991,3.165

Latin America and the Caribbean,,1992,3.092

Latin America and the Caribbean,,1993,3.024

Latin America and the Caribbean,,1994,2.959

Latin America and the Caribbean,,1995,2.897

Latin America and the Caribbean,,1996,2.837

Latin America and the Caribbean,,1997,2.779

Latin America and the Caribbean,,1998,2.723

Latin America and the Caribbean,,1999,2.667

Latin America and the Caribbean,,2000,2.612

Latin America and the Caribbean,,2001,2.558

Latin America and the Caribbean,,2002,2.505

Latin America and the Caribbean,,2003,2.454

Latin America and the Caribbean,,2004,2.405

Latin America and the Caribbean,,2005,2.358

Latin America and the Caribbean,,2006,2.316

Latin America and the Caribbean,,2007,2.279

Latin America and the Caribbean,,2008,2.245

Latin America and the Caribbean,,2009,2.216

Latin America and the Caribbean,,2010,2.191

Latin America and the Caribbean,,2011,2.168

Latin America and the Caribbean,,2012,2.147

Latin America and the Caribbean,,2013,2.126

Latin America and the Caribbean,,2014,2.106

Latin America and the Caribbean,,2015,2.086

Latvia,LVA,1950,2.014

Latvia,LVA,1951,2.011

Latvia,LVA,1952,2.005

Latvia,LVA,1953,1.997

Latvia,LVA,1954,1.988

Latvia,LVA,1955,1.978

Latvia,LVA,1956,1.967

Latvia,LVA,1957,1.955

Latvia,LVA,1958,1.941

Latvia,LVA,1959,1.927

Latvia,LVA,1960,1.911

Latvia,LVA,1961,1.895

Latvia,LVA,1962,1.878

Latvia,LVA,1963,1.862

Latvia,LVA,1964,1.849

Latvia,LVA,1965,1.841

Latvia,LVA,1966,1.841

Latvia,LVA,1967,1.851

Latvia,LVA,1968,1.87

Latvia,LVA,1969,1.897

Latvia,LVA,1970,1.925

Latvia,LVA,1971,1.949

Latvia,LVA,1972,1.964

Latvia,LVA,1973,1.968

Latvia,LVA,1974,1.961

Latvia,LVA,1975,1.946

Latvia,LVA,1976,1.929

Latvia,LVA,1977,1.917

Latvia,LVA,1978,1.914

Latvia,LVA,1979,1.922

Latvia,LVA,1980,1.944

Latvia,LVA,1981,1.979

Latvia,LVA,1982,2.023

Latvia,LVA,1983,2.07

Latvia,LVA,1984,2.113

Latvia,LVA,1985,2.145

Latvia,LVA,1986,2.156

Latvia,LVA,1987,2.142

Latvia,LVA,1988,2.1

Latvia,LVA,1989,2.032

Latvia,LVA,1990,1.94

Latvia,LVA,1991,1.826

Latvia,LVA,1992,1.702

Latvia,LVA,1993,1.577

Latvia,LVA,1994,1.458

Latvia,LVA,1995,1.355

Latvia,LVA,1996,1.273

Latvia,LVA,1997,1.215

Latvia,LVA,1998,1.18

Latvia,LVA,1999,1.168

Latvia,LVA,2000,1.178

Latvia,LVA,2001,1.208

Latvia,LVA,2002,1.251

Latvia,LVA,2003,1.299

Latvia,LVA,2004,1.349

Latvia,LVA,2005,1.395

Latvia,LVA,2006,1.434

Latvia,LVA,2007,1.464

Latvia,LVA,2008,1.485

Latvia,LVA,2009,1.499

Latvia,LVA,2010,1.505

Latvia,LVA,2011,1.507

Latvia,LVA,2012,1.509

Latvia,LVA,2013,1.512

Latvia,LVA,2014,1.52

Latvia,LVA,2015,1.531

Least developed countries,,1950,6.561

Least developed countries,,1951,6.564

Least developed countries,,1952,6.57

Least developed countries,,1953,6.578

Least developed countries,,1954,6.588

Least developed countries,,1955,6.6

Least developed countries,,1956,6.614

Least developed countries,,1957,6.629

Least developed countries,,1958,6.645

Least developed countries,,1959,6.662

Least developed countries,,1960,6.68

Least developed countries,,1961,6.697

Least developed countries,,1962,6.713

Least developed countries,,1963,6.728

Least developed countries,,1964,6.741

Least developed countries,,1965,6.751

Least developed countries,,1966,6.759

Least developed countries,,1967,6.764

Least developed countries,,1968,6.768

Least developed countries,,1969,6.769

Least developed countries,,1970,6.768

Least developed countries,,1971,6.764

Least developed countries,,1972,6.758

Least developed countries,,1973,6.749

Least developed countries,,1974,6.738

Least developed countries,,1975,6.725

Least developed countries,,1976,6.711

Least developed countries,,1977,6.695

Least developed countries,,1978,6.677

Least developed countries,,1979,6.656

Least developed countries,,1980,6.631

Least developed countries,,1981,6.598

Least developed countries,,1982,6.557

Least developed countries,,1983,6.507

Least developed countries,,1984,6.449

Least developed countries,,1985,6.381

Least developed countries,,1986,6.306

Least developed countries,,1987,6.225

Least developed countries,,1988,6.141

Least developed countries,,1989,6.055

Least developed countries,,1990,5.969

Least developed countries,,1991,5.885

Least developed countries,,1992,5.801

Least developed countries,,1993,5.72

Least developed countries,,1994,5.64

Least developed countries,,1995,5.563

Least developed countries,,1996,5.487

Least developed countries,,1997,5.412

Least developed countries,,1998,5.338

Least developed countries,,1999,5.263

Least developed countries,,2000,5.188

Least developed countries,,2001,5.113

Least developed countries,,2002,5.038

Least developed countries,,2003,4.963

Least developed countries,,2004,4.889

Least developed countries,,2005,4.815

Least developed countries,,2006,4.742

Least developed countries,,2007,4.67

Least developed countries,,2008,4.599

Least developed countries,,2009,4.529

Least developed countries,,2010,4.461

Least developed countries,,2011,4.395

Least developed countries,,2012,4.331

Least developed countries,,2013,4.268

Least developed countries,,2014,4.208

Least developed countries,,2015,4.15

Lebanon,LBN,1950,5.76

Lebanon,LBN,1951,5.753

Lebanon,LBN,1952,5.741

Lebanon,LBN,1953,5.733

Lebanon,LBN,1954,5.729

Lebanon,LBN,1955,5.729

Lebanon,LBN,1956,5.732

Lebanon,LBN,1957,5.736

Lebanon,LBN,1958,5.741

Lebanon,LBN,1959,5.743

Lebanon,LBN,1960,5.739

Lebanon,LBN,1961,5.724

Lebanon,LBN,1962,5.694

Lebanon,LBN,1963,5.647

Lebanon,LBN,1964,5.583

Lebanon,LBN,1965,5.501

Lebanon,LBN,1966,5.403

Lebanon,LBN,1967,5.295

Lebanon,LBN,1968,5.181

Lebanon,LBN,1969,5.064

Lebanon,LBN,1970,4.948

Lebanon,LBN,1971,4.836

Lebanon,LBN,1972,4.73

Lebanon,LBN,1973,4.629

Lebanon,LBN,1974,4.534

Lebanon,LBN,1975,4.444

Lebanon,LBN,1976,4.357

Lebanon,LBN,1977,4.271

Lebanon,LBN,1978,4.182

Lebanon,LBN,1979,4.092

Lebanon,LBN,1980,3.997

Lebanon,LBN,1981,3.898

Lebanon,LBN,1982,3.796

Lebanon,LBN,1983,3.692

Lebanon,LBN,1984,3.587

Lebanon,LBN,1985,3.482

Lebanon,LBN,1986,3.379

Lebanon,LBN,1987,3.279

Lebanon,LBN,1988,3.182

Lebanon,LBN,1989,3.09

Lebanon,LBN,1990,3.002

Lebanon,LBN,1991,2.919

Lebanon,LBN,1992,2.841

Lebanon,LBN,1993,2.765

Lebanon,LBN,1994,2.691

Lebanon,LBN,1995,2.617

Lebanon,LBN,1996,2.543

Lebanon,LBN,1997,2.468

Lebanon,LBN,1998,2.39

Lebanon,LBN,1999,2.31

Lebanon,LBN,2000,2.225

Lebanon,LBN,2001,2.134

Lebanon,LBN,2002,2.037

Lebanon,LBN,2003,1.938

Lebanon,LBN,2004,1.839

Lebanon,LBN,2005,1.75

Lebanon,LBN,2006,1.678

Lebanon,LBN,2007,1.628

Lebanon,LBN,2008,1.601

Lebanon,LBN,2009,1.598

Lebanon,LBN,2010,1.614

Lebanon,LBN,2011,1.641

Lebanon,LBN,2012,1.671

Lebanon,LBN,2013,1.696

Lebanon,LBN,2014,1.713

Lebanon,LBN,2015,1.72

Lesotho,LSO,1950,5.811

Lesotho,LSO,1951,5.82

Lesotho,LSO,1952,5.837

Lesotho,LSO,1953,5.849

Lesotho,LSO,1954,5.858

Lesotho,LSO,1955,5.862

Lesotho,LSO,1956,5.863

Lesotho,LSO,1957,5.861

Lesotho,LSO,1958,5.856

Lesotho,LSO,1959,5.848

Lesotho,LSO,1960,5.839

Lesotho,LSO,1961,5.829

Lesotho,LSO,1962,5.82

Lesotho,LSO,1963,5.812

Lesotho,LSO,1964,5.806

Lesotho,LSO,1965,5.802

Lesotho,LSO,1966,5.801

Lesotho,LSO,1967,5.803

Lesotho,LSO,1968,5.805

Lesotho,LSO,1969,5.807

Lesotho,LSO,1970,5.808

Lesotho,LSO,1971,5.807

Lesotho,LSO,1972,5.802

Lesotho,LSO,1973,5.794

Lesotho,LSO,1974,5.78

Lesotho,LSO,1975,5.761

Lesotho,LSO,1976,5.736

Lesotho,LSO,1977,5.706

Lesotho,LSO,1978,5.672

Lesotho,LSO,1979,5.633

Lesotho,LSO,1980,5.589

Lesotho,LSO,1981,5.542

Lesotho,LSO,1982,5.491

Lesotho,LSO,1983,5.436

Lesotho,LSO,1984,5.377

Lesotho,LSO,1985,5.313

Lesotho,LSO,1986,5.244

Lesotho,LSO,1987,5.169

Lesotho,LSO,1988,5.088

Lesotho,LSO,1989,5.004

Lesotho,LSO,1990,4.919

Lesotho,LSO,1991,4.837

Lesotho,LSO,1992,4.759

Lesotho,LSO,1993,4.686

Lesotho,LSO,1994,4.617

Lesotho,LSO,1995,4.548

Lesotho,LSO,1996,4.475

Lesotho,LSO,1997,4.394

Lesotho,LSO,1998,4.302

Lesotho,LSO,1999,4.2

Lesotho,LSO,2000,4.089

Lesotho,LSO,2001,3.972

Lesotho,LSO,2002,3.856

Lesotho,LSO,2003,3.744

Lesotho,LSO,2004,3.64

Lesotho,LSO,2005,3.548

Lesotho,LSO,2006,3.473

Lesotho,LSO,2007,3.412

Lesotho,LSO,2008,3.365

Lesotho,LSO,2009,3.33

Lesotho,LSO,2010,3.303

Lesotho,LSO,2011,3.278

Lesotho,LSO,2012,3.253

Lesotho,LSO,2013,3.222

Lesotho,LSO,2014,3.186

Lesotho,LSO,2015,3.143

Less developed regions,,1950,6.223

Less developed regions,,1951,6.172

Less developed regions,,1952,6.081

Less developed regions,,1953,6.011

Less developed regions,,1954,5.961

Less developed regions,,1955,5.932

Less developed regions,,1956,5.922

Less developed regions,,1957,5.93

Less developed regions,,1958,5.954

Less developed regions,,1959,5.991

Less developed regions,,1960,6.035

Less developed regions,,1961,6.082

Less developed regions,,1962,6.123

Less developed regions,,1963,6.153

Less developed regions,,1964,6.167

Less developed regions,,1965,6.158

Less developed regions,,1966,6.124

Less developed regions,,1967,6.067

Less developed regions,,1968,5.988

Less developed regions,,1969,5.888

Less developed regions,,1970,5.768

Less developed regions,,1971,5.629

Less developed regions,,1972,5.474

Less developed regions,,1973,5.309

Less developed regions,,1974,5.14

Less developed regions,,1975,4.973

Less developed regions,,1976,4.816

Less developed regions,,1977,4.672

Less developed regions,,1978,4.543

Less developed regions,,1979,4.432

Less developed regions,,1980,4.339

Less developed regions,,1981,4.266

Less developed regions,,1982,4.205

Less developed regions,,1983,4.153

Less developed regions,,1984,4.104

Less developed regions,,1985,4.052

Less developed regions,,1986,3.992

Less developed regions,,1987,3.921

Less developed regions,,1988,3.838

Less developed regions,,1989,3.743

Less developed regions,,1990,3.64

Less developed regions,,1991,3.532

Less developed regions,,1992,3.425

Less developed regions,,1993,3.325

Less developed regions,,1994,3.235

Less developed regions,,1995,3.156

Less developed regions,,1996,3.089

Less developed regions,,1997,3.033

Less developed regions,,1998,2.984

Less developed regions,,1999,2.942

Less developed regions,,2000,2.906

Less developed regions,,2001,2.875

Less developed regions,,2002,2.847

Less developed regions,,2003,2.823

Less developed regions,,2004,2.8

Less developed regions,,2005,2.778

Less developed regions,,2006,2.758

Less developed regions,,2007,2.739

Less developed regions,,2008,2.721

Less developed regions,,2009,2.704

Less developed regions,,2010,2.688

Less developed regions,,2011,2.673

Less developed regions,,2012,2.659

Less developed regions,,2013,2.645

Less developed regions,,2014,2.632

Less developed regions,,2015,2.619

"Less developed regions, excluding China",,1950,6.028

"Less developed regions, excluding China",,1951,6.04

"Less developed regions, excluding China",,1952,6.064

"Less developed regions, excluding China",,1953,6.086

"Less developed regions, excluding China",,1954,6.106

"Less developed regions, excluding China",,1955,6.123

"Less developed regions, excluding China",,1956,6.137

"Less developed regions, excluding China",,1957,6.149

"Less developed regions, excluding China",,1958,6.158

"Less developed regions, excluding China",,1959,6.163

"Less developed regions, excluding China",,1960,6.165

"Less developed regions, excluding China",,1961,6.161

"Less developed regions, excluding China",,1962,6.151

"Less developed regions, excluding China",,1963,6.136

"Less developed regions, excluding China",,1964,6.113

"Less developed regions, excluding China",,1965,6.084

"Less developed regions, excluding China",,1966,6.049

"Less developed regions, excluding China",,1967,6.008

"Less developed regions, excluding China",,1968,5.962

"Less developed regions, excluding China",,1969,5.911

"Less developed regions, excluding China",,1970,5.854

"Less developed regions, excluding China",,1971,5.792

"Less developed regions, excluding China",,1972,5.724

"Less developed regions, excluding China",,1973,5.65

"Less developed regions, excluding China",,1974,5.572

"Less developed regions, excluding China",,1975,5.492

"Less developed regions, excluding China",,1976,5.411

"Less developed regions, excluding China",,1977,5.332

"Less developed regions, excluding China",,1978,5.255

"Less developed regions, excluding China",,1979,5.18

"Less developed regions, excluding China",,1980,5.107

"Less developed regions, excluding China",,1981,5.033

"Less developed regions, excluding China",,1982,4.956

"Less developed regions, excluding China",,1983,4.876

"Less developed regions, excluding China",,1984,4.791

"Less developed regions, excluding China",,1985,4.702

"Less developed regions, excluding China",,1986,4.61

"Less developed regions, excluding China",,1987,4.515

"Less developed regions, excluding China",,1988,4.419

"Less developed regions, excluding China",,1989,4.324

"Less developed regions, excluding China",,1990,4.23

"Less developed regions, excluding China",,1991,4.139

"Less developed regions, excluding China",,1992,4.051

"Less developed regions, excluding China",,1993,3.965

"Less developed regions, excluding China",,1994,3.883

"Less developed regions, excluding China",,1995,3.805

"Less developed regions, excluding China",,1996,3.731

"Less developed regions, excluding China",,1997,3.66

"Less developed regions, excluding China",,1998,3.594

"Less developed regions, excluding China",,1999,3.531

"Less developed regions, excluding China",,2000,3.471

"Less developed regions, excluding China",,2001,3.416

"Less developed regions, excluding China",,2002,3.364

"Less developed regions, excluding China",,2003,3.316

"Less developed regions, excluding China",,2004,3.271

"Less developed regions, excluding China",,2005,3.229

"Less developed regions, excluding China",,2006,3.189

"Less developed regions, excluding China",,2007,3.151

"Less developed regions, excluding China",,2008,3.116

"Less developed regions, excluding China",,2009,3.081

"Less developed regions, excluding China",,2010,3.048

"Less developed regions, excluding China",,2011,3.017

"Less developed regions, excluding China",,2012,2.986

"Less developed regions, excluding China",,2013,2.957

"Less developed regions, excluding China",,2014,2.928

"Less developed regions, excluding China",,2015,2.901

"Less developed regions, excluding least developed countries",,1950,6.18

"Less developed regions, excluding least developed countries",,1951,6.123

"Less developed regions, excluding least developed countries",,1952,6.019

"Less developed regions, excluding least developed countries",,1953,5.938

"Less developed regions, excluding least developed countries",,1954,5.88

"Less developed regions, excluding least developed countries",,1955,5.844

"Less developed regions, excluding least developed countries",,1956,5.831

"Less developed regions, excluding least developed countries",,1957,5.838

"Less developed regions, excluding least developed countries",,1958,5.862

"Less developed regions, excluding least developed countries",,1959,5.901

"Less developed regions, excluding least developed countries",,1960,5.949

"Less developed regions, excluding least developed countries",,1961,5.999

"Less developed regions, excluding least developed countries",,1962,6.043

"Less developed regions, excluding least developed countries",,1963,6.076

"Less developed regions, excluding least developed countries",,1964,6.09

"Less developed regions, excluding least developed countries",,1965,6.079

"Less developed regions, excluding least developed countries",,1966,6.04

"Less developed regions, excluding least developed countries",,1967,5.975

"Less developed regions, excluding least developed countries",,1968,5.886

"Less developed regions, excluding least developed countries",,1969,5.773

"Less developed regions, excluding least developed countries",,1970,5.639

"Less developed regions, excluding least developed countries",,1971,5.482

"Less developed regions, excluding least developed countries",,1972,5.308

"Less developed regions, excluding least developed countries",,1973,5.123

"Less developed regions, excluding least developed countries",,1974,4.933

"Less developed regions, excluding least developed countries",,1975,4.747

"Less developed regions, excluding least developed countries",,1976,4.572

"Less developed regions, excluding least developed countries",,1977,4.412

"Less developed regions, excluding least developed countries",,1978,4.27

"Less developed regions, excluding least developed countries",,1979,4.149

"Less developed regions, excluding least developed countries",,1980,4.049

"Less developed regions, excluding least developed countries",,1981,3.972

"Less developed regions, excluding least developed countries",,1982,3.911

"Less developed regions, excluding least developed countries",,1983,3.859

"Less developed regions, excluding least developed countries",,1984,3.813

"Less developed regions, excluding least developed countries",,1985,3.765

"Less developed regions, excluding least developed countries",,1986,3.707

"Less developed regions, excluding least developed countries",,1987,3.638

"Less developed regions, excluding least developed countries",,1988,3.555

"Less developed regions, excluding least developed countries",,1989,3.459

"Less developed regions, excluding least developed countries",,1990,3.352

"Less developed regions, excluding least developed countries",,1991,3.24

"Less developed regions, excluding least developed countries",,1992,3.128

"Less developed regions, excluding least developed countries",,1993,3.023

"Less developed regions, excluding least developed countries",,1994,2.928

"Less developed regions, excluding least developed countries",,1995,2.846

"Less developed regions, excluding least developed countries",,1996,2.778

"Less developed regions, excluding least developed countries",,1997,2.72

"Less developed regions, excluding least developed countries",,1998,2.67

"Less developed regions, excluding least developed countries",,1999,2.629

"Less developed regions, excluding least developed countries",,2000,2.594

"Less developed regions, excluding least developed countries",,2001,2.565

"Less developed regions, excluding least developed countries",,2002,2.54

"Less developed regions, excluding least developed countries",,2003,2.518

"Less developed regions, excluding least developed countries",,2004,2.498

"Less developed regions, excluding least developed countries",,2005,2.479

"Less developed regions, excluding least developed countries",,2006,2.461

"Less developed regions, excluding least developed countries",,2007,2.445

"Less developed regions, excluding least developed countries",,2008,2.43

"Less developed regions, excluding least developed countries",,2009,2.416

"Less developed regions, excluding least developed countries",,2010,2.403

"Less developed regions, excluding least developed countries",,2011,2.39

"Less developed regions, excluding least developed countries",,2012,2.378

"Less developed regions, excluding least developed countries",,2013,2.366

"Less developed regions, excluding least developed countries",,2014,2.354

"Less developed regions, excluding least developed countries",,2015,2.343

Liberia,LBR,1950,6.251

Liberia,LBR,1951,6.255

Liberia,LBR,1952,6.264

Liberia,LBR,1953,6.275

Liberia,LBR,1954,6.289

Liberia,LBR,1955,6.304

Liberia,LBR,1956,6.321

Liberia,LBR,1957,6.34

Liberia,LBR,1958,6.361

Liberia,LBR,1959,6.383

Liberia,LBR,1960,6.406

Liberia,LBR,1961,6.429

Liberia,LBR,1962,6.453

Liberia,LBR,1963,6.476

Liberia,LBR,1964,6.5

Liberia,LBR,1965,6.524

Liberia,LBR,1966,6.552

Liberia,LBR,1967,6.583

Liberia,LBR,1968,6.617

Liberia,LBR,1969,6.655

Liberia,LBR,1970,6.695

Liberia,LBR,1971,6.736

Liberia,LBR,1972,6.777

Liberia,LBR,1973,6.815

Liberia,LBR,1974,6.849

Liberia,LBR,1975,6.88

Liberia,LBR,1976,6.907

Liberia,LBR,1977,6.93

Liberia,LBR,1978,6.95

Liberia,LBR,1979,6.964

Liberia,LBR,1980,6.973

Liberia,LBR,1981,6.973

Liberia,LBR,1982,6.964

Liberia,LBR,1983,6.946

Liberia,LBR,1984,6.917

Liberia,LBR,1985,6.875

Liberia,LBR,1986,6.82

Liberia,LBR,1987,6.752

Liberia,LBR,1988,6.673

Liberia,LBR,1989,6.587

Liberia,LBR,1990,6.499

Liberia,LBR,1991,6.414

Liberia,LBR,1992,6.336

Liberia,LBR,1993,6.267

Liberia,LBR,1994,6.208

Liberia,LBR,1995,6.156

Liberia,LBR,1996,6.109

Liberia,LBR,1997,6.061

Liberia,LBR,1998,6.008

Liberia,LBR,1999,5.948

Liberia,LBR,2000,5.88

Liberia,LBR,2001,5.804

Liberia,LBR,2002,5.722

Liberia,LBR,2003,5.635

Liberia,LBR,2004,5.547

Liberia,LBR,2005,5.456

Liberia,LBR,2006,5.366

Liberia,LBR,2007,5.277

Liberia,LBR,2008,5.19

Liberia,LBR,2009,5.105

Liberia,LBR,2010,5.023

Liberia,LBR,2011,4.944

Liberia,LBR,2012,4.868

Liberia,LBR,2013,4.794

Liberia,LBR,2014,4.721

Liberia,LBR,2015,4.65

Libya,LBY,1950,7.104

Libya,LBY,1951,7.117

Libya,LBY,1952,7.139

Libya,LBY,1953,7.156

Libya,LBY,1954,7.167

Libya,LBY,1955,7.173

Libya,LBY,1956,7.174

Libya,LBY,1957,7.173

Libya,LBY,1958,7.173

Libya,LBY,1959,7.181

Libya,LBY,1960,7.202

Libya,LBY,1961,7.243

Libya,LBY,1962,7.31

Libya,LBY,1963,7.402

Libya,LBY,1964,7.517

Libya,LBY,1965,7.647

Libya,LBY,1966,7.781

Libya,LBY,1967,7.905

Libya,LBY,1968,8.01

Libya,LBY,1969,8.087

Libya,LBY,1970,8.132

Libya,LBY,1971,8.147

Libya,LBY,1972,8.135

Libya,LBY,1973,8.102

Libya,LBY,1974,8.048

Libya,LBY,1975,7.97

Libya,LBY,1976,7.866

Libya,LBY,1977,7.736

Libya,LBY,1978,7.582

Libya,LBY,1979,7.408

Libya,LBY,1980,7.219

Libya,LBY,1981,7.023

Libya,LBY,1982,6.826

Libya,LBY,1983,6.63

Libya,LBY,1984,6.435

Libya,LBY,1985,6.236

Libya,LBY,1986,6.023

Libya,LBY,1987,5.79

Libya,LBY,1988,5.533

Libya,LBY,1989,5.256

Libya,LBY,1990,4.966

Libya,LBY,1991,4.67

Libya,LBY,1992,4.381

Libya,LBY,1993,4.109

Libya,LBY,1994,3.859

Libya,LBY,1995,3.636

Libya,LBY,1996,3.44

Libya,LBY,1997,3.267

Libya,LBY,1998,3.112

Libya,LBY,1999,2.976

Libya,LBY,2000,2.856

Libya,LBY,2001,2.754

Libya,LBY,2002,2.668

Libya,LBY,2003,2.597

Libya,LBY,2004,2.539

Libya,LBY,2005,2.493

Libya,LBY,2006,2.46

Libya,LBY,2007,2.438

Libya,LBY,2008,2.425

Libya,LBY,2009,2.418

Libya,LBY,2010,2.412

Libya,LBY,2011,2.404

Libya,LBY,2012,2.391

Libya,LBY,2013,2.372

Libya,LBY,2014,2.345

Libya,LBY,2015,2.312

Lithuania,LTU,1950,2.895

Lithuania,LTU,1951,2.883

Lithuania,LTU,1952,2.857

Lithuania,LTU,1953,2.828

Lithuania,LTU,1954,2.795

Lithuania,LTU,1955,2.759

Lithuania,LTU,1956,2.719

Lithuania,LTU,1957,2.677

Lithuania,LTU,1958,2.632

Lithuania,LTU,1959,2.586

Lithuania,LTU,1960,2.539

Lithuania,LTU,1961,2.493

Lithuania,LTU,1962,2.45

Lithuania,LTU,1963,2.41

Lithuania,LTU,1964,2.376

Lithuania,LTU,1965,2.348

Lithuania,LTU,1966,2.328

Lithuania,LTU,1967,2.316

Lithuania,LTU,1968,2.311

Lithuania,LTU,1969,2.309

Lithuania,LTU,1970,2.308

Lithuania,LTU,1971,2.303

Lithuania,LTU,1972,2.291

Lithuania,LTU,1973,2.271

Lithuania,LTU,1974,2.243

Lithuania,LTU,1975,2.208

Lithuania,LTU,1976,2.17

Lithuania,LTU,1977,2.133

Lithuania,LTU,1978,2.1

Lithuania,LTU,1979,2.074

Lithuania,LTU,1980,2.056

Lithuania,LTU,1981,2.048

Lithuania,LTU,1982,2.047

Lithuania,LTU,1983,2.05

Lithuania,LTU,1984,2.056

Lithuania,LTU,1985,2.06

Lithuania,LTU,1986,2.06

Lithuania,LTU,1987,2.051

Lithuania,LTU,1988,2.033

Lithuania,LTU,1989,2.004

Lithuania,LTU,1990,1.963

Lithuania,LTU,1991,1.91

Lithuania,LTU,1992,1.848

Lithuania,LTU,1993,1.781

Lithuania,LTU,1994,1.711

Lithuania,LTU,1995,1.64

Lithuania,LTU,1996,1.57

Lithuania,LTU,1997,1.502

Lithuania,LTU,1998,1.439

Lithuania,LTU,1999,1.383

Lithuania,LTU,2000,1.338

Lithuania,LTU,2001,1.307

Lithuania,LTU,2002,1.29

Lithuania,LTU,2003,1.287

Lithuania,LTU,2004,1.297

Lithuania,LTU,2005,1.319

Lithuania,LTU,2006,1.351

Lithuania,LTU,2007,1.389

Lithuania,LTU,2008,1.431

Lithuania,LTU,2009,1.472

Lithuania,LTU,2010,1.51

Lithuania,LTU,2011,1.544

Lithuania,LTU,2012,1.574

Lithuania,LTU,2013,1.6

Lithuania,LTU,2014,1.621

Lithuania,LTU,2015,1.637

Low-income countries,,1950,6.279

Low-income countries,,1951,6.314

Low-income countries,,1952,6.377

Low-income countries,,1953,6.429

Low-income countries,,1954,6.469

Low-income countries,,1955,6.498

Low-income countries,,1956,6.517

Low-income countries,,1957,6.526

Low-income countries,,1958,6.526

Low-income countries,,1959,6.521

Low-income countries,,1960,6.513

Low-income countries,,1961,6.505

Low-income countries,,1962,6.502

Low-income countries,,1963,6.505

Low-income countries,,1964,6.516

Low-income countries,,1965,6.532

Low-income countries,,1966,6.553

Low-income countries,,1967,6.574

Low-income countries,,1968,6.593

Low-income countries,,1969,6.608

Low-income countries,,1970,6.616

Low-income countries,,1971,6.618

Low-income countries,,1972,6.615

Low-income countries,,1973,6.606

Low-income countries,,1974,6.595

Low-income countries,,1975,6.582

Low-income countries,,1976,6.571

Low-income countries,,1977,6.563

Low-income countries,,1978,6.558

Low-income countries,,1979,6.557

Low-income countries,,1980,6.559

Low-income countries,,1981,6.559

Low-income countries,,1982,6.557

Low-income countries,,1983,6.549

Low-income countries,,1984,6.534

Low-income countries,,1985,6.512

Low-income countries,,1986,6.484

Low-income countries,,1987,6.451

Low-income countries,,1988,6.415

Low-income countries,,1989,6.376

Low-income countries,,1990,6.336

Low-income countries,,1991,6.295

Low-income countries,,1992,6.254

Low-income countries,,1993,6.213

Low-income countries,,1994,6.17

Low-income countries,,1995,6.127

Low-income countries,,1996,6.082

Low-income countries,,1997,6.034

Low-income countries,,1998,5.983

Low-income countries,,1999,5.928

Low-income countries,,2000,5.871

Low-income countries,,2001,5.81

Low-income countries,,2002,5.748

Low-income countries,,2003,5.684

Low-income countries,,2004,5.62

Low-income countries,,2005,5.554

Low-income countries,,2006,5.486

Low-income countries,,2007,5.416

Low-income countries,,2008,5.344

Low-income countries,,2009,5.269

Low-income countries,,2010,5.193

Low-income countries,,2011,5.115

Low-income countries,,2012,5.037

Low-income countries,,2013,4.959

Low-income countries,,2014,4.882

Low-income countries,,2015,4.805

Lower-middle-income countries,,1950,5.797

Lower-middle-income countries,,1951,5.811

Lower-middle-income countries,,1952,5.837

Lower-middle-income countries,,1953,5.861

Lower-middle-income countries,,1954,5.882

Lower-middle-income countries,,1955,5.899

Lower-middle-income countries,,1956,5.914

Lower-middle-income countries,,1957,5.926

Lower-middle-income countries,,1958,5.935

Lower-middle-income countries,,1959,5.941

Lower-middle-income countries,,1960,5.943

Lower-middle-income countries,,1961,5.942

Lower-middle-income countries,,1962,5.937

Lower-middle-income countries,,1963,5.928

Lower-middle-income countries,,1964,5.915

Lower-middle-income countries,,1965,5.897

Lower-middle-income countries,,1966,5.874

Lower-middle-income countries,,1967,5.848

Lower-middle-income countries,,1968,5.817

Lower-middle-income countries,,1969,5.782

Lower-middle-income countries,,1970,5.741

Lower-middle-income countries,,1971,5.694

Lower-middle-income countries,,1972,5.641

Lower-middle-income countries,,1973,5.581

Lower-middle-income countries,,1974,5.516

Lower-middle-income countries,,1975,5.448

Lower-middle-income countries,,1976,5.379

Lower-middle-income countries,,1977,5.311

Lower-middle-income countries,,1978,5.243

Lower-middle-income countries,,1979,5.178

Lower-middle-income countries,,1980,5.113

Lower-middle-income countries,,1981,5.047

Lower-middle-income countries,,1982,4.977

Lower-middle-income countries,,1983,4.903

Lower-middle-income countries,,1984,4.824

Lower-middle-income countries,,1985,4.739

Lower-middle-income countries,,1986,4.649

Lower-middle-income countries,,1987,4.554

Lower-middle-income countries,,1988,4.458

Lower-middle-income countries,,1989,4.361

Lower-middle-income countries,,1990,4.263

Lower-middle-income countries,,1991,4.167

Lower-middle-income countries,,1992,4.073

Lower-middle-income countries,,1993,3.982

Lower-middle-income countries,,1994,3.894

Lower-middle-income countries,,1995,3.811

Lower-middle-income countries,,1996,3.732

Lower-middle-income countries,,1997,3.658

Lower-middle-income countries,,1998,3.589

Lower-middle-income countries,,1999,3.523

Lower-middle-income countries,,2000,3.462

Lower-middle-income countries,,2001,3.405

Lower-middle-income countries,,2002,3.35

Lower-middle-income countries,,2003,3.299

Lower-middle-income countries,,2004,3.249

Lower-middle-income countries,,2005,3.201

Lower-middle-income countries,,2006,3.155

Lower-middle-income countries,,2007,3.109

Lower-middle-income countries,,2008,3.065

Lower-middle-income countries,,2009,3.022

Lower-middle-income countries,,2010,2.98

Lower-middle-income countries,,2011,2.941

Lower-middle-income countries,,2012,2.903

Lower-middle-income countries,,2013,2.868

Lower-middle-income countries,,2014,2.836

Lower-middle-income countries,,2015,2.806

Luxembourg,LUX,1950,1.868

Luxembourg,LUX,1951,1.896

Luxembourg,LUX,1952,1.953

Luxembourg,LUX,1953,2.008

Luxembourg,LUX,1954,2.063

Luxembourg,LUX,1955,2.117

Luxembourg,LUX,1956,2.17

Luxembourg,LUX,1957,2.221

Luxembourg,LUX,1958,2.269

Luxembourg,LUX,1959,2.312

Luxembourg,LUX,1960,2.349

Luxembourg,LUX,1961,2.375

Luxembourg,LUX,1962,2.39

Luxembourg,LUX,1963,2.39

Luxembourg,LUX,1964,2.374

Luxembourg,LUX,1965,2.342

Luxembourg,LUX,1966,2.291

Luxembourg,LUX,1967,2.223

Luxembourg,LUX,1968,2.143

Luxembourg,LUX,1969,2.054

Luxembourg,LUX,1970,1.96

Luxembourg,LUX,1971,1.867

Luxembourg,LUX,1972,1.778

Luxembourg,LUX,1973,1.697

Luxembourg,LUX,1974,1.627

Luxembourg,LUX,1975,1.57

Luxembourg,LUX,1976,1.528

Luxembourg,LUX,1977,1.497

Luxembourg,LUX,1978,1.476

Luxembourg,LUX,1979,1.463

Luxembourg,LUX,1980,1.456

Luxembourg,LUX,1981,1.452

Luxembourg,LUX,1982,1.45

Luxembourg,LUX,1983,1.448

Luxembourg,LUX,1984,1.448

Luxembourg,LUX,1985,1.45

Luxembourg,LUX,1986,1.458

Luxembourg,LUX,1987,1.473

Luxembourg,LUX,1988,1.496

Luxembourg,LUX,1989,1.526

Luxembourg,LUX,1990,1.562

Luxembourg,LUX,1991,1.6

Luxembourg,LUX,1992,1.637

Luxembourg,LUX,1993,1.669

Luxembourg,LUX,1994,1.694

Luxembourg,LUX,1995,1.711

Luxembourg,LUX,1996,1.72

Luxembourg,LUX,1997,1.721

Luxembourg,LUX,1998,1.716

Luxembourg,LUX,1999,1.707

Luxembourg,LUX,2000,1.695

Luxembourg,LUX,2001,1.682

Luxembourg,LUX,2002,1.669

Luxembourg,LUX,2003,1.657

Luxembourg,LUX,2004,1.647

Luxembourg,LUX,2005,1.638

Luxembourg,LUX,2006,1.629

Luxembourg,LUX,2007,1.618

Luxembourg,LUX,2008,1.605

Luxembourg,LUX,2009,1.591

Luxembourg,LUX,2010,1.577

Luxembourg,LUX,2011,1.565

Luxembourg,LUX,2012,1.557

Luxembourg,LUX,2013,1.554

Luxembourg,LUX,2014,1.555

Luxembourg,LUX,2015,1.562

Macao,MAC,1950,4.051

Macao,MAC,1951,4.147

Macao,MAC,1952,4.326

Macao,MAC,1953,4.482

Macao,MAC,1954,4.612

Macao,MAC,1955,4.718

Macao,MAC,1956,4.797

Macao,MAC,1957,4.848

Macao,MAC,1958,4.865

Macao,MAC,1959,4.842

Macao,MAC,1960,4.772

Macao,MAC,1961,4.646

Macao,MAC,1962,4.462

Macao,MAC,1963,4.221

Macao,MAC,1964,3.934

Macao,MAC,1965,3.615

Macao,MAC,1966,3.283

Macao,MAC,1967,2.958

Macao,MAC,1968,2.657

Macao,MAC,1969,2.392

Macao,MAC,1970,2.167

Macao,MAC,1971,1.979

Macao,MAC,1972,1.818

Macao,MAC,1973,1.679

Macao,MAC,1974,1.562

Macao,MAC,1975,1.478

Macao,MAC,1976,1.437

Macao,MAC,1977,1.445

Macao,MAC,1978,1.499

Macao,MAC,1979,1.591

Macao,MAC,1980,1.709

Macao,MAC,1981,1.835

Macao,MAC,1982,1.953

Macao,MAC,1983,2.046

Macao,MAC,1984,2.104

Macao,MAC,1985,2.121

Macao,MAC,1986,2.094

Macao,MAC,1987,2.031

Macao,MAC,1988,1.944

Macao,MAC,1989,1.838

Macao,MAC,1990,1.722

Macao,MAC,1991,1.606

Macao,MAC,1992,1.495

Macao,MAC,1993,1.395

Macao,MAC,1994,1.309

Macao,MAC,1995,1.238

Macao,MAC,1996,1.175

Macao,MAC,1997,1.116

Macao,MAC,1998,1.056

Macao,MAC,1999,0.995

Macao,MAC,2000,0.938

Macao,MAC,2001,0.889

Macao,MAC,2002,0.852

Macao,MAC,2003,0.831

Macao,MAC,2004,0.827

Macao,MAC,2005,0.84

Macao,MAC,2006,0.869

Macao,MAC,2007,0.909

Macao,MAC,2008,0.957

Macao,MAC,2009,1.008

Macao,MAC,2010,1.061

Macao,MAC,2011,1.112

Macao,MAC,2012,1.16

Macao,MAC,2013,1.205

Macao,MAC,2014,1.245

Macao,MAC,2015,1.28

Macedonia,MKD,1950,5.521

Macedonia,MKD,1951,5.397

Macedonia,MKD,1952,5.157

Macedonia,MKD,1953,4.933

Macedonia,MKD,1954,4.726

Macedonia,MKD,1955,4.536

Macedonia,MKD,1956,4.362

Macedonia,MKD,1957,4.205

Macedonia,MKD,1958,4.066

Macedonia,MKD,1959,3.945

Macedonia,MKD,1960,3.842

Macedonia,MKD,1961,3.758

Macedonia,MKD,1962,3.691

Macedonia,MKD,1963,3.638

Macedonia,MKD,1964,3.595

Macedonia,MKD,1965,3.552

Macedonia,MKD,1966,3.502

Macedonia,MKD,1967,3.439

Macedonia,MKD,1968,3.36

Macedonia,MKD,1969,3.265

Macedonia,MKD,1970,3.158

Macedonia,MKD,1971,3.044

Macedonia,MKD,1972,2.932

Macedonia,MKD,1973,2.828

Macedonia,MKD,1974,2.738

Macedonia,MKD,1975,2.663

Macedonia,MKD,1976,2.606

Macedonia,MKD,1977,2.562

Macedonia,MKD,1978,2.529

Macedonia,MKD,1979,2.505

Macedonia,MKD,1980,2.486

Macedonia,MKD,1981,2.468

Macedonia,MKD,1982,2.449

Macedonia,MKD,1983,2.426

Macedonia,MKD,1984,2.399

Macedonia,MKD,1985,2.368

Macedonia,MKD,1986,2.335

Macedonia,MKD,1987,2.301

Macedonia,MKD,1988,2.269

Macedonia,MKD,1989,2.238

Macedonia,MKD,1990,2.206

Macedonia,MKD,1991,2.171

Macedonia,MKD,1992,2.131

Macedonia,MKD,1993,2.085

Macedonia,MKD,1994,2.033

Macedonia,MKD,1995,1.977

Macedonia,MKD,1996,1.919

Macedonia,MKD,1997,1.864

Macedonia,MKD,1998,1.812

Macedonia,MKD,1999,1.765

Macedonia,MKD,2000,1.723

Macedonia,MKD,2001,1.683

Macedonia,MKD,2002,1.644

Macedonia,MKD,2003,1.605

Macedonia,MKD,2004,1.566

Macedonia,MKD,2005,1.531

Macedonia,MKD,2006,1.501

Macedonia,MKD,2007,1.478

Macedonia,MKD,2008,1.465

Macedonia,MKD,2009,1.461

Macedonia,MKD,2010,1.465

Macedonia,MKD,2011,1.474

Macedonia,MKD,2012,1.487

Macedonia,MKD,2013,1.501

Macedonia,MKD,2014,1.513

Macedonia,MKD,2015,1.524

Madagascar,MDG,1950,7.299

Madagascar,MDG,1951,7.299

Madagascar,MDG,1952,7.3

Madagascar,MDG,1953,7.3

Madagascar,MDG,1954,7.301

Madagascar,MDG,1955,7.301

Madagascar,MDG,1956,7.3

Madagascar,MDG,1957,7.3

Madagascar,MDG,1958,7.3

Madagascar,MDG,1959,7.3

Madagascar,MDG,1960,7.3

Madagascar,MDG,1961,7.301

Madagascar,MDG,1962,7.303

Madagascar,MDG,1963,7.305

Madagascar,MDG,1964,7.307

Madagascar,MDG,1965,7.308

Madagascar,MDG,1966,7.307

Madagascar,MDG,1967,7.304

Madagascar,MDG,1968,7.297

Madagascar,MDG,1969,7.286

Madagascar,MDG,1970,7.27

Madagascar,MDG,1971,7.248

Madagascar,MDG,1972,7.221

Madagascar,MDG,1973,7.188

Madagascar,MDG,1974,7.149

Madagascar,MDG,1975,7.102

Madagascar,MDG,1976,7.044

Madagascar,MDG,1977,6.976

Madagascar,MDG,1978,6.898

Madagascar,MDG,1979,6.813

Madagascar,MDG,1980,6.725

Madagascar,MDG,1981,6.637

Madagascar,MDG,1982,6.554

Madagascar,MDG,1983,6.477

Madagascar,MDG,1984,6.41

Madagascar,MDG,1985,6.353

Madagascar,MDG,1986,6.307

Madagascar,MDG,1987,6.27

Madagascar,MDG,1988,6.237

Madagascar,MDG,1989,6.208

Madagascar,MDG,1990,6.179

Madagascar,MDG,1991,6.149

Madagascar,MDG,1992,6.116

Madagascar,MDG,1993,6.078

Madagascar,MDG,1994,6.033

Madagascar,MDG,1995,5.979

Madagascar,MDG,1996,5.912

Madagascar,MDG,1997,5.835

Madagascar,MDG,1998,5.748

Madagascar,MDG,1999,5.653

Madagascar,MDG,2000,5.551

Madagascar,MDG,2001,5.447

Madagascar,MDG,2002,5.342

Madagascar,MDG,2003,5.24

Madagascar,MDG,2004,5.141

Madagascar,MDG,2005,5.047

Madagascar,MDG,2006,4.956

Madagascar,MDG,2007,4.866

Madagascar,MDG,2008,4.777

Madagascar,MDG,2009,4.689

Madagascar,MDG,2010,4.603

Madagascar,MDG,2011,4.52

Madagascar,MDG,2012,4.442

Madagascar,MDG,2013,4.37

Madagascar,MDG,2014,4.303

Madagascar,MDG,2015,4.241

Malawi,MWI,1950,6.841

Malawi,MWI,1951,6.842

Malawi,MWI,1952,6.846

Malawi,MWI,1953,6.852

Malawi,MWI,1954,6.859

Malawi,MWI,1955,6.867

Malawi,MWI,1956,6.877

Malawi,MWI,1957,6.889

Malawi,MWI,1958,6.903

Malawi,MWI,1959,6.92

Malawi,MWI,1960,6.94

Malawi,MWI,1961,6.964

Malawi,MWI,1962,6.991

Malawi,MWI,1963,7.023

Malawi,MWI,1964,7.058

Malawi,MWI,1965,7.096

Malawi,MWI,1966,7.137

Malawi,MWI,1967,7.179

Malawi,MWI,1968,7.221

Malawi,MWI,1969,7.262

Malawi,MWI,1970,7.303

Malawi,MWI,1971,7.345

Malawi,MWI,1972,7.389

Malawi,MWI,1973,7.432

Malawi,MWI,1974,7.476

Malawi,MWI,1975,7.517

Malawi,MWI,1976,7.556

Malawi,MWI,1977,7.59

Malawi,MWI,1978,7.618

Malawi,MWI,1979,7.636

Malawi,MWI,1980,7.643

Malawi,MWI,1981,7.637

Malawi,MWI,1982,7.618

Malawi,MWI,1983,7.585

Malawi,MWI,1984,7.538

Malawi,MWI,1985,7.474

Malawi,MWI,1986,7.392

Malawi,MWI,1987,7.291

Malawi,MWI,1988,7.176

Malawi,MWI,1989,7.051

Malawi,MWI,1990,6.922

Malawi,MWI,1991,6.798

Malawi,MWI,1992,6.683

Malawi,MWI,1993,6.582

Malawi,MWI,1994,6.495

Malawi,MWI,1995,6.424

Malawi,MWI,1996,6.364

Malawi,MWI,1997,6.31

Malawi,MWI,1998,6.257

Malawi,MWI,1999,6.203

Malawi,MWI,2000,6.149

Malawi,MWI,2001,6.097

Malawi,MWI,2002,6.049

Malawi,MWI,2003,6.003

Malawi,MWI,2004,5.958

Malawi,MWI,2005,5.903

Malawi,MWI,2006,5.83

Malawi,MWI,2007,5.733

Malawi,MWI,2008,5.611

Malawi,MWI,2009,5.467

Malawi,MWI,2010,5.308

Malawi,MWI,2011,5.146

Malawi,MWI,2012,4.992

Malawi,MWI,2013,4.856

Malawi,MWI,2014,4.74

Malawi,MWI,2015,4.646

Malaysia,MYS,1950,6.364

Malaysia,MYS,1951,6.356

Malaysia,MYS,1952,6.343

Malaysia,MYS,1953,6.34

Malaysia,MYS,1954,6.346

Malaysia,MYS,1955,6.361

Malaysia,MYS,1956,6.383

Malaysia,MYS,1957,6.41

Malaysia,MYS,1958,6.435

Malaysia,MYS,1959,6.452

Malaysia,MYS,1960,6.45

Malaysia,MYS,1961,6.42

Malaysia,MYS,1962,6.353

Malaysia,MYS,1963,6.246

Malaysia,MYS,1964,6.1

Malaysia,MYS,1965,5.923

Malaysia,MYS,1966,5.728

Malaysia,MYS,1967,5.529

Malaysia,MYS,1968,5.34

Malaysia,MYS,1969,5.167

Malaysia,MYS,1970,5.014

Malaysia,MYS,1971,4.88

Malaysia,MYS,1972,4.757

Malaysia,MYS,1973,4.64

Malaysia,MYS,1974,4.528

Malaysia,MYS,1975,4.425

Malaysia,MYS,1976,4.331

Malaysia,MYS,1977,4.249

Malaysia,MYS,1978,4.179

Malaysia,MYS,1979,4.12

Malaysia,MYS,1980,4.068

Malaysia,MYS,1981,4.021

Malaysia,MYS,1982,3.974

Malaysia,MYS,1983,3.925

Malaysia,MYS,1984,3.872

Malaysia,MYS,1985,3.816

Malaysia,MYS,1986,3.759

Malaysia,MYS,1987,3.703

Malaysia,MYS,1988,3.65

Malaysia,MYS,1989,3.6

Malaysia,MYS,1990,3.554

Malaysia,MYS,1991,3.51

Malaysia,MYS,1992,3.466

Malaysia,MYS,1993,3.422

Malaysia,MYS,1994,3.372

Malaysia,MYS,1995,3.313

Malaysia,MYS,1996,3.238

Malaysia,MYS,1997,3.145

Malaysia,MYS,1998,3.036

Malaysia,MYS,1999,2.913

Malaysia,MYS,2000,2.784

Malaysia,MYS,2001,2.657

Malaysia,MYS,2002,2.539

Malaysia,MYS,2003,2.438

Malaysia,MYS,2004,2.355

Malaysia,MYS,2005,2.293

Malaysia,MYS,2006,2.249

Malaysia,MYS,2007,2.217

Malaysia,MYS,2008,2.191

Malaysia,MYS,2009,2.169

Malaysia,MYS,2010,2.149

Malaysia,MYS,2011,2.129

Malaysia,MYS,2012,2.11

Malaysia,MYS,2013,2.092

Malaysia,MYS,2014,2.074

Malaysia,MYS,2015,2.056

Maldives,MDV,1950,5.595

Maldives,MDV,1951,5.715

Maldives,MDV,1952,5.943

Maldives,MDV,1953,6.149

Maldives,MDV,1954,6.335

Maldives,MDV,1955,6.499

Maldives,MDV,1956,6.642

Maldives,MDV,1957,6.764

Maldives,MDV,1958,6.867

Maldives,MDV,1959,6.952

Maldives,MDV,1960,7.021

Maldives,MDV,1961,7.075

Maldives,MDV,1962,7.118

Maldives,MDV,1963,7.152

Maldives,MDV,1964,7.179

Maldives,MDV,1965,7.201

Maldives,MDV,1966,7.218

Maldives,MDV,1967,7.231

Maldives,MDV,1968,7.237

Maldives,MDV,1969,7.237

Maldives,MDV,1970,7.227

Maldives,MDV,1971,7.201

Maldives,MDV,1972,7.159

Maldives,MDV,1973,7.103

Maldives,MDV,1974,7.04

Maldives,MDV,1975,6.981

Maldives,MDV,1976,6.944

Maldives,MDV,1977,6.936

Maldives,MDV,1978,6.961

Maldives,MDV,1979,7.013

Maldives,MDV,1980,7.081

Maldives,MDV,1981,7.151

Maldives,MDV,1982,7.203

Maldives,MDV,1983,7.219

Maldives,MDV,1984,7.189

Maldives,MDV,1985,7.106

Maldives,MDV,1986,6.965

Maldives,MDV,1987,6.776

Maldives,MDV,1988,6.548

Maldives,MDV,1989,6.286

Maldives,MDV,1990,5.993

Maldives,MDV,1991,5.674

Maldives,MDV,1992,5.336

Maldives,MDV,1993,4.987

Maldives,MDV,1994,4.637

Maldives,MDV,1995,4.298

Maldives,MDV,1996,3.975

Maldives,MDV,1997,3.674

Maldives,MDV,1998,3.399

Maldives,MDV,1999,3.155

Maldives,MDV,2000,2.944

Maldives,MDV,2001,2.769

Maldives,MDV,2002,2.625

Maldives,MDV,2003,2.508

Maldives,MDV,2004,2.415

Maldives,MDV,2005,2.344

Maldives,MDV,2006,2.294

Maldives,MDV,2007,2.262

Maldives,MDV,2008,2.244

Maldives,MDV,2009,2.235

Maldives,MDV,2010,2.229

Maldives,MDV,2011,2.221

Maldives,MDV,2012,2.209

Maldives,MDV,2013,2.191

Maldives,MDV,2014,2.165

Maldives,MDV,2015,2.132

Mali,MLI,1950,6.961

Mali,MLI,1951,6.957

Mali,MLI,1952,6.951

Mali,MLI,1953,6.946

Mali,MLI,1954,6.944

Mali,MLI,1955,6.943

Mali,MLI,1956,6.943

Mali,MLI,1957,6.946

Mali,MLI,1958,6.951

Mali,MLI,1959,6.958

Mali,MLI,1960,6.967

Mali,MLI,1961,6.979

Mali,MLI,1962,6.994

Mali,MLI,1963,7.011

Mali,MLI,1964,7.03

Mali,MLI,1965,7.05

Mali,MLI,1966,7.07

Mali,MLI,1967,7.089

Mali,MLI,1968,7.106

Mali,MLI,1969,7.121

Mali,MLI,1970,7.133

Mali,MLI,1971,7.141

Mali,MLI,1972,7.147

Mali,MLI,1973,7.151

Mali,MLI,1974,7.153

Mali,MLI,1975,7.154

Mali,MLI,1976,7.154

Mali,MLI,1977,7.153

Mali,MLI,1978,7.152

Mali,MLI,1979,7.151

Mali,MLI,1980,7.15

Mali,MLI,1981,7.15

Mali,MLI,1982,7.15

Mali,MLI,1983,7.15

Mali,MLI,1984,7.15

Mali,MLI,1985,7.151

Mali,MLI,1986,7.153

Mali,MLI,1987,7.156

Mali,MLI,1988,7.161

Mali,MLI,1989,7.164

Mali,MLI,1990,7.165

Mali,MLI,1991,7.159

Mali,MLI,1992,7.146

Mali,MLI,1993,7.124

Mali,MLI,1994,7.094

Mali,MLI,1995,7.058

Mali,MLI,1996,7.02

Mali,MLI,1997,6.983

Mali,MLI,1998,6.95

Mali,MLI,1999,6.921

Mali,MLI,2000,6.897

Mali,MLI,2001,6.878

Mali,MLI,2002,6.861

Mali,MLI,2003,6.842

Mali,MLI,2004,6.821

Mali,MLI,2005,6.794

Mali,MLI,2006,6.76

Mali,MLI,2007,6.719

Mali,MLI,2008,6.67

Mali,MLI,2009,6.612

Mali,MLI,2010,6.547

Mali,MLI,2011,6.474

Mali,MLI,2012,6.396

Mali,MLI,2013,6.315

Mali,MLI,2014,6.231

Mali,MLI,2015,6.145

Malta,MLT,1950,4.211

Malta,MLT,1951,4.197

Malta,MLT,1952,4.163

Malta,MLT,1953,4.122

Malta,MLT,1954,4.071

Malta,MLT,1955,4.011

Malta,MLT,1956,3.941

Malta,MLT,1957,3.861

Malta,MLT,1958,3.767

Malta,MLT,1959,3.657

Malta,MLT,1960,3.527

Malta,MLT,1961,3.375

Malta,MLT,1962,3.2

Malta,MLT,1963,3.005

Malta,MLT,1964,2.798

Malta,MLT,1965,2.592

Malta,MLT,1966,2.401

Malta,MLT,1967,2.238

Malta,MLT,1968,2.111

Malta,MLT,1969,2.023

Malta,MLT,1970,1.975

Malta,MLT,1971,1.964

Malta,MLT,1972,1.978

Malta,MLT,1973,2.004

Malta,MLT,1974,2.035

Malta,MLT,1975,2.063

Malta,MLT,1976,2.08

Malta,MLT,1977,2.085

Malta,MLT,1978,2.078

Malta,MLT,1979,2.059

Malta,MLT,1980,2.033

Malta,MLT,1981,2.004

Malta,MLT,1982,1.982

Malta,MLT,1983,1.97

Malta,MLT,1984,1.968

Malta,MLT,1985,1.977

Malta,MLT,1986,1.993

Malta,MLT,1987,2.009

Malta,MLT,1988,2.021

Malta,MLT,1989,2.026

Malta,MLT,1990,2.026

Malta,MLT,1991,2.021

Malta,MLT,1992,2.012

Malta,MLT,1993,2.001

Malta,MLT,1994,1.987

Malta,MLT,1995,1.966

Malta,MLT,1996,1.933

Malta,MLT,1997,1.886

Malta,MLT,1998,1.825

Malta,MLT,1999,1.753

Malta,MLT,2000,1.674

Malta,MLT,2001,1.597

Malta,MLT,2002,1.526

Malta,MLT,2003,1.467

Malta,MLT,2004,1.423

Malta,MLT,2005,1.395

Malta,MLT,2006,1.381

Malta,MLT,2007,1.376

Malta,MLT,2008,1.377

Malta,MLT,2009,1.381

Malta,MLT,2010,1.388

Malta,MLT,2011,1.396

Malta,MLT,2012,1.406

Malta,MLT,2013,1.417

Malta,MLT,2014,1.43

Malta,MLT,2015,1.443

Martinique,MTQ,1950,5.643

Martinique,MTQ,1951,5.665

Martinique,MTQ,1952,5.705

Martinique,MTQ,1953,5.733

Martinique,MTQ,1954,5.749

Martinique,MTQ,1955,5.753

Martinique,MTQ,1956,5.746

Martinique,MTQ,1957,5.729

Martinique,MTQ,1958,5.7

Martinique,MTQ,1959,5.663

Martinique,MTQ,1960,5.617

Martinique,MTQ,1961,5.564

Martinique,MTQ,1962,5.504

Martinique,MTQ,1963,5.437

Martinique,MTQ,1964,5.363

Martinique,MTQ,1965,5.278

Martinique,MTQ,1966,5.181

Martinique,MTQ,1967,5.069

Martinique,MTQ,1968,4.939

Martinique,MTQ,1969,4.789

Martinique,MTQ,1970,4.613

Martinique,MTQ,1971,4.404

Martinique,MTQ,1972,4.165

Martinique,MTQ,1973,3.9

Martinique,MTQ,1974,3.619

Martinique,MTQ,1975,3.333

Martinique,MTQ,1976,3.059

Martinique,MTQ,1977,2.809

Martinique,MTQ,1978,2.593

Martinique,MTQ,1979,2.418

Martinique,MTQ,1980,2.287

Martinique,MTQ,1981,2.202

Martinique,MTQ,1982,2.15

Martinique,MTQ,1983,2.123

Martinique,MTQ,1984,2.113

Martinique,MTQ,1985,2.111

Martinique,MTQ,1986,2.111

Martinique,MTQ,1987,2.108

Martinique,MTQ,1988,2.099

Martinique,MTQ,1989,2.081

Martinique,MTQ,1990,2.054

Martinique,MTQ,1991,2.021

Martinique,MTQ,1992,1.987

Martinique,MTQ,1993,1.958

Martinique,MTQ,1994,1.933

Martinique,MTQ,1995,1.916

Martinique,MTQ,1996,1.904

Martinique,MTQ,1997,1.897

Martinique,MTQ,1998,1.893

Martinique,MTQ,1999,1.893

Martinique,MTQ,2000,1.897

Martinique,MTQ,2001,1.908

Martinique,MTQ,2002,1.925

Martinique,MTQ,2003,1.948

Martinique,MTQ,2004,1.974

Martinique,MTQ,2005,2

Martinique,MTQ,2006,2.022

Martinique,MTQ,2007,2.036

Martinique,MTQ,2008,2.04

Martinique,MTQ,2009,2.035

Martinique,MTQ,2010,2.02

Martinique,MTQ,2011,1.999

Martinique,MTQ,2012,1.975

Martinique,MTQ,2013,1.952

Martinique,MTQ,2014,1.93

Martinique,MTQ,2015,1.913

Mauritania,MRT,1950,6.117

Mauritania,MRT,1951,6.179

Mauritania,MRT,1952,6.296

Mauritania,MRT,1953,6.4

Mauritania,MRT,1954,6.49

Mauritania,MRT,1955,6.568

Mauritania,MRT,1956,6.632

Mauritania,MRT,1957,6.685

Mauritania,MRT,1958,6.725

Mauritania,MRT,1959,6.755

Mauritania,MRT,1960,6.775

Mauritania,MRT,1961,6.787

Mauritania,MRT,1962,6.794

Mauritania,MRT,1963,6.798

Mauritania,MRT,1964,6.799

Mauritania,MRT,1965,6.799

Mauritania,MRT,1966,6.798

Mauritania,MRT,1967,6.798

Mauritania,MRT,1968,6.795

Mauritania,MRT,1969,6.791

Mauritania,MRT,1970,6.784

Mauritania,MRT,1971,6.773

Mauritania,MRT,1972,6.757

Mauritania,MRT,1973,6.736

Mauritania,MRT,1974,6.709

Mauritania,MRT,1975,6.676

Mauritania,MRT,1976,6.638

Mauritania,MRT,1977,6.596

Mauritania,MRT,1978,6.551

Mauritania,MRT,1979,6.504

Mauritania,MRT,1980,6.457

Mauritania,MRT,1981,6.411

Mauritania,MRT,1982,6.367

Mauritania,MRT,1983,6.324

Mauritania,MRT,1984,6.283

Mauritania,MRT,1985,6.244

Mauritania,MRT,1986,6.206

Mauritania,MRT,1987,6.168

Mauritania,MRT,1988,6.128

Mauritania,MRT,1989,6.085

Mauritania,MRT,1990,6.04

Mauritania,MRT,1991,5.991

Mauritania,MRT,1992,5.939

Mauritania,MRT,1993,5.883

Mauritania,MRT,1994,5.825

Mauritania,MRT,1995,5.765

Mauritania,MRT,1996,5.703

Mauritania,MRT,1997,5.64

Mauritania,MRT,1998,5.576

Mauritania,MRT,1999,5.514

Mauritania,MRT,2000,5.453

Mauritania,MRT,2001,5.394

Mauritania,MRT,2002,5.337

Mauritania,MRT,2003,5.283

Mauritania,MRT,2004,5.232

Mauritania,MRT,2005,5.183

Mauritania,MRT,2006,5.139

Mauritania,MRT,2007,5.097

Mauritania,MRT,2008,5.059

Mauritania,MRT,2009,5.021

Mauritania,MRT,2010,4.983

Mauritania,MRT,2011,4.942

Mauritania,MRT,2012,4.897

Mauritania,MRT,2013,4.848

Mauritania,MRT,2014,4.794

Mauritania,MRT,2015,4.736

Mauritius,MUS,1950,6.049

Mauritius,MUS,1951,5.993

Mauritius,MUS,1952,5.897

Mauritius,MUS,1953,5.838

Mauritius,MUS,1954,5.816

Mauritius,MUS,1955,5.828

Mauritius,MUS,1956,5.872

Mauritius,MUS,1957,5.942

Mauritius,MUS,1958,6.027

Mauritius,MUS,1959,6.109

Mauritius,MUS,1960,6.167

Mauritius,MUS,1961,6.178

Mauritius,MUS,1962,6.124

Mauritius,MUS,1963,5.993

Mauritius,MUS,1964,5.785

Mauritius,MUS,1965,5.51

Mauritius,MUS,1966,5.187

Mauritius,MUS,1967,4.847

Mauritius,MUS,1968,4.516

Mauritius,MUS,1969,4.213

Mauritius,MUS,1970,3.952

Mauritius,MUS,1971,3.741

Mauritius,MUS,1972,3.575

Mauritius,MUS,1973,3.444

Mauritius,MUS,1974,3.341

Mauritius,MUS,1975,3.255

Mauritius,MUS,1976,3.169

Mauritius,MUS,1977,3.071

Mauritius,MUS,1978,2.954

Mauritius,MUS,1979,2.818

Mauritius,MUS,1980,2.672

Mauritius,MUS,1981,2.532

Mauritius,MUS,1982,2.412

Mauritius,MUS,1983,2.325

Mauritius,MUS,1984,2.273

Mauritius,MUS,1985,2.252

Mauritius,MUS,1986,2.256

Mauritius,MUS,1987,2.272

Mauritius,MUS,1988,2.287

Mauritius,MUS,1989,2.296

Mauritius,MUS,1990,2.293

Mauritius,MUS,1991,2.278

Mauritius,MUS,1992,2.253

Mauritius,MUS,1993,2.221

Mauritius,MUS,1994,2.183

Mauritius,MUS,1995,2.143

Mauritius,MUS,1996,2.104

Mauritius,MUS,1997,2.067

Mauritius,MUS,1998,2.036

Mauritius,MUS,1999,2.009

Mauritius,MUS,2000,1.985

Mauritius,MUS,2001,1.962

Mauritius,MUS,2002,1.936

Mauritius,MUS,2003,1.904

Mauritius,MUS,2004,1.867

Mauritius,MUS,2005,1.823

Mauritius,MUS,2006,1.775

Mauritius,MUS,2007,1.724

Mauritius,MUS,2008,1.673

Mauritius,MUS,2009,1.623

Mauritius,MUS,2010,1.578

Mauritius,MUS,2011,1.539

Mauritius,MUS,2012,1.505

Mauritius,MUS,2013,1.478

Mauritius,MUS,2014,1.458

Mauritius,MUS,2015,1.443

Mayotte,MYT,1950,7.91

Mayotte,MYT,1951,7.91

Mayotte,MYT,1952,7.91

Mayotte,MYT,1953,7.91

Mayotte,MYT,1954,7.91

Mayotte,MYT,1955,7.91

Mayotte,MYT,1956,7.91

Mayotte,MYT,1957,7.91

Mayotte,MYT,1958,7.91

Mayotte,MYT,1959,7.91

Mayotte,MYT,1960,7.91

Mayotte,MYT,1961,7.91

Mayotte,MYT,1962,7.91

Mayotte,MYT,1963,7.91

Mayotte,MYT,1964,7.91

Mayotte,MYT,1965,7.91

Mayotte,MYT,1966,7.91

Mayotte,MYT,1967,7.91

Mayotte,MYT,1968,7.91

Mayotte,MYT,1969,7.91

Mayotte,MYT,1970,7.912

Mayotte,MYT,1971,7.918

Mayotte,MYT,1972,7.928

Mayotte,MYT,1973,7.94

Mayotte,MYT,1974,7.95

Mayotte,MYT,1975,7.953

Mayotte,MYT,1976,7.941

Mayotte,MYT,1977,7.909

Mayotte,MYT,1978,7.854

Mayotte,MYT,1979,7.777

Mayotte,MYT,1980,7.681

Mayotte,MYT,1981,7.574

Mayotte,MYT,1982,7.463

Mayotte,MYT,1983,7.351

Mayotte,MYT,1984,7.236

Mayotte,MYT,1985,7.107

Mayotte,MYT,1986,6.946

Mayotte,MYT,1987,6.746

Mayotte,MYT,1988,6.507

Mayotte,MYT,1989,6.236

Mayotte,MYT,1990,5.954

Mayotte,MYT,1991,5.686

Mayotte,MYT,1992,5.454

Mayotte,MYT,1993,5.273

Mayotte,MYT,1994,5.149

Mayotte,MYT,1995,5.076

Mayotte,MYT,1996,5.041

Mayotte,MYT,1997,5.024

Mayotte,MYT,1998,5.005

Mayotte,MYT,1999,4.978

Mayotte,MYT,2000,4.939

Mayotte,MYT,2001,4.892

Mayotte,MYT,2002,4.844

Mayotte,MYT,2003,4.8

Mayotte,MYT,2004,4.759

Mayotte,MYT,2005,4.717

Mayotte,MYT,2006,4.669

Mayotte,MYT,2007,4.61

Mayotte,MYT,2008,4.539

Mayotte,MYT,2009,4.456

Mayotte,MYT,2010,4.363

Mayotte,MYT,2011,4.264

Mayotte,MYT,2012,4.165

Mayotte,MYT,2013,4.07

Mayotte,MYT,2014,3.981

Mayotte,MYT,2015,3.9

Melanesia,OWID_MNS,1950,6.252

Melanesia,OWID_MNS,1951,6.268

Melanesia,OWID_MNS,1952,6.296

Melanesia,OWID_MNS,1953,6.319

Melanesia,OWID_MNS,1954,6.336

Melanesia,OWID_MNS,1955,6.347

Melanesia,OWID_MNS,1956,6.351

Melanesia,OWID_MNS,1957,6.35

Melanesia,OWID_MNS,1958,6.343

Melanesia,OWID_MNS,1959,6.33

Melanesia,OWID_MNS,1960,6.31

Melanesia,OWID_MNS,1961,6.285

Melanesia,OWID_MNS,1962,6.254

Melanesia,OWID_MNS,1963,6.218

Melanesia,OWID_MNS,1964,6.179

Melanesia,OWID_MNS,1965,6.137

Melanesia,OWID_MNS,1966,6.093

Melanesia,OWID_MNS,1967,6.049

Melanesia,OWID_MNS,1968,6.006

Melanesia,OWID_MNS,1969,5.964

Melanesia,OWID_MNS,1970,5.922

Melanesia,OWID_MNS,1971,5.881

Melanesia,OWID_MNS,1972,5.839

Melanesia,OWID_MNS,1973,5.796

Melanesia,OWID_MNS,1974,5.75

Melanesia,OWID_MNS,1975,5.701

Melanesia,OWID_MNS,1976,5.648

Melanesia,OWID_MNS,1977,5.59

Melanesia,OWID_MNS,1978,5.528

Melanesia,OWID_MNS,1979,5.461

Melanesia,OWID_MNS,1980,5.388

Melanesia,OWID_MNS,1981,5.309

Melanesia,OWID_MNS,1982,5.224

Melanesia,OWID_MNS,1983,5.135

Melanesia,OWID_MNS,1984,5.044

Melanesia,OWID_MNS,1985,4.953

Melanesia,OWID_MNS,1986,4.866

Melanesia,OWID_MNS,1987,4.785

Melanesia,OWID_MNS,1988,4.711

Melanesia,OWID_MNS,1989,4.647

Melanesia,OWID_MNS,1990,4.593

Melanesia,OWID_MNS,1991,4.55

Melanesia,OWID_MNS,1992,4.516

Melanesia,OWID_MNS,1993,4.49

Melanesia,OWID_MNS,1994,4.468

Melanesia,OWID_MNS,1995,4.448

Melanesia,OWID_MNS,1996,4.426

Melanesia,OWID_MNS,1997,4.4

Melanesia,OWID_MNS,1998,4.369

Melanesia,OWID_MNS,1999,4.332

Melanesia,OWID_MNS,2000,4.289

Melanesia,OWID_MNS,2001,4.242

Melanesia,OWID_MNS,2002,4.193

Melanesia,OWID_MNS,2003,4.143

Melanesia,OWID_MNS,2004,4.095

Melanesia,OWID_MNS,2005,4.047

Melanesia,OWID_MNS,2006,4

Melanesia,OWID_MNS,2007,3.952

Melanesia,OWID_MNS,2008,3.903

Melanesia,OWID_MNS,2009,3.852

Melanesia,OWID_MNS,2010,3.801

Melanesia,OWID_MNS,2011,3.75

Melanesia,OWID_MNS,2012,3.699

Melanesia,OWID_MNS,2013,3.65

Melanesia,OWID_MNS,2014,3.603

Melanesia,OWID_MNS,2015,3.557

Mexico,MEX,1950,6.72

Mexico,MEX,1951,6.73

Mexico,MEX,1952,6.746

Mexico,MEX,1953,6.759

Mexico,MEX,1954,6.769

Mexico,MEX,1955,6.775

Mexico,MEX,1956,6.779

Mexico,MEX,1957,6.779

Mexico,MEX,1958,6.777

Mexico,MEX,1959,6.773

Mexico,MEX,1960,6.768

Mexico,MEX,1961,6.762

Mexico,MEX,1962,6.758

Mexico,MEX,1963,6.754

Mexico,MEX,1964,6.752

Mexico,MEX,1965,6.755

Mexico,MEX,1966,6.768

Mexico,MEX,1967,6.789

Mexico,MEX,1968,6.813

Mexico,MEX,1969,6.831

Mexico,MEX,1970,6.83

Mexico,MEX,1971,6.789

Mexico,MEX,1972,6.7

Mexico,MEX,1973,6.558

Mexico,MEX,1974,6.366

Mexico,MEX,1975,6.13

Mexico,MEX,1976,5.864

Mexico,MEX,1977,5.587

Mexico,MEX,1978,5.317

Mexico,MEX,1979,5.064

Mexico,MEX,1980,4.836

Mexico,MEX,1981,4.635

Mexico,MEX,1982,4.457

Mexico,MEX,1983,4.296

Mexico,MEX,1984,4.151

Mexico,MEX,1985,4.019

Mexico,MEX,1986,3.899

Mexico,MEX,1987,3.786

Mexico,MEX,1988,3.678

Mexico,MEX,1989,3.573

Mexico,MEX,1990,3.47

Mexico,MEX,1991,3.369

Mexico,MEX,1992,3.273

Mexico,MEX,1993,3.182

Mexico,MEX,1994,3.097

Mexico,MEX,1995,3.018

Mexico,MEX,1996,2.946

Mexico,MEX,1997,2.88

Mexico,MEX,1998,2.821

Mexico,MEX,1999,2.766

Mexico,MEX,2000,2.716

Mexico,MEX,2001,2.669

Mexico,MEX,2002,2.623

Mexico,MEX,2003,2.579

Mexico,MEX,2004,2.536

Mexico,MEX,2005,2.495

Mexico,MEX,2006,2.456

Mexico,MEX,2007,2.421

Mexico,MEX,2008,2.391

Mexico,MEX,2009,2.364

Mexico,MEX,2010,2.341

Mexico,MEX,2011,2.318

Mexico,MEX,2012,2.295

Mexico,MEX,2013,2.27

Mexico,MEX,2014,2.243

Mexico,MEX,2015,2.215

Micronesia (country),FSM,1950,7.303

Micronesia (country),FSM,1951,7.275

Micronesia (country),FSM,1952,7.222

Micronesia (country),FSM,1953,7.172

Micronesia (country),FSM,1954,7.127

Micronesia (country),FSM,1955,7.085

Micronesia (country),FSM,1956,7.046

Micronesia (country),FSM,1957,7.012

Micronesia (country),FSM,1958,6.982

Micronesia (country),FSM,1959,6.956

Micronesia (country),FSM,1960,6.934

Micronesia (country),FSM,1961,6.917

Micronesia (country),FSM,1962,6.905

Micronesia (country),FSM,1963,6.897

Micronesia (country),FSM,1964,6.893

Micronesia (country),FSM,1965,6.894

Micronesia (country),FSM,1966,6.9

Micronesia (country),FSM,1967,6.911

Micronesia (country),FSM,1968,6.924

Micronesia (country),FSM,1969,6.935

Micronesia (country),FSM,1970,6.938

Micronesia (country),FSM,1971,6.925

Micronesia (country),FSM,1972,6.894

Micronesia (country),FSM,1973,6.842

Micronesia (country),FSM,1974,6.77

Micronesia (country),FSM,1975,6.682

Micronesia (country),FSM,1976,6.586

Micronesia (country),FSM,1977,6.49

Micronesia (country),FSM,1978,6.398

Micronesia (country),FSM,1979,6.311

Micronesia (country),FSM,1980,6.223

Micronesia (country),FSM,1981,6.129

Micronesia (country),FSM,1982,6.02

Micronesia (country),FSM,1983,5.894

Micronesia (country),FSM,1984,5.752

Micronesia (country),FSM,1985,5.6

Micronesia (country),FSM,1986,5.446

Micronesia (country),FSM,1987,5.299

Micronesia (country),FSM,1988,5.167

Micronesia (country),FSM,1989,5.052

Micronesia (country),FSM,1990,4.958

Micronesia (country),FSM,1991,4.882

Micronesia (country),FSM,1992,4.82

Micronesia (country),FSM,1993,4.766

Micronesia (country),FSM,1994,4.716

Micronesia (country),FSM,1995,4.664

Micronesia (country),FSM,1996,4.608

Micronesia (country),FSM,1997,4.544

Micronesia (country),FSM,1998,4.471

Micronesia (country),FSM,1999,4.39

Micronesia (country),FSM,2000,4.3

Micronesia (country),FSM,2001,4.204

Micronesia (country),FSM,2002,4.105

Micronesia (country),FSM,2003,4.007

Micronesia (country),FSM,2004,3.912

Micronesia (country),FSM,2005,3.823

Micronesia (country),FSM,2006,3.739

Micronesia (country),FSM,2007,3.661

Micronesia (country),FSM,2008,3.589

Micronesia (country),FSM,2009,3.522

Micronesia (country),FSM,2010,3.46

Micronesia (country),FSM,2011,3.402

Micronesia (country),FSM,2012,3.347

Micronesia (country),FSM,2013,3.294

Micronesia (country),FSM,2014,3.243

Micronesia (country),FSM,2015,3.194

Micronesia (region),,1950,6.173

Micronesia (region),,1951,6.193

Micronesia (region),,1952,6.232

Micronesia (region),,1953,6.272

Micronesia (region),,1954,6.313

Micronesia (region),,1955,6.354

Micronesia (region),,1956,6.396

Micronesia (region),,1957,6.435

Micronesia (region),,1958,6.47

Micronesia (region),,1959,6.495

Micronesia (region),,1960,6.506

Micronesia (region),,1961,6.496

Micronesia (region),,1962,6.46

Micronesia (region),,1963,6.397

Micronesia (region),,1964,6.308

Micronesia (region),,1965,6.196

Micronesia (region),,1966,6.068

Micronesia (region),,1967,5.932

Micronesia (region),,1968,5.797

Micronesia (region),,1969,5.668

Micronesia (region),,1970,5.546

Micronesia (region),,1971,5.433

Micronesia (region),,1972,5.326

Micronesia (region),,1973,5.223

Micronesia (region),,1974,5.123

Micronesia (region),,1975,5.027

Micronesia (region),,1976,4.933

Micronesia (region),,1977,4.843

Micronesia (region),,1978,4.755

Micronesia (region),,1979,4.67

Micronesia (region),,1980,4.588

Micronesia (region),,1981,4.511

Micronesia (region),,1982,4.438

Micronesia (region),,1983,4.369

Micronesia (region),,1984,4.304

Micronesia (region),,1985,4.241

Micronesia (region),,1986,4.179

Micronesia (region),,1987,4.116

Micronesia (region),,1988,4.052

Micronesia (region),,1989,3.986

Micronesia (region),,1990,3.917

Micronesia (region),,1991,3.849

Micronesia (region),,1992,3.781

Micronesia (region),,1993,3.715

Micronesia (region),,1994,3.651

Micronesia (region),,1995,3.589

Micronesia (region),,1996,3.527

Micronesia (region),,1997,3.465

Micronesia (region),,1998,3.403

Micronesia (region),,1999,3.341

Micronesia (region),,2000,3.282

Micronesia (region),,2001,3.227

Micronesia (region),,2002,3.179

Micronesia (region),,2003,3.138

Micronesia (region),,2004,3.106

Micronesia (region),,2005,3.081

Micronesia (region),,2006,3.064

Micronesia (region),,2007,3.053

Micronesia (region),,2008,3.045

Micronesia (region),,2009,3.038

Micronesia (region),,2010,3.03

Micronesia (region),,2011,3.019

Micronesia (region),,2012,3.005

Micronesia (region),,2013,2.986

Micronesia (region),,2014,2.962

Micronesia (region),,2015,2.932

Middle Africa,,1950,6.087

Middle Africa,,1951,6.082

Middle Africa,,1952,6.074

Middle Africa,,1953,6.07

Middle Africa,,1954,6.072

Middle Africa,,1955,6.078

Middle Africa,,1956,6.089

Middle Africa,,1957,6.105

Middle Africa,,1958,6.124

Middle Africa,,1959,6.147

Middle Africa,,1960,6.172

Middle Africa,,1961,6.198

Middle Africa,,1962,6.224

Middle Africa,,1963,6.25

Middle Africa,,1964,6.274

Middle Africa,,1965,6.298

Middle Africa,,1966,6.32

Middle Africa,,1967,6.344

Middle Africa,,1968,6.369

Middle Africa,,1969,6.395

Middle Africa,,1970,6.423

Middle Africa,,1971,6.453

Middle Africa,,1972,6.484

Middle Africa,,1973,6.516

Middle Africa,,1974,6.548

Middle Africa,,1975,6.58

Middle Africa,,1976,6.61

Middle Africa,,1977,6.637

Middle Africa,,1978,6.662

Middle Africa,,1979,6.684

Middle Africa,,1980,6.702

Middle Africa,,1981,6.718

Middle Africa,,1982,6.732

Middle Africa,,1983,6.743

Middle Africa,,1984,6.752

Middle Africa,,1985,6.759

Middle Africa,,1986,6.761

Middle Africa,,1987,6.76

Middle Africa,,1988,6.754

Middle Africa,,1989,6.744

Middle Africa,,1990,6.729

Middle Africa,,1991,6.71

Middle Africa,,1992,6.687

Middle Africa,,1993,6.661

Middle Africa,,1994,6.633

Middle Africa,,1995,6.603

Middle Africa,,1996,6.573

Middle Africa,,1997,6.544

Middle Africa,,1998,6.514

Middle Africa,,1999,6.486

Middle Africa,,2000,6.458

Middle Africa,,2001,6.432

Middle Africa,,2002,6.407

Middle Africa,,2003,6.381

Middle Africa,,2004,6.354

Middle Africa,,2005,6.325

Middle Africa,,2006,6.292

Middle Africa,,2007,6.254

Middle Africa,,2008,6.211

Middle Africa,,2009,6.162

Middle Africa,,2010,6.106

Middle Africa,,2011,6.044

Middle Africa,,2012,5.976

Middle Africa,,2013,5.904

Middle Africa,,2014,5.828

Middle Africa,,2015,5.749

Middle-income countries,,1950,5.804

Middle-income countries,,1951,5.752

Middle-income countries,,1952,5.658

Middle-income countries,,1953,5.585

Middle-income countries,,1954,5.532

Middle-income countries,,1955,5.499

Middle-income countries,,1956,5.485

Middle-income countries,,1957,5.49

Middle-income countries,,1958,5.511

Middle-income countries,,1959,5.545

Middle-income countries,,1960,5.587

Middle-income countries,,1961,5.631

Middle-income countries,,1962,5.671

Middle-income countries,,1963,5.702

Middle-income countries,,1964,5.717

Middle-income countries,,1965,5.712

Middle-income countries,,1966,5.683

Middle-income countries,,1967,5.633

Middle-income countries,,1968,5.563

Middle-income countries,,1969,5.473

Middle-income countries,,1970,5.364

Middle-income countries,,1971,5.236

Middle-income countries,,1972,5.092

Middle-income countries,,1973,4.937

Middle-income countries,,1974,4.777

Middle-income countries,,1975,4.619

Middle-income countries,,1976,4.47

Middle-income countries,,1977,4.332

Middle-income countries,,1978,4.21

Middle-income countries,,1979,4.105

Middle-income countries,,1980,4.019

Middle-income countries,,1981,3.952

Middle-income countries,,1982,3.9

Middle-income countries,,1983,3.856

Middle-income countries,,1984,3.815

Middle-income countries,,1985,3.771

Middle-income countries,,1986,3.718

Middle-income countries,,1987,3.651

Middle-income countries,,1988,3.569

Middle-income countries,,1989,3.474

Middle-income countries,,1990,3.367

Middle-income countries,,1991,3.254

Middle-income countries,,1992,3.142

Middle-income countries,,1993,3.037

Middle-income countries,,1994,2.942

Middle-income countries,,1995,2.861

Middle-income countries,,1996,2.794

Middle-income countries,,1997,2.737

Middle-income countries,,1998,2.689

Middle-income countries,,1999,2.649

Middle-income countries,,2000,2.615

Middle-income countries,,2001,2.586

Middle-income countries,,2002,2.562

Middle-income countries,,2003,2.54

Middle-income countries,,2004,2.52

Middle-income countries,,2005,2.501

Middle-income countries,,2006,2.483

Middle-income countries,,2007,2.466

Middle-income countries,,2008,2.452

Middle-income countries,,2009,2.438

Middle-income countries,,2010,2.426

Middle-income countries,,2011,2.414

Middle-income countries,,2012,2.403

Middle-income countries,,2013,2.392

Middle-income countries,,2014,2.381

Middle-income countries,,2015,2.371

Moldova,MDA,1950,3.475

Moldova,MDA,1951,3.485

Moldova,MDA,1952,3.502

Moldova,MDA,1953,3.51

Moldova,MDA,1954,3.511

Moldova,MDA,1955,3.504

Moldova,MDA,1956,3.488

Moldova,MDA,1957,3.463

Moldova,MDA,1958,3.429

Moldova,MDA,1959,3.384

Moldova,MDA,1960,3.328

Moldova,MDA,1961,3.259

Moldova,MDA,1962,3.178

Moldova,MDA,1963,3.086

Moldova,MDA,1964,2.989

Moldova,MDA,1965,2.891

Moldova,MDA,1966,2.801

Moldova,MDA,1967,2.722

Moldova,MDA,1968,2.66

Moldova,MDA,1969,2.613

Moldova,MDA,1970,2.582

Moldova,MDA,1971,2.56

Moldova,MDA,1972,2.542

Moldova,MDA,1973,2.523

Moldova,MDA,1974,2.503

Moldova,MDA,1975,2.483

Moldova,MDA,1976,2.465

Moldova,MDA,1977,2.455

Moldova,MDA,1978,2.454

Moldova,MDA,1979,2.462

Moldova,MDA,1980,2.481

Moldova,MDA,1981,2.51

Moldova,MDA,1982,2.546

Moldova,MDA,1983,2.585

Moldova,MDA,1984,2.62

Moldova,MDA,1985,2.643

Moldova,MDA,1986,2.646

Moldova,MDA,1987,2.625

Moldova,MDA,1988,2.578

Moldova,MDA,1989,2.506

Moldova,MDA,1990,2.414

Moldova,MDA,1991,2.309

Moldova,MDA,1992,2.199

Moldova,MDA,1993,2.093

Moldova,MDA,1994,1.994

Moldova,MDA,1995,1.901

Moldova,MDA,1996,1.811

Moldova,MDA,1997,1.719

Moldova,MDA,1998,1.623

Moldova,MDA,1999,1.527

Moldova,MDA,2000,1.435

Moldova,MDA,2001,1.354

Moldova,MDA,2002,1.291

Moldova,MDA,2003,1.247

Moldova,MDA,2004,1.224

Moldova,MDA,2005,1.219

Moldova,MDA,2006,1.227

Moldova,MDA,2007,1.242

Moldova,MDA,2008,1.256

Moldova,MDA,2009,1.268

Moldova,MDA,2010,1.274

Moldova,MDA,2011,1.273

Moldova,MDA,2012,1.269

Moldova,MDA,2013,1.263

Moldova,MDA,2014,1.256

Moldova,MDA,2015,1.248

Mongolia,MNG,1950,5.497

Mongolia,MNG,1951,5.504

Mongolia,MNG,1952,5.537

Mongolia,MNG,1953,5.607

Mongolia,MNG,1954,5.714

Mongolia,MNG,1955,5.859

Mongolia,MNG,1956,6.037

Mongolia,MNG,1957,6.245

Mongolia,MNG,1958,6.476

Mongolia,MNG,1959,6.718

Mongolia,MNG,1960,6.953

Mongolia,MNG,1961,7.168

Mongolia,MNG,1962,7.347

Mongolia,MNG,1963,7.477

Mongolia,MNG,1964,7.558

Mongolia,MNG,1965,7.593

Mongolia,MNG,1966,7.599

Mongolia,MNG,1967,7.591

Mongolia,MNG,1968,7.584

Mongolia,MNG,1969,7.578

Mongolia,MNG,1970,7.569

Mongolia,MNG,1971,7.546

Mongolia,MNG,1972,7.497

Mongolia,MNG,1973,7.413

Mongolia,MNG,1974,7.294

Mongolia,MNG,1975,7.144

Mongolia,MNG,1976,6.969

Mongolia,MNG,1977,6.78

Mongolia,MNG,1978,6.587

Mongolia,MNG,1979,6.395

Mongolia,MNG,1980,6.209

Mongolia,MNG,1981,6.033

Mongolia,MNG,1982,5.864

Mongolia,MNG,1983,5.696

Mongolia,MNG,1984,5.526

Mongolia,MNG,1985,5.345

Mongolia,MNG,1986,5.14

Mongolia,MNG,1987,4.906

Mongolia,MNG,1988,4.644

Mongolia,MNG,1989,4.356

Mongolia,MNG,1990,4.052

Mongolia,MNG,1991,3.746

Mongolia,MNG,1992,3.451

Mongolia,MNG,1993,3.18

Mongolia,MNG,1994,2.941

Mongolia,MNG,1995,2.738

Mongolia,MNG,1996,2.57

Mongolia,MNG,1997,2.43

Mongolia,MNG,1998,2.312

Mongolia,MNG,1999,2.216

Mongolia,MNG,2000,2.143

Mongolia,MNG,2001,2.096

Mongolia,MNG,2002,2.076

Mongolia,MNG,2003,2.083

Mongolia,MNG,2004,2.112

Mongolia,MNG,2005,2.165

Mongolia,MNG,2006,2.239

Mongolia,MNG,2007,2.331

Mongolia,MNG,2008,2.434

Mongolia,MNG,2009,2.539

Mongolia,MNG,2010,2.637

Mongolia,MNG,2011,2.716

Mongolia,MNG,2012,2.774

Mongolia,MNG,2013,2.805

Mongolia,MNG,2014,2.811

Mongolia,MNG,2015,2.793

Montenegro,MNE,1950,4.621

Montenegro,MNE,1951,4.547

Montenegro,MNE,1952,4.404

Montenegro,MNE,1953,4.272

Montenegro,MNE,1954,4.151

Montenegro,MNE,1955,4.04

Montenegro,MNE,1956,3.939

Montenegro,MNE,1957,3.847

Montenegro,MNE,1958,3.762

Montenegro,MNE,1959,3.682

Montenegro,MNE,1960,3.603

Montenegro,MNE,1961,3.522

Montenegro,MNE,1962,3.436

Montenegro,MNE,1963,3.344

Montenegro,MNE,1964,3.246

Montenegro,MNE,1965,3.145

Montenegro,MNE,1966,3.046

Montenegro,MNE,1967,2.953

Montenegro,MNE,1968,2.871

Montenegro,MNE,1969,2.8

Montenegro,MNE,1970,2.737

Montenegro,MNE,1971,2.68

Montenegro,MNE,1972,2.624

Montenegro,MNE,1973,2.567

Montenegro,MNE,1974,2.507

Montenegro,MNE,1975,2.447

Montenegro,MNE,1976,2.391

Montenegro,MNE,1977,2.341

Montenegro,MNE,1978,2.3

Montenegro,MNE,1979,2.268

Montenegro,MNE,1980,2.243

Montenegro,MNE,1981,2.224

Montenegro,MNE,1982,2.208

Montenegro,MNE,1983,2.192

Montenegro,MNE,1984,2.175

Montenegro,MNE,1985,2.156

Montenegro,MNE,1986,2.137

Montenegro,MNE,1987,2.12

Montenegro,MNE,1988,2.104

Montenegro,MNE,1989,2.091

Montenegro,MNE,1990,2.078

Montenegro,MNE,1991,2.063

Montenegro,MNE,1992,2.046

Montenegro,MNE,1993,2.025

Montenegro,MNE,1994,2.002

Montenegro,MNE,1995,1.977

Montenegro,MNE,1996,1.951

Montenegro,MNE,1997,1.927

Montenegro,MNE,1998,1.906

Montenegro,MNE,1999,1.888

Montenegro,MNE,2000,1.875

Montenegro,MNE,2001,1.865

Montenegro,MNE,2002,1.858

Montenegro,MNE,2003,1.852

Montenegro,MNE,2004,1.847

Montenegro,MNE,2005,1.84

Montenegro,MNE,2006,1.832

Montenegro,MNE,2007,1.82

Montenegro,MNE,2008,1.805

Montenegro,MNE,2009,1.787

Montenegro,MNE,2010,1.767

Montenegro,MNE,2011,1.745

Montenegro,MNE,2012,1.725

Montenegro,MNE,2013,1.706

Montenegro,MNE,2014,1.69

Montenegro,MNE,2015,1.677

More developed regions,,1950,2.807

More developed regions,,1951,2.812

More developed regions,,1952,2.82

More developed regions,,1953,2.824

More developed regions,,1954,2.825

More developed regions,,1955,2.822

More developed regions,,1956,2.815

More developed regions,,1957,2.805

More developed regions,,1958,2.79

More developed regions,,1959,2.771

More developed regions,,1960,2.745

More developed regions,,1961,2.714

More developed regions,,1962,2.676

More developed regions,,1963,2.632

More developed regions,,1964,2.582

More developed regions,,1965,2.529

More developed regions,,1966,2.474

More developed regions,,1967,2.42

More developed regions,,1968,2.366

More developed regions,,1969,2.316

More developed regions,,1970,2.267

More developed regions,,1971,2.219

More developed regions,,1972,2.171

More developed regions,,1973,2.122

More developed regions,,1974,2.073

More developed regions,,1975,2.026

More developed regions,,1976,1.982

More developed regions,,1977,1.943

More developed regions,,1978,1.911

More developed regions,,1979,1.886

More developed regions,,1980,1.867

More developed regions,,1981,1.855

More developed regions,,1982,1.847

More developed regions,,1983,1.841

More developed regions,,1984,1.837

More developed regions,,1985,1.831

More developed regions,,1986,1.822

More developed regions,,1987,1.809

More developed regions,,1988,1.791

More developed regions,,1989,1.77

More developed regions,,1990,1.744

More developed regions,,1991,1.715

More developed regions,,1992,1.686

More developed regions,,1993,1.657

More developed regions,,1994,1.63

More developed regions,,1995,1.607

More developed regions,,1996,1.587

More developed regions,,1997,1.573

More developed regions,,1998,1.563

More developed regions,,1999,1.557

More developed regions,,2000,1.557

More developed regions,,2001,1.563

More developed regions,,2002,1.574

More developed regions,,2003,1.588

More developed regions,,2004,1.605

More developed regions,,2005,1.623

More developed regions,,2006,1.639

More developed regions,,2007,1.653

More developed regions,,2008,1.664

More developed regions,,2009,1.67

More developed regions,,2010,1.673

More developed regions,,2011,1.674

More developed regions,,2012,1.674

More developed regions,,2013,1.674

More developed regions,,2014,1.676

More developed regions,,2015,1.679

Morocco,MAR,1950,6.485

Morocco,MAR,1951,6.516

Morocco,MAR,1952,6.577

Morocco,MAR,1953,6.639

Morocco,MAR,1954,6.701

Morocco,MAR,1955,6.763

Morocco,MAR,1956,6.825

Morocco,MAR,1957,6.886

Morocco,MAR,1958,6.944

Morocco,MAR,1959,6.997

Morocco,MAR,1960,7.04

Morocco,MAR,1961,7.072

Morocco,MAR,1962,7.088

Morocco,MAR,1963,7.086

Morocco,MAR,1964,7.065

Morocco,MAR,1965,7.026

Morocco,MAR,1966,6.969

Morocco,MAR,1967,6.9

Morocco,MAR,1968,6.821

Morocco,MAR,1969,6.736

Morocco,MAR,1970,6.646

Morocco,MAR,1971,6.552

Morocco,MAR,1972,6.455

Morocco,MAR,1973,6.356

Morocco,MAR,1974,6.255

Morocco,MAR,1975,6.156

Morocco,MAR,1976,6.06

Morocco,MAR,1977,5.967

Morocco,MAR,1978,5.876

Morocco,MAR,1979,5.783

Morocco,MAR,1980,5.684

Morocco,MAR,1981,5.571

Morocco,MAR,1982,5.441

Morocco,MAR,1983,5.291

Morocco,MAR,1984,5.123

Morocco,MAR,1985,4.942

Morocco,MAR,1986,4.754

Morocco,MAR,1987,4.567

Morocco,MAR,1988,4.387

Morocco,MAR,1989,4.217

Morocco,MAR,1990,4.057

Morocco,MAR,1991,3.903

Morocco,MAR,1992,3.75

Morocco,MAR,1993,3.597

Morocco,MAR,1994,3.445

Morocco,MAR,1995,3.298

Morocco,MAR,1996,3.161

Morocco,MAR,1997,3.038

Morocco,MAR,1998,2.933

Morocco,MAR,1999,2.846

Morocco,MAR,2000,2.777

Morocco,MAR,2001,2.723

Morocco,MAR,2002,2.68

Morocco,MAR,2003,2.644

Morocco,MAR,2004,2.613

Morocco,MAR,2005,2.588

Morocco,MAR,2006,2.571

Morocco,MAR,2007,2.563

Morocco,MAR,2008,2.563

Morocco,MAR,2009,2.569

Morocco,MAR,2010,2.578

Morocco,MAR,2011,2.584

Morocco,MAR,2012,2.583

Morocco,MAR,2013,2.574

Morocco,MAR,2014,2.555

Morocco,MAR,2015,2.526

Mozambique,MOZ,1950,6.972

Mozambique,MOZ,1951,6.981

Mozambique,MOZ,1952,6.998

Mozambique,MOZ,1953,7.01

Mozambique,MOZ,1954,7.016

Mozambique,MOZ,1955,7.017

Mozambique,MOZ,1956,7.012

Mozambique,MOZ,1957,7.003

Mozambique,MOZ,1958,6.99

Mozambique,MOZ,1959,6.973

Mozambique,MOZ,1960,6.954

Mozambique,MOZ,1961,6.935

Mozambique,MOZ,1962,6.916

Mozambique,MOZ,1963,6.898

Mozambique,MOZ,1964,6.884

Mozambique,MOZ,1965,6.872

Mozambique,MOZ,1966,6.864

Mozambique,MOZ,1967,6.859

Mozambique,MOZ,1968,6.854

Mozambique,MOZ,1969,6.849

Mozambique,MOZ,1970,6.84

Mozambique,MOZ,1971,6.824

Mozambique,MOZ,1972,6.801

Mozambique,MOZ,1973,6.769

Mozambique,MOZ,1974,6.729

Mozambique,MOZ,1975,6.683

Mozambique,MOZ,1976,6.633

Mozambique,MOZ,1977,6.585

Mozambique,MOZ,1978,6.539

Mozambique,MOZ,1979,6.499

Mozambique,MOZ,1980,6.464

Mozambique,MOZ,1981,6.436

Mozambique,MOZ,1982,6.413

Mozambique,MOZ,1983,6.392

Mozambique,MOZ,1984,6.373

Mozambique,MOZ,1985,6.354

Mozambique,MOZ,1986,6.332

Mozambique,MOZ,1987,6.308

Mozambique,MOZ,1988,6.281

Mozambique,MOZ,1989,6.248

Mozambique,MOZ,1990,6.211

Mozambique,MOZ,1991,6.167

Mozambique,MOZ,1992,6.119

Mozambique,MOZ,1993,6.067

Mozambique,MOZ,1994,6.015

Mozambique,MOZ,1995,5.964

Mozambique,MOZ,1996,5.919

Mozambique,MOZ,1997,5.882

Mozambique,MOZ,1998,5.853

Mozambique,MOZ,1999,5.832

Mozambique,MOZ,2000,5.818

Mozambique,MOZ,2001,5.807

Mozambique,MOZ,2002,5.795

Mozambique,MOZ,2003,5.781

Mozambique,MOZ,2004,5.761

Mozambique,MOZ,2005,5.736

Mozambique,MOZ,2006,5.706

Mozambique,MOZ,2007,5.674

Mozambique,MOZ,2008,5.639

Mozambique,MOZ,2009,5.602

Mozambique,MOZ,2010,5.562

Mozambique,MOZ,2011,5.519

Mozambique,MOZ,2012,5.472

Mozambique,MOZ,2013,5.421

Mozambique,MOZ,2014,5.365

Mozambique,MOZ,2015,5.305

Myanmar,MMR,1950,6.026

Myanmar,MMR,1951,6.017

Myanmar,MMR,1952,6.002

Myanmar,MMR,1953,5.991

Myanmar,MMR,1954,5.985

Myanmar,MMR,1955,5.985

Myanmar,MMR,1956,5.989

Myanmar,MMR,1957,5.999

Myanmar,MMR,1958,6.012

Myanmar,MMR,1959,6.03

Myanmar,MMR,1960,6.051

Myanmar,MMR,1961,6.073

Myanmar,MMR,1962,6.096

Myanmar,MMR,1963,6.116

Myanmar,MMR,1964,6.13

Myanmar,MMR,1965,6.136

Myanmar,MMR,1966,6.13

Myanmar,MMR,1967,6.111

Myanmar,MMR,1968,6.077

Myanmar,MMR,1969,6.029

Myanmar,MMR,1970,5.964

Myanmar,MMR,1971,5.884

Myanmar,MMR,1972,5.79

Myanmar,MMR,1973,5.685

Myanmar,MMR,1974,5.572

Myanmar,MMR,1975,5.457

Myanmar,MMR,1976,5.343

Myanmar,MMR,1977,5.233

Myanmar,MMR,1978,5.126

Myanmar,MMR,1979,5.024

Myanmar,MMR,1980,4.92

Myanmar,MMR,1981,4.809

Myanmar,MMR,1982,4.685

Myanmar,MMR,1983,4.547

Myanmar,MMR,1984,4.395

Myanmar,MMR,1985,4.231

Myanmar,MMR,1986,4.062

Myanmar,MMR,1987,3.894

Myanmar,MMR,1988,3.734

Myanmar,MMR,1989,3.586

Myanmar,MMR,1990,3.455

Myanmar,MMR,1991,3.341

Myanmar,MMR,1992,3.243

Myanmar,MMR,1993,3.16

Myanmar,MMR,1994,3.09

Myanmar,MMR,1995,3.035

Myanmar,MMR,1996,2.994

Myanmar,MMR,1997,2.965

Myanmar,MMR,1998,2.945

Myanmar,MMR,1999,2.929

Myanmar,MMR,2000,2.914

Myanmar,MMR,2001,2.894

Myanmar,MMR,2002,2.865

Myanmar,MMR,2003,2.827

Myanmar,MMR,2004,2.779

Myanmar,MMR,2005,2.722

Myanmar,MMR,2006,2.658

Myanmar,MMR,2007,2.591

Myanmar,MMR,2008,2.526

Myanmar,MMR,2009,2.465

Myanmar,MMR,2010,2.41

Myanmar,MMR,2011,2.363

Myanmar,MMR,2012,2.322

Myanmar,MMR,2013,2.286

Myanmar,MMR,2014,2.256

Myanmar,MMR,2015,2.23

Namibia,NAM,1950,5.962

Namibia,NAM,1951,5.971

Namibia,NAM,1952,5.99

Namibia,NAM,1953,6.009

Namibia,NAM,1954,6.029

Namibia,NAM,1955,6.049

Namibia,NAM,1956,6.069

Namibia,NAM,1957,6.09

Namibia,NAM,1958,6.111

Namibia,NAM,1959,6.13

Namibia,NAM,1960,6.149

Namibia,NAM,1961,6.167

Namibia,NAM,1962,6.184

Namibia,NAM,1963,6.199

Namibia,NAM,1964,6.216

Namibia,NAM,1965,6.236

Namibia,NAM,1966,6.263

Namibia,NAM,1967,6.301

Namibia,NAM,1968,6.347

Namibia,NAM,1969,6.401

Namibia,NAM,1970,6.459

Namibia,NAM,1971,6.517

Namibia,NAM,1972,6.57

Namibia,NAM,1973,6.613

Namibia,NAM,1974,6.642

Namibia,NAM,1975,6.654

Namibia,NAM,1976,6.647

Namibia,NAM,1977,6.622

Namibia,NAM,1978,6.581

Namibia,NAM,1979,6.524

Namibia,NAM,1980,6.451

Namibia,NAM,1981,6.362

Namibia,NAM,1982,6.259

Namibia,NAM,1983,6.145

Namibia,NAM,1984,6.022

Namibia,NAM,1985,5.893

Namibia,NAM,1986,5.76

Namibia,NAM,1987,5.625

Namibia,NAM,1988,5.491

Namibia,NAM,1989,5.358

Namibia,NAM,1990,5.227

Namibia,NAM,1991,5.098

Namibia,NAM,1992,4.97

Namibia,NAM,1993,4.842

Namibia,NAM,1994,4.715

Namibia,NAM,1995,4.589

Namibia,NAM,1996,4.466

Namibia,NAM,1997,4.345

Namibia,NAM,1998,4.229

Namibia,NAM,1999,4.119

Namibia,NAM,2000,4.018

Namibia,NAM,2001,3.926

Namibia,NAM,2002,3.844

Namibia,NAM,2003,3.773

Namibia,NAM,2004,3.714

Namibia,NAM,2005,3.667

Namibia,NAM,2006,3.635

Namibia,NAM,2007,3.616

Namibia,NAM,2008,3.608

Namibia,NAM,2009,3.606

Namibia,NAM,2010,3.605

Namibia,NAM,2011,3.6

Namibia,NAM,2012,3.586

Namibia,NAM,2013,3.56

Namibia,NAM,2014,3.522

Namibia,NAM,2015,3.473

Nepal,NPL,1950,5.958

Nepal,NPL,1951,5.958

Nepal,NPL,1952,5.959

Nepal,NPL,1953,5.959

Nepal,NPL,1954,5.959

Nepal,NPL,1955,5.959

Nepal,NPL,1956,5.959

Nepal,NPL,1957,5.959

Nepal,NPL,1958,5.959

Nepal,NPL,1959,5.959

Nepal,NPL,1960,5.959

Nepal,NPL,1961,5.96

Nepal,NPL,1962,5.962

Nepal,NPL,1963,5.964

Nepal,NPL,1964,5.965

Nepal,NPL,1965,5.966

Nepal,NPL,1966,5.963

Nepal,NPL,1967,5.958

Nepal,NPL,1968,5.948

Nepal,NPL,1969,5.934

Nepal,NPL,1970,5.918

Nepal,NPL,1971,5.901

Nepal,NPL,1972,5.884

Nepal,NPL,1973,5.868

Nepal,NPL,1974,5.853

Nepal,NPL,1975,5.838

Nepal,NPL,1976,5.822

Nepal,NPL,1977,5.804

Nepal,NPL,1978,5.783

Nepal,NPL,1979,5.756

Nepal,NPL,1980,5.725

Nepal,NPL,1981,5.687

Nepal,NPL,1982,5.645

Nepal,NPL,1983,5.597

Nepal,NPL,1984,5.546

Nepal,NPL,1985,5.49

Nepal,NPL,1986,5.431

Nepal,NPL,1987,5.371

Nepal,NPL,1988,5.308

Nepal,NPL,1989,5.242

Nepal,NPL,1990,5.172

Nepal,NPL,1991,5.097

Nepal,NPL,1992,5.015

Nepal,NPL,1993,4.926

Nepal,NPL,1994,4.828

Nepal,NPL,1995,4.72

Nepal,NPL,1996,4.6

Nepal,NPL,1997,4.47

Nepal,NPL,1998,4.33

Nepal,NPL,1999,4.182

Nepal,NPL,2000,4.03

Nepal,NPL,2001,3.877

Nepal,NPL,2002,3.724

Nepal,NPL,2003,3.575

Nepal,NPL,2004,3.43

Nepal,NPL,2005,3.29

Nepal,NPL,2006,3.151

Nepal,NPL,2007,3.012

Nepal,NPL,2008,2.874

Nepal,NPL,2009,2.737

Nepal,NPL,2010,2.606

Nepal,NPL,2011,2.486

Nepal,NPL,2012,2.38

Nepal,NPL,2013,2.291

Nepal,NPL,2014,2.218

Nepal,NPL,2015,2.162

Netherlands,NLD,1950,3.053

Netherlands,NLD,1951,3.05

Netherlands,NLD,1952,3.047

Netherlands,NLD,1953,3.05

Netherlands,NLD,1954,3.057

Netherlands,NLD,1955,3.069

Netherlands,NLD,1956,3.086

Netherlands,NLD,1957,3.106

Netherlands,NLD,1958,3.128

Netherlands,NLD,1959,3.149

Netherlands,NLD,1960,3.165

Netherlands,NLD,1961,3.172

Netherlands,NLD,1962,3.166

Netherlands,NLD,1963,3.143

Netherlands,NLD,1964,3.101

Netherlands,NLD,1965,3.038

Netherlands,NLD,1966,2.953

Netherlands,NLD,1967,2.848

Netherlands,NLD,1968,2.727

Netherlands,NLD,1969,2.596

Netherlands,NLD,1970,2.458

Netherlands,NLD,1971,2.316

Netherlands,NLD,1972,2.175

Netherlands,NLD,1973,2.039

Netherlands,NLD,1974,1.913

Netherlands,NLD,1975,1.803

Netherlands,NLD,1976,1.711

Netherlands,NLD,1977,1.636

Netherlands,NLD,1978,1.579

Netherlands,NLD,1979,1.539

Netherlands,NLD,1980,1.514

Netherlands,NLD,1981,1.503

Netherlands,NLD,1982,1.502

Netherlands,NLD,1983,1.506

Netherlands,NLD,1984,1.514

Netherlands,NLD,1985,1.524

Netherlands,NLD,1986,1.534

Netherlands,NLD,1987,1.545

Netherlands,NLD,1988,1.556

Netherlands,NLD,1989,1.567

Netherlands,NLD,1990,1.575

Netherlands,NLD,1991,1.581

Netherlands,NLD,1992,1.584

Netherlands,NLD,1993,1.586

Netherlands,NLD,1994,1.586

Netherlands,NLD,1995,1.588

Netherlands,NLD,1996,1.594

Netherlands,NLD,1997,1.605

Netherlands,NLD,1998,1.622

Netherlands,NLD,1999,1.644

Netherlands,NLD,2000,1.67

Netherlands,NLD,2001,1.695

Netherlands,NLD,2002,1.718

Netherlands,NLD,2003,1.736

Netherlands,NLD,2004,1.748

Netherlands,NLD,2005,1.754

Netherlands,NLD,2006,1.754

Netherlands,NLD,2007,1.751

Netherlands,NLD,2008,1.747

Netherlands,NLD,2009,1.743

Netherlands,NLD,2010,1.739

Netherlands,NLD,2011,1.736

Netherlands,NLD,2012,1.735

Netherlands,NLD,2013,1.735

Netherlands,NLD,2014,1.736

Netherlands,NLD,2015,1.739

New Caledonia,NCL,1950,5.224

New Caledonia,NCL,1951,5.223

New Caledonia,NCL,1952,5.223

New Caledonia,NCL,1953,5.223

New Caledonia,NCL,1954,5.223

New Caledonia,NCL,1955,5.223

New Caledonia,NCL,1956,5.223

New Caledonia,NCL,1957,5.223

New Caledonia,NCL,1958,5.224

New Caledonia,NCL,1959,5.224

New Caledonia,NCL,1960,5.224

New Caledonia,NCL,1961,5.224

New Caledonia,NCL,1962,5.223

New Caledonia,NCL,1963,5.222

New Caledonia,NCL,1964,5.22

New Caledonia,NCL,1965,5.222

New Caledonia,NCL,1966,5.234

New Caledonia,NCL,1967,5.255

New Caledonia,NCL,1968,5.28

New Caledonia,NCL,1969,5.302

New Caledonia,NCL,1970,5.304

New Caledonia,NCL,1971,5.264

New Caledonia,NCL,1972,5.173

New Caledonia,NCL,1973,5.027

New Caledonia,NCL,1974,4.83

New Caledonia,NCL,1975,4.596

New Caledonia,NCL,1976,4.345

New Caledonia,NCL,1977,4.1

New Caledonia,NCL,1978,3.881

New Caledonia,NCL,1979,3.696

New Caledonia,NCL,1980,3.551

New Caledonia,NCL,1981,3.44

New Caledonia,NCL,1982,3.351

New Caledonia,NCL,1983,3.274

New Caledonia,NCL,1984,3.205

New Caledonia,NCL,1985,3.144

New Caledonia,NCL,1986,3.093

New Caledonia,NCL,1987,3.053

New Caledonia,NCL,1988,3.024

New Caledonia,NCL,1989,3.002

New Caledonia,NCL,1990,2.983

New Caledonia,NCL,1991,2.96

New Caledonia,NCL,1992,2.928

New Caledonia,NCL,1993,2.886

New Caledonia,NCL,1994,2.832

New Caledonia,NCL,1995,2.768

New Caledonia,NCL,1996,2.695

New Caledonia,NCL,1997,2.621

New Caledonia,NCL,1998,2.548

New Caledonia,NCL,1999,2.482

New Caledonia,NCL,2000,2.424

New Caledonia,NCL,2001,2.378

New Caledonia,NCL,2002,2.341

New Caledonia,NCL,2003,2.314

New Caledonia,NCL,2004,2.295

New Caledonia,NCL,2005,2.283

New Caledonia,NCL,2006,2.277

New Caledonia,NCL,2007,2.274

New Caledonia,NCL,2008,2.272

New Caledonia,NCL,2009,2.269

New Caledonia,NCL,2010,2.264

New Caledonia,NCL,2011,2.255

New Caledonia,NCL,2012,2.243

New Caledonia,NCL,2013,2.229

New Caledonia,NCL,2014,2.213

New Caledonia,NCL,2015,2.194

New Zealand,NZL,1950,3.385

New Zealand,NZL,1951,3.474

New Zealand,NZL,1952,3.638

New Zealand,NZL,1953,3.777

New Zealand,NZL,1954,3.889

New Zealand,NZL,1955,3.975

New Zealand,NZL,1956,4.035

New Zealand,NZL,1957,4.069

New Zealand,NZL,1958,4.079

New Zealand,NZL,1959,4.066

New Zealand,NZL,1960,4.03

New Zealand,NZL,1961,3.975

New Zealand,NZL,1962,3.903

New Zealand,NZL,1963,3.818

New Zealand,NZL,1964,3.723

New Zealand,NZL,1965,3.623

New Zealand,NZL,1966,3.521

New Zealand,NZL,1967,3.418

New Zealand,NZL,1968,3.315

New Zealand,NZL,1969,3.212

New Zealand,NZL,1970,3.107

New Zealand,NZL,1971,2.997

New Zealand,NZL,1972,2.878

New Zealand,NZL,1973,2.751

New Zealand,NZL,1974,2.619

New Zealand,NZL,1975,2.487

New Zealand,NZL,1976,2.361

New Zealand,NZL,1977,2.247

New Zealand,NZL,1978,2.151

New Zealand,NZL,1979,2.075

New Zealand,NZL,1980,2.02

New Zealand,NZL,1981,1.987

New Zealand,NZL,1982,1.971

New Zealand,NZL,1983,1.966

New Zealand,NZL,1984,1.971

New Zealand,NZL,1985,1.982

New Zealand,NZL,1986,1.998

New Zealand,NZL,1987,2.016

New Zealand,NZL,1988,2.034

New Zealand,NZL,1989,2.05

New Zealand,NZL,1990,2.061

New Zealand,NZL,1991,2.064

New Zealand,NZL,1992,2.06

New Zealand,NZL,1993,2.05

New Zealand,NZL,1994,2.033

New Zealand,NZL,1995,2.012

New Zealand,NZL,1996,1.989

New Zealand,NZL,1997,1.966

New Zealand,NZL,1998,1.947

New Zealand,NZL,1999,1.933

New Zealand,NZL,2000,1.929

New Zealand,NZL,2001,1.936

New Zealand,NZL,2002,1.954

New Zealand,NZL,2003,1.982

New Zealand,NZL,2004,2.016

New Zealand,NZL,2005,2.053

New Zealand,NZL,2006,2.085

New Zealand,NZL,2007,2.109

New Zealand,NZL,2008,2.121

New Zealand,NZL,2009,2.12

New Zealand,NZL,2010,2.108

New Zealand,NZL,2011,2.087

New Zealand,NZL,2012,2.063

New Zealand,NZL,2013,2.039

New Zealand,NZL,2014,2.019

New Zealand,NZL,2015,2.002

Nicaragua,NIC,1950,6.884

Nicaragua,NIC,1951,6.982

Nicaragua,NIC,1952,7.158

Nicaragua,NIC,1953,7.297

Nicaragua,NIC,1954,7.4

Nicaragua,NIC,1955,7.466

Nicaragua,NIC,1956,7.497

Nicaragua,NIC,1957,7.495

Nicaragua,NIC,1958,7.463

Nicaragua,NIC,1959,7.408

Nicaragua,NIC,1960,7.336

Nicaragua,NIC,1961,7.257

Nicaragua,NIC,1962,7.179

Nicaragua,NIC,1963,7.109

Nicaragua,NIC,1964,7.052

Nicaragua,NIC,1965,7.01

Nicaragua,NIC,1966,6.981

Nicaragua,NIC,1967,6.961

Nicaragua,NIC,1968,6.941

Nicaragua,NIC,1969,6.919

Nicaragua,NIC,1970,6.892

Nicaragua,NIC,1971,6.855

Nicaragua,NIC,1972,6.807

Nicaragua,NIC,1973,6.748

Nicaragua,NIC,1974,6.678

Nicaragua,NIC,1975,6.597

Nicaragua,NIC,1976,6.51

Nicaragua,NIC,1977,6.42

Nicaragua,NIC,1978,6.327

Nicaragua,NIC,1979,6.232

Nicaragua,NIC,1980,6.132

Nicaragua,NIC,1981,6.022

Nicaragua,NIC,1982,5.9

Nicaragua,NIC,1983,5.762

Nicaragua,NIC,1984,5.611

Nicaragua,NIC,1985,5.448

Nicaragua,NIC,1986,5.278

Nicaragua,NIC,1987,5.104

Nicaragua,NIC,1988,4.931

Nicaragua,NIC,1989,4.762

Nicaragua,NIC,1990,4.597

Nicaragua,NIC,1991,4.433

Nicaragua,NIC,1992,4.27

Nicaragua,NIC,1993,4.105

Nicaragua,NIC,1994,3.942

Nicaragua,NIC,1995,3.78

Nicaragua,NIC,1996,3.623

Nicaragua,NIC,1997,3.473

Nicaragua,NIC,1998,3.331

Nicaragua,NIC,1999,3.201

Nicaragua,NIC,2000,3.083

Nicaragua,NIC,2001,2.978

Nicaragua,NIC,2002,2.887

Nicaragua,NIC,2003,2.807

Nicaragua,NIC,2004,2.738

Nicaragua,NIC,2005,2.677

Nicaragua,NIC,2006,2.622

Nicaragua,NIC,2007,2.571

Nicaragua,NIC,2008,2.522

Nicaragua,NIC,2009,2.475

Nicaragua,NIC,2010,2.428

Nicaragua,NIC,2011,2.383

Nicaragua,NIC,2012,2.34

Nicaragua,NIC,2013,2.3

Nicaragua,NIC,2014,2.264

Nicaragua,NIC,2015,2.231

Niger,NER,1950,7.262

Niger,NER,1951,7.271

Niger,NER,1952,7.29

Niger,NER,1953,7.309

Niger,NER,1954,7.329

Niger,NER,1955,7.35

Niger,NER,1956,7.37

Niger,NER,1957,7.392

Niger,NER,1958,7.413

Niger,NER,1959,7.434

Niger,NER,1960,7.454

Niger,NER,1961,7.473

Niger,NER,1962,7.49

Niger,NER,1963,7.505

Niger,NER,1964,7.518

Niger,NER,1965,7.529

Niger,NER,1966,7.537

Niger,NER,1967,7.544

Niger,NER,1968,7.551

Niger,NER,1969,7.558

Niger,NER,1970,7.567

Niger,NER,1971,7.58

Niger,NER,1972,7.596

Niger,NER,1973,7.616

Niger,NER,1974,7.64

Niger,NER,1975,7.668

Niger,NER,1976,7.701

Niger,NER,1977,7.738

Niger,NER,1978,7.775

Niger,NER,1979,7.812

Niger,NER,1980,7.844

Niger,NER,1981,7.868

Niger,NER,1982,7.882

Niger,NER,1983,7.886

Niger,NER,1984,7.88

Niger,NER,1985,7.865

Niger,NER,1986,7.845

Niger,NER,1987,7.824

Niger,NER,1988,7.803

Niger,NER,1989,7.786

Niger,NER,1990,7.772

Niger,NER,1991,7.761

Niger,NER,1992,7.752

Niger,NER,1993,7.743

Niger,NER,1994,7.734

Niger,NER,1995,7.725

Niger,NER,1996,7.716

Niger,NER,1997,7.707

Niger,NER,1998,7.698

Niger,NER,1999,7.689

Niger,NER,2000,7.679

Niger,NER,2001,7.668

Niger,NER,2002,7.656

Niger,NER,2003,7.642

Niger,NER,2004,7.626

Niger,NER,2005,7.608

Niger,NER,2006,7.588

Niger,NER,2007,7.566

Niger,NER,2008,7.542

Niger,NER,2009,7.515

Niger,NER,2010,7.487

Niger,NER,2011,7.455

Niger,NER,2012,7.42

Niger,NER,2013,7.381

Niger,NER,2014,7.338

Niger,NER,2015,7.29

Nigeria,NGA,1950,6.357

Nigeria,NGA,1951,6.356

Nigeria,NGA,1952,6.354

Nigeria,NGA,1953,6.353

Nigeria,NGA,1954,6.353

Nigeria,NGA,1955,6.353

Nigeria,NGA,1956,6.354

Nigeria,NGA,1957,6.354

Nigeria,NGA,1958,6.355

Nigeria,NGA,1959,6.355

Nigeria,NGA,1960,6.354

Nigeria,NGA,1961,6.351

Nigeria,NGA,1962,6.346

Nigeria,NGA,1963,6.341

Nigeria,NGA,1964,6.336

Nigeria,NGA,1965,6.335

Nigeria,NGA,1966,6.342

Nigeria,NGA,1967,6.359

Nigeria,NGA,1968,6.387

Nigeria,NGA,1969,6.425

Nigeria,NGA,1970,6.471

Nigeria,NGA,1971,6.522

Nigeria,NGA,1972,6.575

Nigeria,NGA,1973,6.625

Nigeria,NGA,1974,6.669

Nigeria,NGA,1975,6.706

Nigeria,NGA,1976,6.735

Nigeria,NGA,1977,6.757

Nigeria,NGA,1978,6.772

Nigeria,NGA,1979,6.781

Nigeria,NGA,1980,6.783

Nigeria,NGA,1981,6.779

Nigeria,NGA,1982,6.767

Nigeria,NGA,1983,6.749

Nigeria,NGA,1984,6.726

Nigeria,NGA,1985,6.698

Nigeria,NGA,1986,6.664

Nigeria,NGA,1987,6.625

Nigeria,NGA,1988,6.582

Nigeria,NGA,1989,6.537

Nigeria,NGA,1990,6.49

Nigeria,NGA,1991,6.443

Nigeria,NGA,1992,6.395

Nigeria,NGA,1993,6.348

Nigeria,NGA,1994,6.303

Nigeria,NGA,1995,6.262

Nigeria,NGA,1996,6.224

Nigeria,NGA,1997,6.19

Nigeria,NGA,1998,6.159

Nigeria,NGA,1999,6.131

Nigeria,NGA,2000,6.106

Nigeria,NGA,2001,6.083

Nigeria,NGA,2002,6.06

Nigeria,NGA,2003,6.036

Nigeria,NGA,2004,6.011

Nigeria,NGA,2005,5.985

Nigeria,NGA,2006,5.958

Nigeria,NGA,2007,5.93

Nigeria,NGA,2008,5.902

Nigeria,NGA,2009,5.872

Nigeria,NGA,2010,5.839

Nigeria,NGA,2011,5.802

Nigeria,NGA,2012,5.758

Nigeria,NGA,2013,5.709

Nigeria,NGA,2014,5.653

Nigeria,NGA,2015,5.591

North Korea,PRK,1950,1.946

North Korea,PRK,1951,2.407

North Korea,PRK,1952,3.247

North Korea,PRK,1953,3.923

North Korea,PRK,1954,4.435

North Korea,PRK,1955,4.786

North Korea,PRK,1956,4.981

North Korea,PRK,1957,5.031

North Korea,PRK,1958,4.957

North Korea,PRK,1959,4.793

North Korea,PRK,1960,4.579

North Korea,PRK,1961,4.357

North Korea,PRK,1962,4.173

North Korea,PRK,1963,4.057

North Korea,PRK,1964,4.022

North Korea,PRK,1965,4.063

North Korea,PRK,1966,4.157

North Korea,PRK,1967,4.257

North Korea,PRK,1968,4.327

North Korea,PRK,1969,4.352

North Korea,PRK,1970,4.315

North Korea,PRK,1971,4.21

North Korea,PRK,1972,4.05

North Korea,PRK,1973,3.852

North Korea,PRK,1974,3.63

North Korea,PRK,1975,3.404

North Korea,PRK,1976,3.197

North Korea,PRK,1977,3.025

North Korea,PRK,1978,2.897

North Korea,PRK,1979,2.815

North Korea,PRK,1980,2.773

North Korea,PRK,1981,2.753

North Korea,PRK,1982,2.734

North Korea,PRK,1983,2.701

North Korea,PRK,1984,2.65

North Korea,PRK,1985,2.581

North Korea,PRK,1986,2.503

North Korea,PRK,1987,2.429

North Korea,PRK,1988,2.369

North Korea,PRK,1989,2.323

North Korea,PRK,1990,2.289

North Korea,PRK,1991,2.262

North Korea,PRK,1992,2.235

North Korea,PRK,1993,2.202

North Korea,PRK,1994,2.164

North Korea,PRK,1995,2.121

North Korea,PRK,1996,2.079

North Korea,PRK,1997,2.042

North Korea,PRK,1998,2.014

North Korea,PRK,1999,1.996

North Korea,PRK,2000,1.987

North Korea,PRK,2001,1.987

North Korea,PRK,2002,1.99

North Korea,PRK,2003,1.995

North Korea,PRK,2004,1.999

North Korea,PRK,2005,2.002

North Korea,PRK,2006,2.001

North Korea,PRK,2007,1.999

North Korea,PRK,2008,1.994

North Korea,PRK,2009,1.987

North Korea,PRK,2010,1.979

North Korea,PRK,2011,1.968

North Korea,PRK,2012,1.957

North Korea,PRK,2013,1.945

North Korea,PRK,2014,1.933

North Korea,PRK,2015,1.921

Northern Africa,,1950,6.773

Northern Africa,,1951,6.78

Northern Africa,,1952,6.793

Northern Africa,,1953,6.808

Northern Africa,,1954,6.823

Northern Africa,,1955,6.839

Northern Africa,,1956,6.855

Northern Africa,,1957,6.873

Northern Africa,,1958,6.89

Northern Africa,,1959,6.906

Northern Africa,,1960,6.92

Northern Africa,,1961,6.93

Northern Africa,,1962,6.936

Northern Africa,,1963,6.935

Northern Africa,,1964,6.927

Northern Africa,,1965,6.91

Northern Africa,,1966,6.884

Northern Africa,,1967,6.848

Northern Africa,,1968,6.803

Northern Africa,,1969,6.751

Northern Africa,,1970,6.694

Northern Africa,,1971,6.631

Northern Africa,,1972,6.567

Northern Africa,,1973,6.501

Northern Africa,,1974,6.433

Northern Africa,,1975,6.366

Northern Africa,,1976,6.297

Northern Africa,,1977,6.226

Northern Africa,,1978,6.151

Northern Africa,,1979,6.071

Northern Africa,,1980,5.985

Northern Africa,,1981,5.894

Northern Africa,,1982,5.797

Northern Africa,,1983,5.694

Northern Africa,,1984,5.582

Northern Africa,,1985,5.46

Northern Africa,,1986,5.325

Northern Africa,,1987,5.177

Northern Africa,,1988,5.017

Northern Africa,,1989,4.846

Northern Africa,,1990,4.668

Northern Africa,,1991,4.485

Northern Africa,,1992,4.301

Northern Africa,,1993,4.122

Northern Africa,,1994,3.951

Northern Africa,,1995,3.793

Northern Africa,,1996,3.652

Northern Africa,,1997,3.527

Northern Africa,,1998,3.419

Northern Africa,,1999,3.328

Northern Africa,,2000,3.255

Northern Africa,,2001,3.197

Northern Africa,,2002,3.152

Northern Africa,,2003,3.118

Northern Africa,,2004,3.094

Northern Africa,,2005,3.082

Northern Africa,,2006,3.084

Northern Africa,,2007,3.102

Northern Africa,,2008,3.133

Northern Africa,,2009,3.172

Northern Africa,,2010,3.214

Northern Africa,,2011,3.248

Northern Africa,,2012,3.27

Northern Africa,,2013,3.274

Northern Africa,,2014,3.26

Northern Africa,,2015,3.229

Northern America,,1950,3.084

Northern America,,1951,3.159

Northern America,,1952,3.298

Northern America,,1953,3.412

Northern America,,1954,3.501

Northern America,,1955,3.566

Northern America,,1956,3.607

Northern America,,1957,3.622

Northern America,,1958,3.613

Northern America,,1959,3.579

Northern America,,1960,3.52

Northern America,,1961,3.437

Northern America,,1962,3.332

Northern America,,1963,3.209

Northern America,,1964,3.073

Northern America,,1965,2.929

Northern America,,1966,2.782

Northern America,,1967,2.636

Northern America,,1968,2.497

Northern America,,1969,2.368

Northern America,,1970,2.252

Northern America,,1971,2.15

Northern America,,1972,2.059

Northern America,,1973,1.979

Northern America,,1974,1.91

Northern America,,1975,1.853

Northern America,,1976,1.809

Northern America,,1977,1.778

Northern America,,1978,1.759

Northern America,,1979,1.749

Northern America,,1980,1.749

Northern America,,1981,1.757

Northern America,,1982,1.77

Northern America,,1983,1.788

Northern America,,1984,1.807

Northern America,,1985,1.828

Northern America,,1986,1.851

Northern America,,1987,1.876

Northern America,,1988,1.902

Northern America,,1989,1.928

Northern America,,1990,1.951

Northern America,,1991,1.969

Northern America,,1992,1.981

Northern America,,1993,1.987

Northern America,,1994,1.986

Northern America,,1995,1.981

Northern America,,1996,1.974

Northern America,,1997,1.968

Northern America,,1998,1.964

Northern America,,1999,1.964

Northern America,,2000,1.969

Northern America,,2001,1.978

Northern America,,2002,1.989

Northern America,,2003,2

Northern America,,2004,2.009

Northern America,,2005,2.015

Northern America,,2006,2.013

Northern America,,2007,2.003

Northern America,,2008,1.985

Northern America,,2009,1.96

Northern America,,2010,1.932

Northern America,,2011,1.903

Northern America,,2012,1.878

Northern America,,2013,1.858

Northern America,,2014,1.846

Northern America,,2015,1.841

Northern Europe,,1950,2.257

Northern Europe,,1951,2.27

Northern Europe,,1952,2.296

Northern Europe,,1953,2.328

Northern Europe,,1954,2.365

Northern Europe,,1955,2.406

Northern Europe,,1956,2.452

Northern Europe,,1957,2.501

Northern Europe,,1958,2.551

Northern Europe,,1959,2.599

Northern Europe,,1960,2.642

Northern Europe,,1961,2.675

Northern Europe,,1962,2.695

Northern Europe,,1963,2.698

Northern Europe,,1964,2.683

Northern Europe,,1965,2.649

Northern Europe,,1966,2.595

Northern Europe,,1967,2.527

Northern Europe,,1968,2.449

Northern Europe,,1969,2.364

Northern Europe,,1970,2.277

Northern Europe,,1971,2.19

Northern Europe,,1972,2.106

Northern Europe,,1973,2.027

Northern Europe,,1974,1.957

Northern Europe,,1975,1.898

Northern Europe,,1976,1.851

Northern Europe,,1977,1.817

Northern Europe,,1978,1.793

Northern Europe,,1979,1.779

Northern Europe,,1980,1.774

Northern Europe,,1981,1.777

Northern Europe,,1982,1.786

Northern Europe,,1983,1.798

Northern Europe,,1984,1.812

Northern Europe,,1985,1.825

Northern Europe,,1986,1.835

Northern Europe,,1987,1.841

Northern Europe,,1988,1.843

Northern Europe,,1989,1.841

Northern Europe,,1990,1.834

Northern Europe,,1991,1.822

Northern Europe,,1992,1.807

Northern Europe,,1993,1.789

Northern Europe,,1994,1.769

Northern Europe,,1995,1.749

Northern Europe,,1996,1.728

Northern Europe,,1997,1.707

Northern Europe,,1998,1.688

Northern Europe,,1999,1.671

Northern Europe,,2000,1.661

Northern Europe,,2001,1.661

Northern Europe,,2002,1.671

Northern Europe,,2003,1.691

Northern Europe,,2004,1.721

Northern Europe,,2005,1.755

Northern Europe,,2006,1.79

Northern Europe,,2007,1.821

Northern Europe,,2008,1.844

Northern Europe,,2009,1.859

Northern Europe,,2010,1.865

Northern Europe,,2011,1.864

Northern Europe,,2012,1.86

Northern Europe,,2013,1.856

Northern Europe,,2014,1.853

Northern Europe,,2015,1.852

Norway,NOR,1950,2.464

Norway,NOR,1951,2.502

Norway,NOR,1952,2.575

Norway,NOR,1953,2.64

Norway,NOR,1954,2.698

Norway,NOR,1955,2.748

Norway,NOR,1956,2.791

Norway,NOR,1957,2.827

Norway,NOR,1958,2.856

Norway,NOR,1959,2.878

Norway,NOR,1960,2.895

Norway,NOR,1961,2.906

Norway,NOR,1962,2.912

Norway,NOR,1963,2.912

Norway,NOR,1964,2.905

Norway,NOR,1965,2.889

Norway,NOR,1966,2.861

Norway,NOR,1967,2.819

Norway,NOR,1968,2.763

Norway,NOR,1969,2.693

Norway,NOR,1970,2.608

Norway,NOR,1971,2.509

Norway,NOR,1972,2.4

Norway,NOR,1973,2.284

Norway,NOR,1974,2.168

Norway,NOR,1975,2.055

Norway,NOR,1976,1.953

Norway,NOR,1977,1.864

Norway,NOR,1978,1.79

Norway,NOR,1979,1.735

Norway,NOR,1980,1.699

Norway,NOR,1981,1.681

Norway,NOR,1982,1.679

Norway,NOR,1983,1.688

Norway,NOR,1984,1.704

Norway,NOR,1985,1.726

Norway,NOR,1986,1.752

Norway,NOR,1987,1.779

Norway,NOR,1988,1.807

Norway,NOR,1989,1.832

Norway,NOR,1990,1.853

Norway,NOR,1991,1.869

Norway,NOR,1992,1.879

Norway,NOR,1993,1.886

Norway,NOR,1994,1.887

Norway,NOR,1995,1.884

Norway,NOR,1996,1.876

Norway,NOR,1997,1.865

Norway,NOR,1998,1.85

Norway,NOR,1999,1.836

Norway,NOR,2000,1.824

Norway,NOR,2001,1.819

Norway,NOR,2002,1.821

Norway,NOR,2003,1.832

Norway,NOR,2004,1.848

Norway,NOR,2005,1.868

Norway,NOR,2006,1.886

Norway,NOR,2007,1.898

Norway,NOR,2008,1.9

Norway,NOR,2009,1.894

Norway,NOR,2010,1.879

Norway,NOR,2011,1.86

Norway,NOR,2012,1.842

Norway,NOR,2013,1.828

Norway,NOR,2014,1.819

Norway,NOR,2015,1.816

Oceania,,1950,3.671

Oceania,,1951,3.72

Oceania,,1952,3.812

Oceania,,1953,3.889

Oceania,,1954,3.953

Oceania,,1955,4.003

Oceania,,1956,4.04

Oceania,,1957,4.062

Oceania,,1958,4.071

Oceania,,1959,4.065

Oceania,,1960,4.045

Oceania,,1961,4.011

Oceania,,1962,3.964

Oceania,,1963,3.905

Oceania,,1964,3.837

Oceania,,1965,3.763

Oceania,,1966,3.687

Oceania,,1967,3.611

Oceania,,1968,3.536

Oceania,,1969,3.464

Oceania,,1970,3.392

Oceania,,1971,3.316

Oceania,,1972,3.234

Oceania,,1973,3.144

Oceania,,1974,3.05

Oceania,,1975,2.955

Oceania,,1976,2.864

Oceania,,1977,2.785

Oceania,,1978,2.719

Oceania,,1979,2.668

Oceania,,1980,2.631

Oceania,,1981,2.606

Oceania,,1982,2.588

Oceania,,1983,2.571

Oceania,,1984,2.556

Oceania,,1985,2.541

Oceania,,1986,2.526

Oceania,,1987,2.514

Oceania,,1988,2.506

Oceania,,1989,2.5

Oceania,,1990,2.496

Oceania,,1991,2.494

Oceania,,1992,2.491

Oceania,,1993,2.487

Oceania,,1994,2.483

Oceania,,1995,2.476

Oceania,,1996,2.468

Oceania,,1997,2.46

Oceania,,1998,2.452

Oceania,,1999,2.445

Oceania,,2000,2.441

Oceania,,2001,2.442

Oceania,,2002,2.449

Oceania,,2003,2.46

Oceania,,2004,2.475

Oceania,,2005,2.491

Oceania,,2006,2.504

Oceania,,2007,2.51

Oceania,,2008,2.509

Oceania,,2009,2.499

Oceania,,2010,2.481

Oceania,,2011,2.459

Oceania,,2012,2.434

Oceania,,2013,2.41

Oceania,,2014,2.389

Oceania,,2015,2.37

Oman,OMN,1950,7.25

Oman,OMN,1951,7.251

Oman,OMN,1952,7.252

Oman,OMN,1953,7.253

Oman,OMN,1954,7.253

Oman,OMN,1955,7.252

Oman,OMN,1956,7.251

Oman,OMN,1957,7.25

Oman,OMN,1958,7.249

Oman,OMN,1959,7.248

Oman,OMN,1960,7.247

Oman,OMN,1961,7.248

Oman,OMN,1962,7.251

Oman,OMN,1963,7.256

Oman,OMN,1964,7.263

Oman,OMN,1965,7.271

Oman,OMN,1966,7.277

Oman,OMN,1967,7.282

Oman,OMN,1968,7.286

Oman,OMN,1969,7.293

Oman,OMN,1970,7.311

Oman,OMN,1971,7.35

Oman,OMN,1972,7.414

Oman,OMN,1973,7.504

Oman,OMN,1974,7.617

Oman,OMN,1975,7.747

Oman,OMN,1976,7.885

Oman,OMN,1977,8.018

Oman,OMN,1978,8.137

Oman,OMN,1979,8.232

Oman,OMN,1980,8.299

Oman,OMN,1981,8.338

Oman,OMN,1982,8.352

Oman,OMN,1983,8.34

Oman,OMN,1984,8.299

Oman,OMN,1985,8.222

Oman,OMN,1986,8.101

Oman,OMN,1987,7.933

Oman,OMN,1988,7.719

Oman,OMN,1989,7.462

Oman,OMN,1990,7.165

Oman,OMN,1991,6.831

Oman,OMN,1992,6.472

Oman,OMN,1993,6.098

Oman,OMN,1994,5.72

Oman,OMN,1995,5.346

Oman,OMN,1996,4.981

Oman,OMN,1997,4.63

Oman,OMN,1998,4.297

Oman,OMN,1999,3.989

Oman,OMN,2000,3.716

Oman,OMN,2001,3.484

Oman,OMN,2002,3.293

Oman,OMN,2003,3.142

Oman,OMN,2004,3.029

Oman,OMN,2005,2.952

Oman,OMN,2006,2.909

Oman,OMN,2007,2.892

Oman,OMN,2008,2.89

Oman,OMN,2009,2.897

Oman,OMN,2010,2.901

Oman,OMN,2011,2.896

Oman,OMN,2012,2.878

Oman,OMN,2013,2.846

Oman,OMN,2014,2.799

Oman,OMN,2015,2.737

Pakistan,PAK,1950,6.6

Pakistan,PAK,1951,6.6

Pakistan,PAK,1952,6.6

Pakistan,PAK,1953,6.6

Pakistan,PAK,1954,6.6

Pakistan,PAK,1955,6.6

Pakistan,PAK,1956,6.6

Pakistan,PAK,1957,6.6

Pakistan,PAK,1958,6.6

Pakistan,PAK,1959,6.6

Pakistan,PAK,1960,6.6

Pakistan,PAK,1961,6.6

Pakistan,PAK,1962,6.6

Pakistan,PAK,1963,6.6

Pakistan,PAK,1964,6.6

Pakistan,PAK,1965,6.6

Pakistan,PAK,1966,6.6

Pakistan,PAK,1967,6.6

Pakistan,PAK,1968,6.6

Pakistan,PAK,1969,6.6

Pakistan,PAK,1970,6.601

Pakistan,PAK,1971,6.602

Pakistan,PAK,1972,6.605

Pakistan,PAK,1973,6.608

Pakistan,PAK,1974,6.611

Pakistan,PAK,1975,6.612

Pakistan,PAK,1976,6.608

Pakistan,PAK,1977,6.599

Pakistan,PAK,1978,6.583

Pakistan,PAK,1979,6.561

Pakistan,PAK,1980,6.535

Pakistan,PAK,1981,6.508

Pakistan,PAK,1982,6.483

Pakistan,PAK,1983,6.46

Pakistan,PAK,1984,6.436

Pakistan,PAK,1985,6.407

Pakistan,PAK,1986,6.367

Pakistan,PAK,1987,6.31

Pakistan,PAK,1988,6.234

Pakistan,PAK,1989,6.138

Pakistan,PAK,1990,6.024

Pakistan,PAK,1991,5.896

Pakistan,PAK,1992,5.76

Pakistan,PAK,1993,5.622

Pakistan,PAK,1994,5.482

Pakistan,PAK,1995,5.342

Pakistan,PAK,1996,5.198

Pakistan,PAK,1997,5.048

Pakistan,PAK,1998,4.892

Pakistan,PAK,1999,4.733

Pakistan,PAK,2000,4.58

Pakistan,PAK,2001,4.438

Pakistan,PAK,2002,4.314

Pakistan,PAK,2003,4.211

Pakistan,PAK,2004,4.129

Pakistan,PAK,2005,4.067

Pakistan,PAK,2006,4.02

Pakistan,PAK,2007,3.98

Pakistan,PAK,2008,3.942

Pakistan,PAK,2009,3.901

Pakistan,PAK,2010,3.855

Pakistan,PAK,2011,3.802

Pakistan,PAK,2012,3.744

Pakistan,PAK,2013,3.682

Pakistan,PAK,2014,3.617

Pakistan,PAK,2015,3.55

Palestine,PSE,1950,7.563

Palestine,PSE,1951,7.499

Palestine,PSE,1952,7.39

Palestine,PSE,1953,7.316

Palestine,PSE,1954,7.279

Palestine,PSE,1955,7.276

Palestine,PSE,1956,7.308

Palestine,PSE,1957,7.371

Palestine,PSE,1958,7.461

Palestine,PSE,1959,7.569

Palestine,PSE,1960,7.687

Palestine,PSE,1961,7.803

Palestine,PSE,1962,7.907

Palestine,PSE,1963,7.988

Palestine,PSE,1964,8.041

Palestine,PSE,1965,8.063

Palestine,PSE,1966,8.054

Palestine,PSE,1967,8.023

Palestine,PSE,1968,7.977

Palestine,PSE,1969,7.921

Palestine,PSE,1970,7.862

Palestine,PSE,1971,7.804

Palestine,PSE,1972,7.749

Palestine,PSE,1973,7.699

Palestine,PSE,1974,7.654

Palestine,PSE,1975,7.611

Palestine,PSE,1976,7.565

Palestine,PSE,1977,7.51

Palestine,PSE,1978,7.445

Palestine,PSE,1979,7.368

Palestine,PSE,1980,7.284

Palestine,PSE,1981,7.194

Palestine,PSE,1982,7.105

Palestine,PSE,1983,7.022

Palestine,PSE,1984,6.947

Palestine,PSE,1985,6.883

Palestine,PSE,1986,6.835

Palestine,PSE,1987,6.799

Palestine,PSE,1988,6.772

Palestine,PSE,1989,6.748

Palestine,PSE,1990,6.718

Palestine,PSE,1991,6.674

Palestine,PSE,1992,6.607

Palestine,PSE,1993,6.515

Palestine,PSE,1994,6.396

Palestine,PSE,1995,6.251

Palestine,PSE,1996,6.085

Palestine,PSE,1997,5.907

Palestine,PSE,1998,5.727

Palestine,PSE,1999,5.55

Palestine,PSE,2000,5.383

Palestine,PSE,2001,5.231

Palestine,PSE,2002,5.094

Palestine,PSE,2003,4.973

Palestine,PSE,2004,4.867

Palestine,PSE,2005,4.775

Palestine,PSE,2006,4.695

Palestine,PSE,2007,4.623

Palestine,PSE,2008,4.554

Palestine,PSE,2009,4.487

Palestine,PSE,2010,4.419

Palestine,PSE,2011,4.35

Palestine,PSE,2012,4.281

Palestine,PSE,2013,4.212

Palestine,PSE,2014,4.143

Palestine,PSE,2015,4.075

Panama,PAN,1950,5.679

Panama,PAN,1951,5.704

Panama,PAN,1952,5.749

Panama,PAN,1953,5.789

Panama,PAN,1954,5.821

Panama,PAN,1955,5.848

Panama,PAN,1956,5.868

Panama,PAN,1957,5.88

Panama,PAN,1958,5.885

Panama,PAN,1959,5.882

Panama,PAN,1960,5.87

Panama,PAN,1961,5.846

Panama,PAN,1962,5.812

Panama,PAN,1963,5.765

Panama,PAN,1964,5.707

Panama,PAN,1965,5.638

Panama,PAN,1966,5.559

Panama,PAN,1967,5.472

Panama,PAN,1968,5.378

Panama,PAN,1969,5.279

Panama,PAN,1970,5.173

Panama,PAN,1971,5.06

Panama,PAN,1972,4.939

Panama,PAN,1973,4.81

Panama,PAN,1974,4.675

Panama,PAN,1975,4.537

Panama,PAN,1976,4.398

Panama,PAN,1977,4.26

Panama,PAN,1978,4.128

Panama,PAN,1979,4.002

Panama,PAN,1980,3.884

Panama,PAN,1981,3.775

Panama,PAN,1982,3.675

Panama,PAN,1983,3.581

Panama,PAN,1984,3.494

Panama,PAN,1985,3.413

Panama,PAN,1986,3.336

Panama,PAN,1987,3.262

Panama,PAN,1988,3.19

Panama,PAN,1989,3.121

Panama,PAN,1990,3.057

Panama,PAN,1991,2.999

Panama,PAN,1992,2.95

Panama,PAN,1993,2.909

Panama,PAN,1994,2.877

Panama,PAN,1995,2.852

Panama,PAN,1996,2.83

Panama,PAN,1997,2.81

Panama,PAN,1998,2.789

Panama,PAN,1999,2.767

Panama,PAN,2000,2.744

Panama,PAN,2001,2.72

Panama,PAN,2002,2.698

Panama,PAN,2003,2.679

Panama,PAN,2004,2.663

Panama,PAN,2005,2.651

Panama,PAN,2006,2.643

Panama,PAN,2007,2.637

Panama,PAN,2008,2.632

Panama,PAN,2009,2.628

Panama,PAN,2010,2.622

Panama,PAN,2011,2.613

Panama,PAN,2012,2.601

Panama,PAN,2013,2.585

Panama,PAN,2014,2.565

Panama,PAN,2015,2.541

Papua New Guinea,PNG,1950,6.231

Papua New Guinea,PNG,1951,6.233

Papua New Guinea,PNG,1952,6.236

Papua New Guinea,PNG,1953,6.239

Papua New Guinea,PNG,1954,6.244

Papua New Guinea,PNG,1955,6.248

Papua New Guinea,PNG,1956,6.254

Papua New Guinea,PNG,1957,6.26

Papua New Guinea,PNG,1958,6.266

Papua New Guinea,PNG,1959,6.271

Papua New Guinea,PNG,1960,6.275

Papua New Guinea,PNG,1961,6.278

Papua New Guinea,PNG,1962,6.278

Papua New Guinea,PNG,1963,6.275

Papua New Guinea,PNG,1964,6.268

Papua New Guinea,PNG,1965,6.258

Papua New Guinea,PNG,1966,6.244

Papua New Guinea,PNG,1967,6.227

Papua New Guinea,PNG,1968,6.208

Papua New Guinea,PNG,1969,6.187

Papua New Guinea,PNG,1970,6.163

Papua New Guinea,PNG,1971,6.137

Papua New Guinea,PNG,1972,6.108

Papua New Guinea,PNG,1973,6.077

Papua New Guinea,PNG,1974,6.041

Papua New Guinea,PNG,1975,6

Papua New Guinea,PNG,1976,5.954

Papua New Guinea,PNG,1977,5.9

Papua New Guinea,PNG,1978,5.839

Papua New Guinea,PNG,1979,5.771

Papua New Guinea,PNG,1980,5.694

Papua New Guinea,PNG,1981,5.609

Papua New Guinea,PNG,1982,5.515

Papua New Guinea,PNG,1983,5.415

Papua New Guinea,PNG,1984,5.312

Papua New Guinea,PNG,1985,5.21

Papua New Guinea,PNG,1986,5.11

Papua New Guinea,PNG,1987,5.018

Papua New Guinea,PNG,1988,4.934

Papua New Guinea,PNG,1989,4.861

Papua New Guinea,PNG,1990,4.802

Papua New Guinea,PNG,1991,4.756

Papua New Guinea,PNG,1992,4.723

Papua New Guinea,PNG,1993,4.7

Papua New Guinea,PNG,1994,4.683

Papua New Guinea,PNG,1995,4.668

Papua New Guinea,PNG,1996,4.653

Papua New Guinea,PNG,1997,4.632

Papua New Guinea,PNG,1998,4.604

Papua New Guinea,PNG,1999,4.569

Papua New Guinea,PNG,2000,4.525

Papua New Guinea,PNG,2001,4.475

Papua New Guinea,PNG,2002,4.422

Papua New Guinea,PNG,2003,4.368

Papua New Guinea,PNG,2004,4.315

Papua New Guinea,PNG,2005,4.263

Papua New Guinea,PNG,2006,4.21

Papua New Guinea,PNG,2007,4.156

Papua New Guinea,PNG,2008,4.101

Papua New Guinea,PNG,2009,4.043

Papua New Guinea,PNG,2010,3.985

Papua New Guinea,PNG,2011,3.926

Papua New Guinea,PNG,2012,3.869

Papua New Guinea,PNG,2013,3.813

Papua New Guinea,PNG,2014,3.759

Papua New Guinea,PNG,2015,3.707

Paraguay,PRY,1950,6.49

Paraguay,PRY,1951,6.493

Paraguay,PRY,1952,6.499

Paraguay,PRY,1953,6.503

Paraguay,PRY,1954,6.506

Paraguay,PRY,1955,6.509

Paraguay,PRY,1956,6.51

Paraguay,PRY,1957,6.511

Paraguay,PRY,1958,6.509

Paraguay,PRY,1959,6.506

Paraguay,PRY,1960,6.5

Paraguay,PRY,1961,6.489

Paraguay,PRY,1962,6.472

Paraguay,PRY,1963,6.447

Paraguay,PRY,1964,6.41

Paraguay,PRY,1965,6.356

Paraguay,PRY,1966,6.276

Paraguay,PRY,1967,6.17

Paraguay,PRY,1968,6.041

Paraguay,PRY,1969,5.894

Paraguay,PRY,1970,5.739

Paraguay,PRY,1971,5.591

Paraguay,PRY,1972,5.458

Paraguay,PRY,1973,5.351

Paraguay,PRY,1974,5.272

Paraguay,PRY,1975,5.223

Paraguay,PRY,1976,5.198

Paraguay,PRY,1977,5.189

Paraguay,PRY,1978,5.186

Paraguay,PRY,1979,5.183

Paraguay,PRY,1980,5.174

Paraguay,PRY,1981,5.155

Paraguay,PRY,1982,5.126

Paraguay,PRY,1983,5.087

Paraguay,PRY,1984,5.037

Paraguay,PRY,1985,4.974

Paraguay,PRY,1986,4.9

Paraguay,PRY,1987,4.818

Paraguay,PRY,1988,4.73

Paraguay,PRY,1989,4.639

Paraguay,PRY,1990,4.547

Paraguay,PRY,1991,4.457

Paraguay,PRY,1992,4.368

Paraguay,PRY,1993,4.282

Paraguay,PRY,1994,4.196

Paraguay,PRY,1995,4.108

Paraguay,PRY,1996,4.014

Paraguay,PRY,1997,3.911

Paraguay,PRY,1998,3.798

Paraguay,PRY,1999,3.678

Paraguay,PRY,2000,3.553

Paraguay,PRY,2001,3.431

Paraguay,PRY,2002,3.315

Paraguay,PRY,2003,3.21

Paraguay,PRY,2004,3.119

Paraguay,PRY,2005,3.04

Paraguay,PRY,2006,2.97

Paraguay,PRY,2007,2.907

Paraguay,PRY,2008,2.846

Paraguay,PRY,2009,2.787

Paraguay,PRY,2010,2.73

Paraguay,PRY,2011,2.675

Paraguay,PRY,2012,2.625

Paraguay,PRY,2013,2.581

Paraguay,PRY,2014,2.542

Paraguay,PRY,2015,2.509

Peru,PER,1950,6.954

Peru,PER,1951,6.952

Peru,PER,1952,6.949

Peru,PER,1953,6.948

Peru,PER,1954,6.948

Peru,PER,1955,6.95

Peru,PER,1956,6.954

Peru,PER,1957,6.958

Peru,PER,1958,6.963

Peru,PER,1959,6.968

Peru,PER,1960,6.971

Peru,PER,1961,6.97

Peru,PER,1962,6.963

Peru,PER,1963,6.948

Peru,PER,1964,6.921

Peru,PER,1965,6.878

Peru,PER,1966,6.816

Peru,PER,1967,6.733

Peru,PER,1968,6.63

Peru,PER,1969,6.51

Peru,PER,1970,6.378

Peru,PER,1971,6.239

Peru,PER,1972,6.099

Peru,PER,1973,5.963

Peru,PER,1974,5.832

Peru,PER,1975,5.705

Peru,PER,1976,5.578

Peru,PER,1977,5.447

Peru,PER,1978,5.31

Peru,PER,1979,5.167

Peru,PER,1980,5.021

Peru,PER,1981,4.875

Peru,PER,1982,4.733

Peru,PER,1983,4.6

Peru,PER,1984,4.475

Peru,PER,1985,4.358

Peru,PER,1986,4.249

Peru,PER,1987,4.142

Peru,PER,1988,4.037

Peru,PER,1989,3.933

Peru,PER,1990,3.828

Peru,PER,1991,3.722

Peru,PER,1992,3.617

Peru,PER,1993,3.514

Peru,PER,1994,3.413

Peru,PER,1995,3.317

Peru,PER,1996,3.226

Peru,PER,1997,3.142

Peru,PER,1998,3.064

Peru,PER,1999,2.993

Peru,PER,2000,2.929

Peru,PER,2001,2.871

Peru,PER,2002,2.818

Peru,PER,2003,2.77

Peru,PER,2004,2.725

Peru,PER,2005,2.684

Peru,PER,2006,2.648

Peru,PER,2007,2.616

Peru,PER,2008,2.589

Peru,PER,2009,2.566

Peru,PER,2010,2.545

Peru,PER,2011,2.525

Peru,PER,2012,2.504

Peru,PER,2013,2.481

Peru,PER,2014,2.456

Peru,PER,2015,2.429

Philippines,PHL,1950,7.448

Philippines,PHL,1951,7.444

Philippines,PHL,1952,7.432

Philippines,PHL,1953,7.416

Philippines,PHL,1954,7.394

Philippines,PHL,1955,7.366

Philippines,PHL,1956,7.334

Philippines,PHL,1957,7.296

Philippines,PHL,1958,7.252

Philippines,PHL,1959,7.203

Philippines,PHL,1960,7.148

Philippines,PHL,1961,7.087

Philippines,PHL,1962,7.02

Philippines,PHL,1963,6.947

Philippines,PHL,1964,6.867

Philippines,PHL,1965,6.781

Philippines,PHL,1966,6.689

Philippines,PHL,1967,6.589

Philippines,PHL,1968,6.485

Philippines,PHL,1969,6.376

Philippines,PHL,1970,6.264

Philippines,PHL,1971,6.152

Philippines,PHL,1972,6.041

Philippines,PHL,1973,5.931

Philippines,PHL,1974,5.824

Philippines,PHL,1975,5.718

Philippines,PHL,1976,5.613

Philippines,PHL,1977,5.506

Philippines,PHL,1978,5.399

Philippines,PHL,1979,5.29

Philippines,PHL,1980,5.183

Philippines,PHL,1981,5.078

Philippines,PHL,1982,4.978

Philippines,PHL,1983,4.883

Philippines,PHL,1984,4.795

Philippines,PHL,1985,4.712

Philippines,PHL,1986,4.632

Philippines,PHL,1987,4.554

Philippines,PHL,1988,4.476

Philippines,PHL,1989,4.397

Philippines,PHL,1990,4.32

Philippines,PHL,1991,4.246

Philippines,PHL,1992,4.177

Philippines,PHL,1993,4.113

Philippines,PHL,1994,4.056

Philippines,PHL,1995,4.005

Philippines,PHL,1996,3.961

Philippines,PHL,1997,3.922

Philippines,PHL,1998,3.886

Philippines,PHL,1999,3.85

Philippines,PHL,2000,3.811

Philippines,PHL,2001,3.766

Philippines,PHL,2002,3.712

Philippines,PHL,2003,3.65

Philippines,PHL,2004,3.579

Philippines,PHL,2005,3.503

Philippines,PHL,2006,3.425

Philippines,PHL,2007,3.349

Philippines,PHL,2008,3.278

Philippines,PHL,2009,3.214

Philippines,PHL,2010,3.158

Philippines,PHL,2011,3.109

Philippines,PHL,2012,3.067

Philippines,PHL,2013,3.028

Philippines,PHL,2014,2.992

Philippines,PHL,2015,2.958

Poland,POL,1950,3.527

Poland,POL,1951,3.569

Poland,POL,1952,3.636

Poland,POL,1953,3.672

Poland,POL,1954,3.676

Poland,POL,1955,3.648

Poland,POL,1956,3.589

Poland,POL,1957,3.502

Poland,POL,1958,3.389

Poland,POL,1959,3.257

Poland,POL,1960,3.113

Poland,POL,1961,2.964

Poland,POL,1962,2.819

Poland,POL,1963,2.687

Poland,POL,1964,2.572

Poland,POL,1965,2.479

Poland,POL,1966,2.407

Poland,POL,1967,2.352

Poland,POL,1968,2.31

Poland,POL,1969,2.278

Poland,POL,1970,2.254

Poland,POL,1971,2.238

Poland,POL,1972,2.227

Poland,POL,1973,2.221

Poland,POL,1974,2.217

Poland,POL,1975,2.217

Poland,POL,1976,2.222

Poland,POL,1977,2.232

Poland,POL,1978,2.247

Poland,POL,1979,2.265

Poland,POL,1980,2.282

Poland,POL,1981,2.295

Poland,POL,1982,2.301

Poland,POL,1983,2.298

Poland,POL,1984,2.284

Poland,POL,1985,2.261

Poland,POL,1986,2.231

Poland,POL,1987,2.197

Poland,POL,1988,2.16

Poland,POL,1989,2.121

Poland,POL,1990,2.077

Poland,POL,1991,2.026

Poland,POL,1992,1.966

Poland,POL,1993,1.896

Poland,POL,1994,1.818

Poland,POL,1995,1.733

Poland,POL,1996,1.646

Poland,POL,1997,1.559

Poland,POL,1998,1.477

Poland,POL,1999,1.405

Poland,POL,2000,1.346

Poland,POL,2001,1.305

Poland,POL,2002,1.282

Poland,POL,2003,1.275

Poland,POL,2004,1.282

Poland,POL,2005,1.299

Poland,POL,2006,1.321

Poland,POL,2007,1.342

Poland,POL,2008,1.357

Poland,POL,2009,1.365

Poland,POL,2010,1.365

Poland,POL,2011,1.357

Poland,POL,2012,1.344

Poland,POL,2013,1.332

Poland,POL,2014,1.319

Poland,POL,2015,1.308

Polynesia,OWID_PYA,1950,6.72

Polynesia,OWID_PYA,1951,6.735

Polynesia,OWID_PYA,1952,6.764

Polynesia,OWID_PYA,1953,6.79

Polynesia,OWID_PYA,1954,6.815

Polynesia,OWID_PYA,1955,6.836

Polynesia,OWID_PYA,1956,6.855

Polynesia,OWID_PYA,1957,6.871

Polynesia,OWID_PYA,1958,6.881

Polynesia,OWID_PYA,1959,6.885

Polynesia,OWID_PYA,1960,6.881

Polynesia,OWID_PYA,1961,6.864

Polynesia,OWID_PYA,1962,6.834

Polynesia,OWID_PYA,1963,6.789

Polynesia,OWID_PYA,1964,6.727

Polynesia,OWID_PYA,1965,6.649

Polynesia,OWID_PYA,1966,6.555

Polynesia,OWID_PYA,1967,6.446

Polynesia,OWID_PYA,1968,6.328

Polynesia,OWID_PYA,1969,6.202

Polynesia,OWID_PYA,1970,6.073

Polynesia,OWID_PYA,1971,5.945

Polynesia,OWID_PYA,1972,5.818

Polynesia,OWID_PYA,1973,5.696

Polynesia,OWID_PYA,1974,5.58

Polynesia,OWID_PYA,1975,5.471

Polynesia,OWID_PYA,1976,5.369

Polynesia,OWID_PYA,1977,5.273

Polynesia,OWID_PYA,1978,5.18

Polynesia,OWID_PYA,1979,5.09

Polynesia,OWID_PYA,1980,5.002

Polynesia,OWID_PYA,1981,4.916

Polynesia,OWID_PYA,1982,4.831

Polynesia,OWID_PYA,1983,4.747

Polynesia,OWID_PYA,1984,4.663

Polynesia,OWID_PYA,1985,4.58

Polynesia,OWID_PYA,1986,4.496

Polynesia,OWID_PYA,1987,4.412

Polynesia,OWID_PYA,1988,4.329

Polynesia,OWID_PYA,1989,4.246

Polynesia,OWID_PYA,1990,4.163

Polynesia,OWID_PYA,1991,4.079

Polynesia,OWID_PYA,1992,3.993

Polynesia,OWID_PYA,1993,3.908

Polynesia,OWID_PYA,1994,3.824

Polynesia,OWID_PYA,1995,3.742

Polynesia,OWID_PYA,1996,3.664

Polynesia,OWID_PYA,1997,3.591

Polynesia,OWID_PYA,1998,3.526

Polynesia,OWID_PYA,1999,3.467

Polynesia,OWID_PYA,2000,3.416

Polynesia,OWID_PYA,2001,3.372

Polynesia,OWID_PYA,2002,3.335

Polynesia,OWID_PYA,2003,3.301

Polynesia,OWID_PYA,2004,3.27

Polynesia,OWID_PYA,2005,3.241

Polynesia,OWID_PYA,2006,3.211

Polynesia,OWID_PYA,2007,3.179

Polynesia,OWID_PYA,2008,3.146

Polynesia,OWID_PYA,2009,3.11

Polynesia,OWID_PYA,2010,3.072

Polynesia,OWID_PYA,2011,3.032

Polynesia,OWID_PYA,2012,2.993

Polynesia,OWID_PYA,2013,2.954

Polynesia,OWID_PYA,2014,2.917

Polynesia,OWID_PYA,2015,2.881

Portugal,PRT,1950,3.104

Portugal,PRT,1951,3.101

Portugal,PRT,1952,3.097

Portugal,PRT,1953,3.096

Portugal,PRT,1954,3.098

Portugal,PRT,1955,3.103

Portugal,PRT,1956,3.111

Portugal,PRT,1957,3.122

Portugal,PRT,1958,3.134

Portugal,PRT,1959,3.148

Portugal,PRT,1960,3.162

Portugal,PRT,1961,3.175

Portugal,PRT,1962,3.185

Portugal,PRT,1963,3.19

Portugal,PRT,1964,3.189

Portugal,PRT,1965,3.18

Portugal,PRT,1966,3.161

Portugal,PRT,1967,3.132

Portugal,PRT,1968,3.093

Portugal,PRT,1969,3.047

Portugal,PRT,1970,2.994

Portugal,PRT,1971,2.938

Portugal,PRT,1972,2.882

Portugal,PRT,1973,2.825

Portugal,PRT,1974,2.769

Portugal,PRT,1975,2.711

Portugal,PRT,1976,2.646

Portugal,PRT,1977,2.571

Portugal,PRT,1978,2.485

Portugal,PRT,1979,2.389

Portugal,PRT,1980,2.286

Portugal,PRT,1981,2.177

Portugal,PRT,1982,2.069

Portugal,PRT,1983,1.965

Portugal,PRT,1984,1.87

Portugal,PRT,1985,1.784

Portugal,PRT,1986,1.711

Portugal,PRT,1987,1.649

Portugal,PRT,1988,1.597

Portugal,PRT,1989,1.555

Portugal,PRT,1990,1.522

Portugal,PRT,1991,1.498

Portugal,PRT,1992,1.481

Portugal,PRT,1993,1.469

Portugal,PRT,1994,1.461

Portugal,PRT,1995,1.456

Portugal,PRT,1996,1.454

Portugal,PRT,1997,1.454

Portugal,PRT,1998,1.455

Portugal,PRT,1999,1.457

Portugal,PRT,2000,1.457

Portugal,PRT,2001,1.455

Portugal,PRT,2002,1.45

Portugal,PRT,2003,1.443

Portugal,PRT,2004,1.432

Portugal,PRT,2005,1.418

Portugal,PRT,2006,1.401

Portugal,PRT,2007,1.382

Portugal,PRT,2008,1.363

Portugal,PRT,2009,1.343

Portugal,PRT,2010,1.325

Portugal,PRT,2011,1.307

Portugal,PRT,2012,1.291

Portugal,PRT,2013,1.277

Portugal,PRT,2014,1.264

Portugal,PRT,2015,1.254

Puerto Rico,PRI,1950,4.975

Puerto Rico,PRI,1951,4.978

Puerto Rico,PRI,1952,4.979

Puerto Rico,PRI,1953,4.973

Puerto Rico,PRI,1954,4.959

Puerto Rico,PRI,1955,4.937

Puerto Rico,PRI,1956,4.906

Puerto Rico,PRI,1957,4.866

Puerto Rico,PRI,1958,4.813

Puerto Rico,PRI,1959,4.745

Puerto Rico,PRI,1960,4.657

Puerto Rico,PRI,1961,4.547

Puerto Rico,PRI,1962,4.411

Puerto Rico,PRI,1963,4.252

Puerto Rico,PRI,1964,4.075

Puerto Rico,PRI,1965,3.888

Puerto Rico,PRI,1966,3.703

Puerto Rico,PRI,1967,3.53

Puerto Rico,PRI,1968,3.377

Puerto Rico,PRI,1969,3.248

Puerto Rico,PRI,1970,3.146

Puerto Rico,PRI,1971,3.068

Puerto Rico,PRI,1972,3.006

Puerto Rico,PRI,1973,2.955

Puerto Rico,PRI,1974,2.908

Puerto Rico,PRI,1975,2.864

Puerto Rico,PRI,1976,2.818

Puerto Rico,PRI,1977,2.769

Puerto Rico,PRI,1978,2.718

Puerto Rico,PRI,1979,2.664

Puerto Rico,PRI,1980,2.607

Puerto Rico,PRI,1981,2.548

Puerto Rico,PRI,1982,2.49

Puerto Rico,PRI,1983,2.436

Puerto Rico,PRI,1984,2.387

Puerto Rico,PRI,1985,2.344

Puerto Rico,PRI,1986,2.308

Puerto Rico,PRI,1987,2.279

Puerto Rico,PRI,1988,2.256

Puerto Rico,PRI,1989,2.236

Puerto Rico,PRI,1990,2.219

Puerto Rico,PRI,1991,2.201

Puerto Rico,PRI,1992,2.179

Puerto Rico,PRI,1993,2.153

Puerto Rico,PRI,1994,2.122

Puerto Rico,PRI,1995,2.086

Puerto Rico,PRI,1996,2.049

Puerto Rico,PRI,1997,2.011

Puerto Rico,PRI,1998,1.975

Puerto Rico,PRI,1999,1.943

Puerto Rico,PRI,2000,1.913

Puerto Rico,PRI,2001,1.887

Puerto Rico,PRI,2002,1.863

Puerto Rico,PRI,2003,1.839

Puerto Rico,PRI,2004,1.815

Puerto Rico,PRI,2005,1.789

Puerto Rico,PRI,2006,1.76

Puerto Rico,PRI,2007,1.728

Puerto Rico,PRI,2008,1.691

Puerto Rico,PRI,2009,1.653

Puerto Rico,PRI,2010,1.614

Puerto Rico,PRI,2011,1.577

Puerto Rico,PRI,2012,1.543

Puerto Rico,PRI,2013,1.516

Puerto Rico,PRI,2014,1.495

Puerto Rico,PRI,2015,1.481

Qatar,QAT,1950,6.968

Qatar,QAT,1951,6.968

Qatar,QAT,1952,6.97

Qatar,QAT,1953,6.971

Qatar,QAT,1954,6.971

Qatar,QAT,1955,6.971

Qatar,QAT,1956,6.971

Qatar,QAT,1957,6.97

Qatar,QAT,1958,6.969

Qatar,QAT,1959,6.969

Qatar,QAT,1960,6.971

Qatar,QAT,1961,6.973

Qatar,QAT,1962,6.977

Qatar,QAT,1963,6.982

Qatar,QAT,1964,6.986

Qatar,QAT,1965,6.988

Qatar,QAT,1966,6.988

Qatar,QAT,1967,6.983

Qatar,QAT,1968,6.97

Qatar,QAT,1969,6.947

Qatar,QAT,1970,6.91

Qatar,QAT,1971,6.855

Qatar,QAT,1972,6.781

Qatar,QAT,1973,6.69

Qatar,QAT,1974,6.581

Qatar,QAT,1975,6.46

Qatar,QAT,1976,6.332

Qatar,QAT,1977,6.201

Qatar,QAT,1978,6.07

Qatar,QAT,1979,5.94

Qatar,QAT,1980,5.806

Qatar,QAT,1981,5.661

Qatar,QAT,1982,5.5

Qatar,QAT,1983,5.322

Qatar,QAT,1984,5.128

Qatar,QAT,1985,4.925

Qatar,QAT,1986,4.718

Qatar,QAT,1987,4.516

Qatar,QAT,1988,4.329

Qatar,QAT,1989,4.159

Qatar,QAT,1990,4.013

Qatar,QAT,1991,3.893

Qatar,QAT,1992,3.795

Qatar,QAT,1993,3.714

Qatar,QAT,1994,3.647

Qatar,QAT,1995,3.588

Qatar,QAT,1996,3.532

Qatar,QAT,1997,3.473

Qatar,QAT,1998,3.407

Qatar,QAT,1999,3.329

Qatar,QAT,2000,3.236

Qatar,QAT,2001,3.124

Qatar,QAT,2002,2.996

Qatar,QAT,2003,2.858

Qatar,QAT,2004,2.713

Qatar,QAT,2005,2.569

Qatar,QAT,2006,2.433

Qatar,QAT,2007,2.312

Qatar,QAT,2008,2.211

Qatar,QAT,2009,2.13

Qatar,QAT,2010,2.07

Qatar,QAT,2011,2.027

Qatar,QAT,2012,1.997

Qatar,QAT,2013,1.972

Qatar,QAT,2014,1.95

Qatar,QAT,2015,1.929

Reunion,REU,1950,7.19

Reunion,REU,1951,7.112

Reunion,REU,1952,6.97

Reunion,REU,1953,6.852

Reunion,REU,1954,6.759

Reunion,REU,1955,6.69

Reunion,REU,1956,6.644

Reunion,REU,1957,6.618

Reunion,REU,1958,6.609

Reunion,REU,1959,6.609

Reunion,REU,1960,6.609

Reunion,REU,1961,6.6

Reunion,REU,1962,6.569

Reunion,REU,1963,6.506

Reunion,REU,1964,6.404

Reunion,REU,1965,6.251

Reunion,REU,1966,6.038

Reunion,REU,1967,5.77

Reunion,REU,1968,5.46

Reunion,REU,1969,5.119

Reunion,REU,1970,4.765

Reunion,REU,1971,4.418

Reunion,REU,1972,4.096

Reunion,REU,1973,3.812

Reunion,REU,1974,3.575

Reunion,REU,1975,3.388

Reunion,REU,1976,3.245

Reunion,REU,1977,3.133

Reunion,REU,1978,3.04

Reunion,REU,1979,2.96

Reunion,REU,1980,2.893

Reunion,REU,1981,2.838

Reunion,REU,1982,2.798

Reunion,REU,1983,2.772

Reunion,REU,1984,2.754

Reunion,REU,1985,2.74

Reunion,REU,1986,2.722

Reunion,REU,1987,2.696

Reunion,REU,1988,2.66

Reunion,REU,1989,2.613

Reunion,REU,1990,2.559

Reunion,REU,1991,2.5

Reunion,REU,1992,2.444

Reunion,REU,1993,2.396

Reunion,REU,1994,2.359

Reunion,REU,1995,2.335

Reunion,REU,1996,2.326

Reunion,REU,1997,2.33

Reunion,REU,1998,2.342

Reunion,REU,1999,2.361

Reunion,REU,2000,2.383

Reunion,REU,2001,2.404

Reunion,REU,2002,2.42

Reunion,REU,2003,2.43

Reunion,REU,2004,2.433

Reunion,REU,2005,2.429

Reunion,REU,2006,2.422

Reunion,REU,2007,2.415

Reunion,REU,2008,2.41

Reunion,REU,2009,2.407

Reunion,REU,2010,2.405

Reunion,REU,2011,2.402

Reunion,REU,2012,2.396

Reunion,REU,2013,2.384

Reunion,REU,2014,2.367

Reunion,REU,2015,2.345

Romania,ROU,1950,3.046

Romania,ROU,1951,3.065

Romania,ROU,1952,3.089

Romania,ROU,1953,3.079

Romania,ROU,1954,3.037

Romania,ROU,1955,2.964

Romania,ROU,1956,2.862

Romania,ROU,1957,2.737

Romania,ROU,1958,2.597

Romania,ROU,1959,2.458

Romania,ROU,1960,2.338

Romania,ROU,1961,2.254

Romania,ROU,1962,2.223

Romania,ROU,1963,2.25

Romania,ROU,1964,2.333

Romania,ROU,1965,2.455

Romania,ROU,1966,2.589

Romania,ROU,1967,2.708

Romania,ROU,1968,2.791

Romania,ROU,1969,2.829

Romania,ROU,1970,2.824

Romania,ROU,1971,2.786

Romania,ROU,1972,2.734

Romania,ROU,1973,2.685

Romania,ROU,1974,2.642

Romania,ROU,1975,2.608

Romania,ROU,1976,2.577

Romania,ROU,1977,2.542

Romania,ROU,1978,2.499

Romania,ROU,1979,2.45

Romania,ROU,1980,2.399

Romania,ROU,1981,2.355

Romania,ROU,1982,2.321

Romania,ROU,1983,2.3

Romania,ROU,1984,2.288

Romania,ROU,1985,2.274

Romania,ROU,1986,2.246

Romania,ROU,1987,2.193

Romania,ROU,1988,2.111

Romania,ROU,1989,2.002

Romania,ROU,1990,1.874

Romania,ROU,1991,1.739

Romania,ROU,1992,1.613

Romania,ROU,1993,1.506

Romania,ROU,1994,1.425

Romania,ROU,1995,1.37

Romania,ROU,1996,1.339

Romania,ROU,1997,1.322

Romania,ROU,1998,1.312

Romania,ROU,1999,1.306

Romania,ROU,2000,1.305

Romania,ROU,2001,1.308

Romania,ROU,2002,1.318

Romania,ROU,2003,1.334

Romania,ROU,2004,1.355

Romania,ROU,2005,1.379

Romania,ROU,2006,1.404

Romania,ROU,2007,1.426

Romania,ROU,2008,1.445

Romania,ROU,2009,1.46

Romania,ROU,2010,1.471

Romania,ROU,2011,1.479

Romania,ROU,2012,1.486

Romania,ROU,2013,1.493

Romania,ROU,2014,1.502

Romania,ROU,2015,1.512

Russia,RUS,1950,2.814

Russia,RUS,1951,2.827

Russia,RUS,1952,2.848

Russia,RUS,1953,2.863

Russia,RUS,1954,2.87

Russia,RUS,1955,2.869

Russia,RUS,1956,2.86

Russia,RUS,1957,2.843

Russia,RUS,1958,2.815

Russia,RUS,1959,2.776

Russia,RUS,1960,2.723

Russia,RUS,1961,2.655

Russia,RUS,1962,2.571

Russia,RUS,1963,2.474

Russia,RUS,1964,2.369

Russia,RUS,1965,2.265

Russia,RUS,1966,2.17

Russia,RUS,1967,2.093

Russia,RUS,1968,2.038

Russia,RUS,1969,2.005

Russia,RUS,1970,1.991

Russia,RUS,1971,1.991

Russia,RUS,1972,1.993

Russia,RUS,1973,1.993

Russia,RUS,1974,1.987

Russia,RUS,1975,1.976

Russia,RUS,1976,1.964

Russia,RUS,1977,1.956

Russia,RUS,1978,1.955

Russia,RUS,1979,1.963

Russia,RUS,1980,1.979

Russia,RUS,1981,2.006

Russia,RUS,1982,2.041

Russia,RUS,1983,2.077

Russia,RUS,1984,2.11

Russia,RUS,1985,2.131

Russia,RUS,1986,2.129

Russia,RUS,1987,2.101

Russia,RUS,1988,2.046

Russia,RUS,1989,1.964

Russia,RUS,1990,1.861

Russia,RUS,1991,1.745

Russia,RUS,1992,1.628

Russia,RUS,1993,1.519

Russia,RUS,1994,1.425

Russia,RUS,1995,1.35

Russia,RUS,1996,1.296

Russia,RUS,1997,1.261

Russia,RUS,1998,1.24

Russia,RUS,1999,1.233

Russia,RUS,2000,1.238

Russia,RUS,2001,1.251

Russia,RUS,2002,1.271

Russia,RUS,2003,1.295

Russia,RUS,2004,1.322

Russia,RUS,2005,1.352

Russia,RUS,2006,1.387

Russia,RUS,2007,1.427

Russia,RUS,2008,1.474

Russia,RUS,2009,1.524

Russia,RUS,2010,1.575

Russia,RUS,2011,1.623

Russia,RUS,2012,1.665

Russia,RUS,2013,1.699

Russia,RUS,2014,1.724

Russia,RUS,2015,1.741

Rwanda,RWA,1950,7.916

Rwanda,RWA,1951,7.939

Rwanda,RWA,1952,7.983

Rwanda,RWA,1953,8.023

Rwanda,RWA,1954,8.059

Rwanda,RWA,1955,8.09

Rwanda,RWA,1956,8.118

Rwanda,RWA,1957,8.141

Rwanda,RWA,1958,8.161

Rwanda,RWA,1959,8.176

Rwanda,RWA,1960,8.187

Rwanda,RWA,1961,8.194

Rwanda,RWA,1962,8.197

Rwanda,RWA,1963,8.198

Rwanda,RWA,1964,8.198

Rwanda,RWA,1965,8.198

Rwanda,RWA,1966,8.198

Rwanda,RWA,1967,8.201

Rwanda,RWA,1968,8.207

Rwanda,RWA,1969,8.217

Rwanda,RWA,1970,8.231

Rwanda,RWA,1971,8.252

Rwanda,RWA,1972,8.278

Rwanda,RWA,1973,8.307

Rwanda,RWA,1974,8.339

Rwanda,RWA,1975,8.37

Rwanda,RWA,1976,8.401

Rwanda,RWA,1977,8.429

Rwanda,RWA,1978,8.451

Rwanda,RWA,1979,8.462

Rwanda,RWA,1980,8.461

Rwanda,RWA,1981,8.444

Rwanda,RWA,1982,8.41

Rwanda,RWA,1983,8.357

Rwanda,RWA,1984,8.282

Rwanda,RWA,1985,8.177

Rwanda,RWA,1986,8.038

Rwanda,RWA,1987,7.863

Rwanda,RWA,1988,7.657

Rwanda,RWA,1989,7.427

Rwanda,RWA,1990,7.184

Rwanda,RWA,1991,6.942

Rwanda,RWA,1992,6.713

Rwanda,RWA,1993,6.505

Rwanda,RWA,1994,6.324

Rwanda,RWA,1995,6.172

Rwanda,RWA,1996,6.046

Rwanda,RWA,1997,5.936

Rwanda,RWA,1998,5.835

Rwanda,RWA,1999,5.737

Rwanda,RWA,2000,5.64

Rwanda,RWA,2001,5.543

Rwanda,RWA,2002,5.445

Rwanda,RWA,2003,5.345

Rwanda,RWA,2004,5.243

Rwanda,RWA,2005,5.135

Rwanda,RWA,2006,5.021

Rwanda,RWA,2007,4.9

Rwanda,RWA,2008,4.774

Rwanda,RWA,2009,4.644

Rwanda,RWA,2010,4.515

Rwanda,RWA,2011,4.389

Rwanda,RWA,2012,4.269

Rwanda,RWA,2013,4.158

Rwanda,RWA,2014,4.058

Rwanda,RWA,2015,3.967

Saint Lucia,LCA,1950,5.338

Saint Lucia,LCA,1951,5.529

Saint Lucia,LCA,1952,5.886

Saint Lucia,LCA,1953,6.191

Saint Lucia,LCA,1954,6.445

Saint Lucia,LCA,1955,6.646

Saint Lucia,LCA,1956,6.798

Saint Lucia,LCA,1957,6.901

Saint Lucia,LCA,1958,6.959

Saint Lucia,LCA,1959,6.979

Saint Lucia,LCA,1960,6.967

Saint Lucia,LCA,1961,6.931

Saint Lucia,LCA,1962,6.88

Saint Lucia,LCA,1963,6.821

Saint Lucia,LCA,1964,6.758

Saint Lucia,LCA,1965,6.688

Saint Lucia,LCA,1966,6.607

Saint Lucia,LCA,1967,6.508

Saint Lucia,LCA,1968,6.387

Saint Lucia,LCA,1969,6.249

Saint Lucia,LCA,1970,6.101

Saint Lucia,LCA,1971,5.953

Saint Lucia,LCA,1972,5.813

Saint Lucia,LCA,1973,5.686

Saint Lucia,LCA,1974,5.571

Saint Lucia,LCA,1975,5.462

Saint Lucia,LCA,1976,5.347

Saint Lucia,LCA,1977,5.216

Saint Lucia,LCA,1978,5.064

Saint Lucia,LCA,1979,4.891

Saint Lucia,LCA,1980,4.703

Saint Lucia,LCA,1981,4.512

Saint Lucia,LCA,1982,4.328

Saint Lucia,LCA,1983,4.161

Saint Lucia,LCA,1984,4.014

Saint Lucia,LCA,1985,3.888

Saint Lucia,LCA,1986,3.781

Saint Lucia,LCA,1987,3.683

Saint Lucia,LCA,1988,3.589

Saint Lucia,LCA,1989,3.495

Saint Lucia,LCA,1990,3.399

Saint Lucia,LCA,1991,3.302

Saint Lucia,LCA,1992,3.203

Saint Lucia,LCA,1993,3.104

Saint Lucia,LCA,1994,3.001

Saint Lucia,LCA,1995,2.892

Saint Lucia,LCA,1996,2.772

Saint Lucia,LCA,1997,2.641

Saint Lucia,LCA,1998,2.499

Saint Lucia,LCA,1999,2.35

Saint Lucia,LCA,2000,2.202

Saint Lucia,LCA,2001,2.06

Saint Lucia,LCA,2002,1.933

Saint Lucia,LCA,2003,1.826

Saint Lucia,LCA,2004,1.74

Saint Lucia,LCA,2005,1.675

Saint Lucia,LCA,2006,1.63

Saint Lucia,LCA,2007,1.598

Saint Lucia,LCA,2008,1.574

Saint Lucia,LCA,2009,1.555

Saint Lucia,LCA,2010,1.538

Saint Lucia,LCA,2011,1.522

Saint Lucia,LCA,2012,1.507

Saint Lucia,LCA,2013,1.495

Saint Lucia,LCA,2014,1.482

Saint Lucia,LCA,2015,1.471

Saint Vincent and the Grenadines,VCT,1950,7.256

Saint Vincent and the Grenadines,VCT,1951,7.281

Saint Vincent and the Grenadines,VCT,1952,7.324

Saint Vincent and the Grenadines,VCT,1953,7.355

Saint Vincent and the Grenadines,VCT,1954,7.373

Saint Vincent and the Grenadines,VCT,1955,7.379

Saint Vincent and the Grenadines,VCT,1956,7.373

Saint Vincent and the Grenadines,VCT,1957,7.354

Saint Vincent and the Grenadines,VCT,1958,7.323

Saint Vincent and the Grenadines,VCT,1959,7.28

Saint Vincent and the Grenadines,VCT,1960,7.224

Saint Vincent and the Grenadines,VCT,1961,7.155

Saint Vincent and the Grenadines,VCT,1962,7.074

Saint Vincent and the Grenadines,VCT,1963,6.981

Saint Vincent and the Grenadines,VCT,1964,6.876

Saint Vincent and the Grenadines,VCT,1965,6.76

Saint Vincent and the Grenadines,VCT,1966,6.632

Saint Vincent and the Grenadines,VCT,1967,6.494

Saint Vincent and the Grenadines,VCT,1968,6.345

Saint Vincent and the Grenadines,VCT,1969,6.185

Saint Vincent and the Grenadines,VCT,1970,6.014

Saint Vincent and the Grenadines,VCT,1971,5.828

Saint Vincent and the Grenadines,VCT,1972,5.629

Saint Vincent and the Grenadines,VCT,1973,5.417

Saint Vincent and the Grenadines,VCT,1974,5.197

Saint Vincent and the Grenadines,VCT,1975,4.973

Saint Vincent and the Grenadines,VCT,1976,4.752

Saint Vincent and the Grenadines,VCT,1977,4.54

Saint Vincent and the Grenadines,VCT,1978,4.339

Saint Vincent and the Grenadines,VCT,1979,4.154

Saint Vincent and the Grenadines,VCT,1980,3.985

Saint Vincent and the Grenadines,VCT,1981,3.832

Saint Vincent and the Grenadines,VCT,1982,3.692

Saint Vincent and the Grenadines,VCT,1983,3.561

Saint Vincent and the Grenadines,VCT,1984,3.439

Saint Vincent and the Grenadines,VCT,1985,3.328

Saint Vincent and the Grenadines,VCT,1986,3.23

Saint Vincent and the Grenadines,VCT,1987,3.144

Saint Vincent and the Grenadines,VCT,1988,3.072

Saint Vincent and the Grenadines,VCT,1989,3.01

Saint Vincent and the Grenadines,VCT,1990,2.956

Saint Vincent and the Grenadines,VCT,1991,2.907

Saint Vincent and the Grenadines,VCT,1992,2.86

Saint Vincent and the Grenadines,VCT,1993,2.811

Saint Vincent and the Grenadines,VCT,1994,2.76

Saint Vincent and the Grenadines,VCT,1995,2.703

Saint Vincent and the Grenadines,VCT,1996,2.641

Saint Vincent and the Grenadines,VCT,1997,2.576

Saint Vincent and the Grenadines,VCT,1998,2.509

Saint Vincent and the Grenadines,VCT,1999,2.442

Saint Vincent and the Grenadines,VCT,2000,2.379

Saint Vincent and the Grenadines,VCT,2001,2.321

Saint Vincent and the Grenadines,VCT,2002,2.271

Saint Vincent and the Grenadines,VCT,2003,2.229

Saint Vincent and the Grenadines,VCT,2004,2.196

Saint Vincent and the Grenadines,VCT,2005,2.17

Saint Vincent and the Grenadines,VCT,2006,2.149

Saint Vincent and the Grenadines,VCT,2007,2.131

Saint Vincent and the Grenadines,VCT,2008,2.112

Saint Vincent and the Grenadines,VCT,2009,2.092

Saint Vincent and the Grenadines,VCT,2010,2.07

Saint Vincent and the Grenadines,VCT,2011,2.046

Saint Vincent and the Grenadines,VCT,2012,2.021

Saint Vincent and the Grenadines,VCT,2013,1.998

Saint Vincent and the Grenadines,VCT,2014,1.975

Saint Vincent and the Grenadines,VCT,2015,1.953

Samoa,WSM,1950,7.639

Samoa,WSM,1951,7.635

Samoa,WSM,1952,7.629

Samoa,WSM,1953,7.626

Samoa,WSM,1954,7.626

Samoa,WSM,1955,7.628

Samoa,WSM,1956,7.633

Samoa,WSM,1957,7.639

Samoa,WSM,1958,7.645

Samoa,WSM,1959,7.65

Samoa,WSM,1960,7.651

Samoa,WSM,1961,7.645

Samoa,WSM,1962,7.63

Samoa,WSM,1963,7.604

Samoa,WSM,1964,7.567

Samoa,WSM,1965,7.519

Samoa,WSM,1966,7.462

Samoa,WSM,1967,7.4

Samoa,WSM,1968,7.334

Samoa,WSM,1969,7.265

Samoa,WSM,1970,7.194

Samoa,WSM,1971,7.119

Samoa,WSM,1972,7.039

Samoa,WSM,1973,6.952

Samoa,WSM,1974,6.859

Samoa,WSM,1975,6.761

Samoa,WSM,1976,6.656

Samoa,WSM,1977,6.547

Samoa,WSM,1978,6.434

Samoa,WSM,1979,6.32

Samoa,WSM,1980,6.203

Samoa,WSM,1981,6.086

Samoa,WSM,1982,5.968

Samoa,WSM,1983,5.85

Samoa,WSM,1984,5.734

Samoa,WSM,1985,5.62

Samoa,WSM,1986,5.51

Samoa,WSM,1987,5.404

Samoa,WSM,1988,5.303

Samoa,WSM,1989,5.208

Samoa,WSM,1990,5.118

Samoa,WSM,1991,5.034

Samoa,WSM,1992,4.956

Samoa,WSM,1993,4.882

Samoa,WSM,1994,4.815

Samoa,WSM,1995,4.751

Samoa,WSM,1996,4.692

Samoa,WSM,1997,4.637

Samoa,WSM,1998,4.587

Samoa,WSM,1999,4.541

Samoa,WSM,2000,4.503

Samoa,WSM,2001,4.476

Samoa,WSM,2002,4.46

Samoa,WSM,2003,4.454

Samoa,WSM,2004,4.456

Samoa,WSM,2005,4.46

Samoa,WSM,2006,4.46

Samoa,WSM,2007,4.45

Samoa,WSM,2008,4.426

Samoa,WSM,2009,4.388

Samoa,WSM,2010,4.338

Samoa,WSM,2011,4.277

Samoa,WSM,2012,4.212

Samoa,WSM,2013,4.148

Samoa,WSM,2014,4.086

Samoa,WSM,2015,4.029

Sao Tome and Principe,STP,1950,6.227

Sao Tome and Principe,STP,1951,6.218

Sao Tome and Principe,STP,1952,6.202

Sao Tome and Principe,STP,1953,6.191

Sao Tome and Principe,STP,1954,6.185

Sao Tome and Principe,STP,1955,6.184

Sao Tome and Principe,STP,1956,6.187

Sao Tome and Principe,STP,1957,6.195

Sao Tome and Principe,STP,1958,6.208

Sao Tome and Principe,STP,1959,6.223

Sao Tome and Principe,STP,1960,6.242

Sao Tome and Principe,STP,1961,6.263

Sao Tome and Principe,STP,1962,6.284

Sao Tome and Principe,STP,1963,6.306

Sao Tome and Principe,STP,1964,6.327

Sao Tome and Principe,STP,1965,6.349

Sao Tome and Principe,STP,1966,6.371

Sao Tome and Principe,STP,1967,6.394

Sao Tome and Principe,STP,1968,6.419

Sao Tome and Principe,STP,1969,6.444

Sao Tome and Principe,STP,1970,6.468

Sao Tome and Principe,STP,1971,6.49

Sao Tome and Principe,STP,1972,6.51

Sao Tome and Principe,STP,1973,6.525

Sao Tome and Principe,STP,1974,6.534

Sao Tome and Principe,STP,1975,6.534

Sao Tome and Principe,STP,1976,6.524

Sao Tome and Principe,STP,1977,6.504

Sao Tome and Principe,STP,1978,6.473

Sao Tome and Principe,STP,1979,6.434

Sao Tome and Principe,STP,1980,6.386

Sao Tome and Principe,STP,1981,6.333

Sao Tome and Principe,STP,1982,6.276

Sao Tome and Principe,STP,1983,6.217

Sao Tome and Principe,STP,1984,6.158

Sao Tome and Principe,STP,1985,6.101

Sao Tome and Principe,STP,1986,6.044

Sao Tome and Principe,STP,1987,5.988

Sao Tome and Principe,STP,1988,5.932

Sao Tome and Principe,STP,1989,5.876

Sao Tome and Principe,STP,1990,5.82

Sao Tome and Principe,STP,1991,5.765

Sao Tome and Principe,STP,1992,5.71

Sao Tome and Principe,STP,1993,5.655

Sao Tome and Principe,STP,1994,5.6

Sao Tome and Principe,STP,1995,5.545

Sao Tome and Principe,STP,1996,5.491

Sao Tome and Principe,STP,1997,5.437

Sao Tome and Principe,STP,1998,5.384

Sao Tome and Principe,STP,1999,5.33

Sao Tome and Principe,STP,2000,5.278

Sao Tome and Principe,STP,2001,5.226

Sao Tome and Principe,STP,2002,5.174

Sao Tome and Principe,STP,2003,5.123

Sao Tome and Principe,STP,2004,5.072

Sao Tome and Principe,STP,2005,5.022

Sao Tome and Principe,STP,2006,4.974

Sao Tome and Principe,STP,2007,4.927

Sao Tome and Principe,STP,2008,4.88

Sao Tome and Principe,STP,2009,4.835

Sao Tome and Principe,STP,2010,4.788

Sao Tome and Principe,STP,2011,4.74

Sao Tome and Principe,STP,2012,4.689

Sao Tome and Principe,STP,2013,4.635

Sao Tome and Principe,STP,2014,4.578

Sao Tome and Principe,STP,2015,4.518

Saudi Arabia,SAU,1950,7.2

Saudi Arabia,SAU,1951,7.191

Saudi Arabia,SAU,1952,7.176

Saudi Arabia,SAU,1953,7.166

Saudi Arabia,SAU,1954,7.161

Saudi Arabia,SAU,1955,7.161

Saudi Arabia,SAU,1956,7.165

Saudi Arabia,SAU,1957,7.174

Saudi Arabia,SAU,1958,7.186

Saudi Arabia,SAU,1959,7.2

Saudi Arabia,SAU,1960,7.216

Saudi Arabia,SAU,1961,7.23

Saudi Arabia,SAU,1962,7.243

Saudi Arabia,SAU,1963,7.252

Saudi Arabia,SAU,1964,7.257

Saudi Arabia,SAU,1965,7.26

Saudi Arabia,SAU,1966,7.262

Saudi Arabia,SAU,1967,7.264

Saudi Arabia,SAU,1968,7.267

Saudi Arabia,SAU,1969,7.273

Saudi Arabia,SAU,1970,7.28

Saudi Arabia,SAU,1971,7.289

Saudi Arabia,SAU,1972,7.299

Saudi Arabia,SAU,1973,7.307

Saudi Arabia,SAU,1974,7.312

Saudi Arabia,SAU,1975,7.313

Saudi Arabia,SAU,1976,7.309

Saudi Arabia,SAU,1977,7.299

Saudi Arabia,SAU,1978,7.281

Saudi Arabia,SAU,1979,7.252

Saudi Arabia,SAU,1980,7.206

Saudi Arabia,SAU,1981,7.138

Saudi Arabia,SAU,1982,7.047

Saudi Arabia,SAU,1983,6.933

Saudi Arabia,SAU,1984,6.798

Saudi Arabia,SAU,1985,6.649

Saudi Arabia,SAU,1986,6.495

Saudi Arabia,SAU,1987,6.342

Saudi Arabia,SAU,1988,6.194

Saudi Arabia,SAU,1989,6.052

Saudi Arabia,SAU,1990,5.911

Saudi Arabia,SAU,1991,5.761

Saudi Arabia,SAU,1992,5.594

Saudi Arabia,SAU,1993,5.407

Saudi Arabia,SAU,1994,5.2

Saudi Arabia,SAU,1995,4.98

Saudi Arabia,SAU,1996,4.754

Saudi Arabia,SAU,1997,4.533

Saudi Arabia,SAU,1998,4.325

Saudi Arabia,SAU,1999,4.137

Saudi Arabia,SAU,2000,3.971

Saudi Arabia,SAU,2001,3.83

Saudi Arabia,SAU,2002,3.708

Saudi Arabia,SAU,2003,3.601

Saudi Arabia,SAU,2004,3.505

Saudi Arabia,SAU,2005,3.416

Saudi Arabia,SAU,2006,3.329

Saudi Arabia,SAU,2007,3.242

Saudi Arabia,SAU,2008,3.151

Saudi Arabia,SAU,2009,3.056

Saudi Arabia,SAU,2010,2.96

Saudi Arabia,SAU,2011,2.866

Saudi Arabia,SAU,2012,2.779

Saudi Arabia,SAU,2013,2.701

Saudi Arabia,SAU,2014,2.635

Saudi Arabia,SAU,2015,2.579

Senegal,SEN,1950,6.787

Senegal,SEN,1951,6.788

Senegal,SEN,1952,6.792

Senegal,SEN,1953,6.801

Senegal,SEN,1954,6.815

Senegal,SEN,1955,6.834

Senegal,SEN,1956,6.858

Senegal,SEN,1957,6.887

Senegal,SEN,1958,6.92

Senegal,SEN,1959,6.957

Senegal,SEN,1960,6.996

Senegal,SEN,1961,7.037

Senegal,SEN,1962,7.078

Senegal,SEN,1963,7.117

Senegal,SEN,1964,7.154

Senegal,SEN,1965,7.186

Senegal,SEN,1966,7.215

Senegal,SEN,1967,7.24

Senegal,SEN,1968,7.26

Senegal,SEN,1969,7.276

Senegal,SEN,1970,7.289

Senegal,SEN,1971,7.296

Senegal,SEN,1972,7.299

Senegal,SEN,1973,7.299

Senegal,SEN,1974,7.296

Senegal,SEN,1975,7.293

Senegal,SEN,1976,7.294

Senegal,SEN,1977,7.298

Senegal,SEN,1978,7.304

Senegal,SEN,1979,7.311

Senegal,SEN,1980,7.312

Senegal,SEN,1981,7.3

Senegal,SEN,1982,7.271

Senegal,SEN,1983,7.221

Senegal,SEN,1984,7.152

Senegal,SEN,1985,7.063

Senegal,SEN,1986,6.962

Senegal,SEN,1987,6.853

Senegal,SEN,1988,6.744

Senegal,SEN,1989,6.636

Senegal,SEN,1990,6.53

Senegal,SEN,1991,6.426

Senegal,SEN,1992,6.322

Senegal,SEN,1993,6.214

Senegal,SEN,1994,6.105

Senegal,SEN,1995,5.995

Senegal,SEN,1996,5.884

Senegal,SEN,1997,5.774

Senegal,SEN,1998,5.667

Senegal,SEN,1999,5.565

Senegal,SEN,2000,5.471

Senegal,SEN,2001,5.387

Senegal,SEN,2002,5.314

Senegal,SEN,2003,5.252

Senegal,SEN,2004,5.201

Senegal,SEN,2005,5.161

Senegal,SEN,2006,5.132

Senegal,SEN,2007,5.111

Senegal,SEN,2008,5.095

Senegal,SEN,2009,5.08

Senegal,SEN,2010,5.063

Senegal,SEN,2011,5.038

Senegal,SEN,2012,5.003

Senegal,SEN,2013,4.959

Senegal,SEN,2014,4.904

Senegal,SEN,2015,4.84

Serbia (including Kosovo),,1950,3.638

Serbia (including Kosovo),,1951,3.519

Serbia (including Kosovo),,1952,3.296

Serbia (including Kosovo),,1953,3.101

Serbia (including Kosovo),,1954,2.936

Serbia (including Kosovo),,1955,2.799

Serbia (including Kosovo),,1956,2.69

Serbia (including Kosovo),,1957,2.608

Serbia (including Kosovo),,1958,2.55

Serbia (including Kosovo),,1959,2.514

Serbia (including Kosovo),,1960,2.494

Serbia (including Kosovo),,1961,2.486

Serbia (including Kosovo),,1962,2.484

Serbia (including Kosovo),,1963,2.482

Serbia (including Kosovo),,1964,2.478

Serbia (including Kosovo),,1965,2.469

Serbia (including Kosovo),,1966,2.454

Serbia (including Kosovo),,1967,2.437

Serbia (including Kosovo),,1968,2.419

Serbia (including Kosovo),,1969,2.403

Serbia (including Kosovo),,1970,2.388

Serbia (including Kosovo),,1971,2.377

Serbia (including Kosovo),,1972,2.369

Serbia (including Kosovo),,1973,2.364

Serbia (including Kosovo),,1974,2.363

Serbia (including Kosovo),,1975,2.364

Serbia (including Kosovo),,1976,2.365

Serbia (including Kosovo),,1977,2.366

Serbia (including Kosovo),,1978,2.364

Serbia (including Kosovo),,1979,2.36

Serbia (including Kosovo),,1980,2.353

Serbia (including Kosovo),,1981,2.344

Serbia (including Kosovo),,1982,2.334

Serbia (including Kosovo),,1983,2.322

Serbia (including Kosovo),,1984,2.309

Serbia (including Kosovo),,1985,2.292

Serbia (including Kosovo),,1986,2.268

Serbia (including Kosovo),,1987,2.236

Serbia (including Kosovo),,1988,2.196

Serbia (including Kosovo),,1989,2.149

Serbia (including Kosovo),,1990,2.098

Serbia (including Kosovo),,1991,2.046

Serbia (including Kosovo),,1992,1.997

Serbia (including Kosovo),,1993,1.953

Serbia (including Kosovo),,1994,1.916

Serbia (including Kosovo),,1995,1.885

Serbia (including Kosovo),,1996,1.859

Serbia (including Kosovo),,1997,1.837

Serbia (including Kosovo),,1998,1.815

Serbia (including Kosovo),,1999,1.792

Serbia (including Kosovo),,2000,1.769

Serbia (including Kosovo),,2001,1.743

Serbia (including Kosovo),,2002,1.716

Serbia (including Kosovo),,2003,1.689

Serbia (including Kosovo),,2004,1.661

Serbia (including Kosovo),,2005,1.636

Serbia (including Kosovo),,2006,1.614

Serbia (including Kosovo),,2007,1.596

Serbia (including Kosovo),,2008,1.584

Serbia (including Kosovo),,2009,1.577

Serbia (including Kosovo),,2010,1.576

Serbia (including Kosovo),,2011,1.578

Serbia (including Kosovo),,2012,1.584

Serbia (including Kosovo),,2013,1.59

Serbia (including Kosovo),,2014,1.597

Serbia (including Kosovo),,2015,1.604

Seychelles,SYC,1950,5.157

Seychelles,SYC,1951,5.103

Seychelles,SYC,1952,5.01

Seychelles,SYC,1953,4.946

Seychelles,SYC,1954,4.912

Seychelles,SYC,1955,4.906

Seychelles,SYC,1956,4.93

Seychelles,SYC,1957,4.981

Seychelles,SYC,1958,5.057

Seychelles,SYC,1959,5.155

Seychelles,SYC,1960,5.27

Seychelles,SYC,1961,5.397

Seychelles,SYC,1962,5.526

Seychelles,SYC,1963,5.649

Seychelles,SYC,1964,5.757

Seychelles,SYC,1965,5.842

Seychelles,SYC,1966,5.894

Seychelles,SYC,1967,5.915

Seychelles,SYC,1968,5.9

Seychelles,SYC,1969,5.85

Seychelles,SYC,1970,5.761

Seychelles,SYC,1971,5.632

Seychelles,SYC,1972,5.468

Seychelles,SYC,1973,5.277

Seychelles,SYC,1974,5.066

Seychelles,SYC,1975,4.845

Seychelles,SYC,1976,4.623

Seychelles,SYC,1977,4.407

Seychelles,SYC,1978,4.203

Seychelles,SYC,1979,4.016

Seychelles,SYC,1980,3.848

Seychelles,SYC,1981,3.698

Seychelles,SYC,1982,3.56

Seychelles,SYC,1983,3.43

Seychelles,SYC,1984,3.307

Seychelles,SYC,1985,3.191

Seychelles,SYC,1986,3.084

Seychelles,SYC,1987,2.987

Seychelles,SYC,1988,2.898

Seychelles,SYC,1989,2.817

Seychelles,SYC,1990,2.74

Seychelles,SYC,1991,2.664

Seychelles,SYC,1992,2.585

Seychelles,SYC,1993,2.505

Seychelles,SYC,1994,2.423

Seychelles,SYC,1995,2.345

Seychelles,SYC,1996,2.275

Seychelles,SYC,1997,2.219

Seychelles,SYC,1998,2.179

Seychelles,SYC,1999,2.157

Seychelles,SYC,2000,2.151

Seychelles,SYC,2001,2.159

Seychelles,SYC,2002,2.177

Seychelles,SYC,2003,2.198

Seychelles,SYC,2004,2.221

Seychelles,SYC,2005,2.245

Seychelles,SYC,2006,2.268

Seychelles,SYC,2007,2.292

Seychelles,SYC,2008,2.316

Seychelles,SYC,2009,2.339

Seychelles,SYC,2010,2.357

Seychelles,SYC,2011,2.368

Seychelles,SYC,2012,2.371

Seychelles,SYC,2013,2.366

Seychelles,SYC,2014,2.353

Seychelles,SYC,2015,2.333

Sierra Leone,SLE,1950,6.09

Sierra Leone,SLE,1951,6.069

Sierra Leone,SLE,1952,6.034

Sierra Leone,SLE,1953,6.009

Sierra Leone,SLE,1954,5.996

Sierra Leone,SLE,1955,5.993

Sierra Leone,SLE,1956,6.002

Sierra Leone,SLE,1957,6.021

Sierra Leone,SLE,1958,6.049

Sierra Leone,SLE,1959,6.085

Sierra Leone,SLE,1960,6.127

Sierra Leone,SLE,1961,6.171

Sierra Leone,SLE,1962,6.216

Sierra Leone,SLE,1963,6.26

Sierra Leone,SLE,1964,6.3

Sierra Leone,SLE,1965,6.337

Sierra Leone,SLE,1966,6.37

Sierra Leone,SLE,1967,6.402

Sierra Leone,SLE,1968,6.434

Sierra Leone,SLE,1969,6.465

Sierra Leone,SLE,1970,6.496

Sierra Leone,SLE,1971,6.524

Sierra Leone,SLE,1972,6.55

Sierra Leone,SLE,1973,6.573

Sierra Leone,SLE,1974,6.594

Sierra Leone,SLE,1975,6.611

Sierra Leone,SLE,1976,6.628

Sierra Leone,SLE,1977,6.644

Sierra Leone,SLE,1978,6.66

Sierra Leone,SLE,1979,6.675

Sierra Leone,SLE,1980,6.69

Sierra Leone,SLE,1981,6.704

Sierra Leone,SLE,1982,6.715

Sierra Leone,SLE,1983,6.724

Sierra Leone,SLE,1984,6.729

Sierra Leone,SLE,1985,6.731

Sierra Leone,SLE,1986,6.732

Sierra Leone,SLE,1987,6.731

Sierra Leone,SLE,1988,6.73

Sierra Leone,SLE,1989,6.726

Sierra Leone,SLE,1990,6.721

Sierra Leone,SLE,1991,6.711

Sierra Leone,SLE,1992,6.696

Sierra Leone,SLE,1993,6.674

Sierra Leone,SLE,1994,6.645

Sierra Leone,SLE,1995,6.608

Sierra Leone,SLE,1996,6.564

Sierra Leone,SLE,1997,6.512

Sierra Leone,SLE,1998,6.455

Sierra Leone,SLE,1999,6.39

Sierra Leone,SLE,2000,6.319

Sierra Leone,SLE,2001,6.242

Sierra Leone,SLE,2002,6.158

Sierra Leone,SLE,2003,6.067

Sierra Leone,SLE,2004,5.969

Sierra Leone,SLE,2005,5.863

Sierra Leone,SLE,2006,5.747

Sierra Leone,SLE,2007,5.621

Sierra Leone,SLE,2008,5.487

Sierra Leone,SLE,2009,5.346

Sierra Leone,SLE,2010,5.202

Sierra Leone,SLE,2011,5.06

Sierra Leone,SLE,2012,4.923

Sierra Leone,SLE,2013,4.793

Sierra Leone,SLE,2014,4.672

Sierra Leone,SLE,2015,4.561

Singapore,SGP,1950,6.487

Singapore,SGP,1951,6.538

Singapore,SGP,1952,6.62

Singapore,SGP,1953,6.661

Singapore,SGP,1954,6.661

Singapore,SGP,1955,6.62

Singapore,SGP,1956,6.538

Singapore,SGP,1957,6.415

Singapore,SGP,1958,6.254

Singapore,SGP,1959,6.055

Singapore,SGP,1960,5.821

Singapore,SGP,1961,5.557

Singapore,SGP,1962,5.27

Singapore,SGP,1963,4.967

Singapore,SGP,1964,4.658

Singapore,SGP,1965,4.356

Singapore,SGP,1966,4.074

Singapore,SGP,1967,3.815

Singapore,SGP,1968,3.582

Singapore,SGP,1969,3.375

Singapore,SGP,1970,3.19

Singapore,SGP,1971,3.014

Singapore,SGP,1972,2.836

Singapore,SGP,1973,2.652

Singapore,SGP,1974,2.461

Singapore,SGP,1975,2.272

Singapore,SGP,1976,2.096

Singapore,SGP,1977,1.945

Singapore,SGP,1978,1.827

Singapore,SGP,1979,1.743

Singapore,SGP,1980,1.692

Singapore,SGP,1981,1.669

Singapore,SGP,1982,1.664

Singapore,SGP,1983,1.667

Singapore,SGP,1984,1.673

Singapore,SGP,1985,1.68

Singapore,SGP,1986,1.687

Singapore,SGP,1987,1.695

Singapore,SGP,1988,1.706

Singapore,SGP,1989,1.718

Singapore,SGP,1990,1.726

Singapore,SGP,1991,1.73

Singapore,SGP,1992,1.727

Singapore,SGP,1993,1.716

Singapore,SGP,1994,1.697

Singapore,SGP,1995,1.67

Singapore,SGP,1996,1.634

Singapore,SGP,1997,1.592

Singapore,SGP,1998,1.547

Singapore,SGP,1999,1.5

Singapore,SGP,2000,1.454

Singapore,SGP,2001,1.411

Singapore,SGP,2002,1.372

Singapore,SGP,2003,1.338

Singapore,SGP,2004,1.31

Singapore,SGP,2005,1.287

Singapore,SGP,2006,1.271

Singapore,SGP,2007,1.258

Singapore,SGP,2008,1.248

Singapore,SGP,2009,1.241

Singapore,SGP,2010,1.236

Singapore,SGP,2011,1.233

Singapore,SGP,2012,1.232

Singapore,SGP,2013,1.234

Singapore,SGP,2014,1.238

Singapore,SGP,2015,1.242

Slovakia,SVK,1950,3.592

Slovakia,SVK,1951,3.571

Slovakia,SVK,1952,3.527

Slovakia,SVK,1953,3.481

Slovakia,SVK,1954,3.433

Slovakia,SVK,1955,3.382

Slovakia,SVK,1956,3.329

Slovakia,SVK,1957,3.273

Slovakia,SVK,1958,3.214

Slovakia,SVK,1959,3.152

Slovakia,SVK,1960,3.085

Slovakia,SVK,1961,3.013

Slovakia,SVK,1962,2.937

Slovakia,SVK,1963,2.858

Slovakia,SVK,1964,2.779

Slovakia,SVK,1965,2.703

Slovakia,SVK,1966,2.637

Slovakia,SVK,1967,2.582

Slovakia,SVK,1968,2.541

Slovakia,SVK,1969,2.514

Slovakia,SVK,1970,2.499

Slovakia,SVK,1971,2.495

Slovakia,SVK,1972,2.497

Slovakia,SVK,1973,2.5

Slovakia,SVK,1974,2.501

Slovakia,SVK,1975,2.496

Slovakia,SVK,1976,2.484

Slovakia,SVK,1977,2.465

Slovakia,SVK,1978,2.44

Slovakia,SVK,1979,2.409

Slovakia,SVK,1980,2.374

Slovakia,SVK,1981,2.338

Slovakia,SVK,1982,2.304

Slovakia,SVK,1983,2.274

Slovakia,SVK,1984,2.246

Slovakia,SVK,1985,2.221

Slovakia,SVK,1986,2.195

Slovakia,SVK,1987,2.166

Slovakia,SVK,1988,2.131

Slovakia,SVK,1989,2.089

Slovakia,SVK,1990,2.036

Slovakia,SVK,1991,1.971

Slovakia,SVK,1992,1.894

Slovakia,SVK,1993,1.809

Slovakia,SVK,1994,1.718

Slovakia,SVK,1995,1.625

Slovakia,SVK,1996,1.534

Slovakia,SVK,1997,1.45

Slovakia,SVK,1998,1.375

Slovakia,SVK,1999,1.314

Slovakia,SVK,2000,1.268

Slovakia,SVK,2001,1.239

Slovakia,SVK,2002,1.226

Slovakia,SVK,2003,1.224

Slovakia,SVK,2004,1.232

Slovakia,SVK,2005,1.248

Slovakia,SVK,2006,1.269

Slovakia,SVK,2007,1.292

Slovakia,SVK,2008,1.315

Slovakia,SVK,2009,1.336

Slovakia,SVK,2010,1.355

Slovakia,SVK,2011,1.37

Slovakia,SVK,2012,1.384

Slovakia,SVK,2013,1.399

Slovakia,SVK,2014,1.413

Slovakia,SVK,2015,1.427

Slovenia,SVN,1950,2.866

Slovenia,SVN,1951,2.813

Slovenia,SVN,1952,2.714

Slovenia,SVN,1953,2.627

Slovenia,SVN,1954,2.553

Slovenia,SVN,1955,2.491

Slovenia,SVN,1956,2.441

Slovenia,SVN,1957,2.402

Slovenia,SVN,1958,2.374

Slovenia,SVN,1959,2.354

Slovenia,SVN,1960,2.341

Slovenia,SVN,1961,2.333

Slovenia,SVN,1962,2.327

Slovenia,SVN,1963,2.32

Slovenia,SVN,1964,2.312

Slovenia,SVN,1965,2.301

Slovenia,SVN,1966,2.287

Slovenia,SVN,1967,2.272

Slovenia,SVN,1968,2.258

Slovenia,SVN,1969,2.243

Slovenia,SVN,1970,2.231

Slovenia,SVN,1971,2.22

Slovenia,SVN,1972,2.213

Slovenia,SVN,1973,2.207

Slovenia,SVN,1974,2.202

Slovenia,SVN,1975,2.195

Slovenia,SVN,1976,2.183

Slovenia,SVN,1977,2.166

Slovenia,SVN,1978,2.14

Slovenia,SVN,1979,2.106

Slovenia,SVN,1980,2.064

Slovenia,SVN,1981,2.016

Slovenia,SVN,1982,1.963

Slovenia,SVN,1983,1.909

Slovenia,SVN,1984,1.853

Slovenia,SVN,1985,1.796

Slovenia,SVN,1986,1.737

Slovenia,SVN,1987,1.674

Slovenia,SVN,1988,1.608

Slovenia,SVN,1989,1.542

Slovenia,SVN,1990,1.478

Slovenia,SVN,1991,1.419

Slovenia,SVN,1992,1.367

Slovenia,SVN,1993,1.325

Slovenia,SVN,1994,1.293

Slovenia,SVN,1995,1.269

Slovenia,SVN,1996,1.251

Slovenia,SVN,1997,1.236

Slovenia,SVN,1998,1.223

Slovenia,SVN,1999,1.213

Slovenia,SVN,2000,1.206

Slovenia,SVN,2001,1.206

Slovenia,SVN,2002,1.217

Slovenia,SVN,2003,1.238

Slovenia,SVN,2004,1.27

Slovenia,SVN,2005,1.31

Slovenia,SVN,2006,1.355

Slovenia,SVN,2007,1.401

Slovenia,SVN,2008,1.444

Slovenia,SVN,2009,1.483

Slovenia,SVN,2010,1.517

Slovenia,SVN,2011,1.543

Slovenia,SVN,2012,1.566

Slovenia,SVN,2013,1.584

Slovenia,SVN,2014,1.6

Slovenia,SVN,2015,1.614

Solomon Islands,SLB,1950,6.401

Solomon Islands,SLB,1951,6.401

Solomon Islands,SLB,1952,6.4

Solomon Islands,SLB,1953,6.4

Solomon Islands,SLB,1954,6.399

Solomon Islands,SLB,1955,6.398

Solomon Islands,SLB,1956,6.397

Solomon Islands,SLB,1957,6.395

Solomon Islands,SLB,1958,6.393

Solomon Islands,SLB,1959,6.391

Solomon Islands,SLB,1960,6.388

Solomon Islands,SLB,1961,6.385

Solomon Islands,SLB,1962,6.383

Solomon Islands,SLB,1963,6.385

Solomon Islands,SLB,1964,6.394

Solomon Islands,SLB,1965,6.419

Solomon Islands,SLB,1966,6.47

Solomon Islands,SLB,1967,6.549

Solomon Islands,SLB,1968,6.655

Solomon Islands,SLB,1969,6.781

Solomon Islands,SLB,1970,6.914

Solomon Islands,SLB,1971,7.04

Solomon Islands,SLB,1972,7.145

Solomon Islands,SLB,1973,7.216

Solomon Islands,SLB,1974,7.248

Solomon Islands,SLB,1975,7.236

Solomon Islands,SLB,1976,7.182

Solomon Islands,SLB,1977,7.096

Solomon Islands,SLB,1978,6.99

Solomon Islands,SLB,1979,6.871

Solomon Islands,SLB,1980,6.748

Solomon Islands,SLB,1981,6.632

Solomon Islands,SLB,1982,6.525

Solomon Islands,SLB,1983,6.431

Solomon Islands,SLB,1984,6.349

Solomon Islands,SLB,1985,6.278

Solomon Islands,SLB,1986,6.211

Solomon Islands,SLB,1987,6.139

Solomon Islands,SLB,1988,6.056

Solomon Islands,SLB,1989,5.961

Solomon Islands,SLB,1990,5.851

Solomon Islands,SLB,1991,5.728

Solomon Islands,SLB,1992,5.596

Solomon Islands,SLB,1993,5.461

Solomon Islands,SLB,1994,5.326

Solomon Islands,SLB,1995,5.197

Solomon Islands,SLB,1996,5.077

Solomon Islands,SLB,1997,4.968

Solomon Islands,SLB,1998,4.872

Solomon Islands,SLB,1999,4.789

Solomon Islands,SLB,2000,4.72

Solomon Islands,SLB,2001,4.665

Solomon Islands,SLB,2002,4.618

Solomon Islands,SLB,2003,4.578

Solomon Islands,SLB,2004,4.54

Solomon Islands,SLB,2005,4.502

Solomon Islands,SLB,2006,4.459

Solomon Islands,SLB,2007,4.412

Solomon Islands,SLB,2008,4.359

Solomon Islands,SLB,2009,4.3

Solomon Islands,SLB,2010,4.235

Solomon Islands,SLB,2011,4.167

Solomon Islands,SLB,2012,4.099

Solomon Islands,SLB,2013,4.032

Solomon Islands,SLB,2014,3.968

Solomon Islands,SLB,2015,3.907

Somalia,SOM,1950,7.248

Somalia,SOM,1951,7.249

Somalia,SOM,1952,7.25

Somalia,SOM,1953,7.251

Somalia,SOM,1954,7.251

Somalia,SOM,1955,7.251

Somalia,SOM,1956,7.25

Somalia,SOM,1957,7.25

Somalia,SOM,1958,7.25

Somalia,SOM,1959,7.25

Somalia,SOM,1960,7.25

Somalia,SOM,1961,7.252

Somalia,SOM,1962,7.255

Somalia,SOM,1963,7.258

Somalia,SOM,1964,7.261

Somalia,SOM,1965,7.261

Somalia,SOM,1966,7.257

Somalia,SOM,1967,7.248

Somalia,SOM,1968,7.232

Somalia,SOM,1969,7.209

Somalia,SOM,1970,7.182

Somalia,SOM,1971,7.151

Somalia,SOM,1972,7.118

Somalia,SOM,1973,7.086

Somalia,SOM,1974,7.057

Somalia,SOM,1975,7.033

Somalia,SOM,1976,7.016

Somalia,SOM,1977,7.005

Somalia,SOM,1978,7

Somalia,SOM,1979,7.003

Somalia,SOM,1980,7.013

Somalia,SOM,1981,7.03

Somalia,SOM,1982,7.053

Somalia,SOM,1983,7.08

Somalia,SOM,1984,7.112

Somalia,SOM,1985,7.149

Somalia,SOM,1986,7.19

Somalia,SOM,1987,7.237

Somalia,SOM,1988,7.288

Somalia,SOM,1989,7.341

Somalia,SOM,1990,7.397

Somalia,SOM,1991,7.454

Somalia,SOM,1992,7.511

Somalia,SOM,1993,7.566

Somalia,SOM,1994,7.614

Somalia,SOM,1995,7.653

Somalia,SOM,1996,7.679

Somalia,SOM,1997,7.689

Somalia,SOM,1998,7.683

Somalia,SOM,1999,7.66

Somalia,SOM,2000,7.623

Somalia,SOM,2001,7.573

Somalia,SOM,2002,7.513

Somalia,SOM,2003,7.448

Somalia,SOM,2004,7.378

Somalia,SOM,2005,7.305

Somalia,SOM,2006,7.227

Somalia,SOM,2007,7.144

Somalia,SOM,2008,7.056

Somalia,SOM,2009,6.963

Somalia,SOM,2010,6.866

Somalia,SOM,2011,6.766

Somalia,SOM,2012,6.665

Somalia,SOM,2013,6.564

Somalia,SOM,2014,6.464

Somalia,SOM,2015,6.365

South Africa,ZAF,1950,6.041

South Africa,ZAF,1951,6.044

South Africa,ZAF,1952,6.049

South Africa,ZAF,1953,6.053

South Africa,ZAF,1954,6.056

South Africa,ZAF,1955,6.057

South Africa,ZAF,1956,6.058

South Africa,ZAF,1957,6.057

South Africa,ZAF,1958,6.055

South Africa,ZAF,1959,6.049

South Africa,ZAF,1960,6.041

South Africa,ZAF,1961,6.028

South Africa,ZAF,1962,6.01

South Africa,ZAF,1963,5.986

South Africa,ZAF,1964,5.956

South Africa,ZAF,1965,5.92

South Africa,ZAF,1966,5.878

South Africa,ZAF,1967,5.832

South Africa,ZAF,1968,5.782

South Africa,ZAF,1969,5.728

South Africa,ZAF,1970,5.67

South Africa,ZAF,1971,5.605

South Africa,ZAF,1972,5.535

South Africa,ZAF,1973,5.457

South Africa,ZAF,1974,5.374

South Africa,ZAF,1975,5.287

South Africa,ZAF,1976,5.198

South Africa,ZAF,1977,5.108

South Africa,ZAF,1978,5.018

South Africa,ZAF,1979,4.928

South Africa,ZAF,1980,4.837

South Africa,ZAF,1981,4.744

South Africa,ZAF,1982,4.646

South Africa,ZAF,1983,4.542

South Africa,ZAF,1984,4.432

South Africa,ZAF,1985,4.315

South Africa,ZAF,1986,4.19

South Africa,ZAF,1987,4.059

South Africa,ZAF,1988,3.924

South Africa,ZAF,1989,3.788

South Africa,ZAF,1990,3.654

South Africa,ZAF,1991,3.525

South Africa,ZAF,1992,3.405

South Africa,ZAF,1993,3.294

South Africa,ZAF,1994,3.196

South Africa,ZAF,1995,3.11

South Africa,ZAF,1996,3.036

South Africa,ZAF,1997,2.973

South Africa,ZAF,1998,2.918

South Africa,ZAF,1999,2.871

South Africa,ZAF,2000,2.829

South Africa,ZAF,2001,2.792

South Africa,ZAF,2002,2.759

South Africa,ZAF,2003,2.729

South Africa,ZAF,2004,2.702

South Africa,ZAF,2005,2.677

South Africa,ZAF,2006,2.655

South Africa,ZAF,2007,2.636

South Africa,ZAF,2008,2.619

South Africa,ZAF,2009,2.603

South Africa,ZAF,2010,2.588

South Africa,ZAF,2011,2.572

South Africa,ZAF,2012,2.554

South Africa,ZAF,2013,2.533

South Africa,ZAF,2014,2.51

South Africa,ZAF,2015,2.485

South America,,1950,5.687

South America,,1951,5.682

South America,,1952,5.672

South America,,1953,5.667

South America,,1954,5.666

South America,,1955,5.669

South America,,1956,5.675

South America,,1957,5.684

South America,,1958,5.694

South America,,1959,5.701

South America,,1960,5.702

South America,,1961,5.692

South America,,1962,5.668

South America,,1963,5.626

South America,,1964,5.566

South America,,1965,5.487

South America,,1966,5.389

South America,,1967,5.276

South America,,1968,5.154

South America,,1969,5.027

South America,,1970,4.901

South America,,1971,4.782

South America,,1972,4.671

South America,,1973,4.571

South America,,1974,4.483

South America,,1975,4.404

South America,,1976,4.332

South America,,1977,4.261

South America,,1978,4.188

South America,,1979,4.111

South America,,1980,4.027

South America,,1981,3.936

South America,,1982,3.841

South America,,1983,3.743

South America,,1984,3.643

South America,,1985,3.544

South America,,1986,3.446

South America,,1987,3.352

South America,,1988,3.261

South America,,1989,3.175

South America,,1990,3.096

South America,,1991,3.022

South America,,1992,2.953

South America,,1993,2.889

South America,,1994,2.828

South America,,1995,2.77

South America,,1996,2.713

South America,,1997,2.657

South America,,1998,2.601

South America,,1999,2.545

South America,,2000,2.489

South America,,2001,2.433

South America,,2002,2.378

South America,,2003,2.326

South America,,2004,2.276

South America,,2005,2.229

South America,,2006,2.188

South America,,2007,2.152

South America,,2008,2.12

South America,,2009,2.094

South America,,2010,2.071

South America,,2011,2.051

South America,,2012,2.034

South America,,2013,2.017

South America,,2014,2

South America,,2015,1.983

South Korea,KOR,1950,5.017

South Korea,KOR,1951,5.209

South Korea,KOR,1952,5.559

South Korea,KOR,1953,5.845

South Korea,KOR,1954,6.064

South Korea,KOR,1955,6.219

South Korea,KOR,1956,6.31

South Korea,KOR,1957,6.338

South Korea,KOR,1958,6.307

South Korea,KOR,1959,6.224

South Korea,KOR,1960,6.095

South Korea,KOR,1961,5.929

South Korea,KOR,1962,5.738

South Korea,KOR,1963,5.533

South Korea,KOR,1964,5.325

South Korea,KOR,1965,5.125

South Korea,KOR,1966,4.941

South Korea,KOR,1967,4.772

South Korea,KOR,1968,4.617

South Korea,KOR,1969,4.474

South Korea,KOR,1970,4.336

South Korea,KOR,1971,4.192

South Korea,KOR,1972,4.036

South Korea,KOR,1973,3.86

South Korea,KOR,1974,3.667

South Korea,KOR,1975,3.462

South Korea,KOR,1976,3.254

South Korea,KOR,1977,3.052

South Korea,KOR,1978,2.866

South Korea,KOR,1979,2.697

South Korea,KOR,1980,2.543

South Korea,KOR,1981,2.399

South Korea,KOR,1982,2.256

South Korea,KOR,1983,2.113

South Korea,KOR,1984,1.971

South Korea,KOR,1985,1.839

South Korea,KOR,1986,1.728

South Korea,KOR,1987,1.646

South Korea,KOR,1988,1.597

South Korea,KOR,1989,1.578

South Korea,KOR,1990,1.584

South Korea,KOR,1991,1.604

South Korea,KOR,1992,1.626

South Korea,KOR,1993,1.638

South Korea,KOR,1994,1.636

South Korea,KOR,1995,1.616

South Korea,KOR,1996,1.576

South Korea,KOR,1997,1.525

South Korea,KOR,1998,1.467

South Korea,KOR,1999,1.407

South Korea,KOR,2000,1.347

South Korea,KOR,2001,1.293

South Korea,KOR,2002,1.246

South Korea,KOR,2003,1.209

South Korea,KOR,2004,1.183

South Korea,KOR,2005,1.167

South Korea,KOR,2006,1.162

South Korea,KOR,2007,1.164

South Korea,KOR,2008,1.17

South Korea,KOR,2009,1.18

South Korea,KOR,2010,1.192

South Korea,KOR,2011,1.206

South Korea,KOR,2012,1.222

South Korea,KOR,2013,1.24

South Korea,KOR,2014,1.258

South Korea,KOR,2015,1.276

South Sudan,SSD,1950,6.628

South Sudan,SSD,1951,6.634

South Sudan,SSD,1952,6.645

South Sudan,SSD,1953,6.656

South Sudan,SSD,1954,6.666

South Sudan,SSD,1955,6.676

South Sudan,SSD,1956,6.685

South Sudan,SSD,1957,6.693

South Sudan,SSD,1958,6.702

South Sudan,SSD,1959,6.711

South Sudan,SSD,1960,6.721

South Sudan,SSD,1961,6.733

South Sudan,SSD,1962,6.747

South Sudan,SSD,1963,6.763

South Sudan,SSD,1964,6.781

South Sudan,SSD,1965,6.8

South Sudan,SSD,1966,6.819

South Sudan,SSD,1967,6.838

South Sudan,SSD,1968,6.855

South Sudan,SSD,1969,6.869

South Sudan,SSD,1970,6.881

South Sudan,SSD,1971,6.892

South Sudan,SSD,1972,6.903

South Sudan,SSD,1973,6.912

South Sudan,SSD,1974,6.92

South Sudan,SSD,1975,6.924

South Sudan,SSD,1976,6.922

South Sudan,SSD,1977,6.913

South Sudan,SSD,1978,6.897

South Sudan,SSD,1979,6.874

South Sudan,SSD,1980,6.85

South Sudan,SSD,1981,6.829

South Sudan,SSD,1982,6.813

South Sudan,SSD,1983,6.806

South Sudan,SSD,1984,6.806

South Sudan,SSD,1985,6.81

South Sudan,SSD,1986,6.816

South Sudan,SSD,1987,6.816

South Sudan,SSD,1988,6.808

South Sudan,SSD,1989,6.789

South Sudan,SSD,1990,6.761

South Sudan,SSD,1991,6.726

South Sudan,SSD,1992,6.686

South Sudan,SSD,1993,6.644

South Sudan,SSD,1994,6.601

South Sudan,SSD,1995,6.554

South Sudan,SSD,1996,6.503

South Sudan,SSD,1997,6.444

South Sudan,SSD,1998,6.377

South Sudan,SSD,1999,6.303

South Sudan,SSD,2000,6.223

South Sudan,SSD,2001,6.139

South Sudan,SSD,2002,6.054

South Sudan,SSD,2003,5.969

South Sudan,SSD,2004,5.885

South Sudan,SSD,2005,5.803

South Sudan,SSD,2006,5.721

South Sudan,SSD,2007,5.638

South Sudan,SSD,2008,5.552

South Sudan,SSD,2009,5.465

South Sudan,SSD,2010,5.376

South Sudan,SSD,2011,5.286

South Sudan,SSD,2012,5.197

South Sudan,SSD,2013,5.109

South Sudan,SSD,2014,5.022

South Sudan,SSD,2015,4.938

South-Central Asia,,1950,5.982

South-Central Asia,,1951,5.985

South-Central Asia,,1952,5.993

South-Central Asia,,1953,6

South-Central Asia,,1954,6.009

South-Central Asia,,1955,6.017

South-Central Asia,,1956,6.027

South-Central Asia,,1957,6.036

South-Central Asia,,1958,6.045

South-Central Asia,,1959,6.052

South-Central Asia,,1960,6.057

South-Central Asia,,1961,6.058

South-Central Asia,,1962,6.053

South-Central Asia,,1963,6.043

South-Central Asia,,1964,6.025

South-Central Asia,,1965,6

South-Central Asia,,1966,5.969

South-Central Asia,,1967,5.931

South-Central Asia,,1968,5.888

South-Central Asia,,1969,5.841

South-Central Asia,,1970,5.789

South-Central Asia,,1971,5.732

South-Central Asia,,1972,5.669

South-Central Asia,,1973,5.602

South-Central Asia,,1974,5.531

South-Central Asia,,1975,5.459

South-Central Asia,,1976,5.389

South-Central Asia,,1977,5.321

South-Central Asia,,1978,5.257

South-Central Asia,,1979,5.197

South-Central Asia,,1980,5.137

South-Central Asia,,1981,5.077

South-Central Asia,,1982,5.014

South-Central Asia,,1983,4.944

South-Central Asia,,1984,4.868

South-Central Asia,,1985,4.784

South-Central Asia,,1986,4.692

South-Central Asia,,1987,4.594

South-Central Asia,,1988,4.492

South-Central Asia,,1989,4.388

South-Central Asia,,1990,4.282

South-Central Asia,,1991,4.178

South-Central Asia,,1992,4.076

South-Central Asia,,1993,3.976

South-Central Asia,,1994,3.881

South-Central Asia,,1995,3.79

South-Central Asia,,1996,3.701

South-Central Asia,,1997,3.615

South-Central Asia,,1998,3.529

South-Central Asia,,1999,3.445

South-Central Asia,,2000,3.363

South-Central Asia,,2001,3.283

South-Central Asia,,2002,3.207

South-Central Asia,,2003,3.134

South-Central Asia,,2004,3.065

South-Central Asia,,2005,2.998

South-Central Asia,,2006,2.933

South-Central Asia,,2007,2.869

South-Central Asia,,2008,2.805

South-Central Asia,,2009,2.742

South-Central Asia,,2010,2.681

South-Central Asia,,2011,2.625

South-Central Asia,,2012,2.573

South-Central Asia,,2013,2.528

South-Central Asia,,2014,2.49

South-Central Asia,,2015,2.457

South-Eastern Asia,,1950,5.795

South-Eastern Asia,,1951,5.833

South-Eastern Asia,,1952,5.904

South-Eastern Asia,,1953,5.965

South-Eastern Asia,,1954,6.017

South-Eastern Asia,,1955,6.059

South-Eastern Asia,,1956,6.091

South-Eastern Asia,,1957,6.114

South-Eastern Asia,,1958,6.129

South-Eastern Asia,,1959,6.135

South-Eastern Asia,,1960,6.134

South-Eastern Asia,,1961,6.126

South-Eastern Asia,,1962,6.113

South-Eastern Asia,,1963,6.093

South-Eastern Asia,,1964,6.067

South-Eastern Asia,,1965,6.035

South-Eastern Asia,,1966,5.996

South-Eastern Asia,,1967,5.947

South-Eastern Asia,,1968,5.889

South-Eastern Asia,,1969,5.819

South-Eastern Asia,,1970,5.738

South-Eastern Asia,,1971,5.644

South-Eastern Asia,,1972,5.537

South-Eastern Asia,,1973,5.419

South-Eastern Asia,,1974,5.292

South-Eastern Asia,,1975,5.159

South-Eastern Asia,,1976,5.023

South-Eastern Asia,,1977,4.888

South-Eastern Asia,,1978,4.754

South-Eastern Asia,,1979,4.625

South-Eastern Asia,,1980,4.5

South-Eastern Asia,,1981,4.376

South-Eastern Asia,,1982,4.253

South-Eastern Asia,,1983,4.128

South-Eastern Asia,,1984,4.003

South-Eastern Asia,,1985,3.878

South-Eastern Asia,,1986,3.757

South-Eastern Asia,,1987,3.64

South-Eastern Asia,,1988,3.529

South-Eastern Asia,,1989,3.424

South-Eastern Asia,,1990,3.325

South-Eastern Asia,,1991,3.23

South-Eastern Asia,,1992,3.137

South-Eastern Asia,,1993,3.046

South-Eastern Asia,,1994,2.958

South-Eastern Asia,,1995,2.874

South-Eastern Asia,,1996,2.796

South-Eastern Asia,,1997,2.728

South-Eastern Asia,,1998,2.671

South-Eastern Asia,,1999,2.624

South-Eastern Asia,,2000,2.586

South-Eastern Asia,,2001,2.557

South-Eastern Asia,,2002,2.532

South-Eastern Asia,,2003,2.51

South-Eastern Asia,,2004,2.49

South-Eastern Asia,,2005,2.47

South-Eastern Asia,,2006,2.45

South-Eastern Asia,,2007,2.432

South-Eastern Asia,,2008,2.415

South-Eastern Asia,,2009,2.399

South-Eastern Asia,,2010,2.383

South-Eastern Asia,,2011,2.368

South-Eastern Asia,,2012,2.351

South-Eastern Asia,,2013,2.334

South-Eastern Asia,,2014,2.316

South-Eastern Asia,,2015,2.297

Southern Africa,,1950,6.052

Southern Africa,,1951,6.056

Southern Africa,,1952,6.062

Southern Africa,,1953,6.067

Southern Africa,,1954,6.072

Southern Africa,,1955,6.075

Southern Africa,,1956,6.076

Southern Africa,,1957,6.077

Southern Africa,,1958,6.076

Southern Africa,,1959,6.072

Southern Africa,,1960,6.065

Southern Africa,,1961,6.055

Southern Africa,,1962,6.04

Southern Africa,,1963,6.019

Southern Africa,,1964,5.994

Southern Africa,,1965,5.963

Southern Africa,,1966,5.928

Southern Africa,,1967,5.888

Southern Africa,,1968,5.845

Southern Africa,,1969,5.799

Southern Africa,,1970,5.748

Southern Africa,,1971,5.692

Southern Africa,,1972,5.629

Southern Africa,,1973,5.56

Southern Africa,,1974,5.486

Southern Africa,,1975,5.406

Southern Africa,,1976,5.324

Southern Africa,,1977,5.24

Southern Africa,,1978,5.155

Southern Africa,,1979,5.07

Southern Africa,,1980,4.982

Southern Africa,,1981,4.891

Southern Africa,,1982,4.794

Southern Africa,,1983,4.691

Southern Africa,,1984,4.582

Southern Africa,,1985,4.465

Southern Africa,,1986,4.34

Southern Africa,,1987,4.209

Southern Africa,,1988,4.075

Southern Africa,,1989,3.94

Southern Africa,,1990,3.808

Southern Africa,,1991,3.68

Southern Africa,,1992,3.56

Southern Africa,,1993,3.449

Southern Africa,,1994,3.349

Southern Africa,,1995,3.26

Southern Africa,,1996,3.183

Southern Africa,,1997,3.114

Southern Africa,,1998,3.053

Southern Africa,,1999,2.999

Southern Africa,,2000,2.95

Southern Africa,,2001,2.906

Southern Africa,,2002,2.866

Southern Africa,,2003,2.831

Southern Africa,,2004,2.799

Southern Africa,,2005,2.771

Southern Africa,,2006,2.746

Southern Africa,,2007,2.725

Southern Africa,,2008,2.707

Southern Africa,,2009,2.692

Southern Africa,,2010,2.677

Southern Africa,,2011,2.661

Southern Africa,,2012,2.643

Southern Africa,,2013,2.622

Southern Africa,,2014,2.598

Southern Africa,,2015,2.57

Southern Asia,,1950,6.033

Southern Asia,,1951,6.035

Southern Asia,,1952,6.039

Southern Asia,,1953,6.043

Southern Asia,,1954,6.048

Southern Asia,,1955,6.053

Southern Asia,,1956,6.059

Southern Asia,,1957,6.065

Southern Asia,,1958,6.071

Southern Asia,,1959,6.075

Southern Asia,,1960,6.078

Southern Asia,,1961,6.077

Southern Asia,,1962,6.073

Southern Asia,,1963,6.063

Southern Asia,,1964,6.047

Southern Asia,,1965,6.024

Southern Asia,,1966,5.995

Southern Asia,,1967,5.96

Southern Asia,,1968,5.918

Southern Asia,,1969,5.872

Southern Asia,,1970,5.82

Southern Asia,,1971,5.762

Southern Asia,,1972,5.699

Southern Asia,,1973,5.632

Southern Asia,,1974,5.562

Southern Asia,,1975,5.491

Southern Asia,,1976,5.422

Southern Asia,,1977,5.356

Southern Asia,,1978,5.294

Southern Asia,,1979,5.234

Southern Asia,,1980,5.176

Southern Asia,,1981,5.116

Southern Asia,,1982,5.051

Southern Asia,,1983,4.98

Southern Asia,,1984,4.901

Southern Asia,,1985,4.814

Southern Asia,,1986,4.72

Southern Asia,,1987,4.619

Southern Asia,,1988,4.514

Southern Asia,,1989,4.408

Southern Asia,,1990,4.301

Southern Asia,,1991,4.197

Southern Asia,,1992,4.095

Southern Asia,,1993,3.997

Southern Asia,,1994,3.903

Southern Asia,,1995,3.813

Southern Asia,,1996,3.727

Southern Asia,,1997,3.642

Southern Asia,,1998,3.558

Southern Asia,,1999,3.475

Southern Asia,,2000,3.393

Southern Asia,,2001,3.312

Southern Asia,,2002,3.234

Southern Asia,,2003,3.159

Southern Asia,,2004,3.086

Southern Asia,,2005,3.016

Southern Asia,,2006,2.947

Southern Asia,,2007,2.879

Southern Asia,,2008,2.812

Southern Asia,,2009,2.745

Southern Asia,,2010,2.681

Southern Asia,,2011,2.622

Southern Asia,,2012,2.568

Southern Asia,,2013,2.522

Southern Asia,,2014,2.483

Southern Asia,,2015,2.449

Southern Europe,,1950,2.753

Southern Europe,,1951,2.732

Southern Europe,,1952,2.693

Southern Europe,,1953,2.662

Southern Europe,,1954,2.64

Southern Europe,,1955,2.626

Southern Europe,,1956,2.619

Southern Europe,,1957,2.62

Southern Europe,,1958,2.628

Southern Europe,,1959,2.64

Southern Europe,,1960,2.656

Southern Europe,,1961,2.673

Southern Europe,,1962,2.688

Southern Europe,,1963,2.7

Southern Europe,,1964,2.707

Southern Europe,,1965,2.708

Southern Europe,,1966,2.703

Southern Europe,,1967,2.695

Southern Europe,,1968,2.682

Southern Europe,,1969,2.666

Southern Europe,,1970,2.643

Southern Europe,,1971,2.614

Southern Europe,,1972,2.578

Southern Europe,,1973,2.534

Southern Europe,,1974,2.482

Southern Europe,,1975,2.421

Southern Europe,,1976,2.353

Southern Europe,,1977,2.278

Southern Europe,,1978,2.198

Southern Europe,,1979,2.115

Southern Europe,,1980,2.031

Southern Europe,,1981,1.95

Southern Europe,,1982,1.872

Southern Europe,,1983,1.799

Southern Europe,,1984,1.733

Southern Europe,,1985,1.674

Southern Europe,,1986,1.622

Southern Europe,,1987,1.577

Southern Europe,,1988,1.537

Southern Europe,,1989,1.502

Southern Europe,,1990,1.471

Southern Europe,,1991,1.444

Southern Europe,,1992,1.42

Southern Europe,,1993,1.4

Southern Europe,,1994,1.382

Southern Europe,,1995,1.367

Southern Europe,,1996,1.355

Southern Europe,,1997,1.347

Southern Europe,,1998,1.343

Southern Europe,,1999,1.343

Southern Europe,,2000,1.347

Southern Europe,,2001,1.355

Southern Europe,,2002,1.367

Southern Europe,,2003,1.381

Southern Europe,,2004,1.397

Southern Europe,,2005,1.412

Southern Europe,,2006,1.424

Southern Europe,,2007,1.432

Southern Europe,,2008,1.436

Southern Europe,,2009,1.436

Southern Europe,,2010,1.433

Southern Europe,,2011,1.428

Southern Europe,,2012,1.425

Southern Europe,,2013,1.423

Southern Europe,,2014,1.425

Southern Europe,,2015,1.43

Spain,ESP,1950,2.449

Spain,ESP,1951,2.47

Spain,ESP,1952,2.512

Spain,ESP,1953,2.551

Spain,ESP,1954,2.589

Spain,ESP,1955,2.624

Spain,ESP,1956,2.657

Spain,ESP,1957,2.688

Spain,ESP,1958,2.717

Spain,ESP,1959,2.743

Spain,ESP,1960,2.766

Spain,ESP,1961,2.786

Spain,ESP,1962,2.802

Spain,ESP,1963,2.816

Spain,ESP,1964,2.826

Spain,ESP,1965,2.834

Spain,ESP,1966,2.842

Spain,ESP,1967,2.851

Spain,ESP,1968,2.86

Spain,ESP,1969,2.868

Spain,ESP,1970,2.872

Spain,ESP,1971,2.87

Spain,ESP,1972,2.86

Spain,ESP,1973,2.839

Spain,ESP,1974,2.804

Spain,ESP,1975,2.751

Spain,ESP,1976,2.679

Spain,ESP,1977,2.587

Spain,ESP,1978,2.478

Spain,ESP,1979,2.355

Spain,ESP,1980,2.224

Spain,ESP,1981,2.091

Spain,ESP,1982,1.96

Spain,ESP,1983,1.838

Spain,ESP,1984,1.728

Spain,ESP,1985,1.633

Spain,ESP,1986,1.554

Spain,ESP,1987,1.487

Spain,ESP,1988,1.431

Spain,ESP,1989,1.383

Spain,ESP,1990,1.343

Spain,ESP,1991,1.309

Spain,ESP,1992,1.28

Spain,ESP,1993,1.254

Spain,ESP,1994,1.231

Spain,ESP,1995,1.213

Spain,ESP,1996,1.2

Spain,ESP,1997,1.195

Spain,ESP,1998,1.198

Spain,ESP,1999,1.208

Spain,ESP,2000,1.225

Spain,ESP,2001,1.248

Spain,ESP,2002,1.275

Spain,ESP,2003,1.303

Spain,ESP,2004,1.331

Spain,ESP,2005,1.354

Spain,ESP,2006,1.37

Spain,ESP,2007,1.379

Spain,ESP,2008,1.38

Spain,ESP,2009,1.375

Spain,ESP,2010,1.365

Spain,ESP,2011,1.354

Spain,ESP,2012,1.346

Spain,ESP,2013,1.342

Spain,ESP,2014,1.344

Spain,ESP,2015,1.352

Sri Lanka,LKA,1950,5.635

Sri Lanka,LKA,1951,5.691

Sri Lanka,LKA,1952,5.789

Sri Lanka,LKA,1953,5.857

Sri Lanka,LKA,1954,5.893

Sri Lanka,LKA,1955,5.9

Sri Lanka,LKA,1956,5.876

Sri Lanka,LKA,1957,5.825

Sri Lanka,LKA,1958,5.749

Sri Lanka,LKA,1959,5.652

Sri Lanka,LKA,1960,5.541

Sri Lanka,LKA,1961,5.421

Sri Lanka,LKA,1962,5.299

Sri Lanka,LKA,1963,5.18

Sri Lanka,LKA,1964,5.067

Sri Lanka,LKA,1965,4.959

Sri Lanka,LKA,1966,4.849

Sri Lanka,LKA,1967,4.734

Sri Lanka,LKA,1968,4.609

Sri Lanka,LKA,1969,4.477

Sri Lanka,LKA,1970,4.342

Sri Lanka,LKA,1971,4.208

Sri Lanka,LKA,1972,4.083

Sri Lanka,LKA,1973,3.97

Sri Lanka,LKA,1974,3.87

Sri Lanka,LKA,1975,3.783

Sri Lanka,LKA,1976,3.705

Sri Lanka,LKA,1977,3.633

Sri Lanka,LKA,1978,3.562

Sri Lanka,LKA,1979,3.488

Sri Lanka,LKA,1980,3.408

Sri Lanka,LKA,1981,3.319

Sri Lanka,LKA,1982,3.223

Sri Lanka,LKA,1983,3.12

Sri Lanka,LKA,1984,3.012

Sri Lanka,LKA,1985,2.904

Sri Lanka,LKA,1986,2.8

Sri Lanka,LKA,1987,2.703

Sri Lanka,LKA,1988,2.617

Sri Lanka,LKA,1989,2.544

Sri Lanka,LKA,1990,2.483

Sri Lanka,LKA,1991,2.433

Sri Lanka,LKA,1992,2.39

Sri Lanka,LKA,1993,2.353

Sri Lanka,LKA,1994,2.32

Sri Lanka,LKA,1995,2.291

Sri Lanka,LKA,1996,2.268

Sri Lanka,LKA,1997,2.252

Sri Lanka,LKA,1998,2.242

Sri Lanka,LKA,1999,2.239

Sri Lanka,LKA,2000,2.241

Sri Lanka,LKA,2001,2.247

Sri Lanka,LKA,2002,2.257

Sri Lanka,LKA,2003,2.268

Sri Lanka,LKA,2004,2.278

Sri Lanka,LKA,2005,2.284

Sri Lanka,LKA,2006,2.283

Sri Lanka,LKA,2007,2.274

Sri Lanka,LKA,2008,2.257

Sri Lanka,LKA,2009,2.232

Sri Lanka,LKA,2010,2.203

Sri Lanka,LKA,2011,2.17

Sri Lanka,LKA,2012,2.138

Sri Lanka,LKA,2013,2.109

Sri Lanka,LKA,2014,2.083

Sri Lanka,LKA,2015,2.063

Sub-Saharan Africa,,1950,6.568

Sub-Saharan Africa,,1951,6.567

Sub-Saharan Africa,,1952,6.566

Sub-Saharan Africa,,1953,6.567

Sub-Saharan Africa,,1954,6.571

Sub-Saharan Africa,,1955,6.577

Sub-Saharan Africa,,1956,6.585

Sub-Saharan Africa,,1957,6.596

Sub-Saharan Africa,,1958,6.608

Sub-Saharan Africa,,1959,6.621

Sub-Saharan Africa,,1960,6.634

Sub-Saharan Africa,,1961,6.646

Sub-Saharan Africa,,1962,6.657

Sub-Saharan Africa,,1963,6.666

Sub-Saharan Africa,,1964,6.674

Sub-Saharan Africa,,1965,6.681

Sub-Saharan Africa,,1966,6.688

Sub-Saharan Africa,,1967,6.697

Sub-Saharan Africa,,1968,6.708

Sub-Saharan Africa,,1969,6.721

Sub-Saharan Africa,,1970,6.736

Sub-Saharan Africa,,1971,6.75

Sub-Saharan Africa,,1972,6.764

Sub-Saharan Africa,,1973,6.775

Sub-Saharan Africa,,1974,6.783

Sub-Saharan Africa,,1975,6.788

Sub-Saharan Africa,,1976,6.788

Sub-Saharan Africa,,1977,6.786

Sub-Saharan Africa,,1978,6.78

Sub-Saharan Africa,,1979,6.77

Sub-Saharan Africa,,1980,6.757

Sub-Saharan Africa,,1981,6.739

Sub-Saharan Africa,,1982,6.716

Sub-Saharan Africa,,1983,6.688

Sub-Saharan Africa,,1984,6.654

Sub-Saharan Africa,,1985,6.614

Sub-Saharan Africa,,1986,6.568

Sub-Saharan Africa,,1987,6.515

Sub-Saharan Africa,,1988,6.456

Sub-Saharan Africa,,1989,6.394

Sub-Saharan Africa,,1990,6.329

Sub-Saharan Africa,,1991,6.263

Sub-Saharan Africa,,1992,6.199

Sub-Saharan Africa,,1993,6.137

Sub-Saharan Africa,,1994,6.078

Sub-Saharan Africa,,1995,6.022

Sub-Saharan Africa,,1996,5.969

Sub-Saharan Africa,,1997,5.919

Sub-Saharan Africa,,1998,5.87

Sub-Saharan Africa,,1999,5.82

Sub-Saharan Africa,,2000,5.772

Sub-Saharan Africa,,2001,5.723

Sub-Saharan Africa,,2002,5.675

Sub-Saharan Africa,,2003,5.626

Sub-Saharan Africa,,2004,5.577

Sub-Saharan Africa,,2005,5.528

Sub-Saharan Africa,,2006,5.477

Sub-Saharan Africa,,2007,5.424

Sub-Saharan Africa,,2008,5.37

Sub-Saharan Africa,,2009,5.313

Sub-Saharan Africa,,2010,5.254

Sub-Saharan Africa,,2011,5.193

Sub-Saharan Africa,,2012,5.129

Sub-Saharan Africa,,2013,5.063

Sub-Saharan Africa,,2014,4.995

Sub-Saharan Africa,,2015,4.927

Sudan,SDN,1950,6.675

Sudan,SDN,1951,6.667

Sudan,SDN,1952,6.652

Sudan,SDN,1953,6.641

Sudan,SDN,1954,6.635

Sudan,SDN,1955,6.634

Sudan,SDN,1956,6.637

Sudan,SDN,1957,6.645

Sudan,SDN,1958,6.657

Sudan,SDN,1959,6.672

Sudan,SDN,1960,6.691

Sudan,SDN,1961,6.713

Sudan,SDN,1962,6.737

Sudan,SDN,1963,6.762

Sudan,SDN,1964,6.787

Sudan,SDN,1965,6.81

Sudan,SDN,1966,6.831

Sudan,SDN,1967,6.85

Sudan,SDN,1968,6.865

Sudan,SDN,1969,6.878

Sudan,SDN,1970,6.888

Sudan,SDN,1971,6.899

Sudan,SDN,1972,6.91

Sudan,SDN,1973,6.921

Sudan,SDN,1974,6.93

Sudan,SDN,1975,6.936

Sudan,SDN,1976,6.933

Sudan,SDN,1977,6.919

Sudan,SDN,1978,6.892

Sudan,SDN,1979,6.853

Sudan,SDN,1980,6.802

Sudan,SDN,1981,6.741

Sudan,SDN,1982,6.674

Sudan,SDN,1983,6.605

Sudan,SDN,1984,6.535

Sudan,SDN,1985,6.467

Sudan,SDN,1986,6.401

Sudan,SDN,1987,6.337

Sudan,SDN,1988,6.274

Sudan,SDN,1989,6.213

Sudan,SDN,1990,6.152

Sudan,SDN,1991,6.091

Sudan,SDN,1992,6.028

Sudan,SDN,1993,5.964

Sudan,SDN,1994,5.897

Sudan,SDN,1995,5.829

Sudan,SDN,1996,5.758

Sudan,SDN,1997,5.686

Sudan,SDN,1998,5.614

Sudan,SDN,1999,5.542

Sudan,SDN,2000,5.471

Sudan,SDN,2001,5.402

Sudan,SDN,2002,5.334

Sudan,SDN,2003,5.268

Sudan,SDN,2004,5.204

Sudan,SDN,2005,5.143

Sudan,SDN,2006,5.085

Sudan,SDN,2007,5.03

Sudan,SDN,2008,4.977

Sudan,SDN,2009,4.926

Sudan,SDN,2010,4.876

Sudan,SDN,2011,4.825

Sudan,SDN,2012,4.771

Sudan,SDN,2013,4.715

Sudan,SDN,2014,4.656

Sudan,SDN,2015,4.595

Suriname,SUR,1950,6.582

Suriname,SUR,1951,6.573

Suriname,SUR,1952,6.559

Suriname,SUR,1953,6.551

Suriname,SUR,1954,6.55

Suriname,SUR,1955,6.555

Suriname,SUR,1956,6.566

Suriname,SUR,1957,6.58

Suriname,SUR,1958,6.595

Suriname,SUR,1959,6.606

Suriname,SUR,1960,6.608

Suriname,SUR,1961,6.594

Suriname,SUR,1962,6.558

Suriname,SUR,1963,6.497

Suriname,SUR,1964,6.412

Suriname,SUR,1965,6.304

Suriname,SUR,1966,6.182

Suriname,SUR,1967,6.053

Suriname,SUR,1968,5.922

Suriname,SUR,1969,5.79

Suriname,SUR,1970,5.653

Suriname,SUR,1971,5.504

Suriname,SUR,1972,5.336

Suriname,SUR,1973,5.148

Suriname,SUR,1974,4.945

Suriname,SUR,1975,4.734

Suriname,SUR,1976,4.526

Suriname,SUR,1977,4.332

Suriname,SUR,1978,4.161

Suriname,SUR,1979,4.015

Suriname,SUR,1980,3.895

Suriname,SUR,1981,3.798

Suriname,SUR,1982,3.715

Suriname,SUR,1983,3.64

Suriname,SUR,1984,3.57

Suriname,SUR,1985,3.505

Suriname,SUR,1986,3.445

Suriname,SUR,1987,3.391

Suriname,SUR,1988,3.345

Suriname,SUR,1989,3.304

Suriname,SUR,1990,3.267

Suriname,SUR,1991,3.232

Suriname,SUR,1992,3.197

Suriname,SUR,1993,3.16

Suriname,SUR,1994,3.12

Suriname,SUR,1995,3.077

Suriname,SUR,1996,3.033

Suriname,SUR,1997,2.988

Suriname,SUR,1998,2.944

Suriname,SUR,1999,2.902

Suriname,SUR,2000,2.861

Suriname,SUR,2001,2.821

Suriname,SUR,2002,2.783

Suriname,SUR,2003,2.745

Suriname,SUR,2004,2.708

Suriname,SUR,2005,2.672

Suriname,SUR,2006,2.638

Suriname,SUR,2007,2.606

Suriname,SUR,2008,2.575

Suriname,SUR,2009,2.547

Suriname,SUR,2010,2.52

Suriname,SUR,2011,2.495

Suriname,SUR,2012,2.47

Suriname,SUR,2013,2.445

Suriname,SUR,2014,2.421

Suriname,SUR,2015,2.396

Swaziland,SWZ,1950,6.711

Swaziland,SWZ,1951,6.707

Swaziland,SWZ,1952,6.701

Swaziland,SWZ,1953,6.697

Swaziland,SWZ,1954,6.694

Swaziland,SWZ,1955,6.693

Swaziland,SWZ,1956,6.693

Swaziland,SWZ,1957,6.696

Swaziland,SWZ,1958,6.701

Swaziland,SWZ,1959,6.708

Swaziland,SWZ,1960,6.717

Swaziland,SWZ,1961,6.73

Swaziland,SWZ,1962,6.745

Swaziland,SWZ,1963,6.763

Swaziland,SWZ,1964,6.782

Swaziland,SWZ,1965,6.802

Swaziland,SWZ,1966,6.822

Swaziland,SWZ,1967,6.841

Swaziland,SWZ,1968,6.857

Swaziland,SWZ,1969,6.869

Swaziland,SWZ,1970,6.875

Swaziland,SWZ,1971,6.876

Swaziland,SWZ,1972,6.871

Swaziland,SWZ,1973,6.86

Swaziland,SWZ,1974,6.843

Swaziland,SWZ,1975,6.82

Swaziland,SWZ,1976,6.793

Swaziland,SWZ,1977,6.762

Swaziland,SWZ,1978,6.728

Swaziland,SWZ,1979,6.69

Swaziland,SWZ,1980,6.646

Swaziland,SWZ,1981,6.596

Swaziland,SWZ,1982,6.537

Swaziland,SWZ,1983,6.468

Swaziland,SWZ,1984,6.387

Swaziland,SWZ,1985,6.294

Swaziland,SWZ,1986,6.185

Swaziland,SWZ,1987,6.061

Swaziland,SWZ,1988,5.924

Swaziland,SWZ,1989,5.776

Swaziland,SWZ,1990,5.619

Swaziland,SWZ,1991,5.455

Swaziland,SWZ,1992,5.287

Swaziland,SWZ,1993,5.119

Swaziland,SWZ,1994,4.955

Swaziland,SWZ,1995,4.798

Swaziland,SWZ,1996,4.652

Swaziland,SWZ,1997,4.518

Swaziland,SWZ,1998,4.394

Swaziland,SWZ,1999,4.284

Swaziland,SWZ,2000,4.187

Swaziland,SWZ,2001,4.105

Swaziland,SWZ,2002,4.036

Swaziland,SWZ,2003,3.977

Swaziland,SWZ,2004,3.925

Swaziland,SWZ,2005,3.873

Swaziland,SWZ,2006,3.819

Swaziland,SWZ,2007,3.758

Swaziland,SWZ,2008,3.689

Swaziland,SWZ,2009,3.611

Swaziland,SWZ,2010,3.527

Swaziland,SWZ,2011,3.44

Swaziland,SWZ,2012,3.355

Swaziland,SWZ,2013,3.276

Swaziland,SWZ,2014,3.204

Swaziland,SWZ,2015,3.141

Sweden,SWE,1950,2.254

Sweden,SWE,1951,2.247

Sweden,SWE,1952,2.237

Sweden,SWE,1953,2.231

Sweden,SWE,1954,2.229

Sweden,SWE,1955,2.232

Sweden,SWE,1956,2.239

Sweden,SWE,1957,2.25

Sweden,SWE,1958,2.263

Sweden,SWE,1959,2.278

Sweden,SWE,1960,2.291

Sweden,SWE,1961,2.3

Sweden,SWE,1962,2.304

Sweden,SWE,1963,2.3

Sweden,SWE,1964,2.287

Sweden,SWE,1965,2.265

Sweden,SWE,1966,2.233

Sweden,SWE,1967,2.194

Sweden,SWE,1968,2.148

Sweden,SWE,1969,2.099

Sweden,SWE,1970,2.046

Sweden,SWE,1971,1.99

Sweden,SWE,1972,1.932

Sweden,SWE,1973,1.874

Sweden,SWE,1974,1.818

Sweden,SWE,1975,1.766

Sweden,SWE,1976,1.719

Sweden,SWE,1977,1.68

Sweden,SWE,1978,1.648

Sweden,SWE,1979,1.625

Sweden,SWE,1980,1.616

Sweden,SWE,1981,1.621

Sweden,SWE,1982,1.641

Sweden,SWE,1983,1.674

Sweden,SWE,1984,1.717

Sweden,SWE,1985,1.768

Sweden,SWE,1986,1.825

Sweden,SWE,1987,1.883

Sweden,SWE,1988,1.937

Sweden,SWE,1989,1.982

Sweden,SWE,1990,2.009

Sweden,SWE,1991,2.01

Sweden,SWE,1992,1.984

Sweden,SWE,1993,1.934

Sweden,SWE,1994,1.863

Sweden,SWE,1995,1.782

Sweden,SWE,1996,1.701

Sweden,SWE,1997,1.632

Sweden,SWE,1998,1.585

Sweden,SWE,1999,1.562

Sweden,SWE,2000,1.566

Sweden,SWE,2001,1.594

Sweden,SWE,2002,1.637

Sweden,SWE,2003,1.686

Sweden,SWE,2004,1.738

Sweden,SWE,2005,1.786

Sweden,SWE,2006,1.828

Sweden,SWE,2007,1.862

Sweden,SWE,2008,1.887

Sweden,SWE,2009,1.904

Sweden,SWE,2010,1.912

Sweden,SWE,2011,1.914

Sweden,SWE,2012,1.912

Sweden,SWE,2013,1.91

Sweden,SWE,2014,1.908

Sweden,SWE,2015,1.907

Switzerland,CHE,1950,2.325

Switzerland,CHE,1951,2.316

Switzerland,CHE,1952,2.303

Switzerland,CHE,1953,2.301

Switzerland,CHE,1954,2.309

Switzerland,CHE,1955,2.328

Switzerland,CHE,1956,2.356

Switzerland,CHE,1957,2.392

Switzerland,CHE,1958,2.434

Switzerland,CHE,1959,2.478

Switzerland,CHE,1960,2.52

Switzerland,CHE,1961,2.554

Switzerland,CHE,1962,2.575

Switzerland,CHE,1963,2.579

Switzerland,CHE,1964,2.564

Switzerland,CHE,1965,2.528

Switzerland,CHE,1966,2.471

Switzerland,CHE,1967,2.397

Switzerland,CHE,1968,2.312

Switzerland,CHE,1969,2.218

Switzerland,CHE,1970,2.121

Switzerland,CHE,1971,2.021

Switzerland,CHE,1972,1.923

Switzerland,CHE,1973,1.829

Switzerland,CHE,1974,1.743

Switzerland,CHE,1975,1.669

Switzerland,CHE,1976,1.61

Switzerland,CHE,1977,1.566

Switzerland,CHE,1978,1.535

Switzerland,CHE,1979,1.518

Switzerland,CHE,1980,1.512

Switzerland,CHE,1981,1.515

Switzerland,CHE,1982,1.522

Switzerland,CHE,1983,1.53

Switzerland,CHE,1984,1.537

Switzerland,CHE,1985,1.543

Switzerland,CHE,1986,1.546

Switzerland,CHE,1987,1.547

Switzerland,CHE,1988,1.548

Switzerland,CHE,1989,1.549

Switzerland,CHE,1990,1.548

Switzerland,CHE,1991,1.545

Switzerland,CHE,1992,1.541

Switzerland,CHE,1993,1.535

Switzerland,CHE,1994,1.527

Switzerland,CHE,1995,1.516

Switzerland,CHE,1996,1.503

Switzerland,CHE,1997,1.488

Switzerland,CHE,1998,1.47

Switzerland,CHE,1999,1.453

Switzerland,CHE,2000,1.437

Switzerland,CHE,2001,1.425

Switzerland,CHE,2002,1.418

Switzerland,CHE,2003,1.416

Switzerland,CHE,2004,1.42

Switzerland,CHE,2005,1.429

Switzerland,CHE,2006,1.442

Switzerland,CHE,2007,1.457

Switzerland,CHE,2008,1.472

Switzerland,CHE,2009,1.487

Switzerland,CHE,2010,1.5

Switzerland,CHE,2011,1.511

Switzerland,CHE,2012,1.52

Switzerland,CHE,2013,1.528

Switzerland,CHE,2014,1.535

Switzerland,CHE,2015,1.54

Syria,SYR,1950,7.176

Syria,SYR,1951,7.188

Syria,SYR,1952,7.212

Syria,SYR,1953,7.239

Syria,SYR,1954,7.267

Syria,SYR,1955,7.298

Syria,SYR,1956,7.33

Syria,SYR,1957,7.365

Syria,SYR,1958,7.399

Syria,SYR,1959,7.434

Syria,SYR,1960,7.467

Syria,SYR,1961,7.497

Syria,SYR,1962,7.522

Syria,SYR,1963,7.541

Syria,SYR,1964,7.555

Syria,SYR,1965,7.564

Syria,SYR,1966,7.569

Syria,SYR,1967,7.572

Syria,SYR,1968,7.574

Syria,SYR,1969,7.574

Syria,SYR,1970,7.572

Syria,SYR,1971,7.566

Syria,SYR,1972,7.556

Syria,SYR,1973,7.537

Syria,SYR,1974,7.51

Syria,SYR,1975,7.472

Syria,SYR,1976,7.422

Syria,SYR,1977,7.359

Syria,SYR,1978,7.285

Syria,SYR,1979,7.197

Syria,SYR,1980,7.094

Syria,SYR,1981,6.976

Syria,SYR,1982,6.843

Syria,SYR,1983,6.695

Syria,SYR,1984,6.534

Syria,SYR,1985,6.359

Syria,SYR,1986,6.168

Syria,SYR,1987,5.962

Syria,SYR,1988,5.746

Syria,SYR,1989,5.526

Syria,SYR,1990,5.31

Syria,SYR,1991,5.107

Syria,SYR,1992,4.923

Syria,SYR,1993,4.762

Syria,SYR,1994,4.625

Syria,SYR,1995,4.509

Syria,SYR,1996,4.41

Syria,SYR,1997,4.319

Syria,SYR,1998,4.229

Syria,SYR,1999,4.138

Syria,SYR,2000,4.043

Syria,SYR,2001,3.944

Syria,SYR,2002,3.844

Syria,SYR,2003,3.744

Syria,SYR,2004,3.648

Syria,SYR,2005,3.556

Syria,SYR,2006,3.471

Syria,SYR,2007,3.394

Syria,SYR,2008,3.325

Syria,SYR,2009,3.264

Syria,SYR,2010,3.21

Syria,SYR,2011,3.16

Syria,SYR,2012,3.113

Syria,SYR,2013,3.065

Syria,SYR,2014,3.016

Syria,SYR,2015,2.966

Taiwan,TWN,1950,6.902

Taiwan,TWN,1951,6.861

Taiwan,TWN,1952,6.774

Taiwan,TWN,1953,6.679

Taiwan,TWN,1954,6.575

Taiwan,TWN,1955,6.463

Taiwan,TWN,1956,6.342

Taiwan,TWN,1957,6.212

Taiwan,TWN,1958,6.074

Taiwan,TWN,1959,5.927

Taiwan,TWN,1960,5.772

Taiwan,TWN,1961,5.609

Taiwan,TWN,1962,5.438

Taiwan,TWN,1963,5.26

Taiwan,TWN,1964,5.076

Taiwan,TWN,1965,4.884

Taiwan,TWN,1966,4.683

Taiwan,TWN,1967,4.474

Taiwan,TWN,1968,4.259

Taiwan,TWN,1969,4.042

Taiwan,TWN,1970,3.829

Taiwan,TWN,1971,3.627

Taiwan,TWN,1972,3.441

Taiwan,TWN,1973,3.273

Taiwan,TWN,1974,3.125

Taiwan,TWN,1975,2.995

Taiwan,TWN,1976,2.88

Taiwan,TWN,1977,2.775

Taiwan,TWN,1978,2.674

Taiwan,TWN,1979,2.575

Taiwan,TWN,1980,2.475

Taiwan,TWN,1981,2.371

Taiwan,TWN,1982,2.265

Taiwan,TWN,1983,2.159

Taiwan,TWN,1984,2.057

Taiwan,TWN,1985,1.962

Taiwan,TWN,1986,1.88

Taiwan,TWN,1987,1.817

Taiwan,TWN,1988,1.772

Taiwan,TWN,1989,1.746

Taiwan,TWN,1990,1.735

Taiwan,TWN,1991,1.736

Taiwan,TWN,1992,1.742

Taiwan,TWN,1993,1.748

Taiwan,TWN,1994,1.747

Taiwan,TWN,1995,1.737

Taiwan,TWN,1996,1.713

Taiwan,TWN,1997,1.677

Taiwan,TWN,1998,1.631

Taiwan,TWN,1999,1.574

Taiwan,TWN,2000,1.51

Taiwan,TWN,2001,1.438

Taiwan,TWN,2002,1.363

Taiwan,TWN,2003,1.288

Taiwan,TWN,2004,1.219

Taiwan,TWN,2005,1.158

Taiwan,TWN,2006,1.11

Taiwan,TWN,2007,1.075

Taiwan,TWN,2008,1.053

Taiwan,TWN,2009,1.045

Taiwan,TWN,2010,1.048

Taiwan,TWN,2011,1.063

Taiwan,TWN,2012,1.084

Taiwan,TWN,2013,1.108

Taiwan,TWN,2014,1.133

Taiwan,TWN,2015,1.158

Tajikistan,TJK,1950,5.039

Tajikistan,TJK,1951,5.132

Tajikistan,TJK,1952,5.316

Tajikistan,TJK,1953,5.493

Tajikistan,TJK,1954,5.665

Tajikistan,TJK,1955,5.83

Tajikistan,TJK,1956,5.989

Tajikistan,TJK,1957,6.142

Tajikistan,TJK,1958,6.286

Tajikistan,TJK,1959,6.422

Tajikistan,TJK,1960,6.547

Tajikistan,TJK,1961,6.661

Tajikistan,TJK,1962,6.76

Tajikistan,TJK,1963,6.844

Tajikistan,TJK,1964,6.911

Tajikistan,TJK,1965,6.962

Tajikistan,TJK,1966,6.999

Tajikistan,TJK,1967,7.024

Tajikistan,TJK,1968,7.035

Tajikistan,TJK,1969,7.031

Tajikistan,TJK,1970,7.003

Tajikistan,TJK,1971,6.943

Tajikistan,TJK,1972,6.845

Tajikistan,TJK,1973,6.714

Tajikistan,TJK,1974,6.553

Tajikistan,TJK,1975,6.376

Tajikistan,TJK,1976,6.197

Tajikistan,TJK,1977,6.029

Tajikistan,TJK,1978,5.885

Tajikistan,TJK,1979,5.77

Tajikistan,TJK,1980,5.688

Tajikistan,TJK,1981,5.64

Tajikistan,TJK,1982,5.613

Tajikistan,TJK,1983,5.599

Tajikistan,TJK,1984,5.588

Tajikistan,TJK,1985,5.573

Tajikistan,TJK,1986,5.543

Tajikistan,TJK,1987,5.495

Tajikistan,TJK,1988,5.427

Tajikistan,TJK,1989,5.336

Tajikistan,TJK,1990,5.226

Tajikistan,TJK,1991,5.101

Tajikistan,TJK,1992,4.971

Tajikistan,TJK,1993,4.841

Tajikistan,TJK,1994,4.714

Tajikistan,TJK,1995,4.588

Tajikistan,TJK,1996,4.461

Tajikistan,TJK,1997,4.327

Tajikistan,TJK,1998,4.188

Tajikistan,TJK,1999,4.045

Tajikistan,TJK,2000,3.907

Tajikistan,TJK,2001,3.781

Tajikistan,TJK,2002,3.675

Tajikistan,TJK,2003,3.591

Tajikistan,TJK,2004,3.533

Tajikistan,TJK,2005,3.498

Tajikistan,TJK,2006,3.485

Tajikistan,TJK,2007,3.486

Tajikistan,TJK,2008,3.493

Tajikistan,TJK,2009,3.502

Tajikistan,TJK,2010,3.507

Tajikistan,TJK,2011,3.504

Tajikistan,TJK,2012,3.492

Tajikistan,TJK,2013,3.472

Tajikistan,TJK,2014,3.442

Tajikistan,TJK,2015,3.404

Tanzania,TZA,1950,6.699

Tanzania,TZA,1951,6.711

Tanzania,TZA,1952,6.733

Tanzania,TZA,1953,6.752

Tanzania,TZA,1954,6.768

Tanzania,TZA,1955,6.781

Tanzania,TZA,1956,6.791

Tanzania,TZA,1957,6.798

Tanzania,TZA,1958,6.803

Tanzania,TZA,1959,6.805

Tanzania,TZA,1960,6.806

Tanzania,TZA,1961,6.806

Tanzania,TZA,1962,6.804

Tanzania,TZA,1963,6.803

Tanzania,TZA,1964,6.801

Tanzania,TZA,1965,6.798

Tanzania,TZA,1966,6.795

Tanzania,TZA,1967,6.791

Tanzania,TZA,1968,6.785

Tanzania,TZA,1969,6.778

Tanzania,TZA,1970,6.771

Tanzania,TZA,1971,6.765

Tanzania,TZA,1972,6.761

Tanzania,TZA,1973,6.758

Tanzania,TZA,1974,6.755

Tanzania,TZA,1975,6.751

Tanzania,TZA,1976,6.743

Tanzania,TZA,1977,6.73

Tanzania,TZA,1978,6.711

Tanzania,TZA,1979,6.685

Tanzania,TZA,1980,6.653

Tanzania,TZA,1981,6.618

Tanzania,TZA,1982,6.58

Tanzania,TZA,1983,6.542

Tanzania,TZA,1984,6.504

Tanzania,TZA,1985,6.465

Tanzania,TZA,1986,6.424

Tanzania,TZA,1987,6.379

Tanzania,TZA,1988,6.328

Tanzania,TZA,1989,6.273

Tanzania,TZA,1990,6.213

Tanzania,TZA,1991,6.148

Tanzania,TZA,1992,6.081

Tanzania,TZA,1993,6.013

Tanzania,TZA,1994,5.946

Tanzania,TZA,1995,5.883

Tanzania,TZA,1996,5.828

Tanzania,TZA,1997,5.78

Tanzania,TZA,1998,5.741

Tanzania,TZA,1999,5.711

Tanzania,TZA,2000,5.689

Tanzania,TZA,2001,5.675

Tanzania,TZA,2002,5.666

Tanzania,TZA,2003,5.659

Tanzania,TZA,2004,5.651

Tanzania,TZA,2005,5.638

Tanzania,TZA,2006,5.616

Tanzania,TZA,2007,5.584

Tanzania,TZA,2008,5.542

Tanzania,TZA,2009,5.489

Tanzania,TZA,2010,5.427

Tanzania,TZA,2011,5.359

Tanzania,TZA,2012,5.287

Tanzania,TZA,2013,5.215

Tanzania,TZA,2014,5.146

Tanzania,TZA,2015,5.079

Thailand,THA,1950,6.132

Thailand,THA,1951,6.134

Thailand,THA,1952,6.137

Thailand,THA,1953,6.14

Thailand,THA,1954,6.141

Thailand,THA,1955,6.142

Thailand,THA,1956,6.143

Thailand,THA,1957,6.143

Thailand,THA,1958,6.143

Thailand,THA,1959,6.144

Thailand,THA,1960,6.147

Thailand,THA,1961,6.151

Thailand,THA,1962,6.155

Thailand,THA,1963,6.157

Thailand,THA,1964,6.151

Thailand,THA,1965,6.129

Thailand,THA,1966,6.084

Thailand,THA,1967,6.009

Thailand,THA,1968,5.903

Thailand,THA,1969,5.764

Thailand,THA,1970,5.595

Thailand,THA,1971,5.397

Thailand,THA,1972,5.181

Thailand,THA,1973,4.953

Thailand,THA,1974,4.721

Thailand,THA,1975,4.488

Thailand,THA,1976,4.257

Thailand,THA,1977,4.03

Thailand,THA,1978,3.808

Thailand,THA,1979,3.594

Thailand,THA,1980,3.392

Thailand,THA,1981,3.202

Thailand,THA,1982,3.024

Thailand,THA,1983,2.859

Thailand,THA,1984,2.707

Thailand,THA,1985,2.571

Thailand,THA,1986,2.451

Thailand,THA,1987,2.346

Thailand,THA,1988,2.256

Thailand,THA,1989,2.179

Thailand,THA,1990,2.113

Thailand,THA,1991,2.055

Thailand,THA,1992,2.003

Thailand,THA,1993,1.956

Thailand,THA,1994,1.911

Thailand,THA,1995,1.867

Thailand,THA,1996,1.823

Thailand,THA,1997,1.781

Thailand,THA,1998,1.742

Thailand,THA,1999,1.705

Thailand,THA,2000,1.671

Thailand,THA,2001,1.641

Thailand,THA,2002,1.616

Thailand,THA,2003,1.595

Thailand,THA,2004,1.58

Thailand,THA,2005,1.568

Thailand,THA,2006,1.561

Thailand,THA,2007,1.557

Thailand,THA,2008,1.553

Thailand,THA,2009,1.551

Thailand,THA,2010,1.547

Thailand,THA,2011,1.542

Thailand,THA,2012,1.534

Thailand,THA,2013,1.524

Thailand,THA,2014,1.512

Thailand,THA,2015,1.498

Timor,TLS,1950,6.509

Timor,TLS,1951,6.488

Timor,TLS,1952,6.451

Timor,TLS,1953,6.421

Timor,TLS,1954,6.397

Timor,TLS,1955,6.38

Timor,TLS,1956,6.369

Timor,TLS,1957,6.364

Timor,TLS,1958,6.365

Timor,TLS,1959,6.368

Timor,TLS,1960,6.373

Timor,TLS,1961,6.376

Timor,TLS,1962,6.375

Timor,TLS,1963,6.367

Timor,TLS,1964,6.347

Timor,TLS,1965,6.314

Timor,TLS,1966,6.268

Timor,TLS,1967,6.207

Timor,TLS,1968,6.132

Timor,TLS,1969,6.039

Timor,TLS,1970,5.917

Timor,TLS,1971,5.751

Timor,TLS,1972,5.541

Timor,TLS,1973,5.298

Timor,TLS,1974,5.037

Timor,TLS,1975,4.794

Timor,TLS,1976,4.611

Timor,TLS,1977,4.514

Timor,TLS,1978,4.513

Timor,TLS,1979,4.606

Timor,TLS,1980,4.767

Timor,TLS,1981,4.956

Timor,TLS,1982,5.13

Timor,TLS,1983,5.256

Timor,TLS,1984,5.324

Timor,TLS,1985,5.336

Timor,TLS,1986,5.308

Timor,TLS,1987,5.271

Timor,TLS,1988,5.256

Timor,TLS,1989,5.273

Timor,TLS,1990,5.34

Timor,TLS,1991,5.469

Timor,TLS,1992,5.651

Timor,TLS,1993,5.874

Timor,TLS,1994,6.125

Timor,TLS,1995,6.384

Timor,TLS,1996,6.627

Timor,TLS,1997,6.832

Timor,TLS,1998,6.986

Timor,TLS,1999,7.079

Timor,TLS,2000,7.112

Timor,TLS,2001,7.092

Timor,TLS,2002,7.037

Timor,TLS,2003,6.965

Timor,TLS,2004,6.881

Timor,TLS,2005,6.79

Timor,TLS,2006,6.694

Timor,TLS,2007,6.59

Timor,TLS,2008,6.477

Timor,TLS,2009,6.357

Timor,TLS,2010,6.234

Timor,TLS,2011,6.109

Timor,TLS,2012,5.983

Timor,TLS,2013,5.859

Timor,TLS,2014,5.737

Timor,TLS,2015,5.618

Togo,TGO,1950,6.335

Togo,TGO,1951,6.332

Togo,TGO,1952,6.328

Togo,TGO,1953,6.331

Togo,TGO,1954,6.34

Togo,TGO,1955,6.355

Togo,TGO,1956,6.377

Togo,TGO,1957,6.404

Togo,TGO,1958,6.438

Togo,TGO,1959,6.477

Togo,TGO,1960,6.521

Togo,TGO,1961,6.57

Togo,TGO,1962,6.622

Togo,TGO,1963,6.677

Togo,TGO,1964,6.734

Togo,TGO,1965,6.792

Togo,TGO,1966,6.851

Togo,TGO,1967,6.911

Togo,TGO,1968,6.971

Togo,TGO,1969,7.029

Togo,TGO,1970,7.084

Togo,TGO,1971,7.136

Togo,TGO,1972,7.182

Togo,TGO,1973,7.222

Togo,TGO,1974,7.254

Togo,TGO,1975,7.275

Togo,TGO,1976,7.286

Togo,TGO,1977,7.284

Togo,TGO,1978,7.27

Togo,TGO,1979,7.245

Togo,TGO,1980,7.207

Togo,TGO,1981,7.158

Togo,TGO,1982,7.099

Togo,TGO,1983,7.031

Togo,TGO,1984,6.954

Togo,TGO,1985,6.869

Togo,TGO,1986,6.775

Togo,TGO,1987,6.672

Togo,TGO,1988,6.561

Togo,TGO,1989,6.444

Togo,TGO,1990,6.324

Togo,TGO,1991,6.202

Togo,TGO,1992,6.082

Togo,TGO,1993,5.965

Togo,TGO,1994,5.855

Togo,TGO,1995,5.755

Togo,TGO,1996,5.666

Togo,TGO,1997,5.588

Togo,TGO,1998,5.52

Togo,TGO,1999,5.461

Togo,TGO,2000,5.41

Togo,TGO,2001,5.364

Togo,TGO,2002,5.32

Togo,TGO,2003,5.276

Togo,TGO,2004,5.229

Togo,TGO,2005,5.179

Togo,TGO,2006,5.123

Togo,TGO,2007,5.064

Togo,TGO,2008,5.001

Togo,TGO,2009,4.936

Togo,TGO,2010,4.868

Togo,TGO,2011,4.798

Togo,TGO,2012,4.727

Togo,TGO,2013,4.656

Togo,TGO,2014,4.586

Togo,TGO,2015,4.517

Tonga,TON,1950,7.327

Tonga,TON,1951,7.316

Tonga,TON,1952,7.299

Tonga,TON,1953,7.289

Tonga,TON,1954,7.288

Tonga,TON,1955,7.294

Tonga,TON,1956,7.308

Tonga,TON,1957,7.326

Tonga,TON,1958,7.345

Tonga,TON,1959,7.36

Tonga,TON,1960,7.363

Tonga,TON,1961,7.346

Tonga,TON,1962,7.302

Tonga,TON,1963,7.226

Tonga,TON,1964,7.117

Tonga,TON,1965,6.973

Tonga,TON,1966,6.795

Tonga,TON,1967,6.59

Tonga,TON,1968,6.371

Tonga,TON,1969,6.15

Tonga,TON,1970,5.941

Tonga,TON,1971,5.761

Tonga,TON,1972,5.616

Tonga,TON,1973,5.512

Tonga,TON,1974,5.449

Tonga,TON,1975,5.426

Tonga,TON,1976,5.438

Tonga,TON,1977,5.471

Tonga,TON,1978,5.509

Tonga,TON,1979,5.541

Tonga,TON,1980,5.553

Tonga,TON,1981,5.532

Tonga,TON,1982,5.475

Tonga,TON,1983,5.385

Tonga,TON,1984,5.266

Tonga,TON,1985,5.128

Tonga,TON,1986,4.989

Tonga,TON,1987,4.864

Tonga,TON,1988,4.763

Tonga,TON,1989,4.691

Tonga,TON,1990,4.644

Tonga,TON,1991,4.612

Tonga,TON,1992,4.583

Tonga,TON,1993,4.547

Tonga,TON,1994,4.501

Tonga,TON,1995,4.447

Tonga,TON,1996,4.391

Tonga,TON,1997,4.34

Tonga,TON,1998,4.3

Tonga,TON,1999,4.27

Tonga,TON,2000,4.25

Tonga,TON,2001,4.236

Tonga,TON,2002,4.221

Tonga,TON,2003,4.202

Tonga,TON,2004,4.177

Tonga,TON,2005,4.144

Tonga,TON,2006,4.104

Tonga,TON,2007,4.059

Tonga,TON,2008,4.012

Tonga,TON,2009,3.963

Tonga,TON,2010,3.913

Tonga,TON,2011,3.864

Tonga,TON,2012,3.815

Tonga,TON,2013,3.768

Tonga,TON,2014,3.722

Tonga,TON,2015,3.678

Trinidad and Tobago,TTO,1950,5.276

Trinidad and Tobago,TTO,1951,5.282

Trinidad and Tobago,TTO,1952,5.294

Trinidad and Tobago,TTO,1953,5.306

Trinidad and Tobago,TTO,1954,5.318

Trinidad and Tobago,TTO,1955,5.33

Trinidad and Tobago,TTO,1956,5.339

Trinidad and Tobago,TTO,1957,5.344

Trinidad and Tobago,TTO,1958,5.338

Trinidad and Tobago,TTO,1959,5.315

Trinidad and Tobago,TTO,1960,5.264

Trinidad and Tobago,TTO,1961,5.177

Trinidad and Tobago,TTO,1962,5.047

Trinidad and Tobago,TTO,1963,4.873

Trinidad and Tobago,TTO,1964,4.664

Trinidad and Tobago,TTO,1965,4.433

Trinidad and Tobago,TTO,1966,4.2

Trinidad and Tobago,TTO,1967,3.985

Trinidad and Tobago,TTO,1968,3.802

Trinidad and Tobago,TTO,1969,3.658

Trinidad and Tobago,TTO,1970,3.554

Trinidad and Tobago,TTO,1971,3.484

Trinidad and Tobago,TTO,1972,3.432

Trinidad and Tobago,TTO,1973,3.389

Trinidad and Tobago,TTO,1974,3.349

Trinidad and Tobago,TTO,1975,3.313

Trinidad and Tobago,TTO,1976,3.287

Trinidad and Tobago,TTO,1977,3.273

Trinidad and Tobago,TTO,1978,3.271

Trinidad and Tobago,TTO,1979,3.278

Trinidad and Tobago,TTO,1980,3.284

Trinidad and Tobago,TTO,1981,3.281

Trinidad and Tobago,TTO,1982,3.261

Trinidad and Tobago,TTO,1983,3.218

Trinidad and Tobago,TTO,1984,3.151

Trinidad and Tobago,TTO,1985,3.06

Trinidad and Tobago,TTO,1986,2.949

Trinidad and Tobago,TTO,1987,2.827

Trinidad and Tobago,TTO,1988,2.7

Trinidad and Tobago,TTO,1989,2.574

Trinidad and Tobago,TTO,1990,2.453

Trinidad and Tobago,TTO,1991,2.339

Trinidad and Tobago,TTO,1992,2.231

Trinidad and Tobago,TTO,1993,2.131

Trinidad and Tobago,TTO,1994,2.04

Trinidad and Tobago,TTO,1995,1.961

Trinidad and Tobago,TTO,1996,1.896

Trinidad and Tobago,TTO,1997,1.843

Trinidad and Tobago,TTO,1998,1.801

Trinidad and Tobago,TTO,1999,1.772

Trinidad and Tobago,TTO,2000,1.753

Trinidad and Tobago,TTO,2001,1.744

Trinidad and Tobago,TTO,2002,1.744

Trinidad and Tobago,TTO,2003,1.749

Trinidad and Tobago,TTO,2004,1.758

Trinidad and Tobago,TTO,2005,1.769

Trinidad and Tobago,TTO,2006,1.78

Trinidad and Tobago,TTO,2007,1.79

Trinidad and Tobago,TTO,2008,1.799

Trinidad and Tobago,TTO,2009,1.804

Trinidad and Tobago,TTO,2010,1.806

Trinidad and Tobago,TTO,2011,1.804

Trinidad and Tobago,TTO,2012,1.798

Trinidad and Tobago,TTO,2013,1.789

Trinidad and Tobago,TTO,2014,1.778

Trinidad and Tobago,TTO,2015,1.766

Tunisia,TUN,1950,6.557

Tunisia,TUN,1951,6.581

Tunisia,TUN,1952,6.628

Tunisia,TUN,1953,6.674

Tunisia,TUN,1954,6.718

Tunisia,TUN,1955,6.76

Tunisia,TUN,1956,6.801

Tunisia,TUN,1957,6.84

Tunisia,TUN,1958,6.876

Tunisia,TUN,1959,6.911

Tunisia,TUN,1960,6.942

Tunisia,TUN,1961,6.97

Tunisia,TUN,1962,6.993

Tunisia,TUN,1963,7.009

Tunisia,TUN,1964,7.015

Tunisia,TUN,1965,7.008

Tunisia,TUN,1966,6.984

Tunisia,TUN,1967,6.942

Tunisia,TUN,1968,6.881

Tunisia,TUN,1969,6.802

Tunisia,TUN,1970,6.705

Tunisia,TUN,1971,6.591

Tunisia,TUN,1972,6.465

Tunisia,TUN,1973,6.33

Tunisia,TUN,1974,6.189

Tunisia,TUN,1975,6.042

Tunisia,TUN,1976,5.89

Tunisia,TUN,1977,5.733

Tunisia,TUN,1978,5.572

Tunisia,TUN,1979,5.408

Tunisia,TUN,1980,5.243

Tunisia,TUN,1981,5.078

Tunisia,TUN,1982,4.915

Tunisia,TUN,1983,4.752

Tunisia,TUN,1984,4.59

Tunisia,TUN,1985,4.425

Tunisia,TUN,1986,4.252

Tunisia,TUN,1987,4.07

Tunisia,TUN,1988,3.878

Tunisia,TUN,1989,3.678

Tunisia,TUN,1990,3.476

Tunisia,TUN,1991,3.276

Tunisia,TUN,1992,3.085

Tunisia,TUN,1993,2.907

Tunisia,TUN,1994,2.747

Tunisia,TUN,1995,2.606

Tunisia,TUN,1996,2.485

Tunisia,TUN,1997,2.379

Tunisia,TUN,1998,2.287

Tunisia,TUN,1999,2.208

Tunisia,TUN,2000,2.142

Tunisia,TUN,2001,2.088

Tunisia,TUN,2002,2.046

Tunisia,TUN,2003,2.016

Tunisia,TUN,2004,1.997

Tunisia,TUN,2005,1.991

Tunisia,TUN,2006,1.999

Tunisia,TUN,2007,2.022

Tunisia,TUN,2008,2.057

Tunisia,TUN,2009,2.099

Tunisia,TUN,2010,2.144

Tunisia,TUN,2011,2.184

Tunisia,TUN,2012,2.214

Tunisia,TUN,2013,2.231

Tunisia,TUN,2014,2.234

Tunisia,TUN,2015,2.223

Turkey,TUR,1950,6.738

Turkey,TUR,1951,6.727

Turkey,TUR,1952,6.703

Turkey,TUR,1953,6.675

Turkey,TUR,1954,6.643

Turkey,TUR,1955,6.607

Turkey,TUR,1956,6.567

Turkey,TUR,1957,6.523

Turkey,TUR,1958,6.475

Turkey,TUR,1959,6.423

Turkey,TUR,1960,6.366

Turkey,TUR,1961,6.304

Turkey,TUR,1962,6.237

Turkey,TUR,1963,6.165

Turkey,TUR,1964,6.089

Turkey,TUR,1965,6.011

Turkey,TUR,1966,5.933

Turkey,TUR,1967,5.855

Turkey,TUR,1968,5.778

Turkey,TUR,1969,5.7

Turkey,TUR,1970,5.619

Turkey,TUR,1971,5.529

Turkey,TUR,1972,5.429

Turkey,TUR,1973,5.316

Turkey,TUR,1974,5.191

Turkey,TUR,1975,5.058

Turkey,TUR,1976,4.921

Turkey,TUR,1977,4.785

Turkey,TUR,1978,4.654

Turkey,TUR,1979,4.528

Turkey,TUR,1980,4.405

Turkey,TUR,1981,4.282

Turkey,TUR,1982,4.155

Turkey,TUR,1983,4.021

Turkey,TUR,1984,3.883

Turkey,TUR,1985,3.741

Turkey,TUR,1986,3.598

Turkey,TUR,1987,3.461

Turkey,TUR,1988,3.331

Turkey,TUR,1989,3.213

Turkey,TUR,1990,3.107

Turkey,TUR,1991,3.016

Turkey,TUR,1992,2.937

Turkey,TUR,1993,2.868

Turkey,TUR,1994,2.809

Turkey,TUR,1995,2.755

Turkey,TUR,1996,2.705

Turkey,TUR,1997,2.657

Turkey,TUR,1998,2.607

Turkey,TUR,1999,2.556

Turkey,TUR,2000,2.503

Turkey,TUR,2001,2.451

Turkey,TUR,2002,2.4

Turkey,TUR,2003,2.353

Turkey,TUR,2004,2.31

Turkey,TUR,2005,2.273

Turkey,TUR,2006,2.241

Turkey,TUR,2007,2.214

Turkey,TUR,2008,2.192

Turkey,TUR,2009,2.172

Turkey,TUR,2010,2.155

Turkey,TUR,2011,2.139

Turkey,TUR,2012,2.124

Turkey,TUR,2013,2.108

Turkey,TUR,2014,2.09

Turkey,TUR,2015,2.072

Turkmenistan,TKM,1950,4.89

Turkmenistan,TKM,1951,4.995

Turkmenistan,TKM,1952,5.203

Turkmenistan,TKM,1953,5.405

Turkmenistan,TKM,1954,5.602

Turkmenistan,TKM,1955,5.793

Turkmenistan,TKM,1956,5.976

Turkmenistan,TKM,1957,6.152

Turkmenistan,TKM,1958,6.315

Turkmenistan,TKM,1959,6.463

Turkmenistan,TKM,1960,6.59

Turkmenistan,TKM,1961,6.689

Turkmenistan,TKM,1962,6.756

Turkmenistan,TKM,1963,6.788

Turkmenistan,TKM,1964,6.787

Turkmenistan,TKM,1965,6.756

Turkmenistan,TKM,1966,6.703

Turkmenistan,TKM,1967,6.635

Turkmenistan,TKM,1968,6.56

Turkmenistan,TKM,1969,6.482

Turkmenistan,TKM,1970,6.404

Turkmenistan,TKM,1971,6.323

Turkmenistan,TKM,1972,6.237

Turkmenistan,TKM,1973,6.143

Turkmenistan,TKM,1974,6.04

Turkmenistan,TKM,1975,5.926

Turkmenistan,TKM,1976,5.796

Turkmenistan,TKM,1977,5.651

Turkmenistan,TKM,1978,5.494

Turkmenistan,TKM,1979,5.331

Turkmenistan,TKM,1980,5.17

Turkmenistan,TKM,1981,5.022

Turkmenistan,TKM,1982,4.895

Turkmenistan,TKM,1983,4.791

Turkmenistan,TKM,1984,4.711

Turkmenistan,TKM,1985,4.65

Turkmenistan,TKM,1986,4.603

Turkmenistan,TKM,1987,4.557

Turkmenistan,TKM,1988,4.504

Turkmenistan,TKM,1989,4.436

Turkmenistan,TKM,1990,4.344

Turkmenistan,TKM,1991,4.222

Turkmenistan,TKM,1992,4.071

Turkmenistan,TKM,1993,3.898

Turkmenistan,TKM,1994,3.707

Turkmenistan,TKM,1995,3.512

Turkmenistan,TKM,1996,3.323

Turkmenistan,TKM,1997,3.154

Turkmenistan,TKM,1998,3.013

Turkmenistan,TKM,1999,2.903

Turkmenistan,TKM,2000,2.824

Turkmenistan,TKM,2001,2.77

Turkmenistan,TKM,2002,2.73

Turkmenistan,TKM,2003,2.698

Turkmenistan,TKM,2004,2.671

Turkmenistan,TKM,2005,2.654

Turkmenistan,TKM,2006,2.653

Turkmenistan,TKM,2007,2.672

Turkmenistan,TKM,2008,2.712

Turkmenistan,TKM,2009,2.769

Turkmenistan,TKM,2010,2.833

Turkmenistan,TKM,2011,2.892

Turkmenistan,TKM,2012,2.936

Turkmenistan,TKM,2013,2.958

Turkmenistan,TKM,2014,2.956

Turkmenistan,TKM,2015,2.931

Uganda,UGA,1950,6.893

Uganda,UGA,1951,6.894

Uganda,UGA,1952,6.896

Uganda,UGA,1953,6.9

Uganda,UGA,1954,6.907

Uganda,UGA,1955,6.917

Uganda,UGA,1956,6.929

Uganda,UGA,1957,6.944

Uganda,UGA,1958,6.96

Uganda,UGA,1959,6.979

Uganda,UGA,1960,6.999

Uganda,UGA,1961,7.019

Uganda,UGA,1962,7.04

Uganda,UGA,1963,7.06

Uganda,UGA,1964,7.078

Uganda,UGA,1965,7.094

Uganda,UGA,1966,7.106

Uganda,UGA,1967,7.113

Uganda,UGA,1968,7.117

Uganda,UGA,1969,7.118

Uganda,UGA,1970,7.115

Uganda,UGA,1971,7.111

Uganda,UGA,1972,7.107

Uganda,UGA,1973,7.103

Uganda,UGA,1974,7.1

Uganda,UGA,1975,7.099

Uganda,UGA,1976,7.099

Uganda,UGA,1977,7.099

Uganda,UGA,1978,7.099

Uganda,UGA,1979,7.1

Uganda,UGA,1980,7.1

Uganda,UGA,1981,7.101

Uganda,UGA,1982,7.101

Uganda,UGA,1983,7.102

Uganda,UGA,1984,7.103

Uganda,UGA,1985,7.103

Uganda,UGA,1986,7.103

Uganda,UGA,1987,7.102

Uganda,UGA,1988,7.1

Uganda,UGA,1989,7.097

Uganda,UGA,1990,7.091

Uganda,UGA,1991,7.082

Uganda,UGA,1992,7.071

Uganda,UGA,1993,7.057

Uganda,UGA,1994,7.039

Uganda,UGA,1995,7.018

Uganda,UGA,1996,6.994

Uganda,UGA,1997,6.967

Uganda,UGA,1998,6.937

Uganda,UGA,1999,6.903

Uganda,UGA,2000,6.865

Uganda,UGA,2001,6.822

Uganda,UGA,2002,6.772

Uganda,UGA,2003,6.716

Uganda,UGA,2004,6.653

Uganda,UGA,2005,6.583

Uganda,UGA,2006,6.507

Uganda,UGA,2007,6.424

Uganda,UGA,2008,6.337

Uganda,UGA,2009,6.247

Uganda,UGA,2010,6.154

Uganda,UGA,2011,6.059

Uganda,UGA,2012,5.964

Uganda,UGA,2013,5.869

Uganda,UGA,2014,5.775

Uganda,UGA,2015,5.682

Ukraine,UKR,1950,2.718

Ukraine,UKR,1951,2.754

Ukraine,UKR,1952,2.814

Ukraine,UKR,1953,2.846

Ukraine,UKR,1954,2.852

Ukraine,UKR,1955,2.832

Ukraine,UKR,1956,2.787

Ukraine,UKR,1957,2.718

Ukraine,UKR,1958,2.629

Ukraine,UKR,1959,2.527

Ukraine,UKR,1960,2.418

Ukraine,UKR,1961,2.31

Ukraine,UKR,1962,2.212

Ukraine,UKR,1963,2.131

Ukraine,UKR,1964,2.07

Ukraine,UKR,1965,2.032

Ukraine,UKR,1966,2.015

Ukraine,UKR,1967,2.014

Ukraine,UKR,1968,2.021

Ukraine,UKR,1969,2.034

Ukraine,UKR,1970,2.047

Ukraine,UKR,1971,2.056

Ukraine,UKR,1972,2.059

Ukraine,UKR,1973,2.056

Ukraine,UKR,1974,2.046

Ukraine,UKR,1975,2.032

Ukraine,UKR,1976,2.015

Ukraine,UKR,1977,2.001

Ukraine,UKR,1978,1.992

Ukraine,UKR,1979,1.988

Ukraine,UKR,1980,1.99

Ukraine,UKR,1981,1.996

Ukraine,UKR,1982,2.003

Ukraine,UKR,1983,2.009

Ukraine,UKR,1984,2.01

Ukraine,UKR,1985,2.004

Ukraine,UKR,1986,1.987

Ukraine,UKR,1987,1.96

Ukraine,UKR,1988,1.921

Ukraine,UKR,1989,1.871

Ukraine,UKR,1990,1.81

Ukraine,UKR,1991,1.738

Ukraine,UKR,1992,1.659

Ukraine,UKR,1993,1.576

Ukraine,UKR,1994,1.493

Ukraine,UKR,1995,1.412

Ukraine,UKR,1996,1.338

Ukraine,UKR,1997,1.272

Ukraine,UKR,1998,1.216

Ukraine,UKR,1999,1.173

Ukraine,UKR,2000,1.146

Ukraine,UKR,2001,1.137

Ukraine,UKR,2002,1.146

Ukraine,UKR,2003,1.169

Ukraine,UKR,2004,1.204

Ukraine,UKR,2005,1.248

Ukraine,UKR,2006,1.295

Ukraine,UKR,2007,1.342

Ukraine,UKR,2008,1.384

Ukraine,UKR,2009,1.42

Ukraine,UKR,2010,1.448

Ukraine,UKR,2011,1.469

Ukraine,UKR,2012,1.486

Ukraine,UKR,2013,1.501

Ukraine,UKR,2014,1.514

Ukraine,UKR,2015,1.528

United Arab Emirates,ARE,1950,6.941

United Arab Emirates,ARE,1951,6.951

United Arab Emirates,ARE,1952,6.968

United Arab Emirates,ARE,1953,6.98

United Arab Emirates,ARE,1954,6.986

United Arab Emirates,ARE,1955,6.987

United Arab Emirates,ARE,1956,6.983

United Arab Emirates,ARE,1957,6.975

United Arab Emirates,ARE,1958,6.962

United Arab Emirates,ARE,1959,6.946

United Arab Emirates,ARE,1960,6.929

United Arab Emirates,ARE,1961,6.91

United Arab Emirates,ARE,1962,6.892

United Arab Emirates,ARE,1963,6.875

United Arab Emirates,ARE,1964,6.857

United Arab Emirates,ARE,1965,6.838

United Arab Emirates,ARE,1966,6.817

United Arab Emirates,ARE,1967,6.791

United Arab Emirates,ARE,1968,6.757

United Arab Emirates,ARE,1969,6.713

United Arab Emirates,ARE,1970,6.655

United Arab Emirates,ARE,1971,6.579

United Arab Emirates,ARE,1972,6.484

United Arab Emirates,ARE,1973,6.372

United Arab Emirates,ARE,1974,6.245

United Arab Emirates,ARE,1975,6.111

United Arab Emirates,ARE,1976,5.973

United Arab Emirates,ARE,1977,5.84

United Arab Emirates,ARE,1978,5.716

United Arab Emirates,ARE,1979,5.603

United Arab Emirates,ARE,1980,5.505

United Arab Emirates,ARE,1981,5.422

United Arab Emirates,ARE,1982,5.35

United Arab Emirates,ARE,1983,5.283

United Arab Emirates,ARE,1984,5.215

United Arab Emirates,ARE,1985,5.139

United Arab Emirates,ARE,1986,5.047

United Arab Emirates,ARE,1987,4.934

United Arab Emirates,ARE,1988,4.797

United Arab Emirates,ARE,1989,4.636

United Arab Emirates,ARE,1990,4.454

United Arab Emirates,ARE,1991,4.253

United Arab Emirates,ARE,1992,4.041

United Arab Emirates,ARE,1993,3.827

United Arab Emirates,ARE,1994,3.618

United Arab Emirates,ARE,1995,3.418

United Arab Emirates,ARE,1996,3.232

United Arab Emirates,ARE,1997,3.062

United Arab Emirates,ARE,1998,2.907

United Arab Emirates,ARE,1999,2.768

United Arab Emirates,ARE,2000,2.644

United Arab Emirates,ARE,2001,2.532

United Arab Emirates,ARE,2002,2.428

United Arab Emirates,ARE,2003,2.33

United Arab Emirates,ARE,2004,2.238

United Arab Emirates,ARE,2005,2.151

United Arab Emirates,ARE,2006,2.073

United Arab Emirates,ARE,2007,2.005

United Arab Emirates,ARE,2008,1.949

United Arab Emirates,ARE,2009,1.904

United Arab Emirates,ARE,2010,1.869

United Arab Emirates,ARE,2011,1.841

United Arab Emirates,ARE,2012,1.82

United Arab Emirates,ARE,2013,1.801

United Arab Emirates,ARE,2014,1.783

United Arab Emirates,ARE,2015,1.765

United Kingdom,GBR,1950,2.076

United Kingdom,GBR,1951,2.099

United Kingdom,GBR,1952,2.148

United Kingdom,GBR,1953,2.203

United Kingdom,GBR,1954,2.264

United Kingdom,GBR,1955,2.33

United Kingdom,GBR,1956,2.402

United Kingdom,GBR,1957,2.477

United Kingdom,GBR,1958,2.553

United Kingdom,GBR,1959,2.627

United Kingdom,GBR,1960,2.694

United Kingdom,GBR,1961,2.748

United Kingdom,GBR,1962,2.785

United Kingdom,GBR,1963,2.8

United Kingdom,GBR,1964,2.791

United Kingdom,GBR,1965,2.757

United Kingdom,GBR,1966,2.696

United Kingdom,GBR,1967,2.613

United Kingdom,GBR,1968,2.515

United Kingdom,GBR,1969,2.407

United Kingdom,GBR,1970,2.294

United Kingdom,GBR,1971,2.182

United Kingdom,GBR,1972,2.074

United Kingdom,GBR,1973,1.976

United Kingdom,GBR,1974,1.892

United Kingdom,GBR,1975,1.825

United Kingdom,GBR,1976,1.776

United Kingdom,GBR,1977,1.744

United Kingdom,GBR,1978,1.727

United Kingdom,GBR,1979,1.721

United Kingdom,GBR,1980,1.726

United Kingdom,GBR,1981,1.739

United Kingdom,GBR,1982,1.757

United Kingdom,GBR,1983,1.777

United Kingdom,GBR,1984,1.796

United Kingdom,GBR,1985,1.812

United Kingdom,GBR,1986,1.822

United Kingdom,GBR,1987,1.827

United Kingdom,GBR,1988,1.827

United Kingdom,GBR,1989,1.822

United Kingdom,GBR,1990,1.813

United Kingdom,GBR,1991,1.801

United Kingdom,GBR,1992,1.789

United Kingdom,GBR,1993,1.777

United Kingdom,GBR,1994,1.766

United Kingdom,GBR,1995,1.756

United Kingdom,GBR,1996,1.744

United Kingdom,GBR,1997,1.728

United Kingdom,GBR,1998,1.71

United Kingdom,GBR,1999,1.691

United Kingdom,GBR,2000,1.676

United Kingdom,GBR,2001,1.669

United Kingdom,GBR,2002,1.674

United Kingdom,GBR,2003,1.691

United Kingdom,GBR,2004,1.721

United Kingdom,GBR,2005,1.758

United Kingdom,GBR,2006,1.798

United Kingdom,GBR,2007,1.835

United Kingdom,GBR,2008,1.864

United Kingdom,GBR,2009,1.883

United Kingdom,GBR,2010,1.892

United Kingdom,GBR,2011,1.893

United Kingdom,GBR,2012,1.889

United Kingdom,GBR,2013,1.884

United Kingdom,GBR,2014,1.879

United Kingdom,GBR,2015,1.876

United States,USA,1950,3.052

United States,USA,1951,3.13

United States,USA,1952,3.272

United States,USA,1953,3.389

United States,USA,1954,3.479

United States,USA,1955,3.544

United States,USA,1956,3.582

United States,USA,1957,3.595

United States,USA,1958,3.583

United States,USA,1959,3.545

United States,USA,1960,3.483

United States,USA,1961,3.399

United States,USA,1962,3.294

United States,USA,1963,3.173

United States,USA,1964,3.041

United States,USA,1965,2.902

United States,USA,1966,2.762

United States,USA,1967,2.623

United States,USA,1968,2.49

United States,USA,1969,2.366

United States,USA,1970,2.253

United States,USA,1971,2.153

United States,USA,1972,2.063

United States,USA,1973,1.984

United States,USA,1974,1.915

United States,USA,1975,1.858

United States,USA,1976,1.814

United States,USA,1977,1.783

United States,USA,1978,1.764

United States,USA,1979,1.757

United States,USA,1980,1.759

United States,USA,1981,1.769

United States,USA,1982,1.786

United States,USA,1983,1.806

United States,USA,1984,1.829

United States,USA,1985,1.853

United States,USA,1986,1.878

United States,USA,1987,1.904

United States,USA,1988,1.932

United States,USA,1989,1.958

United States,USA,1990,1.983

United States,USA,1991,2.002

United States,USA,1992,2.015

United States,USA,1993,2.021

United States,USA,1994,2.022

United States,USA,1995,2.019

United States,USA,1996,2.013

United States,USA,1997,2.009

United States,USA,1998,2.008

United States,USA,1999,2.011

United States,USA,2000,2.017

United States,USA,2001,2.027

United States,USA,2002,2.039

United States,USA,2003,2.05

United States,USA,2004,2.058

United States,USA,2005,2.062

United States,USA,2006,2.057

United States,USA,2007,2.044

United States,USA,2008,2.023

United States,USA,2009,1.995

United States,USA,2010,1.963

United States,USA,2011,1.932

United States,USA,2012,1.905

United States,USA,2013,1.884

United States,USA,2014,1.872

United States,USA,2015,1.868

United States Virgin Islands,VIR,1950,5.142

United States Virgin Islands,VIR,1951,5.17

United States Virgin Islands,VIR,1952,5.225

United States Virgin Islands,VIR,1953,5.279

United States Virgin Islands,VIR,1954,5.331

United States Virgin Islands,VIR,1955,5.383

United States Virgin Islands,VIR,1956,5.433

United States Virgin Islands,VIR,1957,5.482

United States Virgin Islands,VIR,1958,5.529

United States Virgin Islands,VIR,1959,5.574

United States Virgin Islands,VIR,1960,5.615

United States Virgin Islands,VIR,1961,5.651

United States Virgin Islands,VIR,1962,5.679

United States Virgin Islands,VIR,1963,5.696

United States Virgin Islands,VIR,1964,5.698

United States Virgin Islands,VIR,1965,5.679

United States Virgin Islands,VIR,1966,5.634

United States Virgin Islands,VIR,1967,5.561

United States Virgin Islands,VIR,1968,5.459

United States Virgin Islands,VIR,1969,5.328

United States Virgin Islands,VIR,1970,5.167

United States Virgin Islands,VIR,1971,4.976

United States Virgin Islands,VIR,1972,4.76

United States Virgin Islands,VIR,1973,4.527

United States Virgin Islands,VIR,1974,4.286

United States Virgin Islands,VIR,1975,4.047
[truncated: 26,637 more chars]
