# Supplementary material for: The impacts of parity on lung function data (LFD) of healthy females aged 40 years and more issued from an upper middle income country (Algeria): A comparative study
Source: PLoS One. 2019 Nov 8;14(11):e0225067. doi: 10.1371/journal.pone.0225067 (PMC6839841; doi:10.1371/journal.pone.0225067)
Supplement: S2 File — (DOCX) [file pone.0225067.s002.docx]

| **S2 File. Studies evaluating the effects of parity on lung function data (LFD) of healthy females and: designs and results.** | | | | | | | |
| --- | --- | --- | --- | --- | --- | --- | --- |
| ***1^st^ author*** | **Harik-Khan [1]** | **Ben Saad [2]** | **Ben Saad [3]** | **Ben Saad [4, 5]** | **Omorogiuwa and Iyawe [6]** | **Pastro [7]** | **Omorogiuwa and Osazee [8]** |
| ***Aims:*** to analyze the | .Effects of parity on FEV_1_ | .Relationship between parity and LFD | .Effects of parity on LFD | .Relationship between parity and ELA | .Spirometric data in all trimesters (T) of pregnancy as parity increases | .Influence of parity on LFD of pregnant females | .LFD of pregnant females in T_3_ with some lying down positions viz a viz parity |
| ***Country*** | .USA | .Tunisia | .Tunisia | .Tunisia | .Nigeria | .Brazil | .Nigeria |
| ***Race/ethnicity*** | .Caucasian white | .Arab white | .Arab white | .Arab white | .Black | .Latin American (white/brown/black) | .Black |
| ***Number*** | .397 | .123 | .108 | .364 | .200 | .120 | .150 |
| ***Age (Y)*** | .48 (18-92)**^a^**  .Younger (≤50 Y): 57.4**^f^**  **^.^**Older (>50 Y):42.6**^f^** | .69±7**^b^**  .60-96**^c^** | .50 (47-55)**^d^**  .45-90**^c^** | .50±11 (49-52)^e^  .19-90^c^ | .Primigravida: 24±2**^b^**  .Nullipara: 24±2**^b^**  .Primipara: 26±3**^b^**  .Para_2_: 26±3**^b^**  .Para_3_: 30±4**^b^** | .26±7^b^ | .21-28**^c^** |
| ***Parity data*** | .NR | .NR | .4±2**^b^** | .5±3 (4-5)^e^ | .NR | .NR | .NR |
| ***Parity groups or classes*** | **.2 groups:**  G_1_: 0: 35^f^  G_2_: ≥ 1: 65^f^ | .**2 groups:**  G_1_: ≤ 4: 22^f^  G_2_: > 4: 78^f^ | **.2 groups:**  G_1_: ≤ 3: 49.1^f^  G_2_: ≥ 4: 50.9^f^  **.3 classes:**  C_1_: < 2: 12.0^f^  C_2_: 3-4: 58.3^f^  C_3_: > 4: 29.7^f^ | .NA | **.5 groups:**  Primigravida: 20^f^  Nullipara: 20^f^  Primipara: 20^f^  Para_2_: 20^f^  Para_3_: 20^f^ | **.2 groups:**  G_1_: 0: 45^f^  G_2_: ≥ 1: 55^f^ | **.5 groups:**  Primigravida: 20^f^  Nullipara: 20^f^  Primipara: 20^f^  Para_2_: 20^f^  Para_3_: 20^f^ |
| ***Parity: other details*** | .1.5**^f^**: twin births  .2.5**^f^**: 3-4 children  .7.0**^f^** of younger group: 3-4 children |  |  |  |  | ≤25 Y-0:33.3^f^  ≤25 Y-≥ 1:16.7^f^  >25 Y-0:11.7^f^  >25 Y-≥ 1:38.3^f^ | .No multiple pregnancy |
| ***Characteristics*** | .Good health  .Well-educated  .Current smokers: 15.6**^f^**  .Never smokers: 53.1**^f^** | .Healthy  .Never smokers | .Healthy  .Ex-smokers (% NR)  .No current smokers | .Healthy  .Never smokers  .Normal spirometric data  .Normal weight:24**^f^**  .Overweight:49**^f^**  .Moderate obesity:27**^f^** | .Never smokers  .Hemoglobinemia > 10 mg/dl  .Apparently healthy volunteer (***eg***, no surgery, bad obstetric, caesarean) | .Healthy  .Singleton pregnancy .Gestational age > 14 weeks at T_1_  .No abortion  .Normal spirometric data at T_1_ and T_3_ | .Non-smoker  .Apparently healthy volunteer (**eg**, no: pregnancy induced hypertension, diabetes) |
| ***Performed tests*** | .Spirometry | .Spirometry | .Plethysmography  .Respiratory muscle strength | .Spirometry | .Spirometry | .Spirometry | .Spirometry in sitting (control) and supine, left lateral and right lateral positions (tests) |
| ***Collected data*** | .FEV_1_ | .FVC, FEV_1_, FEV_1_/FVC, MMEF, PEF | .FVC, FEV_1_, PEF, FEF_x%_, MMEF, SVC, FEV_1_/FVC, FEV_1_/SVC, TGV, TLC, RV, MIP, Gawtot | .ELA, CLA  .FVC, FEV_1_, MMEF, FEF_x%_, PEF, FEV_1_/FVC | .FEV_1_, FVC | .FVC, FEV_1_, FEV_1_/FVC, MMEF, PEF | .FEV_1_, FVC |
| **S2 File. Continued.** | | | | | | | |
| ***1^st^ author*** | **Harik-Khan [1]** | **Ben Saad [2]** | **Ben Saad [3]** | **Ben Saad [4, 5]** | **Omorogiuwa and Iyawe [6]** | **Pastro [7]** | **Omorogiuwa and Osazee [8]** |
| ***Main results*** | **Parity and FEV_1_ (L)**  .Parity (numeric): correlation with FEV_1_ (entire cohort (r=0.0285), younger group (r=0.0528).  .Parity (dichotomous): increased the correlation (entire cohort (r=0.0934), younger group (r=0.1392).  .Effects could not account for by the educational level, occupation, health status, presence of a cohort effect.  **Parity and FEV_1_ (%) - younger group**  .Among age, height, smoking status, weight, cohort group, education, occupation and currently homemaker, **only parity** was found to be significant on FEV_1_  .Incremental increase in FEV_1_ (%): largest after the 1^st^ child birth.  **Parity and FVC**  .Parity (numeric): correlation (r=NR).  .Parity (dichotomous): “r” = 0.144 (younger group) and = 0.164 (older group) | .Parity: not correlated with FVC, FEV_1_, MMEF or PEF  .FVC: similar values between G_1_ and G_2_.  .Compared to the G_2_, the G_1_ had higher FEV_1_ (1.99±0.57 vs.2.25±0.39^b^), FEV_1_/FVC (0.81±0.07 vs. 0.84±0.07^b^), MMEF (2.90±1.06 vs. 3.42±0.93^b^) and PEF (4.94±1.69 vs. 5.67±1.35^b^) | .Parity: negative correlation with FVC, FEV_1_, FEV_1_/SVC, PEF, FEF_75%_, FEF_50%_, MMEF, SVC.  .Parity introduced in the multiple regression models of the above spirometric data, increased the r^2^ values  .Females aged 45-49 or 50-54 or 55-59 Y: no difference between G_1_ and G_2_.  .G_2_ females aged ≥ 60 Y: tendency for bronchial obstruction (decrease of FEV_1_/SVC and FEV_1_/FVC) and decrease in MIP.  .Comparison between the 3 classes of parity: with a high parity, there is a tendency for bronchial obstruction (decrease of FEV_1_/FVC and Gawtot) without an associated restriction  .Parity: independent effect | .CLA: correlation with parity (r=0.51)  .Parity: positive independent data included in the multiple regression models for ELA:  ELA (Y) = 52.8 - 3.6 x FEV_1_ + **1.2 x Parity** + 1.1 x PEF - 1.5 x MMEF - 3.3 x FVC + 11.3 x Height (m) - 0.2 x BMI (kg/m^2^)- 0.5 x FEF_25%_ | .Increase in FVC and FEV_1_ as parity increases.  .FVC and FEV_1_ were increased in Para_3_ when compared to primigravid control females | **Data (absolute values)**  .FEV_1_/FVC and MMEF: lower in the G_1_ when compared to the G_2_ during T_1_ (0.82±0.06 vs. 0.86±0.05^b^; 3.21±0.91 vs. 3.66±0.80^b^) and during T_3_ (0.83±0.05 vs. 0.86±0.05^b^; 3.25±0.79 vs. 3.56±0.73^b^, respectively)  .Females ≤ 25 Y: **i)** FEV_1_/FVC lower in the G_2_ when compared to the G_1_ during T_1_ (0.83±0.05 vs. 0.87±0.05^b^) and during T_3_ (0.83±0.04 vs. 0.86±0.05^b^); **ii)** MMEF lower in the G_2_ when compared to the G_1_ during T_1_ (3.23±0.67 vs. 3.72±0.83^b^).  .Females > 25 Y: FEV_1_/FVC lower in the G_2_ when compared to the G_1_ during T_1_ (0.81±0.06 vs. 0.85±0.04^b^)  **Data (% predicted)**  .Similar values between the 2 groups  .Females ≤ 25 Y: **i)** MMEF lower in the G_2_ when compared to the G_1_ during T_1_ (83±18 vs. 97±22^b^); **ii)** PEF lower in the G_2_ when compared to the G_1_ during T_3_ (71±12 vs. 80±13^b^)  .Females > 25 Y: similar values between the 2 groups | **.**FVC and FEV_1_ in the lateral positions: higher than the supine position across parities  .Increase in FVC and FEV_1_ as the parity increases across all lying down position. |
| **S2 File. Continued.** | | | | | | | |
| ***1^st^ author*** | **Harik-Khan [1]** | **Ben Saad [2]** | **Ben Saad [3]** | **Ben Saad [4, 5]** | **Omorogiuwa and Iyawe [6]** | **Pastro [7]** | **Omorogiuwa and Osazee [8]** |
| ***Conclusions*** | .Parity: association with larger FEV_1_ and FVC during the younger age. | .High parity: association with a reduction in peak flow rate. | .Lung function declines with increasing parity. | .Parity accelerated the lung aging. | .Increased parity favorably affects pulmonary function | .Parity influenced spirometric data. | .Parity: a cumulative positive effect on spirometric data across the postures studied |
| **BMI:** body mass index. **C:** class. **CLA:** chronological lung age. **ELA:** estimated lung age. **FEF_X%_:** forced expiratory flow when X% of FVC has been exhaled. **FEV_1_:** forced expiratory volume in 1 s. **FVC:** forced vital capacity. **G:** group. **GAWtot:** total airway conductance. **MIP:** maximal inspiratory pressure. **MMEF:** maximal mid-expiratory flow. **NA:** not applied. **NR:** not reported. **Nullipara:** pregnant but previous pregnancies terminated before the age of viability. **Para_2_:** pregnant females who have carried 2 previous pregnancies beyond the age of viability. **Para_3_:** pregnant females who have carried 3 previous pregnancies beyond the age of viability. **PEF:** peak expiratory flow. **Primigravida:** pregnant for the 1^st^ time irrespective of the gestational age. **Primipara:** pregnancy carried beyond the age of viability irrespective of the outcome. **r:** coefficient of correlation. **r^2^:** coefficient of determination. **RV:** residual volume. **SVC:** slow vital capacity. **T_1_:** 1^st^ trimester. **T_3_:** 3^rd^ trimester. **TGV:** thoracic gas volume. **TLC:** total lung capacity. **Y:** year.  Data were: **^a^**Mean (minimum-maximum). **^b^**Mean±SD. **^c^**Minimum-maximum. **^d^**Median (lower-upper quartiles). ^e^Mean (95% confidence interval). **^f^**Percentage. | | | | | | | |

**References**

**1.** Harik-Khan R, Wise RA, Lou C, Morrell CH, Brant LJ, Fozard JL. The effect of gestational parity on FEV1 in a group of healthy volunteer women. Respiratory medicine. 1999;93(6):382-8. Epub 1999/08/28. doi: 10.1053/rmed.1999.0572. PubMed PMID: 10464819.

**2.** Ben Saad H, Rouatbi S, Raoudha S, Tabka Z, Laouani Kechrid C, Hassen G, et al. [Vital capacity and peak expiratory flow rates in a North-African population aged 60 years and over: influence of anthropometric data and parity]. Revue des maladies respiratoires. 2003;20(4):521-30. Epub 2003/10/07. PubMed PMID: 14528154.

**3.** Ben Saad H, Tfifha M, Harrabi I, Tabka Z, Guenard H, Hayot M, et al. [Factors influencing pulmonary function in Tunisian women aged 45 years and more]. Revue des maladies respiratoires. 2006;23(4 Pt 1):324-38. Epub 2006/11/28. PubMed PMID: 17127908.

**4.** Ben Saad H, Selmi H, Hadj Mabrouk K, Gargouri I, Nouira A, Said Latiri H, et al. Spirometric “Lung Age” estimation for North African population. Egyptian Journal of Chest Diseases and Tuberculosis. 2014;63(2):491-503. doi: 10.1016/j.ejcdt.2014.01.003.

**5.** Ben Saad H, Elhraiech A, Hadj Mabrouk K, Ben Mdalla S, Essghaier M, Maatoug C, et al. Estimated lung age in healthy North African adults cannot be predicted using reference equations derived from other populations. Egyptian Journal of Chest Diseases and Tuberculosis. 2013;62(4):789-804. doi: 10.1016/j.ejcdt.2013.09.018.

**6.** Omorogiuwa O, Iyawe V. Effect of Parity on FVC and FEV1 during Pregnancy. British Journal of Medicine & Medical Research. 2015;9(8):1-9.

**7.** Pastro LDM, Lemos M, Fernandes FLA, Saldiva S, Vieira SE, Romanholo BMS, et al. Longitudinal study of lung function in pregnant women: Influence of parity and smoking. Clinics. 2017;72(10):595-9. Epub 2017/11/22. doi: 10.6061/clinics/2017(10)02. PubMed PMID: 29160421; PubMed Central PMCID: PMCPMC5666444.

**8.** Omorogiuwa A, Osazee K. Effect of posture on pulmonary function in the third trimester of pregnancy of primigravid, nulliparous and multiparous women in Benin city. Ibom Medical Journal. 2018;11(2):68-76.
