# Supplementary material for: The impacts of parity on lung function data (LFD) of healthy females aged 40 years and more issued from an upper middle income country (Algeria): A comparative study
Source: PLoS One. 2019 Nov 8;14(11):e0225067. doi: 10.1371/journal.pone.0225067 (PMC6839841; doi:10.1371/journal.pone.0225067)
Supplement: S3 File — (DOCX) [file pone.0225067.s003.docx]

**S3 File: DISCUSSION**

**Methodology discussion**

The variability in the results for lung aging can be explained by the type of epidemiological study, with a larger decline in longitudinal studies **[1]**. This would be due to the cohort effect (environmental and individual factors, such as loss of height) and period (change of techniques and instruments) encountered in the longitudinal study and the effect of selection in the cross sectional and/or comparative studies **[2]**. However, comparative studies (like this one), are economical, easy to apply, requires less time and provides several useful information **[1]**. In order to avoid/minimize biased assessment of outcomes, two actions were performed **[3]**. First, all females, who reported being healthy, were interviewed via a medical questionnaire **[4]** to detect a possible non-inclusion criterion **[3]**. Secondly, the 66 females were randomly chosen from the total sample of females (n=123) aged ≥ 40 years with at least one parity **[3]**. In general, studies exploring lung aging are of interest to people aged ≥ 65 **[5]**, based on a SEL criterion. However, since the decline in LFD could begin at an early age, between 35 and 45 years **[6]**, this study design opted for the limit of 40 years. Two reasons were advanced to explain the applied cutoff of six parities. First, it was created based on the literature related to the effects of parity on cardiorespiratory health **[7-18] (S2 File)**. Some studies **[13, 19-21]** indicated that females with six or more parities have worse lung function and/or PAL. Secondly, among the 244 females included in the whole project, the parity’ mean±SD of the 123 females aged ≥ 40 years and having at least one parity was 6.4±3.5.

The non-inclusion criteria were respected. The exact definition of the "healthy" group is difficult to establish on elderly subjects. According to the ATS/ERS, to be considered “healthy”, the subject must be non-smoker and free from any chronic cardiorespiratory diseases and any respiratory symptomatology **[22]**. Consequently, females with known cardiorespiratory diseases weren’t included. Since smoking accelerated the decline of LFD **[23]**, current or ex-smokers of more than one pack-year weren’t included. This was contrary to some previous related studies where current-smokers (15.6% in an American study **[7]**) or ex-smokers (percentage not reported in a Tunisian study **[24]**) were included **(S2 File)**. Females with chronic diseases or under chronic medications (***eg***, corticosteroids, β-blockers, nervous system stimulant, muscle relaxant or hormone replacement therapy) weren’t included, because such chronic conditions/specific treatments significantly affect and/or interfere with LFD **[5, 25]**. Females with a BMI < 18.5 or > 34.5 kg/m^2^ weren’t included. Leanness indicates malnutrition or chronic pathology leading to pulmonary functional deficiency **[26]**, and obesity ≥ stage 2 is associated with lung impairment in females without any confirmed pulmonary disease **[27]**. In this study, almost 41% of females were obese stage 1. On the one hand, this was similar to the percentage reported in the Tunisian study **[24]**, where 42% of females were obese. On the other hand, 30.1% of the Algerian females aged 35-70 years showed obesity **[28]**, and this study group composition reflected this “healthy” females as they exist in the real Algerian population. This increases the external validity of this study.

The following females’ LFD influencing factors weren’t evaluated: passive smoking, antioxidant vitamin consumption, pollution, occupational exposure, bronchial hyperreactivity, peripheral muscle strength and forearm circumference **[2]**. It is possible that other factors not considered in this study or yet unclear may improve understanding of the LFD difference observed between the two groups and the three classes of females. For example, standing height, the major determinant of LFD **[29]**, declines with advanced age due to vertebral compression for example **[30]**. For that reason, it was better to measure the arm span that varies much less than height with age **[31]**. Recently, one study concluded that some LFD (***ie***, FVC) declined more rapidly among transitional and post-menopausal females, beyond the expected age change **[32]**. Since the G_1_ and the G_2_ included similar percentages of menopauses females **(Table 2)**, the difference between their TGV, TLC and RV can’t be explained by their menopause status.

**REFERENCES**

**1.** Ware JH, Dockery DW, Louis TA, Xu XP, Ferris BG, Jr., Speizer FE. Longitudinal and cross-sectional estimates of pulmonary function decline in never-smoking adults. American journal of epidemiology. 1990;132(4):685-700. Epub 1990/10/01. doi: 10.1093/oxfordjournals.aje.a115710. PubMed PMID: 2403109.

**2.** Guénard H, Rouatbi S. Physiological aspects of the decline of pulmonary function with age. Revue des maladies respiratoires. 2004;21(5 III):8S13-8S24.

**3.** Suresh K. An overview of randomization techniques: An unbiased assessment of outcome in clinical research. Journal of human reproductive sciences. 2011;4(1):8-11. Epub 2011/07/21. doi: 10.4103/0974-1208.82352. PubMed PMID: 21772732; PubMed Central PMCID: PMCPMC3136079.

**4.** Ferris BG. Epidemiology Standardization Project (American Thoracic Society). The American review of respiratory disease. 1978;118(6 Pt 2):1-120. Epub 1978/12/01. PubMed PMID: 742764.

**5.** Griffith KA, Sherrill DL, Siegel EM, Manolio TA, Bonekat HW, Enright PL. Predictors of loss of lung function in the elderly: the Cardiovascular Health Study. American journal of respiratory and critical care medicine. 2001;163(1):61-8. Epub 2001/02/24. doi: 10.1164/ajrccm.163.1.9906089. PubMed PMID: 11208627.

**6.** Sharma G, Goodwin J. Effect of aging on respiratory system physiology and immunology. Clinical interventions in aging. 2006;1(3):253-60. Epub 2007/12/01. PubMed PMID: 18046878; PubMed Central PMCID: PMCPMC2695176.

**7.** Harik-Khan R, Wise RA, Lou C, Morrell CH, Brant LJ, Fozard JL. The effect of gestational parity on FEV_1_ in a group of healthy volunteer women. Respiratory medicine. 1999;93(6):382-8. Epub 1999/08/28. doi: 10.1053/rmed.1999.0572. PubMed PMID: 10464819.

**8.** Ben Saad H, Selmi H, Hadj Mabrouk K, Gargouri I, Nouira A, Said Latiri H, et al. Spirometric “Lung Age” estimation for North African population. Egyptian Journal of Chest Diseases and Tuberculosis. 2014;63(2):491-503. doi: 10.1016/j.ejcdt.2014.01.003.

**9.** Ben Saad H, Elhraiech A, Hadj Mabrouk K, Ben Mdalla S, Essghaier M, Maatoug C, et al. Estimated lung age in healthy North African adults cannot be predicted using reference equations derived from other populations. Egyptian Journal of Chest Diseases and Tuberculosis. 2013;62(4):789-804. doi: 10.1016/j.ejcdt.2013.09.018.

**10.** Omorogiuwa O, Iyawe V. Effect of Parity on FVC and FEV_1_ during Pregnancy. British Journal of Medicine & Medical Research. 2015;9(8):1-9.

**11.** Pastro LDM, Lemos M, Fernandes FLA, Saldiva S, Vieira SE, Romanholo BMS, et al. Longitudinal study of lung function in pregnant women: Influence of parity and smoking. Clinics (Sao Paulo). 2017;72(10):595-9. Epub 2017/11/22. doi: 10.6061/clinics/2017(10)02. PubMed PMID: 29160421; PubMed Central PMCID: PMCPMC5666444.

**12.** Omorogiuwa A, Osazee K. Effect of posture on pulmonary function in the third trimester of pregnancy of primigravid, nulliparous and multiparous women in Benin city. Ibom Medical Journal. 2018;11(2):68-76.

**13.** Azevedo IG, da Camara SMA, Pirkle CM, Maciel ACC, Viana ESR. Relationship between maximal respiratory pressures and multiple childbearing in Brazilian middle-aged and older women: A cross-sectional community-based study. PLoS One. 2018;13(12):e0208500. Epub 2018/12/05. doi: 10.1371/journal.pone.0208500. PubMed PMID: 30513117; PubMed Central PMCID: PMCPMC6279230.

**14.** Camara SM, Pirkle C, Moreira MA, Vieira MC, Vafaei A, Maciel AC. Early maternal age and multiparity are associated to poor physical performance in middle-aged women from Northeast Brazil: a cross-sectional community based study. BMC women's health. 2015;15:56. Epub 2015/08/06. doi: 10.1186/s12905-015-0214-1. PubMed PMID: 26243283; PubMed Central PMCID: PMCPMC4526418.

**15.** Horne SL, Chen Y, Cockcroft DW, Dosman JA. Risk factors for reduced pulmonary function in women. A possible relationship between Pi phenotype, number of children, and pulmonary function. Chest. 1992;102(1):158-63. Epub 1992/07/01. doi: 10.1378/chest.102.1.158. PubMed PMID: 1623745.

**16.** Krzyzanowski M, Jedrychowski W, Wysocki M. Factors associated with the change in ventilatory function and the development of chronic obstructive pulmonary disease in a 13-year follow-up of the Cracow Study. Risk of chronic obstructive pulmonary disease. The American review of respiratory disease. 1986;134(5):1011-9. Epub 1986/11/01. doi: 10.1164/arrd.1986.134.5.1011. PubMed PMID: 3777663.

**17.** Lemos A, Souza AI, Andrade AD, Figueiroa JN, Cabral-Filho JE. Respiratory muscle strength: comparison between primigravidae and nulligravidae. Jornal brasileiro de pneumologia : publicacao oficial da Sociedade Brasileira de Pneumologia e Tisilogia. 2011;37(2):193-9. Epub 2011/05/04. doi: 10.1590/s1806-37132011000200009. PubMed PMID: 21537655.

**18.** Pirkle CM, de Albuquerque Sousa AC, Alvarado B, Zunzunegui MV, Group IR. Early maternal age at first birth is associated with chronic diseases and poor physical performance in older age: cross-sectional analysis from the International Mobility in Aging Study. BMC Public Health. 2014;14(1):293. Epub 2014/04/02. doi: 10.1186/1471-2458-14-293. PubMed PMID: 24684705; PubMed Central PMCID: PMCPMC3977880.

**19.** Ben Saad H, Ben Hassen I, Ghannouchi I, Latiri I, Rouatbi S, Escourrou P, et al. 6-Min walk-test data in severe obstructive-sleep-apnea-hypopnea-syndrome (OSAHS) under continuous-positive-airway-pressure (CPAP) treatment. Respiratory medicine. 2015;109(5):642-55. Epub 2015/03/31. doi: 10.1016/j.rmed.2015.03.001. PubMed PMID: 25820157.

**20.** Ben Saad H, Prefaut C, Tabka Z, Mtir AH, Chemit M, Hassaoune R, et al. 6-minute walk distance in healthy North Africans older than 40 years: influence of parity. Respiratory medicine. 2009;103(1):74-84. Epub 2008/12/02. doi: 10.1016/j.rmed.2008.07.023. PubMed PMID: 19041233.

**21.** Latiri I, Elbey R, Hcini K, Zaoui A, Charfeddine B, Maarouf MR, et al. Six-minute walk test in non-insulin-dependent diabetes mellitus patients living in Northwest Africa. Diabetes, metabolic syndrome and obesity : targets and therapy. 2012;5:227-45. Epub 2012/08/28. doi: 10.2147/DMSO.S28642. PubMed PMID: 22924002; PubMed Central PMCID: PMCPMC3422904.

**22.** Pellegrino R, Viegi G, Brusasco V, Crapo RO, Burgos F, Casaburi R, et al. Interpretative strategies for lung function tests. European Respiratory Journal. 2005;26(5):948-68. Epub 2005/11/03. doi: 10.1183/09031936.05.00035205. PubMed PMID: 16264058.

**23.** Sherrill DL, Lebowitz MD, Knudson RJ, Burrows B. Longitudinal methods for describing the relationship between pulmonary function, respiratory symptoms and smoking in elderly subjects: The Tucson Study. European Respiratory Journal. 1993;6(3):342-8.

**24.** Ben Saad H, Tfifha M, Harrabi I, Tabka Z, Guenard H, Hayot M, et al. Factors influencing pulmonary function in Tunisian women aged 45 years and more. Revue des maladies respiratoires. 2006;23(4 Pt 1):324-38. Epub 2006/11/28. PubMed PMID: 17127908.

**25.** Harik-Khan RI, Wise RA, Fozard JL. Determinants of maximal inspiratory pressure. The Baltimore Longitudinal Study of Aging. American journal of respiratory and critical care medicine. 1998;158(5 Pt 1):1459-64. Epub 1998/11/17. doi: 10.1164/ajrccm.158.5.9712006. PubMed PMID: 9817693.

**26.** Kollias J, Boileau RA, Barlett HL, Buskirk ER. Pulmonary function and physical conditioning in lean and obese subjects. Archives of environmental health. 1972;25(2):146-50. Epub 1972/08/01. PubMed PMID: 5045066.

**27.** Dore MF, Orvoen-Frija E. Respiratory function in the obese subject. Revue de pneumologie clinique. 2002;58(2):73-81. Epub 2002/06/26. PubMed PMID: 12082445.

**28.** Atek M, Traissac P, El Ati J, Laid Y, Aounallah-Skhiri H, Eymard-Duvernay S, et al. Obesity and association with area of residence, gender and socio-economic factors in Algerian and Tunisian adults. PLoS One. 2013;8(10):e75640. Epub 2013/10/12. doi: 10.1371/journal.pone.0075640. PubMed PMID: 24116063; PubMed Central PMCID: PMCPMC3792975.

**29.** Enright PL, Kronmal RA, Higgins M, Schenker M, Haponik EF. Spirometry reference values for women and men 65 to 85 years of age. Cardiovascular health study. The American review of respiratory disease. 1993;147(1):125-33. Epub 1993/01/01. doi: 10.1164/ajrccm/147.1.125. PubMed PMID: 8420405.

**30.** Borsboom GJ, van Pelt W, van Houwelingen HC, van Vianen BG, Schouten JP, Quanjer PH. Diurnal variation in lung function in subgroups from two Dutch populations: consequences for longitudinal analysis. American journal of respiratory and critical care medicine. 1999;159(4 Pt 1):1163-71. Epub 1999/04/08. doi: 10.1164/ajrccm.159.4.9703106. PubMed PMID: 10194161.

**31.** Parker JM, Dillard TA, Phillips YY. Arm span-height relationships in patients referred for spirometry. American journal of respiratory and critical care medicine. 1996;154(2 Pt 1):533-6. Epub 1996/08/01. doi: 10.1164/ajrccm.154.2.8756834. PubMed PMID: 8756834.

**32.** Triebner K, Matulonga B, Johannessen A, Suske S, Benediktsdottir B, Demoly P, et al. Menopause is associated with accelerated lung function decline. American journal of respiratory and critical care medicine. 2017;195(8):1058-65. Epub 2016/12/03. doi: 10.1164/rccm.201605-0968OC. PubMed PMID: 27907454.
